# Ground spiders (Chelicerata, Araneae) of an urban green space: intensive sampling in a protected area of Rome (Italy) reveals a high diversity and new records to the Italian territory

**DOI:** 10.3897/BDJ.12.e122896

**Published:** 2024-06-07

**Authors:** Tommaso Fusco, Simone Fattorini, Lorenzo Fortini, Enrico Ruzzier, Andrea Di Giulio

**Affiliations:** 1 Department of Science, Roma Tre University, Viale G. Marconi 446, Rome, Italy Department of Science, Roma Tre University, Viale G. Marconi 446 Rome Italy; 2 Università dell'Aquila, Dipartimento di Medicina clinica, sanità pubblica, scienze della vita e dell'ambiente, L'Aquila, Italy Università dell'Aquila, Dipartimento di Medicina clinica, sanità pubblica, scienze della vita e dell'ambiente L'Aquila Italy; 3 NBFC, National Biodiversity Future Center, Palermo, Italy NBFC, National Biodiversity Future Center Palermo Italy

**Keywords:** Arachnids, biodiversity, conservation, faunistic, distribution records, Mediterranean, urban fauna

## Abstract

**Background:**

Urbanisation is a rapidly growing global phenomenon leading to habitat destruction, fragmentation and degradation. However, urban areas can offer opportunities for conservation, particularly through the presence of green spaces which can even provide important habitats for imperilled species. Spiders, which play crucial roles in ecosystem functioning, include many species that can successfully exploit urban environments. Placed in the middle of the Mediterranean global biodiversity hotspot, Italy possesses an exceptionally rich spider fauna, yet comprehensive data on urban spider communities are still limited. More information on urban spiders in Italy would be extremely beneficial to support conservation efforts, especially in central and southern Italy, where knowledge on the spider fauna is largely incomplete.

**New information:**

The current study focused on the spider diversity of a large protected area (Appia Antica Regional Park) in urban Rome, Italy. A total of 120 spider species belonging to 83 genera and 28 families were identified, with 70 species being new records to the Province of Rome, 39 to the Latium Region and two (*Pelecopsisdigitulus* Bosmans & Abrous, 1992 and *Palliduphantesarenicola* (Denis, 1964)) to Italy.

Forty-one species were recorded during autumn/winter sampling and 107 in spring/summer. The spider fauna recorded from the study area included about 37% of the total spider fauna known from the Province of Rome, 28% of that of the Latium Region and 7% of the entire Italian territory. The most represented families in terms of species richness were Gnaphosidae and Linyphiidae, which accounted for more than 40% of the sampled fauna. Lycosidae were the most abundant family (29% of captured individuals), followed by Zodariidae (16% of captured individuals), Linyphiidae (13% of captured individuals) and Gnaphosidae (7.5% of captured individuals). From a biogeographical point of view, most of the collected species belonged to chorotypes that extend for large areas across Europe and the Mediterranean. The research highlights the role of urban green spaces as refuges for spiders and the importance of arachnological research in urban areas as sources of information on spider biodiversity at larger scales.

## Introduction

Urbanisation is a rapidly growing phenomenon and its impact on biodiversity is a cause for concern worldwide. Urbanisation often leads to habitat destruction, fragmentation and degradation, which can have adverse effects on species diversity and abundance (Mckinney 2002, Shochat et al. 2004, Mckinney 2006, McDonald et al. 2013, Fattorini 2019, Fenoglio et al. 2020, Peng et al. 2020). Usually, urbanisation causes changes in species composition due to species loss or replacement, especially of rare and specialist species (Niemelä and Kotze 2009, Vergnes et al. 2014, Knop 2016, Piano et al. 2020a). Urbanisation therefore represents a serious threat for biodiversity conservation (Alaruikka et al. 2002, Shochat et al. 2004, McKinney 2008, Luck and Smallbone 2010, Horváth et al. 2014, Piano et al. 2020a, Piano et al. 2020b) and some species are particularly vulnerable to habitat fragmentation or disturbance caused by urban development (McKinney 2008, Fattorini 2011a, Fattorini 2011b, Fattorini 2019, Olivier et al. 2020). However, not all species are adversely affected by urbanisation and it is recognised that urban areas can provide unique opportunities for conservation (Magura et al. 2010, Moorhead and Philpott 2013, Fattorini 2019, Fattorini et al. 2020, Wenzel et al. 2020, Francoeur et al. 2021).

Urban green spaces, such as parks, gardens, street trees and other types of urban vegetation, may represent important habitats for many organisms, including imperilled species (Fattorini 2019). These areas can provide shelter, food and breeding sites for many animal species, including arthropods, reptiles, birds and mammals (Forman 1995, Angold et al. 2006, Jones and Leather 2012, Adler and Tanner 2013, Fattorini and Galassi 2016, Fattorini 2019).

Spiders (Arachnida, Araneae) are one of the most diverse and ecologically important groups of arthropods, playing key roles in maintaining ecosystem functioning (Turnbull 1973, Ratschker and Roth 2000, Paschetta et al. 2013, Horváth et al. 2014, Nyffeler and Birkhofer 2017). They can be found in a variety of environments, from vegetation-rich areas to ecosystems characterised by low productivity and harsh climatic conditions (Nyffeler and Birkhofer 2017, Milano et al. 2021). Spiders are also key organisms in protecting agroecosystems from harmful organisms (Haddad et al. 2004, King and Hardy 2013, Michalko et al. 2019). As top predators are typically more sensitive to fragmentation compared to species of lower trophic levels (Gibb and Hochuli 2002, Langellotto and Denno 2004, Sarthou et al. 2014, Egerer et al. 2017, El‐Sabaawi 2018), understanding spider responses to urbanisation can provide important insights into the impacts of anthropogenic factors on biodiversity and ecosystem functioning (Magura et al. 2010, Horváth et al. 2012).

In general, spiders exhibit high ecological plasticity, which is the reason why many species can successfully exploit urban environments, where they find many trophic resources (Mammola et al. 2018), being one of the arthropod groups that show the highest species richness values in urban green spaces (Braschler et al. 2020, Trigos-Peral et al. 2020). Research on urban spiders is, however, still limited (Nyffeler and Sunderland 2003, Magura et al. 2010, Krehenwinkel and Tautz 2013, Leroy et al. 2013). Some studies have focused specifically on web-producing species (Mammola et al. 2018, Willmott et al. 2019), while others on the ground-dwelling spiders of green areas (Magura et al. 2010, Braschler et al. 2020, Piano et al. 2020a). In general, urban green spaces tend to be characterised by a high incidence of the most generalist species as anthropogenic factors (e.g. the presence of human structure) negatively affects specialist species, thus reducing species richness (Gibb and Hochuli 2002, Trigos-Peral et al. 2020). However, species richness is also influenced by the size of these green spaces, which – if sufficiently large and ecologically diversified – might in fact represent potentially important areas for biodiversity conservation in urban contexts (Peng et al. 2020).

Despite the high diversity of the spider fauna of Italy (with about 1700 recorded species, Pantini and Isaia 2019, Nentwig et al. 2024), urban spider communities in Italy are still poorly documented (Hansen 1988, Hansen 1992, Hansen 1995, Hansen 1996, Arnò et al. 1998, Giordano et al. 2002, Pilon et al. 2010, Mammola et al. 2018, Piano et al. 2020a, Piano et al. 2020b, Ballarin and Petri 2021). This lack of knowledge is particularly unfortunate, given the prominent importance of the Italian territory within the Mediterranean biodiversity hotspot (Médail and Quezél 1999, Perret et al. 2023). Italy is a country characterised by a rich mosaic of landscapes and high endemism due to its complex climatic, topographic and geological setting (European Environment Agency 2023, Nentwig et al. 2024). Knowledge on spider distribution and community structure in Italy is, however, still largely incomplete, especially in central and southern regions (Pantini and Isaia 2019, Picchi 2020) and more research is urgently needed to provide information for appropriate conservation actions (Branco and Cardoso 2020, Milano et al. 2021, Milano et al. 2022).

The urban fauna of Rome (the largest Italian city) has provided many occasions for research addressing a variety of ecological issues (Piattella et al. 1999, Fattorini 2011a, Fattorini 2011b, Fattorini 2013a, Fattorini 2013b, Fattorini 2014a, Fattorini 2014b, Fattorini and Galassi 2016, Fattorini et al. 2018, Fattorini et al. 2020, Di Pietro et al. 2021). However, the spider fauna of this large urban area has been, so far, largely overlooked. The first studies on the araneofauna of Rome date back to the late 19^th^ and early 20^th^ century (Lucas 1869, Pavesi and Pirotta 1878, Antonelli 1911). Subsequently, studies in the area were carried out by Brignoli (Brignoli 1966, Brignoli 1967b, Brignoli 1971, Brignoli 1972, Brignoli 1977, Brignoli 1979a, Brignoli 1979b), Di Franco (Di Franco 1989, Di Franco 1992, Di Franco 1996, Di Franco 1997) and other specialists, but most of these studies consist of point records or studies that refer to a few spider families (Millidge 1977, Gasparo 1996, Gasparo and Di Franco 2008, Pantini and Isaia 2008, Lacasella et al. 2014, Ballarin and Pantini 2020). To contribute to fill this gap, we present here the results of a study aimed at assessing the species diversity of the ground spiders of a large protected green space in urban Rome, the Appia Antica Regional Park. By increasing our understanding of the spider fauna of this protected area, our study has important implications for conservation and management.

## Materials and methods

### Study area

Rome, with approximately 3 million residents, holds the third position amongst all European Union cities, following Berlin (4 million people) and Madrid (3.2 million people). Urban Rome is typically defined as the region within the motorway ring known as the Grande Raccordo Anulare (GRA), which encompasses an area of approximately 360 km^2^ (Fattorini 2011a, Di Pietro et al. 2021). The Appia Antica Regional Park (41°50’00” N – 12°33’00” E) is a protected green space covering roughly 4,580 hectares, extending from Rome's city centre to the surrounding rural zones of the Alban Hills. This protected area includes archaeological sites along the historical Appian Way, from Rome's centre to the 10^th^ Mile, such as the Villa of the Quintilii and the Tombs of Via Latina. The Park also includes the Caffarella Park and the Park of the Aqueducts.

The Appia Antica Regional Park has a highly diversified landscape, with a mosaic of cultivated fields and natural and semi-natural areas. Vegetation is mainly represented by Mediterranean maquis, including species such as *Pistacialentiscus* L., *Rhamnusalaternus* L. and *Euonymuseuropaeus* L. Due to the millenary human presence, ruderal species such as *Sonchusasper* (L.) Hill, *Pteridiumaquilinum* (L.) Kuhn and *Cymbalariamuralis* G.Gaertn., B.Mey. & Scherb., as well as cultivated species, such as *Oleaeuropaea* L. and *Prunusamygdalus* Batsch, are widely distributed. The area also incorporates fragments of wet meadows and ponds, along with watercourses with associated vegetation (*Populusnigra* L., *Salixalba* L., and *Ulmusminor* Mill.). Further information on the vegetation of the study area can be found in Buccomino and Stanisci (2000), Ceschin et al. (2006) and Iamonico (2022).

### Sampling

**Figure 1.**
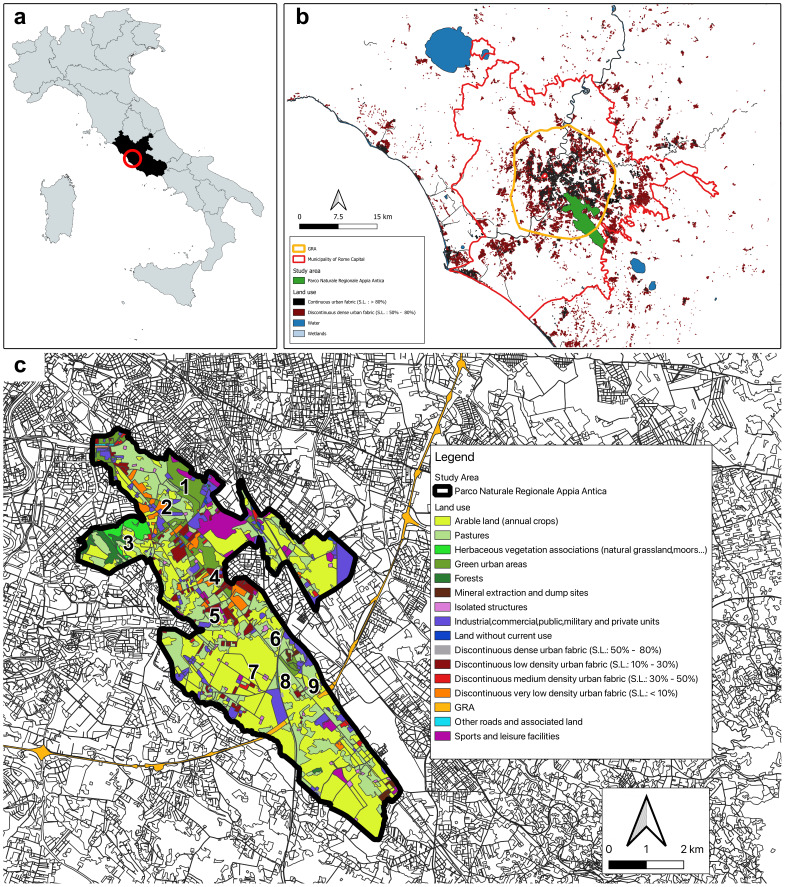
Study area. **a**: Location of Rome Municipality (red circle) within the Italian territory. Latium Regioni is in black. **b**: Location of the study area (in green) within the Rome Municipality (red line). GRA is the Grande Raccordo Anulare, a motorway conventionally used to define the urban area of Rome. S.L. = Sealing Layer. **c**: Land use categories occurring in the study area. Numbers indicate location of sampling sites, numbered according to the urban-rural gradient.

Sampling was conducted in nine sites, distributed along a transect of about 8 km, at increasing distances from the city (Fig. 1): the closest site was at about 5.5 km from the city centre and was surrounded mainly by built-up areas (minimum distance from buildings: 300 m); the most peripherical site was at about 12 km from the city centre, near the GRA, i.e. at the borders of the city. A minimum distance of 500 m separated sampling sites.

**Caffarella Park (Site 1, 19 traps)**: This site is highly heterogeneous, including a small forest area, a pond, areas with sparse vegetation, shrub vegetation, grasslands, riparian vegetation with sparse willow and poplar trees, remnants of old constructions.

**San Sebastiano (Site 2, 7 traps)**: This site corresponds to the archaeological area of catacombs of San Sebastiano, an underground cemetery located along the Via Appia Antica. Traps were placed in the area between the building of the Basilica of San Sebastiano and Via delle Sette Chiese. Vegetation is mainly artificial, including trees and herbs, which are frequently mowed and cut.

**Tor Marancia (Site 3, 7 traps)**: This site is located near a densely populated area with numerous archaeological remains. In 2002, the City Council of Rome and later the Council of Rome decided to establish the Tor Marancia Park as a part of the Appia Antica Park. Vegetation in this site includes a woodland and cultivated fields.

**Acqua Santa (Site 4, 6 traps)**: This site is mainly occupied by open vegetation and wooded edges. The site is partially crossed by Via dell'Acqua Santa, a road with no motorised traffic. Traps were placed close to trees to minimise potential disturbance.

**Farnesiana (Site 5, 5 trap)**: Vegetation in this site was mostly herbaceous, with a predominance of gramineous plants (cultivated fields); a few trees are present in small groups. Ruins of old buildings are also present.

**Cava Fiorucci (Site 6, 7 traps)**: This site (located a few dozen metres south of State Road 7 - via Appia Nuova) has an irregular surface, with depressions and ditches due to past excavation activities. Vegetation includes sporadic bushes and trees; a small gravel road crosses the quarry.

**Casal Verbeni (Site 7, 5 traps)**: This site is characterised by the presence of a few old buildings in the centre of a cultivated area. Trees have been planted between and around the buildings for ornamental purposes and to visually isolate the area from the outside.

**Torre Selce (Site 8, 5 traps)**: This site is adjacent to a section of the original Via Appia and is characterised by the ruins of a tower that stands on a large tumulus from the 1^st^ century. This site includes a small grassy area surrounding the tower, shrubby vegetation and a wooded patch.

**Appia Antica 300 (Site 9, 3 traps)**: Traps were placed near the via Appia Antica, where there are bushes and trees, between the edge of an adjacent cultivated field and the road. The vegetation cover was characterised by a predominance of herbaceous species.

Pitfall traps consisted of clear plastic cups (diameter: 9.5 cm, depth: 15 cm) sunk in the ground with the cup-lip level with the soil surface and covered by sloped stones to limit the rainwater influx and capture of non-target taxa. Covering stones were elevated 5-7 cm above the ground using smaller stones at their corners. Each trap was filled with 250 ml of beer with salt, with a drop of unscented detergent to break the surface tension. The number of pitfall traps varied amongst sites, from a minimum of three traps to a maximum of 19 traps, depending on site habitat heterogeneity. In total, 64 pitfal traps were placed in the study area. Moreover, the number of recovered traps per site varied amongst sampling sessions because of trap damage and trap loss. Traps within sites were separated by at least 10 m from each other.

Sampling was conducted in four autumn sampling periods (from 18 October to 6 December 2013) and four spring sampling periods (from 7 May to 24 June 2014). Each sampling period lasted about ten days. We used this temporal distribution of sampling periods at the turn of two consecutive years because Mediterranean spring climatic conditions and, hence, biotic responses recorded in a given spring-summer are strongly influenced by those of the previous autumn-winter (Fanfani et al. 2014). For each trapping period, traps were active for a minimum of seven days to a maximum of twelve. Variation in trapping duration and time between trapping sessions was due to unstable weather conditions. Upon collection, trap content was rinsed with water and stored in vials with 70% ethanol.

### Sorting and identification

Spiders were identified by the first author on morphological basis under A Zeiss DiscoveryV.12 stereomicroscope using multiple taxonomic keys (Roberts 1987, Trotta 2005, Nentwig et al. 2024). When necessary, female genitals were dissected, observed with an Olympus BX51 microscope and compared with images in Oger (2024). Nomenclature follows the World Spider Catalog (2024). Juveniles were not assigned to a species and a few specimens that were not identifiable due to bad conditions were not considered. All material is preserved in 70% alcohol in the Department of Science of the University of Roma Tre.

### Taxonomic analysis

To describe the taxonomic composition of the ground spider fauna, we calculated the proportion of species in each family and compared these proportions with those that can be obtained at progressively larger scales, i.e. at the province (Rome Province), regional (Latium) and national level (the whole Italian territory), using the data reported in Pantini and Isaia (2019), updated with Trotta (2024), Decae (2024) and the new records given in the current paper. This led to the following values of number of recorded species: Rome Province 322 species, Latium Region 431 species, Italy 1716 species. It is important to take into account that these comparisons are biased by the specific sampling procedure used in our study. We collected only ground-dwelling species, whereas estimates at province, regional and national scale also consider species with different (e.g. arboreal) ecology. Thus, our values are underestimates of the total spider communities. Due to the non-linearity of the species-area relationship (SAR), it is not correct to divide the number of species by the size of the area to compare areas with different size (Fattorini 2021). Assuming that the SAR is best modelled by the power function, *S = cA^z^*, where *S* represents species richness, *A* the area, *c* is the number of species per unit area and *z* is a measure of the rate of change in the slope with increasing area (Lomolino 2001, Fattorini et al. 2017), we calculated the *c*-parameter (*c = S/A^z^)* as a measure of species richness standardised by area (Brummitt and Lughadha 2003, Ovadia 2003). To obtain realistic estimates of relative diversity, it is, however, important to use appropriate values of *z.* For example, for hotspot identification, Ovadia (2003) and Brummitt and Lughadha (2003) used a priori *z*-values such as 0.14 (Brummitt and Lughadha 2003), 0.18 (Ovadia 2003) and 0.25 (Brummitt and Lughadha 2003). Due tothe lack of empirical information on spider SARs in Italy, we conducted analyses using alternatively *z* = 0.14, *z* = 0.18 and *z* = 0.25, as they are representative of the full range of *z*-values commonly found in mainland and island systems (see Fattorini (2021)). As area values, we used *A* = 46 km^2^ for the Appia Antica Regional Park, *A* = 5,363 km^2^ for Rome Province, *A* = 17,232 km^2^ for Latium Region and *A* = 302,073 km^2^ for whole Italian territory.

To study possible variations in taxonomic composition at the assemblage level, we calculated the proportion of species in each family in the nine sampled sites. To express the contribution of each family in terms of abundance, we calculated the proportion of individuals belonging to each family both for the entire fauna of the study area and the nine sampled sites separately.

To describe the biogeographical composition of the Italian spider fauna, species were assigned to chorotypes, i.e. groups of species with similar distributions (Fattorini 2015, Fattorini 2016, Fattorini 2017a, Gatto and Cohn-Haft 2021). Chorotypes are established by an inductive and recursive process in which species distributions are mapped, their contours are compared and species with similar ranges are classified with the same group, i.e. they form a given chorotype (Fattorini 2015, Fattorini 2016, Gatto and Cohn-Haft 2021). After a chorotype is defined by the overlap of multiple species distributions, any other species showing a similar distribution can be assigned to that chorotype. Species that are classified under the same chorotype can belong to completely unrelated taxa or ecological groups. Thus, chorotypes can be compared to the concept of biota of Morrone (2014). As “abstractions” used to express recurrent species distributions, chorotypes are also roughly similar to the “generalised tracks” of Croizat (1958), although, in the case of chorotypes, no shared history is implied. Chorotypes are widely used both to shortly indicate species distributions and to make hypotheses about the origin of plant and animal assemblages (Fattorini 2015, Fattorini 2016, Gatto and Cohn-Haft 2021). As species with similar distributions should also have similar macroecological needs (Olivero et al. 2011), the analysis of the chorotype composition of local species assemblages can be used to draw inferences about which ecological and historical factors have shaped such assemblages.

We extracted species chorotypes from the checklist of Pantini and Isaia (2018), who followed the nomenclature proposed by Vigna Taglianti et al. (1999). For the species not included in Pantini and Isaia (2018) (e.g. new records for the Italian fauna reported here for the first time and other species not included in the checklist) and species for which the known distribution has changed, we examined individual distributions and assigned to each species the respective chorotype following the nomenclature proposed by Vigna Taglianti et al. (1999). For species endemic and subendemic to Italy, Pantini and Isaia (2018) used codes proposed by Vigna Taglianti et al. (1999) for recurrent patterns of species with restricted distributions. We categorised all endemic species as Endemic (END) and assigned subendemic species to the respective main chorotype as follows: Tyrrhenian subendemics were assigned to the West Mediterranean (WME) chorotype; Central-Apenninic, Apennino-Dinaric and Alpino-Apenninic subendemics were assigned to the S-European (SEU) chorotype. Therefore, the word ‘endemic’ is used to indicate the exclusive occurrence of a species in a defined geographical area (the Italian territory in our case) and does not necessarily imply narrow distributions (Fattorini 2017b).

The distribution of each species in the checklist was obtained from World Spider Catalog (2024).

## Checklists

### Spider checklist

#### Agelenidae

C. L. Koch, 1837

1D3F25B2-F544-59DF-8684-FE04D3ED3F08

#### Lycosoides coarcata

(Dufour, 1831)

42A0A00F-9CFF-510D-9A40-C1FD8D3790C8

##### Materials

- **Type status:** Other material. **Occurrence:** recordedBy: Fattorini S., Di Giulio A.; individualCount: 1; sex: male; lifeStage: adult; occurrenceID: 4CA74492-49F3-5C40-9EC0-86A840A1CBA0; **Taxon:** scientificName: Lycosoides coarctata Dufour, 1831); order: Araneae; family: Agelenidae; genus: Lycosoides; **Location:** country: Italy; countryCode: IT; stateProvince: Rome; county: Rome; municipality: Rome; locality: Appia Antica Regional Park, Rome; locationRemarks: Torre Selce; decimalLatitude: 41.816611; decimalLongitude: 12.560667; geodeticDatum: WGS84; **Identification:** identifiedBy: Tommaso Fusco; dateIdentified: 2022; **Event:** samplingProtocol: Pitfall traps; eventDate: 2013-11-07; **Record Level:** collectionID: Roma3_5.8

##### Distribution

Canary Islands, Mediterranean, Georgia, Azerbaijan, Jordan. Mediterranean (MED) chorotype.

#### Tegenaria dalmatica

Kulczyński, 1906

B311A343-CB9B-5DAD-9CAF-9E573030C7A2

##### Materials

- **Type status:** Other material. **Occurrence:** recordedBy: Fattorini S., Di Giulio A.; individualCount: 1; sex: female; lifeStage: adult; occurrenceID: 04C02026-F6BC-5544-B9EA-4F9472838C47; **Taxon:** scientificName: Tegenariadalmatica Kulczyński, 1906; order: Araneae; family: Agelenidae; genus: Tegenaria; **Location:** country: Italy; countryCode: IT; stateProvince: Rome; county: Rome; municipality: Rome; locality: Appia Antica Regional Park, Rome; locationRemarks: Appia Antica; decimalLatitude: 41.812575; decimalLongitude: 12.564011; geodeticDatum: WGS84; **Identification:** identifiedBy: Tommaso Fusco; dateIdentified: 2022; **Event:** samplingProtocol: Pitfall traps; eventDate: 2014-06-05; **Record Level:** collectionID: Roma3_5.8

##### Distribution

Mediterranean to Ukraine. Europeo-Mediterranean (EUM) chorotype.

#### Tegenaria hasperi

Chyzer, 1897

9E630048-0FC3-5CA3-A8B4-8EB401403A71

##### Materials

- **Type status:** Other material. **Occurrence:** recordedBy: Fattorini S., Di Giulio A.; individualCount: 1; sex: male; lifeStage: adult; occurrenceID: 6135AB83-8360-58DF-B7D0-3AC9097B919C; **Taxon:** scientificName: Tegenariahasperi Chyzer, 1897; order: Araneae; family: Agelenidae; genus: Tegenaria; **Location:** country: Italy; countryCode: IT; stateProvince: Rome; county: Rome; municipality: Rome; locality: Appia Antica Regional Park, Rome; locationRemarks: Farnesiana; decimalLatitude: 41.839667; decimalLongitude: 12.525528; geodeticDatum: WGS84; **Identification:** identifiedBy: Tommaso Fusco; dateIdentified: 2022; **Event:** samplingProtocol: Pitfall traps; eventDate: 2014-06-19; **Record Level:** collectionID: Roma3_5.8
- **Type status:** Other material. **Occurrence:** recordedBy: Fattorini S., Di Giulio A.; individualCount: 2; sex: 1 male, 1 female; lifeStage: adult; occurrenceID: 0EAE6E6C-23F6-5DBE-8DFB-A42BA52EFB11; **Taxon:** scientificName: Tegenariahasperi Chyzer, 1897; order: Araneae; family: Agelenidae; genus: Tegenaria; **Location:** country: Italy; countryCode: IT; stateProvince: Rome; county: Rome; municipality: Rome; locality: Appia Antica Regional Park, Rome; locationRemarks: Farnesiana; decimalLatitude: 41.839667; decimalLongitude: 12.525528; geodeticDatum: WGS84; **Identification:** identifiedBy: Tommaso Fusco; dateIdentified: 2022; **Event:** samplingProtocol: Pitfall traps; eventDate: 2014-06-09; **Record Level:** collectionID: Roma3_5.8

##### Distribution

France to Turkey, Russia (Europe, Caucasus). Introduced to Britain. S-European (SEU) chorotype.

#### Amaurobiidae

Thorell, 1869

2AAC71A3-8BE6-5E48-B067-57D0F6DB8F48

#### Amaurobius erberi

(Keyserling, 1863)

C29DC3E5-E4C6-5019-B525-3A0586B0A3EC

##### Materials

- **Type status:** Other material. **Occurrence:** recordedBy: Fattorini S., Di Giulio A.; individualCount: 1; sex: male; lifeStage: adult; occurrenceID: 376F666B-22A9-5794-90FF-860F8F4D1F93; **Taxon:** scientificName: Amaurobiuserberi (Keyserling, 1863); order: Araneae; family: Amaurobiidae; genus: Amaurobius; **Location:** country: Italy; countryCode: IT; stateProvince: Rome; county: Rome; municipality: Rome; locality: Appia Antica Regional Park, Rome; locationRemarks: Appia Antica; decimalLatitude: 41.812575; decimalLongitude: 12.564011; geodeticDatum: WGS84; **Identification:** identifiedBy: Tommaso Fusco; dateIdentified: 2022; **Event:** samplingProtocol: Pitfall traps; eventDate: 2013-11-18; **Record Level:** collectionID: Roma3_5.8
- **Type status:** Other material. **Occurrence:** recordedBy: Fattorini S., Di Giulio A.; individualCount: 2; sex: female; lifeStage: adult; occurrenceID: C91C6B4A-DD25-5279-9FA3-3F8B31C8E7E0; **Taxon:** scientificName: Amaurobiuserberi (Keyserling, 1863); order: Araneae; family: Amaurobiidae; genus: Amaurobius; **Location:** country: Italy; countryCode: IT; stateProvince: Rome; county: Rome; municipality: Rome; locality: Appia Antica Regional Park, Rome; locationRemarks: Casal Verbeni; decimalLatitude: 41.815250; decimalLongitude: 12.552222; geodeticDatum: WGS84; **Identification:** identifiedBy: Tommaso Fusco; dateIdentified: 2022; **Event:** samplingProtocol: Pitfall traps; eventDate: 2013-11-18; **Record Level:** collectionID: Roma3_5.8
- **Type status:** Other material. **Occurrence:** recordedBy: Fattorini S., Di Giulio A.; individualCount: 1; sex: female; lifeStage: adult; occurrenceID: 15BA63C9-8461-526A-BCCD-C4387185AFD8; **Taxon:** scientificName: Amaurobiuserberi (Keyserling, 1863); order: Araneae; family: Amaurobiidae; genus: Amaurobius; **Location:** country: Italy; countryCode: IT; stateProvince: Rome; county: Rome; municipality: Rome; locality: Appia Antica Regional Park, Rome; locationRemarks: Torre Selce; decimalLatitude: 41.816611; decimalLongitude: 12.560667; geodeticDatum: WGS84; **Identification:** identifiedBy: Tommaso Fusco; dateIdentified: 2022; **Event:** samplingProtocol: Pitfall traps; eventDate: 2013-11-18; **Record Level:** collectionID: Roma3_5.8
- **Type status:** Other material. **Occurrence:** recordedBy: Fattorini S., Di Giulio A.; individualCount: 1; sex: male; lifeStage: adult; occurrenceID: B42886CC-3010-5217-8407-998233FA9ACE; **Taxon:** scientificName: Amaurobiuserberi (Keyserling, 1863); order: Araneae; family: Amaurobiidae; genus: Amaurobius; **Location:** country: Italy; countryCode: IT; stateProvince: Rome; county: Rome; municipality: Rome; locality: Appia Antica Regional Park, Rome; locationRemarks: Caffarella centro; decimalLatitude: 41.864889; decimalLongitude: 12.516389; geodeticDatum: WGS84; **Identification:** identifiedBy: Tommaso Fusco; dateIdentified: 2022; **Event:** samplingProtocol: Pitfall traps; eventDate: 2013-11-13; **Record Level:** collectionID: Roma3_5.8
- **Type status:** Other material. **Occurrence:** recordedBy: Fattorini S., Di Giulio A.; individualCount: 4; sex: male; lifeStage: adult; occurrenceID: 6F5720C1-1289-5E5C-BB9D-2319DE32F915; **Taxon:** scientificName: Amaurobiuserberi (Keyserling, 1863); order: Araneae; family: Amaurobiidae; genus: Amaurobius; **Location:** country: Italy; countryCode: IT; stateProvince: Rome; county: Rome; municipality: Rome; locality: Appia Antica Regional Park, Rome; locationRemarks: Casal Verbeni; decimalLatitude: 41.815250; decimalLongitude: 12.552222; geodeticDatum: WGS84; **Identification:** identifiedBy: Tommaso Fusco; dateIdentified: 2022; **Event:** samplingProtocol: Pitfall traps; eventDate: 2013-12-06; **Record Level:** collectionID: Roma3_5.8
- **Type status:** Other material. **Occurrence:** recordedBy: Fattorini S., Di Giulio A.; individualCount: 1; sex: male; lifeStage: adult; occurrenceID: 260F1F5A-2EC2-5A12-8EA3-6024173F1BE7; **Taxon:** scientificName: Amaurobiuserberi (Keyserling, 1863); order: Araneae; family: Amaurobiidae; genus: Amaurobius; **Location:** country: Italy; countryCode: IT; stateProvince: Rome; county: Rome; municipality: Rome; locality: Appia Antica Regional Park, Rome; locationRemarks: Torre Selce; decimalLatitude: 41.816611; decimalLongitude: 12.560667; geodeticDatum: WGS84; **Identification:** identifiedBy: Tommaso Fusco; dateIdentified: 2022; **Event:** samplingProtocol: Pitfall traps; eventDate: 2013-12-06; **Record Level:** collectionID: Roma3_5.8
- **Type status:** Other material. **Occurrence:** recordedBy: Fattorini S., Di Giulio A.; individualCount: 2; sex: 1 male, 1 female; lifeStage: adult; occurrenceID: 67B25115-EA47-5F0C-8645-6B755744222D; **Taxon:** scientificName: Amaurobiuserberi (Keyserling, 1863); order: Araneae; family: Amaurobiidae; genus: Amaurobius; **Location:** country: Italy; countryCode: IT; stateProvince: Rome; county: Rome; municipality: Rome; locality: Appia Antica Regional Park, Rome; locationRemarks: Appia Antica; decimalLatitude: 41.812575; decimalLongitude: 12.564011; geodeticDatum: WGS84; **Identification:** identifiedBy: Tommaso Fusco; dateIdentified: 2022; **Event:** samplingProtocol: Pitfall traps; eventDate: 2013-12-06; **Record Level:** collectionID: Roma3_5.8
- **Type status:** Other material. **Occurrence:** recordedBy: Fattorini S., Di Giulio A.; individualCount: 1; sex: male; lifeStage: adult; occurrenceID: B507B28A-577D-56F1-9BDA-AAC067879719; **Taxon:** scientificName: Amaurobiuserberi (Keyserling, 1863); order: Araneae; family: Amaurobiidae; genus: Amaurobius; **Location:** country: Italy; countryCode: IT; stateProvince: Rome; county: Rome; municipality: Rome; locality: Appia Antica Regional Park, Rome; locationRemarks: Farnesiana; decimalLatitude: 41.839667; decimalLongitude: 12.525528; geodeticDatum: WGS84; **Identification:** identifiedBy: Tommaso Fusco; dateIdentified: 2022; **Event:** samplingProtocol: Pitfall traps; eventDate: 2013-12-02; **Record Level:** collectionID: Roma3_5.8
- **Type status:** Other material. **Occurrence:** recordedBy: Fattorini S., Di Giulio A.; individualCount: 3; sex: male; lifeStage: adult; occurrenceID: 15C46EC5-9827-58D2-A7DD-9AD5B49E4379; **Taxon:** scientificName: Amaurobiuserberi (Keyserling, 1863); order: Araneae; family: Amaurobiidae; genus: Amaurobius; **Location:** country: Italy; countryCode: IT; stateProvince: Rome; county: Rome; municipality: Rome; locality: Appia Antica Regional Park, Rome; locationRemarks: Casal Verbeni; decimalLatitude: 41.815250; decimalLongitude: 12.552222; geodeticDatum: WGS84; **Identification:** identifiedBy: Tommaso Fusco; dateIdentified: 2022; **Event:** samplingProtocol: Pitfall traps; eventDate: 2013-11-07; **Record Level:** collectionID: Roma3_5.8
- **Type status:** Other material. **Occurrence:** recordedBy: Fattorini S., Di Giulio A.; individualCount: 1; sex: male; lifeStage: adult; occurrenceID: 7614302C-9E78-5D8C-8A56-DFFD8C821FBF; **Taxon:** scientificName: Amaurobiuserberi (Keyserling, 1863); order: Araneae; family: Amaurobiidae; genus: Amaurobius; **Location:** country: Italy; countryCode: IT; stateProvince: Rome; county: Rome; municipality: Rome; locality: Appia Antica Regional Park, Rome; locationRemarks: Torre Selce; decimalLatitude: 41.816611; decimalLongitude: 12.560667; geodeticDatum: WGS84; **Identification:** identifiedBy: Tommaso Fusco; dateIdentified: 2022; **Event:** samplingProtocol: Pitfall traps; eventDate: 2013-11-07; **Record Level:** collectionID: Roma3_5.8

##### Distribution

Canary Islands, Algeria, Europe (not in UK and northern Europe), Turkey, Caucasus, Iran. Turano-Europeo-Mediterranean (TEM) chorotype.

#### Anapidae

Simon, 1895

8972DA79-BD50-50F9-B88E-CCC5D54623E4

#### Zangherella algerica

(Simon, 1895)

ED0D4759-BF48-55BF-B31E-A577068B3F9A

##### Materials

- **Type status:** Other material. **Occurrence:** recordedBy: Fattorini S., Di Giulio A.; individualCount: 1; sex: female; lifeStage: adult; occurrenceID: 21FF597A-983D-5891-A4D3-8F7721E8F83D; **Taxon:** scientificName: Zangherellaalgerica (Simon, 1895); order: Araneae; family: Anapidae; genus: Zangherella; **Location:** country: Italy; countryCode: IT; stateProvince: Rome; county: Rome; municipality: Rome; locality: Appia Antica Regional Park, Rome; locationRemarks: Tor Marancia; decimalLatitude: 41.850308; decimalLongitude: 12.503178; geodeticDatum: WGS84; **Identification:** identifiedBy: Tommaso Fusco; dateIdentified: 2022; **Event:** samplingProtocol: Pitfall traps; eventDate: 2014-05-26; **Record Level:** collectionID: Roma3_5.8

##### Distribution

Italy, Algeria, Tunisia. W-Mediterranean (WME) chorotype.

##### Notes

Habitus in Fig. 2

#### Atypidae

Thorell, 1870

356CFD26-E4D6-5E14-B233-124153E1F5E5

#### Atypus affinis

Eichwald, 1830

DDD5181C-FD81-54CB-ABA2-D31D94DC38F5

##### Materials

- **Type status:** Other material. **Occurrence:** recordedBy: Fattorini S., Di Giulio A.; individualCount: 1; sex: male; lifeStage: adult; occurrenceID: B2B9FDAA-D88A-56BB-8D99-DE856C71120E; **Taxon:** scientificName: Atypusaffinis Eichwald, 1830; order: Araneae; family: Atypidae; genus: Atypus; **Location:** country: Italy; countryCode: IT; stateProvince: Rome; county: Rome; municipality: Rome; locality: Appia Antica Regional Park, Rome; locationRemarks: San Sebastiano; decimalLatitude: 41.855733; decimalLongitude: 12.515114; geodeticDatum: WGS84; **Identification:** identifiedBy: Tommaso Fusco; dateIdentified: 2022; **Event:** samplingProtocol: Pitfall traps; eventDate: 2013-11-28; **Record Level:** collectionID: Roma3_5.8
- **Type status:** Other material. **Occurrence:** recordedBy: Fattorini S., Di Giulio A.; individualCount: 1; sex: male; lifeStage: adult; occurrenceID: 3FAB75F9-D187-5139-97D0-10D245A64526; **Taxon:** scientificName: Atypusaffinis Eichwald, 1830; order: Araneae; family: Atypidae; genus: Atypus; **Location:** country: Italy; countryCode: IT; stateProvince: Rome; county: Rome; municipality: Rome; locality: Appia Antica Regional Park, Rome; locationRemarks: San Sebastiano; decimalLatitude: 41.855733; decimalLongitude: 12.515114; geodeticDatum: WGS84; **Identification:** identifiedBy: Tommaso Fusco; dateIdentified: 2022; **Event:** samplingProtocol: Pitfall traps; eventDate: 2013-11-12; **Record Level:** collectionID: Roma3_5.8

##### Distribution

Most of Europe and North Africa. Europeo-Mediterranean (EUM) chorotype.

#### Cheiracanthiidae

Wagner, 1887

928101E9-2FC1-5BAF-B79F-C847032D31C8

#### Cheiracantium mildei

L. Koch, 1864

09C8E1AF-47EF-5309-BBFF-7EC2C3DD2F50

##### Materials

- **Type status:** Other material. **Occurrence:** recordedBy: Fattorini S., Di Giulio A.; individualCount: 1; sex: female; lifeStage: adult; occurrenceID: EBBD7C7E-F93D-5EC0-BF55-E70BE640CCA1; **Taxon:** scientificName: Cheiracanthium mildei L. Koch, 1864; order: Araneae; family: Cheiracanthiidae; genus: Cheiracanthium; **Location:** country: Italy; countryCode: IT; stateProvince: Rome; county: Rome; municipality: Rome; locality: Appia Antica Regional Park, Rome; locationRemarks: Tor Marancia; decimalLatitude: 41.850308; decimalLongitude: 12.503178; geodeticDatum: WGS84; **Identification:** identifiedBy: Tommaso Fusco; dateIdentified: 2022; **Event:** samplingProtocol: Pitfall traps; eventDate: 2014-05-26; **Record Level:** collectionID: Roma3_5.8

##### Distribution

Europe, North Africa, Turkey, Middle East, Caucasus, Russia (Europe) to Central Asia. Introduced to North America, Argentina. Centralasiatic-Europeo-Mediterranean (CEM) chorotype.

#### Dictynidae

O. Pickard-Cambridge, 1871

07A177E1-17D4-5CA3-8AE7-5E4F04F9DA52

#### Argenna subnigra

(O. Pickard-Cambridge, 1861)

24E34195-A375-5E4B-9461-E110EE81BCBB

##### Materials

- **Type status:** Other material. **Occurrence:** recordedBy: Fattorini S., Di Giulio A.; individualCount: 1; sex: male; lifeStage: adult; occurrenceID: 1C4BA699-87C7-543F-9ED2-AE6FCFE75875; **Taxon:** scientificName: Argennasubnigra (O. Pickard-Cambridge, 1861); order: Araneae; family: Dictynidae; genus: Argenna; **Location:** country: Italy; countryCode: IT; stateProvince: Rome; county: Rome; municipality: Rome; locality: Appia Antica Regional Park, Rome; locationRemarks: Acqua Santa; decimalLatitude: 41.850561; decimalLongitude: 12.530861; geodeticDatum: WGS84; **Identification:** identifiedBy: Tommaso Fusco; dateIdentified: 2022; **Event:** samplingProtocol: Pitfall traps; eventDate: 2014-05-28; **Record Level:** collectionID: Roma3_5.8
- **Type status:** Other material. **Occurrence:** recordedBy: Fattorini S., Di Giulio A.; individualCount: 1; sex: male; lifeStage: adult; occurrenceID: 6F022F15-7F1D-5C01-BCBA-A6ED0400AC4F; **Taxon:** scientificName: Argennasubnigra (O. Pickard-Cambridge, 1861); order: Araneae; family: Dictynidae; genus: Argenna; **Location:** country: Italy; countryCode: IT; stateProvince: Rome; county: Rome; municipality: Rome; locality: Appia Antica Regional Park, Rome; locationRemarks: Caffarella Nord; decimalLatitude: 41.867753; decimalLongitude: 12.512414; geodeticDatum: WGS84; **Identification:** identifiedBy: Tommaso Fusco; dateIdentified: 2022; **Event:** samplingProtocol: Pitfall traps; eventDate: 2014-05-27; **Record Level:** collectionID: Roma3_5.8
- **Type status:** Other material. **Occurrence:** recordedBy: Fattorini S., Di Giulio A.; individualCount: 1; sex: male; lifeStage: adult; occurrenceID: 9B0E56DC-A45B-5046-9F8A-4201EF8D1395; **Taxon:** scientificName: Argennasubnigra (O. Pickard-Cambridge, 1861); order: Araneae; family: Dictynidae; genus: Argenna; **Location:** country: Italy; countryCode: IT; stateProvince: Rome; county: Rome; municipality: Rome; locality: Appia Antica Regional Park, Rome; locationRemarks: Caffarella Nord; decimalLatitude: 41.867753; decimalLongitude: 12.512414; geodeticDatum: WGS84; **Identification:** identifiedBy: Tommaso Fusco; dateIdentified: 2022; **Event:** samplingProtocol: Pitfall traps; eventDate: 2014-05-19; **Record Level:** collectionID: Roma3_5.8
- **Type status:** Other material. **Occurrence:** recordedBy: Fattorini S., Di Giulio A.; individualCount: 1; sex: male; lifeStage: adult; occurrenceID: B86F6D10-D95D-548D-81C3-DF9F7A9C0BD4; **Taxon:** scientificName: Argennasubnigra (O. Pickard-Cambridge, 1861); order: Araneae; family: Dictynidae; genus: Argenna; **Location:** country: Italy; countryCode: IT; stateProvince: Rome; county: Rome; municipality: Rome; locality: Appia Antica Regional Park, Rome; locationRemarks: Caffarella Sud 1; decimalLatitude: 41.857247; decimalLongitude: 12.529211; geodeticDatum: WGS84; **Identification:** identifiedBy: Tommaso Fusco; dateIdentified: 2022; **Event:** samplingProtocol: Pitfall traps; eventDate: 2014-05-19; **Record Level:** collectionID: Roma3_5.8
- **Type status:** Other material. **Occurrence:** recordedBy: Fattorini S., Di Giulio A.; individualCount: 1; sex: male; lifeStage: adult; occurrenceID: 9679629C-D809-535A-A2A9-F91B76758AF6; **Taxon:** scientificName: Argennasubnigra (O. Pickard-Cambridge, 1861); order: Araneae; family: Dictynidae; genus: Argenna; **Location:** country: Italy; countryCode: IT; stateProvince: Rome; county: Rome; municipality: Rome; locality: Appia Antica Regional Park, Rome; locationRemarks: Caffarella Sud 2; decimalLatitude: 41.856742; decimalLongitude: 12.529453; geodeticDatum: WGS84; **Identification:** identifiedBy: Tommaso Fusco; dateIdentified: 2022; **Event:** samplingProtocol: Pitfall traps; eventDate: 2014-06-06; **Record Level:** collectionID: Roma3_5.8
- **Type status:** Other material. **Occurrence:** recordedBy: Fattorini S., Di Giulio A.; individualCount: 2; sex: male; lifeStage: adult; occurrenceID: CA21B53A-2E0F-59B4-B448-50E5F1F29A94; **Taxon:** scientificName: Argennasubnigra (O. Pickard-Cambridge, 1861); order: Araneae; family: Dictynidae; genus: Argenna; **Location:** country: Italy; countryCode: IT; stateProvince: Rome; county: Rome; municipality: Rome; locality: Appia Antica Regional Park, Rome; locationRemarks: Caffarella Sud 3; decimalLatitude: 41.856928; decimalLongitude: 12.528406; geodeticDatum: WGS84; **Identification:** identifiedBy: Tommaso Fusco; dateIdentified: 2022; **Event:** samplingProtocol: Pitfall traps; eventDate: 2014-05-19; **Record Level:** collectionID: Roma3_5.8
- **Type status:** Other material. **Occurrence:** recordedBy: Fattorini S., Di Giulio A.; individualCount: 1; sex: male; lifeStage: adult; occurrenceID: BA547D91-C87E-5FA3-8442-FC79AADFDF33; **Taxon:** scientificName: Argennasubnigra (O. Pickard-Cambridge, 1861); order: Araneae; family: Dictynidae; genus: Argenna; **Location:** country: Italy; countryCode: IT; stateProvince: Rome; county: Rome; municipality: Rome; locality: Appia Antica Regional Park, Rome; locationRemarks: Cava Fiorucci; decimalLatitude: 41.834106; decimalLongitude: 12.549264; geodeticDatum: WGS84; **Identification:** identifiedBy: Tommaso Fusco; dateIdentified: 2022; **Event:** samplingProtocol: Pitfall traps; eventDate: 2014-05-26; **Record Level:** collectionID: Roma3_5.8
- **Type status:** Other material. **Occurrence:** recordedBy: Fattorini S., Di Giulio A.; individualCount: 2; sex: male; lifeStage: adult; occurrenceID: 4B9489C5-08AB-5521-BFC4-666F4C78C3C4; **Taxon:** scientificName: Argennasubnigra (O. Pickard-Cambridge, 1861); order: Araneae; family: Dictynidae; genus: Argenna; **Location:** country: Italy; countryCode: IT; stateProvince: Rome; county: Rome; municipality: Rome; locality: Appia Antica Regional Park, Rome; locationRemarks: Farnesiana; decimalLatitude: 41.839667; decimalLongitude: 12.525528; geodeticDatum: WGS84; **Identification:** identifiedBy: Tommaso Fusco; dateIdentified: 2022; **Event:** samplingProtocol: Pitfall traps; eventDate: 2014-05-20; **Record Level:** collectionID: Roma3_5.8
- **Type status:** Other material. **Occurrence:** recordedBy: Fattorini S., Di Giulio A.; individualCount: 1; sex: male; lifeStage: adult; occurrenceID: 63C1C571-A332-5B98-8411-9BE009B75DD7; **Taxon:** scientificName: Argennasubnigra (O. Pickard-Cambridge, 1861); order: Araneae; family: Dictynidae; genus: Argenna; **Location:** country: Italy; countryCode: IT; stateProvince: Rome; county: Rome; municipality: Rome; locality: Appia Antica Regional Park, Rome; locationRemarks: Tor marancia; decimalLatitude: 41.850308; decimalLongitude: 12.503178; geodeticDatum: WGS84; **Identification:** identifiedBy: Tommaso Fusco; dateIdentified: 2022; **Event:** samplingProtocol: Pitfall traps; eventDate: 2014-05-26; **Record Level:** collectionID: Roma3_5.8

##### Distribution

Europe, Caucasus (Russia, Azerbaijan), Iran, China. Asiatic-European (ASE) chorotype.

#### Dysderidae

C. L. Koch, 1837

FC8A34B2-E754-5291-9177-561175DFEC25

#### Dysdera bottazziae

Caporiacco, 1951

A067BE21-C530-5B1F-91F3-05B2171BFB79

##### Materials

- **Type status:** Other material. **Occurrence:** recordedBy: Fattorini S., Di Giulio A.; individualCount: 1; sex: female; lifeStage: adult; occurrenceID: C1BDCF39-514D-535A-B15B-84B920A8CAFF; **Taxon:** scientificName: Dysderabottazziae Caporiacco, 1951; order: Araneae; family: Dysderidae; genus: Dysdera; **Location:** country: Italy; countryCode: IT; stateProvince: Rome; county: Rome; municipality: Rome; locality: Appia Antica Regional Park, Rome; locationRemarks: Appia Antica; decimalLatitude: 41.812575; decimalLongitude: 12.564011; geodeticDatum: WGS84; **Identification:** identifiedBy: Tommaso Fusco; dateIdentified: 2022; **Event:** samplingProtocol: Pitfall traps; eventDate: 2014-06-13; **Record Level:** collectionID: Roma3_5.8
- **Type status:** Other material. **Occurrence:** recordedBy: Fattorini S., Di Giulio A.; individualCount: 1; sex: male; lifeStage: adult; occurrenceID: 48AD8356-4B35-5294-8F87-1EDF8C76EC81; **Taxon:** scientificName: Dysderabottazziae Caporiacco, 1951; order: Araneae; family: Dysderidae; genus: Dysdera; **Location:** country: Italy; countryCode: IT; stateProvince: Rome; county: Rome; municipality: Rome; locality: Appia Antica Regional Park, Rome; locationRemarks: Acqua Santa; decimalLatitude: 41.850561; decimalLongitude: 12.530861; geodeticDatum: WGS84; **Identification:** identifiedBy: Tommaso Fusco; dateIdentified: 2022; **Event:** samplingProtocol: Pitfall traps; eventDate: 2013-11-25; **Record Level:** collectionID: Roma3_5.8
- **Type status:** Other material. **Occurrence:** recordedBy: Fattorini S., Di Giulio A.; individualCount: 1; sex: female; lifeStage: adult; occurrenceID: BADF62B1-BA28-5A49-BA04-47ED2D32B6B3; **Taxon:** scientificName: Dysderabottazziae Caporiacco, 1951; order: Araneae; family: Dysderidae; genus: Dysdera; **Location:** country: Italy; countryCode: IT; stateProvince: Rome; county: Rome; municipality: Rome; locality: Appia Antica Regional Park, Rome; locationRemarks: Caffarella Centro; decimalLatitude: 41.864889; decimalLongitude: 12.516389; geodeticDatum: WGS84; **Identification:** identifiedBy: Tommaso Fusco; dateIdentified: 2022; **Event:** samplingProtocol: Pitfall traps; eventDate: 2013-11-20; **Record Level:** collectionID: Roma3_5.8
- **Type status:** Other material. **Occurrence:** recordedBy: Fattorini S., Di Giulio A.; individualCount: 1; sex: male; lifeStage: adult; occurrenceID: 66BCD524-9381-5021-81CA-123D42123EEF; **Taxon:** scientificName: Dysderabottazziae Caporiacco, 1951; order: Araneae; family: Dysderidae; genus: Dysdera; **Location:** country: Italy; countryCode: IT; stateProvince: Rome; county: Rome; municipality: Rome; locality: Appia Antica Regional Park, Rome; locationRemarks: Farnesiana; decimalLatitude: 41.839667; decimalLongitude: 12.525528; geodeticDatum: WGS84; **Identification:** identifiedBy: Tommaso Fusco; dateIdentified: 2022; **Event:** samplingProtocol: Pitfall traps; eventDate: 2014-06-19; **Record Level:** collectionID: Roma3_5.8
- **Type status:** Other material. **Occurrence:** recordedBy: Fattorini S., Di Giulio A.; individualCount: 1; sex: female; lifeStage: adult; occurrenceID: 0A45305F-515C-5D89-86C2-98013797235C; **Taxon:** scientificName: Dysderabottazziae Caporiacco, 1951; order: Araneae; family: Dysderidae; genus: Dysdera; **Location:** country: Italy; countryCode: IT; stateProvince: Rome; county: Rome; municipality: Rome; locality: Appia Antica Regional Park, Rome; locationRemarks: Acqua Santa; decimalLatitude: 41.850561; decimalLongitude: 12.530861; geodeticDatum: WGS84; **Identification:** identifiedBy: Tommaso Fusco; dateIdentified: 2022; **Event:** samplingProtocol: Pitfall traps; eventDate: 2013-11-04; **Record Level:** collectionID: Roma3_5.8

##### Distribution

Only known from Italy, Croatia and Bosnia and Herzegovina. S-European (SEU) chorotype.

##### Notes

Habitus and male palp in Fig. 3.

#### Dysdera crocata

C. L. Koch, 1838

36AEE7AC-DE13-58E1-AC04-90D129D50DC2

##### Materials

- **Type status:** Other material. **Occurrence:** recordedBy: Fattorini S., Di Giulio A.; individualCount: 2; sex: 1 male, 1 female; lifeStage: adult; occurrenceID: 490B46A5-11A9-52E8-B0A5-6D934A2549BA; **Taxon:** scientificName: Dysderacrocata C. L. Koch, 1838; order: Araneae; family: Dysderidae; genus: Dysdera; **Location:** country: Italy; countryCode: IT; stateProvince: Rome; county: Rome; municipality: Rome; locality: Appia Antica Regional Park, Rome; locationRemarks: Acqua Santa; decimalLatitude: 41.850561; decimalLongitude: 12.530861; geodeticDatum: WGS84; **Identification:** identifiedBy: Tommaso Fusco; dateIdentified: 2022; **Event:** samplingProtocol: Pitfall traps; eventDate: 2014-05-20; **Record Level:** collectionID: Roma3_5.8
- **Type status:** Other material. **Occurrence:** recordedBy: Fattorini S., Di Giulio A.; individualCount: 2; sex: 1 male, 1 female; lifeStage: adult; occurrenceID: DA37870B-23CF-51B0-A544-7D2B5722185B; **Taxon:** scientificName: Dysderacrocata C. L. Koch, 1838; order: Araneae; family: Dysderidae; genus: Dysdera; **Location:** country: Italy; countryCode: IT; stateProvince: Rome; county: Rome; municipality: Rome; locality: Appia Antica Regional Park, Rome; locationRemarks: Acqua Santa; decimalLatitude: 41.850561; decimalLongitude: 12.530861; geodeticDatum: WGS84; **Identification:** identifiedBy: Tommaso Fusco; dateIdentified: 2022; **Event:** samplingProtocol: Pitfall traps; eventDate: 2014-05-28; **Record Level:** collectionID: Roma3_5.8
- **Type status:** Other material. **Occurrence:** recordedBy: Fattorini S., Di Giulio A.; individualCount: 1; sex: male; lifeStage: adult; occurrenceID: 8D2BDC41-0F84-521A-A55F-AE0E347E4DD9; **Taxon:** scientificName: Dysderacrocata C. L. Koch, 1838; order: Araneae; family: Dysderidae; genus: Dysdera; **Location:** country: Italy; countryCode: IT; stateProvince: Rome; county: Rome; municipality: Rome; locality: Appia Antica Regional Park, Rome; locationRemarks: Acqua Santa; decimalLatitude: 41.850561; decimalLongitude: 12.530861; geodeticDatum: WGS84; **Identification:** identifiedBy: Tommaso Fusco; dateIdentified: 2022; **Event:** samplingProtocol: Pitfall traps; eventDate: 2013-11-25; **Record Level:** collectionID: Roma3_5.8
- **Type status:** Other material. **Occurrence:** recordedBy: Fattorini S., Di Giulio A.; individualCount: 1; sex: male; lifeStage: adult; occurrenceID: 312B7EC9-4A28-565D-9D5D-CA4E466882DE; **Taxon:** scientificName: Dysderacrocata C. L. Koch, 1838; order: Araneae; family: Dysderidae; genus: Dysdera; **Location:** country: Italy; countryCode: IT; stateProvince: Rome; county: Rome; municipality: Rome; locality: Appia Antica Regional Park, Rome; locationRemarks: Acqua Santa; decimalLatitude: 41.850561; decimalLongitude: 12.530861; geodeticDatum: WGS84; **Identification:** identifiedBy: Tommaso Fusco; dateIdentified: 2022; **Event:** samplingProtocol: Pitfall traps; eventDate: 2014-06-09; **Record Level:** collectionID: Roma3_5.8
- **Type status:** Other material. **Occurrence:** recordedBy: Fattorini S., Di Giulio A.; individualCount: 2; sex: male; lifeStage: adult; occurrenceID: 4F5260B8-F6BD-5AF7-BD3A-C57FEE1DEB75; **Taxon:** scientificName: Dysderacrocata C. L. Koch, 1838; order: Araneae; family: Dysderidae; genus: Dysdera; **Location:** country: Italy; countryCode: IT; stateProvince: Rome; county: Rome; municipality: Rome; locality: Appia Antica Regional Park, Rome; locationRemarks: Appia Antica; decimalLatitude: 41.812575; decimalLongitude: 12.564011; geodeticDatum: WGS84; **Identification:** identifiedBy: Tommaso Fusco; dateIdentified: 2022; **Event:** samplingProtocol: Pitfall traps; eventDate: 2014-06-13; **Record Level:** collectionID: Roma3_5.8
- **Type status:** Other material. **Occurrence:** recordedBy: Fattorini S., Di Giulio A.; individualCount: 3; sex: 2 male, 1 female; lifeStage: adult; occurrenceID: 86A9506D-DA57-5363-B993-F9EC5C1B6EE7; **Taxon:** scientificName: Dysderacrocata C. L. Koch, 1838; order: Araneae; family: Dysderidae; genus: Dysdera; **Location:** country: Italy; countryCode: IT; stateProvince: Rome; county: Rome; municipality: Rome; locality: Appia Antica Regional Park, Rome; locationRemarks: Caffarella Centro; decimalLatitude: 41.864889; decimalLongitude: 12.516389; geodeticDatum: WGS84; **Identification:** identifiedBy: Tommaso Fusco; dateIdentified: 2022; **Event:** samplingProtocol: Pitfall traps; eventDate: 2013-11-13; **Record Level:** collectionID: Roma3_5.8
- **Type status:** Other material. **Occurrence:** recordedBy: Fattorini S., Di Giulio A.; individualCount: 1; sex: male; lifeStage: adult; occurrenceID: CF945601-529F-5AA4-820D-DA275CFA2D29; **Taxon:** scientificName: Dysderacrocata C. L. Koch, 1838; order: Araneae; family: Dysderidae; genus: Dysdera; **Location:** country: Italy; countryCode: IT; stateProvince: Rome; county: Rome; municipality: Rome; locality: Appia Antica Regional Park, Rome; locationRemarks: Caffarella Centro; decimalLatitude: 41.864889; decimalLongitude: 12.516389; geodeticDatum: WGS84; **Identification:** identifiedBy: Tommaso Fusco; dateIdentified: 2022; **Event:** samplingProtocol: Pitfall traps; eventDate: 2014-05-27; **Record Level:** collectionID: Roma3_5.8
- **Type status:** Other material. **Occurrence:** recordedBy: Fattorini S., Di Giulio A.; individualCount: 1; sex: male; lifeStage: adult; occurrenceID: D62C9667-D223-5EE3-B5AF-7150D7257A78; **Taxon:** scientificName: Dysderacrocata C. L. Koch, 1838; order: Araneae; family: Dysderidae; genus: Dysdera; **Location:** country: Italy; countryCode: IT; stateProvince: Rome; county: Rome; municipality: Rome; locality: Appia Antica Regional Park, Rome; locationRemarks: Caffarella Centro; decimalLatitude: 41.864889; decimalLongitude: 12.516389; geodeticDatum: WGS84; **Identification:** identifiedBy: Tommaso Fusco; dateIdentified: 2022; **Event:** samplingProtocol: Pitfall traps; eventDate: 2014-06-06; **Record Level:** collectionID: Roma3_5.8
- **Type status:** Other material. **Occurrence:** recordedBy: Fattorini S., Di Giulio A.; individualCount: 1; sex: female; lifeStage: adult; occurrenceID: 49DAA350-6244-5E18-8432-00171AAF7860; **Taxon:** scientificName: Dysderacrocata C. L. Koch, 1838; order: Araneae; family: Dysderidae; genus: Dysdera; **Location:** country: Italy; countryCode: IT; stateProvince: Rome; county: Rome; municipality: Rome; locality: Appia Antica Regional Park, Rome; locationRemarks: Caffarella Sud; decimalLatitude: 41.857247; decimalLongitude: 12.529211; geodeticDatum: WGS84; **Identification:** identifiedBy: Tommaso Fusco; dateIdentified: 2022; **Event:** samplingProtocol: Pitfall traps; eventDate: 2013-10-31; **Record Level:** collectionID: Roma3_5.8
- **Type status:** Other material. **Occurrence:** recordedBy: Fattorini S., Di Giulio A.; individualCount: 1; sex: female; lifeStage: adult; occurrenceID: 849DF6D4-4BC0-58E0-AFAD-761D0B0200FB; **Taxon:** scientificName: Dysderacrocata C. L. Koch, 1838; order: Araneae; family: Dysderidae; genus: Dysdera; **Location:** country: Italy; countryCode: IT; stateProvince: Rome; county: Rome; municipality: Rome; locality: Appia Antica Regional Park, Rome; locationRemarks: Caffarella Sud 1; decimalLatitude: 41.857247; decimalLongitude: 12.529211; geodeticDatum: WGS84; **Identification:** identifiedBy: Tommaso Fusco; dateIdentified: 2022; **Event:** samplingProtocol: Pitfall traps; eventDate: 2014-06-18; **Record Level:** collectionID: Roma3_5.8
- **Type status:** Other material. **Occurrence:** recordedBy: Fattorini S., Di Giulio A.; individualCount: 1; sex: female; lifeStage: adult; occurrenceID: E60861D3-E210-5D9F-94A9-F255C55593B4; **Taxon:** scientificName: Dysderacrocata C. L. Koch, 1838; order: Araneae; family: Dysderidae; genus: Dysdera; **Location:** country: Italy; countryCode: IT; stateProvince: Rome; county: Rome; municipality: Rome; locality: Appia Antica Regional Park, Rome; locationRemarks: Casal Verbeni; decimalLatitude: 41.815250; decimalLongitude: 12.552222; geodeticDatum: WGS84; **Identification:** identifiedBy: Tommaso Fusco; dateIdentified: 2022; **Event:** samplingProtocol: Pitfall traps; eventDate: 2013-11-07; **Record Level:** collectionID: Roma3_5.8
- **Type status:** Other material. **Occurrence:** recordedBy: Fattorini S., Di Giulio A.; individualCount: 2; sex: male; lifeStage: adult; occurrenceID: B7F55325-0851-5792-88C2-1F96F7F71F10; **Taxon:** scientificName: Dysderacrocata C. L. Koch, 1838; order: Araneae; family: Dysderidae; genus: Dysdera; **Location:** country: Italy; countryCode: IT; stateProvince: Rome; county: Rome; municipality: Rome; locality: Appia Antica Regional Park, Rome; locationRemarks: Casal Verbeni; decimalLatitude: 41.815250; decimalLongitude: 12.552222; geodeticDatum: WGS84; **Identification:** identifiedBy: Tommaso Fusco; dateIdentified: 2022; **Event:** samplingProtocol: Pitfall traps; eventDate: 2014-06-24; **Record Level:** collectionID: Roma3_5.8
- **Type status:** Other material. **Occurrence:** recordedBy: Fattorini S., Di Giulio A.; individualCount: 3; sex: 1 male, 2 female; lifeStage: adult; occurrenceID: 10C336D5-272E-5100-B3E8-AA6F37904178; **Taxon:** scientificName: Dysderacrocata C. L. Koch, 1838; order: Araneae; family: Dysderidae; genus: Dysdera; **Location:** country: Italy; countryCode: IT; stateProvince: Rome; county: Rome; municipality: Rome; locality: Appia Antica Regional Park, Rome; locationRemarks: Casal Verbeni; decimalLatitude: 41.815250; decimalLongitude: 12.552222; geodeticDatum: WGS84; **Identification:** identifiedBy: Tommaso Fusco; dateIdentified: 2022; **Event:** samplingProtocol: Pitfall traps; eventDate: 2013-12-05; **Record Level:** collectionID: Roma3_5.8
- **Type status:** Other material. **Occurrence:** recordedBy: Fattorini S., Di Giulio A.; individualCount: 2; sex: female; lifeStage: adult; occurrenceID: 4059D390-0C4B-5970-A033-48F5C124F1DD; **Taxon:** scientificName: Dysderacrocata C. L. Koch, 1838; order: Araneae; family: Dysderidae; genus: Dysdera; **Location:** country: Italy; countryCode: IT; stateProvince: Rome; county: Rome; municipality: Rome; locality: Appia Antica Regional Park, Rome; locationRemarks: Cava Fiorucci; decimalLatitude: 41.834106; decimalLongitude: 12.549264; geodeticDatum: WGS84; **Identification:** identifiedBy: Tommaso Fusco; dateIdentified: 2022; **Event:** samplingProtocol: Pitfall traps; eventDate: 2014-06-24; **Record Level:** collectionID: Roma3_5.8
- **Type status:** Other material. **Occurrence:** recordedBy: Fattorini S., Di Giulio A.; individualCount: 1; sex: male; lifeStage: adult; occurrenceID: 16D65942-4A35-5649-BCA8-70D4ED871563; **Taxon:** scientificName: Dysderacrocata C. L. Koch, 1838; order: Araneae; family: Dysderidae; genus: Dysdera; **Location:** country: Italy; countryCode: IT; stateProvince: Rome; county: Rome; municipality: Rome; locality: Appia Antica Regional Park, Rome; locationRemarks: Farnesiana; decimalLatitude: 41.839667; decimalLongitude: 12.525528; geodeticDatum: WGS84; **Identification:** identifiedBy: Tommaso Fusco; dateIdentified: 2022; **Event:** samplingProtocol: Pitfall traps; eventDate: 2014-05-26; **Record Level:** collectionID: Roma3_5.8
- **Type status:** Other material. **Occurrence:** recordedBy: Fattorini S., Di Giulio A.; individualCount: 1; sex: female; lifeStage: adult; occurrenceID: 085AE62D-8643-5203-870F-62BE364C1CEA; **Taxon:** scientificName: Dysderacrocata C. L. Koch, 1838; order: Araneae; family: Dysderidae; genus: Dysdera; **Location:** country: Italy; countryCode: IT; stateProvince: Rome; county: Rome; municipality: Rome; locality: Appia Antica Regional Park, Rome; locationRemarks: Farnesiana; decimalLatitude: 41.839667; decimalLongitude: 12.525528; geodeticDatum: WGS84; **Identification:** identifiedBy: Tommaso Fusco; dateIdentified: 2022; **Event:** samplingProtocol: Pitfall traps; eventDate: 2014-05-20; **Record Level:** collectionID: Roma3_5.8
- **Type status:** Other material. **Occurrence:** recordedBy: Fattorini S., Di Giulio A.; individualCount: 1; sex: male; lifeStage: adult; occurrenceID: 32D72959-71B7-5D0E-B138-30534EDE905A; **Taxon:** scientificName: Dysderacrocata C. L. Koch, 1838; order: Araneae; family: Dysderidae; genus: Dysdera; **Location:** country: Italy; countryCode: IT; stateProvince: Rome; county: Rome; municipality: Rome; locality: Appia Antica Regional Park, Rome; locationRemarks: Farnesiana; decimalLatitude: 41.839667; decimalLongitude: 12.525528; geodeticDatum: WGS84; **Identification:** identifiedBy: Tommaso Fusco; dateIdentified: 2022; **Event:** samplingProtocol: Pitfall traps; eventDate: 2013-11-25; **Record Level:** collectionID: Roma3_5.8
- **Type status:** Other material. **Occurrence:** recordedBy: Fattorini S., Di Giulio A.; individualCount: 4; sex: 3 male, 1 female; lifeStage: adult; occurrenceID: 5AF16B95-C4D8-5DD3-A445-C1DBDB96458E; **Taxon:** scientificName: Dysderacrocata C. L. Koch, 1838; order: Araneae; family: Dysderidae; genus: Dysdera; **Location:** country: Italy; countryCode: IT; stateProvince: Rome; county: Rome; municipality: Rome; locality: Appia Antica Regional Park, Rome; locationRemarks: Farnesiana; decimalLatitude: 41.839667; decimalLongitude: 12.525528; geodeticDatum: WGS84; **Identification:** identifiedBy: Tommaso Fusco; dateIdentified: 2022; **Event:** samplingProtocol: Pitfall traps; eventDate: 2014-06-09; **Record Level:** collectionID: Roma3_5.8
- **Type status:** Other material. **Occurrence:** recordedBy: Fattorini S., Di Giulio A.; individualCount: 1; sex: male; lifeStage: adult; occurrenceID: CB0D8622-CF7A-59E7-B6AC-483FEACD78C8; **Taxon:** scientificName: Dysderacrocata C. L. Koch, 1838; order: Araneae; family: Dysderidae; genus: Dysdera; **Location:** country: Italy; countryCode: IT; stateProvince: Rome; county: Rome; municipality: Rome; locality: Appia Antica Regional Park, Rome; locationRemarks: Farnesiana; decimalLatitude: 41.839667; decimalLongitude: 12.525528; geodeticDatum: WGS84; **Identification:** identifiedBy: Tommaso Fusco; dateIdentified: 2022; **Event:** samplingProtocol: Pitfall traps; eventDate: 2014-06-19; **Record Level:** collectionID: Roma3_5.8
- **Type status:** Other material. **Occurrence:** recordedBy: Fattorini S., Di Giulio A.; individualCount: 1; sex: female; lifeStage: adult; occurrenceID: 96154FD5-487C-596D-8592-53C42E903DCA; **Taxon:** scientificName: Dysderacrocata C. L. Koch, 1838; order: Araneae; family: Dysderidae; genus: Dysdera; **Location:** country: Italy; countryCode: IT; stateProvince: Rome; county: Rome; municipality: Rome; locality: Appia Antica Regional Park, Rome; locationRemarks: San Sebastiano; decimalLatitude: 41.855733; decimalLongitude: 12.515114; geodeticDatum: WGS84; **Identification:** identifiedBy: Tommaso Fusco; dateIdentified: 2022; **Event:** samplingProtocol: Pitfall traps; eventDate: 2013-11-12; **Record Level:** collectionID: Roma3_5.8
- **Type status:** Other material. **Occurrence:** recordedBy: Fattorini S., Di Giulio A.; individualCount: 1; sex: female; lifeStage: adult; occurrenceID: 6B4BC2F7-4839-5AA8-A7AA-9CC6B3E9EE14; **Taxon:** scientificName: Dysderacrocata C. L. Koch, 1838; order: Araneae; family: Dysderidae; genus: Dysdera; **Location:** country: Italy; countryCode: IT; stateProvince: Rome; county: Rome; municipality: Rome; locality: Appia Antica Regional Park, Rome; locationRemarks: San Sebastiano; decimalLatitude: 41.855733; decimalLongitude: 12.515114; geodeticDatum: WGS84; **Identification:** identifiedBy: Tommaso Fusco; dateIdentified: 2022; **Event:** samplingProtocol: Pitfall traps; eventDate: 2014-06-19; **Record Level:** collectionID: Roma3_5.8
- **Type status:** Other material. **Occurrence:** recordedBy: Fattorini S., Di Giulio A.; individualCount: 1; sex: female; lifeStage: adult; occurrenceID: C6773F2E-3D69-5896-A999-948F906CDABD; **Taxon:** scientificName: Dysderacrocata C. L. Koch, 1838; order: Araneae; family: Dysderidae; genus: Dysdera; **Location:** country: Italy; countryCode: IT; stateProvince: Rome; county: Rome; municipality: Rome; locality: Appia Antica Regional Park, Rome; locationRemarks: Tor Marancia; decimalLatitude: 41.850308; decimalLongitude: 12.503178; geodeticDatum: WGS84; **Identification:** identifiedBy: Tommaso Fusco; dateIdentified: 2022; **Event:** samplingProtocol: Pitfall traps; eventDate: 2013-10-28; **Record Level:** collectionID: Roma3_5.8
- **Type status:** Other material. **Occurrence:** recordedBy: Fattorini S., Di Giulio A.; individualCount: 3; sex: male; lifeStage: adult; occurrenceID: 7D9FB3EA-EDC3-58AF-ACE2-541F238C1A2F; **Taxon:** scientificName: Dysderacrocata C. L. Koch, 1838; order: Araneae; family: Dysderidae; genus: Dysdera; **Location:** country: Italy; countryCode: IT; stateProvince: Rome; county: Rome; municipality: Rome; locality: Appia Antica Regional Park, Rome; locationRemarks: Tor Marancia; decimalLatitude: 41.850308; decimalLongitude: 12.503178; geodeticDatum: WGS84; **Identification:** identifiedBy: Tommaso Fusco; dateIdentified: 2022; **Event:** samplingProtocol: Pitfall traps; eventDate: 2014-05-15; **Record Level:** collectionID: Roma3_5.8
- **Type status:** Other material. **Occurrence:** recordedBy: Fattorini S., Di Giulio A.; individualCount: 1; sex: male; lifeStage: adult; occurrenceID: 29025D38-A474-54F6-8157-37E49317FF91; **Taxon:** scientificName: Dysderacrocata C. L. Koch, 1838; order: Araneae; family: Dysderidae; genus: Dysdera; **Location:** country: Italy; countryCode: IT; stateProvince: Rome; county: Rome; municipality: Rome; locality: Appia Antica Regional Park, Rome; locationRemarks: Tor Marancia; decimalLatitude: 41.850308; decimalLongitude: 12.503178; geodeticDatum: WGS84; **Identification:** identifiedBy: Tommaso Fusco; dateIdentified: 2022; **Event:** samplingProtocol: Pitfall traps; eventDate: 2013-11-07; **Record Level:** collectionID: Roma3_5.8
- **Type status:** Other material. **Occurrence:** recordedBy: Fattorini S., Di Giulio A.; individualCount: 1; sex: female; lifeStage: adult; occurrenceID: 0B6C201D-F75C-50B5-8780-442AED982F67; **Taxon:** scientificName: Dysderacrocata C. L. Koch, 1838; order: Araneae; family: Dysderidae; genus: Dysdera; **Location:** country: Italy; countryCode: IT; stateProvince: Rome; county: Rome; municipality: Rome; locality: Appia Antica Regional Park, Rome; locationRemarks: Tor Marancia; decimalLatitude: 41.850308; decimalLongitude: 12.503178; geodeticDatum: WGS84; **Identification:** identifiedBy: Tommaso Fusco; dateIdentified: 2022; **Event:** samplingProtocol: Pitfall traps; eventDate: 2013-11-27; **Record Level:** collectionID: Roma3_5.8
- **Type status:** Other material. **Occurrence:** recordedBy: Fattorini S., Di Giulio A.; individualCount: 1; sex: male; lifeStage: adult; occurrenceID: BF95B566-19B5-5BA2-A422-043AFECA2844; **Taxon:** scientificName: Dysderacrocata C. L. Koch, 1838; order: Araneae; family: Dysderidae; genus: Dysdera; **Location:** country: Italy; countryCode: IT; stateProvince: Rome; county: Rome; municipality: Rome; locality: Appia Antica Regional Park, Rome; locationRemarks: Tor Marancia; decimalLatitude: 41.850308; decimalLongitude: 12.503178; geodeticDatum: WGS84; **Identification:** identifiedBy: Tommaso Fusco; dateIdentified: 2022; **Event:** samplingProtocol: Pitfall traps; eventDate: 2014-06-17; **Record Level:** collectionID: Roma3_5.8
- **Type status:** Other material. **Occurrence:** recordedBy: Fattorini S., Di Giulio A.; individualCount: 1; sex: female; lifeStage: adult; occurrenceID: 047F9895-57E5-579C-B3F3-87ECE22998C2; **Taxon:** scientificName: Dysderacrocata C. L. Koch, 1838; order: Araneae; family: Dysderidae; genus: Dysdera; **Location:** country: Italy; countryCode: IT; stateProvince: Rome; county: Rome; municipality: Rome; locality: Appia Antica Regional Park, Rome; locationRemarks: Torre Selce; decimalLatitude: 41.816611; decimalLongitude: 12.560667; geodeticDatum: WGS84; **Identification:** identifiedBy: Tommaso Fusco; dateIdentified: 2022; **Event:** samplingProtocol: Pitfall traps; eventDate: 2013-11-07; **Record Level:** collectionID: Roma3_5.8
- **Type status:** Other material. **Occurrence:** recordedBy: Fattorini S., Di Giulio A.; individualCount: 1; sex: male; lifeStage: adult; occurrenceID: B743FC1E-7BB1-5AC1-850C-22F170377438; **Taxon:** scientificName: Dysderacrocata C. L. Koch, 1838; order: Araneae; family: Dysderidae; genus: Dysdera; **Location:** country: Italy; countryCode: IT; stateProvince: Rome; county: Rome; municipality: Rome; locality: Appia Antica Regional Park, Rome; locationRemarks: Torre Selce; decimalLatitude: 41.816611; decimalLongitude: 12.560667; geodeticDatum: WGS84; **Identification:** identifiedBy: Tommaso Fusco; dateIdentified: 2022; **Event:** samplingProtocol: Pitfall traps; eventDate: 2014-06-13; **Record Level:** collectionID: Roma3_5.8
- **Type status:** Other material. **Occurrence:** recordedBy: Fattorini S., Di Giulio A.; individualCount: 2; sex: male; lifeStage: adult; occurrenceID: 0DD66E9B-A369-5011-8135-77E9DDDE1B5F; **Taxon:** scientificName: Dysderacrocata C. L. Koch, 1838; order: Araneae; family: Dysderidae; genus: Dysdera; **Location:** country: Italy; countryCode: IT; stateProvince: Rome; county: Rome; municipality: Rome; locality: Appia Antica Regional Park, Rome; locationRemarks: Torre Selce; decimalLatitude: 41.816611; decimalLongitude: 12.560667; geodeticDatum: WGS84; **Identification:** identifiedBy: Tommaso Fusco; dateIdentified: 2022; **Event:** samplingProtocol: Pitfall traps; eventDate: 2013-11-27; **Record Level:** collectionID: Roma3_5.8
- **Type status:** Other material. **Occurrence:** recordedBy: Fattorini S., Di Giulio A.; individualCount: 1; sex: male; lifeStage: adult; occurrenceID: E28D2B79-A35C-56A6-9C90-3F0EE004FC75; **Taxon:** scientificName: Dysderacrocata C. L. Koch, 1838; order: Araneae; family: Dysderidae; genus: Dysdera; **Location:** country: Italy; countryCode: IT; stateProvince: Rome; county: Rome; municipality: Rome; locality: Appia Antica Regional Park, Rome; locationRemarks: Torre Selce; decimalLatitude: 41.816611; decimalLongitude: 12.560667; geodeticDatum: WGS84; **Identification:** identifiedBy: Tommaso Fusco; dateIdentified: 2022; **Event:** samplingProtocol: Pitfall traps; eventDate: 2014-06-24; **Record Level:** collectionID: Roma3_5.8

##### Distribution

Europe, Turkey, Caucasus, Middle East, Central Asia. Introduced to North America, Chile, Brazil, South Africa, Australia, New Zealand, Hawaii. Cosmopolitan (COS) chorotype.

#### Dysdera kollari

Doblika, 1853

52FED15B-D1A2-5701-8C11-193620F67F79

##### Materials

- **Type status:** Other material. **Occurrence:** recordedBy: Fattorini S., Di Giulio A.; individualCount: 1; sex: male; lifeStage: adult; occurrenceID: 97A416A9-18DB-564C-AFC8-A887C7AFD49F; **Taxon:** scientificName: Dysderakollari Doblika, 1853; order: Araneae; family: Dysderidae; genus: Dysdera; **Location:** country: Italy; countryCode: IT; stateProvince: Rome; county: Rome; municipality: Rome; locality: Appia Antica Regional Park, Rome; locationRemarks: Torre Selce; decimalLatitude: 41.816611; decimalLongitude: 12.560667; geodeticDatum: WGS84; **Identification:** identifiedBy: Tommaso Fusco; dateIdentified: 2022; **Event:** samplingProtocol: Pitfall traps; eventDate: 2013-11-07; **Record Level:** collectionID: Roma3_5.8

##### Distribution

Italy, Malta, Balkans and Turkey. E-Mediterranean (EME) chorotype.

#### Dysdera lantosquensis

Simon, 1882

305E80CE-34EF-5966-87BC-E439F822B37F

##### Materials

- **Type status:** Other material. **Occurrence:** recordedBy: Fattorini S., Di Giulio A.; individualCount: 1; sex: male; lifeStage: adult; occurrenceID: 1D060312-22CF-558B-8FC5-9F9CE450D66F; **Taxon:** scientificName: Dysderalantosquensis Simon, 1882; order: Araneae; family: Dysderidae; genus: Dysdera; **Location:** country: Italy; countryCode: IT; stateProvince: Rome; county: Rome; municipality: Rome; locality: Appia Antica Regional Park, Rome; locationRemarks: Acqua Santa; decimalLatitude: 41.850561; decimalLongitude: 12.530861; geodeticDatum: WGS84; **Identification:** identifiedBy: Tommaso Fusco; dateIdentified: 2022; **Event:** samplingProtocol: Pitfall traps; eventDate: 2014-05-20; **Record Level:** collectionID: Roma3_5.8
- **Type status:** Other material. **Occurrence:** recordedBy: Fattorini S., Di Giulio A.; individualCount: 1; sex: male; lifeStage: adult; occurrenceID: 3864A935-0DE6-5BE9-96D4-7C570D50B9E7; **Taxon:** scientificName: Dysderalantosquensis Simon, 1882; order: Araneae; family: Dysderidae; genus: Dysdera; **Location:** country: Italy; countryCode: IT; stateProvince: Rome; county: Rome; municipality: Rome; locality: Appia Antica Regional Park, Rome; locationRemarks: Acqua Santa; decimalLatitude: 41.850561; decimalLongitude: 12.530861; geodeticDatum: WGS84; **Identification:** identifiedBy: Tommaso Fusco; dateIdentified: 2022; **Event:** samplingProtocol: Pitfall traps; eventDate: 2013-11-13; **Record Level:** collectionID: Roma3_5.8
- **Type status:** Other material. **Occurrence:** recordedBy: Fattorini S., Di Giulio A.; individualCount: 1; sex: female; lifeStage: adult; occurrenceID: AC7752A6-AA54-57E3-B7A4-E0903212DBAE; **Taxon:** scientificName: Dysderalantosquensis Simon, 1882; order: Araneae; family: Dysderidae; genus: Dysdera; **Location:** country: Italy; countryCode: IT; stateProvince: Rome; county: Rome; municipality: Rome; locality: Appia Antica Regional Park, Rome; locationRemarks: Acqua Santa; decimalLatitude: 41.850561; decimalLongitude: 12.530861; geodeticDatum: WGS84; **Identification:** identifiedBy: Tommaso Fusco; dateIdentified: 2022; **Event:** samplingProtocol: Pitfall traps; eventDate: 2014-06-19; **Record Level:** collectionID: Roma3_5.8
- **Type status:** Other material. **Occurrence:** recordedBy: Fattorini S., Di Giulio A.; individualCount: 1; sex: female; lifeStage: adult; occurrenceID: DDDF2587-D14F-5AD3-851C-288D8DA1C0A7; **Taxon:** scientificName: Dysderalantosquensis Simon, 1882; order: Araneae; family: Dysderidae; genus: Dysdera; **Location:** country: Italy; countryCode: IT; stateProvince: Rome; county: Rome; municipality: Rome; locality: Appia Antica Regional Park, Rome; locationRemarks: Caffarella Centro; decimalLatitude: 41.864889; decimalLongitude: 12.516389; geodeticDatum: WGS84; **Identification:** identifiedBy: Tommaso Fusco; dateIdentified: 2022; **Event:** samplingProtocol: Pitfall traps; eventDate: 2014-05-27; **Record Level:** collectionID: Roma3_5.8
- **Type status:** Other material. **Occurrence:** recordedBy: Fattorini S., Di Giulio A.; individualCount: 1; sex: female; lifeStage: adult; occurrenceID: 3A9F27E8-9C36-50B8-831D-6EDB33538ACC; **Taxon:** scientificName: Dysderalantosquensis Simon, 1882; order: Araneae; family: Dysderidae; genus: Dysdera; **Location:** country: Italy; countryCode: IT; stateProvince: Rome; county: Rome; municipality: Rome; locality: Appia Antica Regional Park, Rome; locationRemarks: Caffarella Nord; decimalLatitude: 41.867753; decimalLongitude: 12.512414; geodeticDatum: WGS84; **Identification:** identifiedBy: Tommaso Fusco; dateIdentified: 2022; **Event:** samplingProtocol: Pitfall traps; eventDate: 2014-06-18; **Record Level:** collectionID: Roma3_5.8
- **Type status:** Other material. **Occurrence:** recordedBy: Fattorini S., Di Giulio A.; individualCount: 1; sex: male; lifeStage: adult; occurrenceID: ECFCF3C6-F8BF-5497-8029-798B64269C71; **Taxon:** scientificName: Dysderalantosquensis Simon, 1882; order: Araneae; family: Dysderidae; genus: Dysdera; **Location:** country: Italy; countryCode: IT; stateProvince: Rome; county: Rome; municipality: Rome; locality: Appia Antica Regional Park, Rome; locationRemarks: Casal Verbeni; decimalLatitude: 41.815250; decimalLongitude: 12.552222; geodeticDatum: WGS84; **Identification:** identifiedBy: Tommaso Fusco; dateIdentified: 2022; **Event:** samplingProtocol: Pitfall traps; eventDate: 2014-06-05; **Record Level:** collectionID: Roma3_5.8
- **Type status:** Other material. **Occurrence:** recordedBy: Fattorini S., Di Giulio A.; individualCount: 2; sex: male; lifeStage: adult; occurrenceID: 7B111D10-2AA4-5B2E-A09B-9F42BFDCD3D4; **Taxon:** scientificName: Dysderalantosquensis Simon, 1882; order: Araneae; family: Dysderidae; genus: Dysdera; **Location:** country: Italy; countryCode: IT; stateProvince: Rome; county: Rome; municipality: Rome; locality: Appia Antica Regional Park, Rome; locationRemarks: Casal Verbeni; decimalLatitude: 41.815250; decimalLongitude: 12.552222; geodeticDatum: WGS84; **Identification:** identifiedBy: Tommaso Fusco; dateIdentified: 2022; **Event:** samplingProtocol: Pitfall traps; eventDate: 2013-11-27; **Record Level:** collectionID: Roma3_5.8
- **Type status:** Other material. **Occurrence:** recordedBy: Fattorini S., Di Giulio A.; individualCount: 1; sex: male; lifeStage: adult; occurrenceID: BEA60B1B-77E4-5293-8136-5D12B52B61EF; **Taxon:** scientificName: Dysderalantosquensis Simon, 1882; order: Araneae; family: Dysderidae; genus: Dysdera; **Location:** country: Italy; countryCode: IT; stateProvince: Rome; county: Rome; municipality: Rome; locality: Appia Antica Regional Park, Rome; locationRemarks: Casal Verbeni; decimalLatitude: 41.815250; decimalLongitude: 12.552222; geodeticDatum: WGS84; **Identification:** identifiedBy: Tommaso Fusco; dateIdentified: 2022; **Event:** samplingProtocol: Pitfall traps; eventDate: 2014-06-24; **Record Level:** collectionID: Roma3_5.8
- **Type status:** Other material. **Occurrence:** recordedBy: Fattorini S., Di Giulio A.; individualCount: 1; sex: female; lifeStage: adult; occurrenceID: E1F19640-03D0-5571-9E97-2F9215D31CC7; **Taxon:** scientificName: Dysderalantosquensis Simon, 1882; order: Araneae; family: Dysderidae; genus: Dysdera; **Location:** country: Italy; countryCode: IT; stateProvince: Rome; county: Rome; municipality: Rome; locality: Appia Antica Regional Park, Rome; locationRemarks: Farnesiana; decimalLatitude: 41.839667; decimalLongitude: 12.525528; geodeticDatum: WGS84; **Identification:** identifiedBy: Tommaso Fusco; dateIdentified: 2022; **Event:** samplingProtocol: Pitfall traps; eventDate: 2013-11-04; **Record Level:** collectionID: Roma3_5.8
- **Type status:** Other material. **Occurrence:** recordedBy: Fattorini S., Di Giulio A.; individualCount: 2; sex: male; lifeStage: adult; occurrenceID: C4230F14-3020-50AE-8691-DCBE93D266A9; **Taxon:** scientificName: Dysderalantosquensis Simon, 1882; order: Araneae; family: Dysderidae; genus: Dysdera; **Location:** country: Italy; countryCode: IT; stateProvince: Rome; county: Rome; municipality: Rome; locality: Appia Antica Regional Park, Rome; locationRemarks: Farnesiana; decimalLatitude: 41.839667; decimalLongitude: 12.525528; geodeticDatum: WGS84; **Identification:** identifiedBy: Tommaso Fusco; dateIdentified: 2022; **Event:** samplingProtocol: Pitfall traps; eventDate: 2014-06-19; **Record Level:** collectionID: Roma3_5.8
- **Type status:** Other material. **Occurrence:** recordedBy: Fattorini S., Di Giulio A.; individualCount: 1; sex: female; lifeStage: adult; occurrenceID: 14D8EA8D-1C7D-58D6-9DB6-C185FC721CEE; **Taxon:** scientificName: Dysderalantosquensis Simon, 1882; order: Araneae; family: Dysderidae; genus: Dysdera; **Location:** country: Italy; countryCode: IT; stateProvince: Rome; county: Rome; municipality: Rome; locality: Appia Antica Regional Park, Rome; locationRemarks: San Sebastiano; decimalLatitude: 41.855733; decimalLongitude: 12.515114; geodeticDatum: WGS84; **Identification:** identifiedBy: Tommaso Fusco; dateIdentified: 2022; **Event:** samplingProtocol: Pitfall traps; eventDate: 2014-06-19; **Record Level:** collectionID: Roma3_5.8
- **Type status:** Other material. **Occurrence:** recordedBy: Fattorini S., Di Giulio A.; individualCount: 1; sex: female; lifeStage: adult; occurrenceID: 8889BAA7-532E-5D7C-B823-22029C4FB2B6; **Taxon:** scientificName: Dysderalantosquensis Simon, 1882; order: Araneae; family: Dysderidae; genus: Dysdera; **Location:** country: Italy; countryCode: IT; stateProvince: Rome; county: Rome; municipality: Rome; locality: Appia Antica Regional Park, Rome; locationRemarks: Tor Marancia; decimalLatitude: 41.850308; decimalLongitude: 12.503178; geodeticDatum: WGS84; **Identification:** identifiedBy: Tommaso Fusco; dateIdentified: 2022; **Event:** samplingProtocol: Pitfall traps; eventDate: 2013-10-28; **Record Level:** collectionID: Roma3_5.8
- **Type status:** Other material. **Occurrence:** recordedBy: Fattorini S., Di Giulio A.; individualCount: 2; sex: 1 male, 1 female; lifeStage: adult; occurrenceID: 5899F773-749B-5393-B929-805E1762C729; **Taxon:** scientificName: Dysderalantosquensis Simon, 1882; order: Araneae; family: Dysderidae; genus: Dysdera; **Location:** country: Italy; countryCode: IT; stateProvince: Rome; county: Rome; municipality: Rome; locality: Appia Antica Regional Park, Rome; locationRemarks: Tor marancia; decimalLatitude: 41.850308; decimalLongitude: 12.503178; geodeticDatum: WGS84; **Identification:** identifiedBy: Tommaso Fusco; dateIdentified: 2022; **Event:** samplingProtocol: Pitfall traps; eventDate: 2014-05-27; **Record Level:** collectionID: Roma3_5.8
- **Type status:** Other material. **Occurrence:** recordedBy: Fattorini S., Di Giulio A.; individualCount: 2; sex: 1 male, 1 female; lifeStage: adult; occurrenceID: 73CA6810-2424-5F8C-BA6B-0A422F1E12B1; **Taxon:** scientificName: Dysderalantosquensis Simon, 1882; order: Araneae; family: Dysderidae; genus: Dysdera; **Location:** country: Italy; countryCode: IT; stateProvince: Rome; county: Rome; municipality: Rome; locality: Appia Antica Regional Park, Rome; locationRemarks: Tor Marancia; decimalLatitude: 41.850308; decimalLongitude: 12.503178; geodeticDatum: WGS84; **Identification:** identifiedBy: Tommaso Fusco; dateIdentified: 2022; **Event:** samplingProtocol: Pitfall traps; eventDate: 2013-11-18; **Record Level:** collectionID: Roma3_5.8
- **Type status:** Other material. **Occurrence:** recordedBy: Fattorini S., Di Giulio A.; individualCount: 2; sex: male; lifeStage: adult; occurrenceID: 71FC5673-F25A-5E5F-955F-F2337328FEB6; **Taxon:** scientificName: Dysderalantosquensis Simon, 1882; order: Araneae; family: Dysderidae; genus: Dysdera; **Location:** country: Italy; countryCode: IT; stateProvince: Rome; county: Rome; municipality: Rome; locality: Appia Antica Regional Park, Rome; locationRemarks: Tor Marancia; decimalLatitude: 41.850308; decimalLongitude: 12.503178; geodeticDatum: WGS84; **Identification:** identifiedBy: Tommaso Fusco; dateIdentified: 2022; **Event:** samplingProtocol: Pitfall traps; eventDate: 2014-06-17; **Record Level:** collectionID: Roma3_5.8

##### Distribution

France (including Corsica), Italy. S-European (SEU) chorotype.

#### Dysdera romana

Gasparo & Di Franco, 2008

F7660D24-6CA9-5690-A1EA-453D8FBBB032

##### Materials

- **Type status:** Other material. **Occurrence:** recordedBy: Fattorini S., Di Giulio A.; individualCount: 1; sex: male; lifeStage: adult; occurrenceID: 49BF2809-8474-5857-BD53-D436BCEB0D83; **Taxon:** scientificName: Dysderaromana Gasparo & Di Franco, 2008; order: Araneae; family: Dysderidae; genus: Dysdera; **Location:** country: Italy; countryCode: IT; stateProvince: Rome; county: Rome; municipality: Rome; locality: Appia Antica Regional Park, Rome; locationRemarks: Cava Fiorucci; decimalLatitude: 41.834106; decimalLongitude: 12.549264; geodeticDatum: WGS84; **Identification:** identifiedBy: Tommaso Fusco; dateIdentified: 2022; **Event:** samplingProtocol: Pitfall traps; eventDate: 2014-06-13; **Record Level:** collectionID: Roma3_5.8

##### Distribution

Endemic (END) species only known from a few localities in Lazio (Gasparo and Di Franco 2008) and Emilia-Romagna (Lami et al. 2023).

##### Notes

Habitus and male palp in Figs 4, 5

#### Harpactea sardoa

Alicata, 1966

8AFFF787-CA5F-51F7-BECB-BD6FA89601E5

##### Materials

- **Type status:** Other material. **Occurrence:** recordedBy: Fattorini S., Di Giulio A.; individualCount: 1; sex: male; lifeStage: adult; occurrenceID: EDE87968-CDCF-5991-98CC-13417B28332F; **Taxon:** scientificName: Harpacteasardoa Alicata, 1966; order: Araneae; family: Dysderidae; genus: Harpactea; **Location:** country: Italy; countryCode: IT; stateProvince: Rome; county: Rome; municipality: Rome; locality: Appia Antica Regional Park, Rome; locationRemarks: Caffarella Centro; decimalLatitude: 41.864889; decimalLongitude: 12.516389; geodeticDatum: WGS84; **Identification:** identifiedBy: Tommaso Fusco; dateIdentified: 2022; **Event:** samplingProtocol: Pitfall traps; eventDate: 2013-10-31; **Record Level:** collectionID: Roma3_5.8
- **Type status:** Other material. **Occurrence:** recordedBy: Fattorini S., Di Giulio A.; individualCount: 1; sex: male; lifeStage: adult; occurrenceID: 353380A1-D60D-59AE-B26E-A8D7ABE4D87B; **Taxon:** scientificName: Harpacteasardoa Alicata, 1966; order: Araneae; family: Dysderidae; genus: Harpactea; **Location:** country: Italy; countryCode: IT; stateProvince: Rome; county: Rome; municipality: Rome; locality: Appia Antica Regional Park, Rome; locationRemarks: Caffarella Centro; decimalLatitude: 41.864889; decimalLongitude: 12.516389; geodeticDatum: WGS84; **Identification:** identifiedBy: Tommaso Fusco; dateIdentified: 2022; **Event:** samplingProtocol: Pitfall traps; eventDate: 2014-05-19; **Record Level:** collectionID: Roma3_5.8
- **Type status:** Other material. **Occurrence:** recordedBy: Fattorini S., Di Giulio A.; individualCount: 1; sex: male; lifeStage: adult; occurrenceID: 62B0E227-CEB5-5E4B-B5E0-BD907E8216C6; **Taxon:** scientificName: Harpacteasardoa Alicata, 1966; order: Araneae; family: Dysderidae; genus: Harpactea; **Location:** country: Italy; countryCode: IT; stateProvince: Rome; county: Rome; municipality: Rome; locality: Appia Antica Regional Park, Rome; locationRemarks: Caffarella Nord; decimalLatitude: 41.867753; decimalLongitude: 12.512414; geodeticDatum: WGS84; **Identification:** identifiedBy: Tommaso Fusco; dateIdentified: 2022; **Event:** samplingProtocol: Pitfall traps; eventDate: 2013-10-31; **Record Level:** collectionID: Roma3_5.8
- **Type status:** Other material. **Occurrence:** recordedBy: Fattorini S., Di Giulio A.; individualCount: 3; sex: male; lifeStage: adult; occurrenceID: D21ED366-A94D-56F1-AF9F-292D630731A1; **Taxon:** scientificName: Harpacteasardoa Alicata, 1966; order: Araneae; family: Dysderidae; genus: Harpactea; **Location:** country: Italy; countryCode: IT; stateProvince: Rome; county: Rome; municipality: Rome; locality: Appia Antica Regional Park, Rome; locationRemarks: Caffarella Nord; decimalLatitude: 41.867753; decimalLongitude: 12.512414; geodeticDatum: WGS84; **Identification:** identifiedBy: Tommaso Fusco; dateIdentified: 2022; **Event:** samplingProtocol: Pitfall traps; eventDate: 2014-05-19; **Record Level:** collectionID: Roma3_5.8
- **Type status:** Other material. **Occurrence:** recordedBy: Fattorini S., Di Giulio A.; individualCount: 1; sex: male; lifeStage: adult; occurrenceID: 491378F6-2849-563E-AB8E-A108053EAB42; **Taxon:** scientificName: Harpacteasardoa Alicata, 1966; order: Araneae; family: Dysderidae; genus: Harpactea; **Location:** country: Italy; countryCode: IT; stateProvince: Rome; county: Rome; municipality: Rome; locality: Appia Antica Regional Park, Rome; locationRemarks: Cava Fiorucci; decimalLatitude: 41.834106; decimalLongitude: 12.549264; geodeticDatum: WGS84; **Identification:** identifiedBy: Tommaso Fusco; dateIdentified: 2022; **Event:** samplingProtocol: Pitfall traps; eventDate: 2014-05-26; **Record Level:** collectionID: Roma3_5.8
- **Type status:** Other material. **Occurrence:** recordedBy: Fattorini S., Di Giulio A.; individualCount: 1; sex: female; lifeStage: adult; occurrenceID: 5918FEDD-1AF1-5ABE-94C6-E7C5CA770D71; **Taxon:** scientificName: Harpacteasardoa Alicata, 1966; order: Araneae; family: Dysderidae; genus: Harpactea; **Location:** country: Italy; countryCode: IT; stateProvince: Rome; county: Rome; municipality: Rome; locality: Appia Antica Regional Park, Rome; locationRemarks: Cava Fiorucci; decimalLatitude: 41.834106; decimalLongitude: 12.549264; geodeticDatum: WGS84; **Identification:** identifiedBy: Tommaso Fusco; dateIdentified: 2022; **Event:** samplingProtocol: Pitfall traps; eventDate: 2013-11-07; **Record Level:** collectionID: Roma3_5.8
- **Type status:** Other material. **Occurrence:** recordedBy: Fattorini S., Di Giulio A.; individualCount: 3; sex: female; lifeStage: adult; occurrenceID: B544DEB4-5241-5878-82A3-E8C702515A50; **Taxon:** scientificName: Harpacteasardoa Alicata, 1966; order: Araneae; family: Dysderidae; genus: Harpactea; **Location:** country: Italy; countryCode: IT; stateProvince: Rome; county: Rome; municipality: Rome; locality: Appia Antica Regional Park, Rome; locationRemarks: Tor Marancia; decimalLatitude: 41.850308; decimalLongitude: 12.503178; geodeticDatum: WGS84; **Identification:** identifiedBy: Tommaso Fusco; dateIdentified: 2022; **Event:** samplingProtocol: Pitfall traps; eventDate: 2013-10-28; **Record Level:** collectionID: Roma3_5.8
- **Type status:** Other material. **Occurrence:** recordedBy: Fattorini S., Di Giulio A.; individualCount: 5; sex: female; lifeStage: adult; occurrenceID: 65D74FA2-6CC5-5AE0-8520-8CB530A91938; **Taxon:** scientificName: Harpacteasardoa Alicata, 1966; order: Araneae; family: Dysderidae; genus: Harpactea; **Location:** country: Italy; countryCode: IT; stateProvince: Rome; county: Rome; municipality: Rome; locality: Appia Antica Regional Park, Rome; locationRemarks: Tor Marancia; decimalLatitude: 41.850308; decimalLongitude: 12.503178; geodeticDatum: WGS84; **Identification:** identifiedBy: Tommaso Fusco; dateIdentified: 2022; **Event:** samplingProtocol: Pitfall traps; eventDate: 2014-05-15; **Record Level:** collectionID: Roma3_5.8
- **Type status:** Other material. **Occurrence:** recordedBy: Fattorini S., Di Giulio A.; individualCount: 1; sex: female; lifeStage: adult; occurrenceID: 3F964836-07A1-57B9-BCC5-D821341F5956; **Taxon:** scientificName: Harpacteasardoa Alicata, 1966; order: Araneae; family: Dysderidae; genus: Harpactea; **Location:** country: Italy; countryCode: IT; stateProvince: Rome; county: Rome; municipality: Rome; locality: Appia Antica Regional Park, Rome; locationRemarks: Appia Antica; decimalLatitude: 41.812575; decimalLongitude: 12.564011; geodeticDatum: WGS84; **Identification:** identifiedBy: Tommaso Fusco; dateIdentified: 2022; **Event:** samplingProtocol: Pitfall traps; eventDate: 2014-06-05; **Record Level:** collectionID: Roma3_5.8
- **Type status:** Other material. **Occurrence:** recordedBy: Fattorini S., Di Giulio A.; individualCount: 1; sex: male; lifeStage: adult; occurrenceID: 3694DC0D-2A94-5934-A4F2-10610448CB4B; **Taxon:** scientificName: Harpacteasardoa Alicata, 1966; order: Araneae; family: Dysderidae; genus: Harpactea; **Location:** country: Italy; countryCode: IT; stateProvince: Rome; county: Rome; municipality: Rome; locality: Appia Antica Regional Park, Rome; locationRemarks: Caffarella Centro; decimalLatitude: 41.864889; decimalLongitude: 12.516389; geodeticDatum: WGS84; **Identification:** identifiedBy: Tommaso Fusco; dateIdentified: 2022; **Event:** samplingProtocol: Pitfall traps; eventDate: 2013-11-13; **Record Level:** collectionID: Roma3_5.8
- **Type status:** Other material. **Occurrence:** recordedBy: Fattorini S., Di Giulio A.; individualCount: 1; sex: male; lifeStage: adult; occurrenceID: B0045DE2-A5DB-5930-822C-B1AD39FEB479; **Taxon:** scientificName: Harpacteasardoa Alicata, 1966; order: Araneae; family: Dysderidae; genus: Harpactea; **Location:** country: Italy; countryCode: IT; stateProvince: Rome; county: Rome; municipality: Rome; locality: Appia Antica Regional Park, Rome; locationRemarks: Caffarella Centro; decimalLatitude: 41.864889; decimalLongitude: 12.516389; geodeticDatum: WGS84; **Identification:** identifiedBy: Tommaso Fusco; dateIdentified: 2022; **Event:** samplingProtocol: Pitfall traps; eventDate: 2014-05-27; **Record Level:** collectionID: Roma3_5.8
- **Type status:** Other material. **Occurrence:** recordedBy: Fattorini S., Di Giulio A.; individualCount: 3; sex: male; lifeStage: adult; occurrenceID: 78352996-AD35-5D04-B344-E0B6F7B1FF1B; **Taxon:** scientificName: Harpacteasardoa Alicata, 1966; order: Araneae; family: Dysderidae; genus: Harpactea; **Location:** country: Italy; countryCode: IT; stateProvince: Rome; county: Rome; municipality: Rome; locality: Appia Antica Regional Park, Rome; locationRemarks: Caffarella Nord; decimalLatitude: 41.867753; decimalLongitude: 12.512414; geodeticDatum: WGS84; **Identification:** identifiedBy: Tommaso Fusco; dateIdentified: 2022; **Event:** samplingProtocol: Pitfall traps; eventDate: 2014-05-27; **Record Level:** collectionID: Roma3_5.8
- **Type status:** Other material. **Occurrence:** recordedBy: Fattorini S., Di Giulio A.; individualCount: 1; sex: male; lifeStage: adult; occurrenceID: E0DC19E3-BD53-5773-9C8D-F231C90FF369; **Taxon:** scientificName: Harpacteasardoa Alicata, 1966; order: Araneae; family: Dysderidae; genus: Harpactea; **Location:** country: Italy; countryCode: IT; stateProvince: Rome; county: Rome; municipality: Rome; locality: Appia Antica Regional Park, Rome; locationRemarks: San Sebastiano; decimalLatitude: 41.855733; decimalLongitude: 12.515114; geodeticDatum: WGS84; **Identification:** identifiedBy: Tommaso Fusco; dateIdentified: 2022; **Event:** samplingProtocol: Pitfall traps; eventDate: 2014-05-28; **Record Level:** collectionID: Roma3_5.8
- **Type status:** Other material. **Occurrence:** recordedBy: Fattorini S., Di Giulio A.; individualCount: 2; sex: 1 male, 1 female; lifeStage: adult; occurrenceID: 01A3269A-2332-536B-93BE-02791CEAD4BA; **Taxon:** scientificName: Harpacteasardoa Alicata, 1966; order: Araneae; family: Dysderidae; genus: Harpactea; **Location:** country: Italy; countryCode: IT; stateProvince: Rome; county: Rome; municipality: Rome; locality: Appia Antica Regional Park, Rome; locationRemarks: San Sebastiano; decimalLatitude: 41.855733; decimalLongitude: 12.515114; geodeticDatum: WGS84; **Identification:** identifiedBy: Tommaso Fusco; dateIdentified: 2022; **Event:** samplingProtocol: Pitfall traps; eventDate: 2013-11-12; **Record Level:** collectionID: Roma3_5.8
- **Type status:** Other material. **Occurrence:** recordedBy: Fattorini S., Di Giulio A.; individualCount: 8; sex: 2 male, 6 female; lifeStage: adult; occurrenceID: D2939BCB-3AF9-578B-82E3-931BD8DEF6C6; **Taxon:** scientificName: Harpacteasardoa Alicata, 1966; order: Araneae; family: Dysderidae; genus: Harpactea; **Location:** country: Italy; countryCode: IT; stateProvince: Rome; county: Rome; municipality: Rome; locality: Appia Antica Regional Park, Rome; locationRemarks: Tor marancia; decimalLatitude: 41.850308; decimalLongitude: 12.503178; geodeticDatum: WGS84; **Identification:** identifiedBy: Tommaso Fusco; dateIdentified: 2022; **Event:** samplingProtocol: Pitfall traps; eventDate: 2014-05-27; **Record Level:** collectionID: Roma3_5.8
- **Type status:** Other material. **Occurrence:** recordedBy: Fattorini S., Di Giulio A.; individualCount: 1; sex: male; lifeStage: adult; occurrenceID: 22CECEFD-9527-503A-97CA-67D2D2F8B084; **Taxon:** scientificName: Harpacteasardoa Alicata, 1966; order: Araneae; family: Dysderidae; genus: Harpactea; **Location:** country: Italy; countryCode: IT; stateProvince: Rome; county: Rome; municipality: Rome; locality: Appia Antica Regional Park, Rome; locationRemarks: Caffarella Nord; decimalLatitude: 41.867753; decimalLongitude: 12.512414; geodeticDatum: WGS84; **Identification:** identifiedBy: Tommaso Fusco; dateIdentified: 2022; **Event:** samplingProtocol: Pitfall traps; eventDate: 2014-06-06; **Record Level:** collectionID: Roma3_5.8
- **Type status:** Other material. **Occurrence:** recordedBy: Fattorini S., Di Giulio A.; individualCount: 1; sex: male; lifeStage: adult; occurrenceID: EF7CE7D5-23FE-565D-A22B-2314B6BCCF80; **Taxon:** scientificName: Harpacteasardoa Alicata, 1966; order: Araneae; family: Dysderidae; genus: Harpactea; **Location:** country: Italy; countryCode: IT; stateProvince: Rome; county: Rome; municipality: Rome; locality: Appia Antica Regional Park, Rome; locationRemarks: Cava Fiorucci; decimalLatitude: 41.834106; decimalLongitude: 12.549264; geodeticDatum: WGS84; **Identification:** identifiedBy: Tommaso Fusco; dateIdentified: 2022; **Event:** samplingProtocol: Pitfall traps; eventDate: 2014-06-13; **Record Level:** collectionID: Roma3_5.8
- **Type status:** Other material. **Occurrence:** recordedBy: Fattorini S., Di Giulio A.; individualCount: 3; sex: 2 male, 1 female; lifeStage: adult; occurrenceID: 25351A2D-9E0A-5529-89CA-5DE594558614; **Taxon:** scientificName: Harpacteasardoa Alicata, 1966; order: Araneae; family: Dysderidae; genus: Harpactea; **Location:** country: Italy; countryCode: IT; stateProvince: Rome; county: Rome; municipality: Rome; locality: Appia Antica Regional Park, Rome; locationRemarks: Tor Marancia; decimalLatitude: 41.850308; decimalLongitude: 12.503178; geodeticDatum: WGS84; **Identification:** identifiedBy: Tommaso Fusco; dateIdentified: 2022; **Event:** samplingProtocol: Pitfall traps; eventDate: 2014-06-05; **Record Level:** collectionID: Roma3_5.8
- **Type status:** Other material. **Occurrence:** recordedBy: Fattorini S., Di Giulio A.; individualCount: 1; sex: male; lifeStage: adult; occurrenceID: 4B2D6562-31CC-5A3A-B7E2-F3C3D4511240; **Taxon:** scientificName: Harpacteasardoa Alicata, 1966; order: Araneae; family: Dysderidae; genus: Harpactea; **Location:** country: Italy; countryCode: IT; stateProvince: Rome; county: Rome; municipality: Rome; locality: Appia Antica Regional Park, Rome; locationRemarks: Caffarella Nord; decimalLatitude: 41.867753; decimalLongitude: 12.512414; geodeticDatum: WGS84; **Identification:** identifiedBy: Tommaso Fusco; dateIdentified: 2022; **Event:** samplingProtocol: Pitfall traps; eventDate: 2014-06-18; **Record Level:** collectionID: Roma3_5.8
- **Type status:** Other material. **Occurrence:** recordedBy: Fattorini S., Di Giulio A.; individualCount: 2; sex: 1 male, 1 female; lifeStage: adult; occurrenceID: E46A7C3C-09B6-5089-9B88-2BFF5E93273D; **Taxon:** scientificName: Harpacteasardoa Alicata, 1966; order: Araneae; family: Dysderidae; genus: Harpactea; **Location:** country: Italy; countryCode: IT; stateProvince: Rome; county: Rome; municipality: Rome; locality: Appia Antica Regional Park, Rome; locationRemarks: Cava Fiorucci; decimalLatitude: 41.834106; decimalLongitude: 12.549264; geodeticDatum: WGS84; **Identification:** identifiedBy: Tommaso Fusco; dateIdentified: 2022; **Event:** samplingProtocol: Pitfall traps; eventDate: 2014-06-24; **Record Level:** collectionID: Roma3_5.8
- **Type status:** Other material. **Occurrence:** recordedBy: Fattorini S., Di Giulio A.; individualCount: 1; sex: female; lifeStage: adult; occurrenceID: FE23F3B1-370D-5219-BD85-D0CECCAE9A0D; **Taxon:** scientificName: Harpacteasardoa Alicata, 1966; order: Araneae; family: Dysderidae; genus: Harpactea; **Location:** country: Italy; countryCode: IT; stateProvince: Rome; county: Rome; municipality: Rome; locality: Appia Antica Regional Park, Rome; locationRemarks: San Sebastiano; decimalLatitude: 41.855733; decimalLongitude: 12.515114; geodeticDatum: WGS84; **Identification:** identifiedBy: Tommaso Fusco; dateIdentified: 2022; **Event:** samplingProtocol: Pitfall traps; eventDate: 2014-05-27; **Record Level:** collectionID: Roma3_5.8
- **Type status:** Other material. **Occurrence:** recordedBy: Fattorini S., Di Giulio A.; individualCount: 1; sex: male; lifeStage: adult; occurrenceID: B3BB80B9-B2D2-5DFF-882F-89D2580DC224; **Taxon:** scientificName: Harpacteasardoa Alicata, 1966; order: Araneae; family: Dysderidae; genus: Harpactea; **Location:** country: Italy; countryCode: IT; stateProvince: Rome; county: Rome; municipality: Rome; locality: Appia Antica Regional Park, Rome; locationRemarks: San Sebastiano; decimalLatitude: 41.855733; decimalLongitude: 12.515114; geodeticDatum: WGS84; **Identification:** identifiedBy: Tommaso Fusco; dateIdentified: 2022; **Event:** samplingProtocol: Pitfall traps; eventDate: 2014-06-19; **Record Level:** collectionID: Roma3_5.8
- **Type status:** Other material. **Occurrence:** recordedBy: Fattorini S., Di Giulio A.; individualCount: 1; sex: female; lifeStage: adult; occurrenceID: 35659B46-423E-57F8-BB89-14380827BAA1; **Taxon:** scientificName: Harpacteasardoa Alicata, 1966; order: Araneae; family: Dysderidae; genus: Harpactea; **Location:** country: Italy; countryCode: IT; stateProvince: Rome; county: Rome; municipality: Rome; locality: Appia Antica Regional Park, Rome; locationRemarks: Tor Marancia; decimalLatitude: 41.850308; decimalLongitude: 12.503178; geodeticDatum: WGS84; **Identification:** identifiedBy: Tommaso Fusco; dateIdentified: 2022; **Event:** samplingProtocol: Pitfall traps; eventDate: 2013-11-27; **Record Level:** collectionID: Roma3_5.8
- **Type status:** Other material. **Occurrence:** recordedBy: Fattorini S., Di Giulio A.; individualCount: 4; sex: 2 male, 2 female; lifeStage: adult; occurrenceID: BECAC3D0-1E63-5B38-9D47-27B346DC5341; **Taxon:** scientificName: Harpacteasardoa Alicata, 1966; order: Araneae; family: Dysderidae; genus: Harpactea; **Location:** country: Italy; countryCode: IT; stateProvince: Rome; county: Rome; municipality: Rome; locality: Appia Antica Regional Park, Rome; locationRemarks: Tor Marancia; decimalLatitude: 41.850308; decimalLongitude: 12.503178; geodeticDatum: WGS84; **Identification:** identifiedBy: Tommaso Fusco; dateIdentified: 2022; **Event:** samplingProtocol: Pitfall traps; eventDate: 2014-06-17; **Record Level:** collectionID: Roma3_5.8

##### Distribution

Endemic (END) species known from Latium and Sardinia (Brignoli 1979a, Pantini et al. 2013).

#### Eresidae

C. L. Koch, 1845

B72D47B5-8CED-5CD8-9FE3-CAC56E11454B

#### Eresus kollari

Rossi, 1846

FC6D83F7-13F3-5157-999A-708750E04BD3

##### Materials

- **Type status:** Other material. **Occurrence:** recordedBy: Fattorini S., Di Giulio A.; individualCount: 1; sex: male; lifeStage: adult; occurrenceID: FCBB3822-530B-5AB0-A83E-E036E2F2BEB4; **Taxon:** scientificName: Eresuskollari Rossi, 1846; order: Araneae; family: Eresidae; genus: Eresus; **Location:** country: Italy; countryCode: IT; stateProvince: Rome; county: Rome; municipality: Rome; locality: Appia Antica Regional Park, Rome; locationRemarks: Cava Fiorucci; decimalLatitude: 41.834106; decimalLongitude: 12.549264; geodeticDatum: WGS84; **Identification:** identifiedBy: Tommaso Fusco; dateIdentified: 2022; **Event:** samplingProtocol: Pitfall traps; eventDate: 2014-05-24; **Record Level:** collectionID: Roma3_5.8
- **Type status:** Other material. **Occurrence:** recordedBy: Fattorini S., Di Giulio A.; individualCount: 1; sex: male; lifeStage: adult; occurrenceID: B65C3B9B-F3AB-57A4-BC2E-5B6004FFA91D; **Taxon:** scientificName: Eresuskollari Rossi, 1846; order: Araneae; family: Eresidae; genus: Eresus; **Location:** country: Italy; countryCode: IT; stateProvince: Rome; county: Rome; municipality: Rome; locality: Appia Antica Regional Park, Rome; locationRemarks: Cava Fiorucci; decimalLatitude: 41.834106; decimalLongitude: 12.549264; geodeticDatum: WGS84; **Identification:** identifiedBy: Tommaso Fusco; dateIdentified: 2022; **Event:** samplingProtocol: Pitfall traps; eventDate: 2014-06-13; **Record Level:** collectionID: Roma3_5.8

##### Distribution

Europe, North Africa, Turkey, Caucasus, Iran, China, Korea, Russia. Palaearctic (PAL) chorotype.

#### Gnaphosidae

Pocock, 1898

F3AFC048-7B75-54DD-B972-8A3A2368BB8B

#### Anagraphis ochracea

(L. Koch, 1867)

2098F752-EA95-5564-B62B-11C4270547AF

##### Materials

- **Type status:** Other material. **Occurrence:** recordedBy: Fattorini S., Di Giulio A.; individualCount: 1; sex: male; lifeStage: adult; occurrenceID: C99AEA9D-A888-5494-B0FA-77389F500921; **Taxon:** scientificName: Anagraphisochracea (L. Koch, 1867); order: Araneae; family: Gnaphosidae; genus: Anagraphis; **Location:** country: Italy; countryCode: IT; stateProvince: Rome; county: Rome; municipality: Rome; locality: Appia Antica Regional Park, Rome; locationRemarks: Torre Selce; decimalLatitude: 41.816611; decimalLongitude: 12.560667; geodeticDatum: WGS84; **Identification:** identifiedBy: Tommaso Fusco; dateIdentified: 2022; **Event:** samplingProtocol: Pitfall traps; eventDate: 2014-06-05; **Record Level:** collectionID: Roma3_5.8
- **Type status:** Other material. **Occurrence:** recordedBy: Fattorini S., Di Giulio A.; individualCount: 1; sex: male; lifeStage: adult; occurrenceID: 731197C0-9F8C-5464-BAD3-E59EAE28D139; **Taxon:** scientificName: Anagraphisochracea (L. Koch, 1867); order: Araneae; family: Gnaphosidae; genus: Anagraphis; **Location:** country: Italy; countryCode: IT; stateProvince: Rome; county: Rome; municipality: Rome; locality: Appia Antica Regional Park, Rome; locationRemarks: Farnesiana; decimalLatitude: 41.839667; decimalLongitude: 12.525528; geodeticDatum: WGS84; **Identification:** identifiedBy: Tommaso Fusco; dateIdentified: 2022; **Event:** samplingProtocol: Pitfall traps; eventDate: 2014-05-20; **Record Level:** collectionID: Roma3_5.8

##### Distribution

Italy, Balkans, Turkey, southern Russia. S-European (SEU) chorotype.

#### Haplodrassus dalmatensis

(L. Koch, 1866)

59A2AD57-E5E4-5B98-9D47-2C866402C3C9

##### Materials

- **Type status:** Other material. **Occurrence:** recordedBy: Fattorini S., Di Giulio A.; individualCount: 1; sex: m; lifeStage: adult; occurrenceID: 8C3EBC3B-9453-5A46-9F5B-C57884AC7F1F; **Taxon:** scientificName: Haplodrassusdalmatensis (L. Koch, 1866); order: Araneae; family: Gnaphosidae; genus: Haplodrassus; **Location:** country: Italy; countryCode: IT; stateProvince: Rome; county: Rome; municipality: Rome; locality: Appia Antica Regional Park, Rome; locationRemarks: Appia Antica; decimalLatitude: 41.812575; decimalLongitude: 12.564011; geodeticDatum: WGS84; **Identification:** identifiedBy: Tommaso Fusco; dateIdentified: 2022; **Event:** samplingProtocol: Pitfall traps; eventDate: 2014-06-13; **Record Level:** collectionID: Roma3_5.8
- **Type status:** Other material. **Occurrence:** recordedBy: Fattorini S., Di Giulio A.; individualCount: 1; sex: male; lifeStage: adult; occurrenceID: 0C1A6313-E880-5B00-AB4B-5E1B69278526; **Taxon:** scientificName: Haplodrassusdalmatensis (L. Koch, 1866); order: Araneae; family: Gnaphosidae; genus: Haplodrassus; **Location:** country: Italy; countryCode: IT; stateProvince: Rome; county: Rome; municipality: Rome; locality: Appia Antica Regional Park, Rome; locationRemarks: Appia Antica; decimalLatitude: 41.812575; decimalLongitude: 12.564011; geodeticDatum: WGS84; **Identification:** identifiedBy: Tommaso Fusco; dateIdentified: 2022; **Event:** samplingProtocol: Pitfall traps; eventDate: 2014-06-13; **Record Level:** collectionID: Roma3_5.8
- **Type status:** Other material. **Occurrence:** recordedBy: Fattorini S., Di Giulio A.; individualCount: 1; sex: male; lifeStage: adult; occurrenceID: 11B7C537-6D63-5A2E-B0D7-568E48FD9C98; **Taxon:** scientificName: Haplodrassusdalmatensis (L. Koch, 1866); order: Araneae; family: Gnaphosidae; genus: Haplodrassus; **Location:** country: Italy; countryCode: IT; stateProvince: Rome; county: Rome; municipality: Rome; locality: Appia Antica Regional Park, Rome; locationRemarks: Caffarella Sud 1; decimalLatitude: 41.857247; decimalLongitude: 12.529211; geodeticDatum: WGS84; **Identification:** identifiedBy: Tommaso Fusco; dateIdentified: 2022; **Event:** samplingProtocol: Pitfall traps; eventDate: 2014-06-06; **Record Level:** collectionID: Roma3_5.8
- **Type status:** Other material. **Occurrence:** recordedBy: Fattorini S., Di Giulio A.; individualCount: 1; sex: male; lifeStage: adult; occurrenceID: 8A18E2C9-A55B-5A80-9A5E-6587D22BFE16; **Taxon:** scientificName: Haplodrassusdalmatensis (L. Koch, 1866); order: Araneae; family: Gnaphosidae; genus: Haplodrassus; **Location:** country: Italy; countryCode: IT; stateProvince: Rome; county: Rome; municipality: Rome; locality: Appia Antica Regional Park, Rome; locationRemarks: Caffarella Sud 1; decimalLatitude: 41.857247; decimalLongitude: 12.529211; geodeticDatum: WGS84; **Identification:** identifiedBy: Tommaso Fusco; dateIdentified: 2022; **Event:** samplingProtocol: Pitfall traps; eventDate: 2014-06-18; **Record Level:** collectionID: Roma3_5.8
- **Type status:** Other material. **Occurrence:** recordedBy: Fattorini S., Di Giulio A.; individualCount: 2; sex: male; lifeStage: adult; occurrenceID: 0F9851F1-ACD6-59C3-B29C-1041D1FBAC3B; **Taxon:** scientificName: Haplodrassusdalmatensis (L. Koch, 1866); order: Araneae; family: Gnaphosidae; genus: Haplodrassus; **Location:** country: Italy; countryCode: IT; stateProvince: Rome; county: Rome; municipality: Rome; locality: Appia Antica Regional Park, Rome; locationRemarks: Casal Verbeni; decimalLatitude: 41.815250; decimalLongitude: 12.552222; geodeticDatum: WGS84; **Identification:** identifiedBy: Tommaso Fusco; dateIdentified: 2022; **Event:** samplingProtocol: Pitfall traps; eventDate: 2014-05-26; **Record Level:** collectionID: Roma3_5.8
- **Type status:** Other material. **Occurrence:** recordedBy: Fattorini S., Di Giulio A.; individualCount: 1; sex: female; lifeStage: adult; occurrenceID: D6131766-B2A0-555E-9033-90D51730F410; **Taxon:** scientificName: Haplodrassusdalmatensis (L. Koch, 1866); order: Araneae; family: Gnaphosidae; genus: Haplodrassus; **Location:** country: Italy; countryCode: IT; stateProvince: Rome; county: Rome; municipality: Rome; locality: Appia Antica Regional Park, Rome; locationRemarks: Casal Verbeni; decimalLatitude: 41.815250; decimalLongitude: 12.552222; geodeticDatum: WGS84; **Identification:** identifiedBy: Tommaso Fusco; dateIdentified: 2022; **Event:** samplingProtocol: Pitfall traps; eventDate: 2014-06-13; **Record Level:** collectionID: Roma3_5.8
- **Type status:** Other material. **Occurrence:** recordedBy: Fattorini S., Di Giulio A.; individualCount: 5; sex: 3 male, 2 female; lifeStage: adult; occurrenceID: 1FB0B2F9-7E38-56FF-ABEB-925442161D49; **Taxon:** scientificName: Haplodrassusdalmatensis (L. Koch, 1866); order: Araneae; family: Gnaphosidae; genus: Haplodrassus; **Location:** country: Italy; countryCode: IT; stateProvince: Rome; county: Rome; municipality: Rome; locality: Appia Antica Regional Park, Rome; locationRemarks: Farnesiana; decimalLatitude: 41.839667; decimalLongitude: 12.525528; geodeticDatum: WGS84; **Identification:** identifiedBy: Tommaso Fusco; dateIdentified: 2022; **Event:** samplingProtocol: Pitfall traps; eventDate: 2014-05-20; **Record Level:** collectionID: Roma3_5.8
- **Type status:** Other material. **Occurrence:** recordedBy: Fattorini S., Di Giulio A.; individualCount: 1; sex: male; lifeStage: adult; occurrenceID: 0A2E6144-10D2-5DCC-8EC5-8D8E0A4E9CA2; **Taxon:** scientificName: Haplodrassusdalmatensis (L. Koch, 1866); order: Araneae; family: Gnaphosidae; genus: Haplodrassus; **Location:** country: Italy; countryCode: IT; stateProvince: Rome; county: Rome; municipality: Rome; locality: Appia Antica Regional Park, Rome; locationRemarks: Farnesiana; decimalLatitude: 41.839667; decimalLongitude: 12.525528; geodeticDatum: WGS84; **Identification:** identifiedBy: Tommaso Fusco; dateIdentified: 2022; **Event:** samplingProtocol: Pitfall traps; eventDate: 2014-05-28; **Record Level:** collectionID: Roma3_5.8
- **Type status:** Other material. **Occurrence:** recordedBy: Fattorini S., Di Giulio A.; individualCount: 2; sex: male; lifeStage: adult; occurrenceID: 7FC858F5-50FF-580F-B4CA-5B72E2F6E4BC; **Taxon:** scientificName: Haplodrassusdalmatensis (L. Koch, 1866); order: Araneae; family: Gnaphosidae; genus: Haplodrassus; **Location:** country: Italy; countryCode: IT; stateProvince: Rome; county: Rome; municipality: Rome; locality: Appia Antica Regional Park, Rome; locationRemarks: Farnesiana; decimalLatitude: 41.839667; decimalLongitude: 12.525528; geodeticDatum: WGS84; **Identification:** identifiedBy: Tommaso Fusco; dateIdentified: 2022; **Event:** samplingProtocol: Pitfall traps; eventDate: 2014-06-09; **Record Level:** collectionID: Roma3_5.8

##### Distribution

Europe, North Africa, Turkey, Caucasus, Middle East, Russia (Europe) to Iran. Palaeartic (PAL) chorotype.

#### Haplodrassus signifer

(C. L. Koch, 1839)

008BB560-0080-5D8D-A9F6-2C677F247BE8

##### Materials

- **Type status:** Other material. **Occurrence:** recordedBy: Fattorini S., Di Giulio A.; individualCount: 1; sex: male; lifeStage: adult; occurrenceID: C92D7521-2830-5701-9C50-6F1C9B36B551; **Taxon:** scientificName: Haplodrassussignifer (C. L. Koch, 1839); order: Araneae; family: Gnaphosidae; genus: Haplodrassus; **Location:** country: Italy; countryCode: IT; stateProvince: Rome; county: Rome; municipality: Rome; locality: Appia Antica Regional Park, Rome; locationRemarks: Appia Antica; decimalLatitude: 41.812575; decimalLongitude: 12.564011; geodeticDatum: WGS84; **Identification:** identifiedBy: Tommaso Fusco; dateIdentified: 2022; **Event:** samplingProtocol: Pitfall traps; eventDate: 2014-06-05; **Record Level:** collectionID: Roma3_5.8

##### Distribution

North America, Europe, North Africa, Turkey, Caucasus, Russia, Middle East, Central Asia to Korea. Holarctic (OLA) chorotype.

#### Heser nilicola

(O. Pickard-Cambridge, 1874)

11AAD71D-B3F5-55D1-96F2-D6EC1CB95FBB

##### Materials

- **Type status:** Other material. **Occurrence:** recordedBy: Fattorini S., Di Giulio A.; individualCount: 4; sex: male; lifeStage: adult; occurrenceID: 939B15FF-84DF-5D1D-8D88-A4E78F829188; **Taxon:** scientificName: Hesernilicola (O. Pickard-Cambridge, 1874); order: Araneae; family: Gnaphosidae; genus: Heser; **Location:** country: Italy; countryCode: IT; stateProvince: Rome; county: Rome; municipality: Rome; locality: Appia Antica Regional Park, Rome; locationRemarks: Casal Verbeni; decimalLatitude: 41.815250; decimalLongitude: 12.552222; geodeticDatum: WGS84; **Identification:** identifiedBy: Tommaso Fusco; dateIdentified: 2022; **Event:** samplingProtocol: Pitfall traps; eventDate: 2014-06-13; **Record Level:** collectionID: Roma3_5.8

##### Distribution

Mediterranean, introduced to USA and Mexico. Mediterranean (OLA) chorotype.

#### Leptodrassus albidus

Simon, 1914

7B301FD4-5920-52D6-BD79-6F21762B5D10

##### Materials

- **Type status:** Other material. **Occurrence:** recordedBy: Fattorini S., Di Giulio A.; individualCount: 1; sex: male; lifeStage: adult; occurrenceID: 8787BD14-DE4A-5AE6-BDC4-D5FEA31E9DF7; **Taxon:** scientificName: Leptodrassusalbidus Simon, 1914; order: Araneae; family: Gnaphosidae; genus: Leptodrassus; **Location:** country: Italy; countryCode: IT; stateProvince: Rome; county: Rome; municipality: Rome; locality: Appia Antica Regional Park, Rome; locationRemarks: Farnesiana; decimalLatitude: 41.839667; decimalLongitude: 12.525528; geodeticDatum: WGS84; **Identification:** identifiedBy: Tommaso Fusco; dateIdentified: 2022; **Event:** samplingProtocol: Pitfall traps; eventDate: 2014-06-09; **Record Level:** collectionID: Roma3_5.8

##### Distribution

Azores, Canary Islands, Spain to Greece, Turkey, Israel. Mediterranean (MED) chorotype.

#### Leptodrassus femineus

(Simon, 1873)

96658DAF-7074-5A2A-AC4C-84934B8CC2D1

##### Materials

- **Type status:** Other material. **Occurrence:** recordedBy: Fattorini S., Di Giulio A.; individualCount: 2; sex: female; lifeStage: adult; occurrenceID: 9B499256-B211-5462-A8A8-7608DDCAF0AC; **Taxon:** scientificName: Leptodrassusfemineus (Simon, 1873); order: Araneae; family: Gnaphosidae; genus: Leptodrassus; **Location:** country: Italy; countryCode: IT; stateProvince: Rome; county: Rome; municipality: Rome; locality: Appia Antica Regional Park, Rome; locationRemarks: Cava Fiorucci; decimalLatitude: 41.834106; decimalLongitude: 12.549264; geodeticDatum: WGS84; **Identification:** identifiedBy: Tommaso Fusco; dateIdentified: 2022; **Event:** samplingProtocol: Pitfall traps; eventDate: 2014-06-13; **Record Level:** collectionID: Roma3_5.8

##### Distribution

Portugal to Bulgaria and Greece, Cyprus, Israel. Mediterranean (MED) chorotype.

#### Marinarozelotes adriaticus

(Caporiacco, 1951)

9301CC74-F7C1-5A20-9C73-0829CEBDDC48

##### Materials

- **Type status:** Other material. **Occurrence:** recordedBy: Fattorini S., Di Giulio A.; individualCount: 1; sex: male; lifeStage: adult; occurrenceID: 5AAFE846-2B7B-5BDE-AF55-9FCB50CA5C00; **Taxon:** scientificName: Marinarozelotesadriaticus (Caporiacco, 1951); order: Araneae; family: Gnaphosidae; genus: Marinarozelotes; **Location:** country: Italy; countryCode: IT; stateProvince: Rome; county: Rome; municipality: Rome; locality: Appia Antica Regional Park, Rome; locationRemarks: Cava Fiorucci; decimalLatitude: 41.834106; decimalLongitude: 12.549264; geodeticDatum: WGS84; **Identification:** identifiedBy: Tommaso Fusco; dateIdentified: 2022; **Event:** samplingProtocol: Pitfall traps; eventDate: 2014-06-13; **Record Level:** collectionID: Roma3_5.8
- **Type status:** Other material. **Occurrence:** recordedBy: Fattorini S., Di Giulio A.; individualCount: 1; sex: male; lifeStage: adult; occurrenceID: EDC2E5C4-38C7-5D30-88F2-2AD6EB9176B0; **Taxon:** scientificName: Marinarozelotesadriaticus (Caporiacco, 1951); order: Araneae; family: Gnaphosidae; genus: Marinarozelotes; **Location:** country: Italy; countryCode: IT; stateProvince: Rome; county: Rome; municipality: Rome; locality: Appia Antica Regional Park, Rome; locationRemarks: Cava Fiorucci; decimalLatitude: 41.834106; decimalLongitude: 12.549264; geodeticDatum: WGS84; **Identification:** identifiedBy: Tommaso Fusco; dateIdentified: 2022; **Event:** samplingProtocol: Pitfall traps; eventDate: 2014-06-24; **Record Level:** collectionID: Roma3_5.8
- **Type status:** Other material. **Occurrence:** recordedBy: Fattorini S., Di Giulio A.; individualCount: 1; sex: male; lifeStage: adult; occurrenceID: 0A323196-7C66-5E18-B2C7-8AE2E6C8C31E; **Taxon:** scientificName: Marinarozelotesadriaticus (Caporiacco, 1951); order: Araneae; family: Gnaphosidae; genus: Marinarozelotes; **Location:** country: Italy; countryCode: IT; stateProvince: Rome; county: Rome; municipality: Rome; locality: Appia Antica Regional Park, Rome; locationRemarks: Farnesiana; decimalLatitude: 41.839667; decimalLongitude: 12.525528; geodeticDatum: WGS84; **Identification:** identifiedBy: Tommaso Fusco; dateIdentified: 2022; **Event:** samplingProtocol: Pitfall traps; eventDate: 2014-06-09; **Record Level:** collectionID: Roma3_5.8
- **Type status:** Other material. **Occurrence:** recordedBy: Fattorini S., Di Giulio A.; individualCount: 1; sex: female; lifeStage: adult; occurrenceID: 2B980BE4-E478-5862-9AFF-71171AB2D1CA; **Taxon:** scientificName: Marinarozelotesadriaticus (Caporiacco, 1951); order: Araneae; family: Gnaphosidae; genus: Marinarozelotes; **Location:** country: Italy; countryCode: IT; stateProvince: Rome; county: Rome; municipality: Rome; locality: Appia Antica Regional Park, Rome; locationRemarks: San Sebastiano; decimalLatitude: 41.855733; decimalLongitude: 12.515114; geodeticDatum: WGS84; **Identification:** identifiedBy: Tommaso Fusco; dateIdentified: 2022; **Event:** samplingProtocol: Pitfall traps; eventDate: 2014-06-19; **Record Level:** collectionID: Roma3_5.8
- **Type status:** Other material. **Occurrence:** recordedBy: Fattorini S., Di Giulio A.; individualCount: 1; sex: male; lifeStage: adult; occurrenceID: B51CE3EB-E5F7-5683-A04F-3B162723EE40; **Taxon:** scientificName: Marinarozelotesadriaticus (Caporiacco, 1951); order: Araneae; family: Gnaphosidae; genus: Marinarozelotes; **Location:** country: Italy; countryCode: IT; stateProvince: Rome; county: Rome; municipality: Rome; locality: Appia Antica Regional Park, Rome; locationRemarks: Tor Marancia; decimalLatitude: 41.850308; decimalLongitude: 12.503178; geodeticDatum: WGS84; **Identification:** identifiedBy: Tommaso Fusco; dateIdentified: 2022; **Event:** samplingProtocol: Pitfall traps; eventDate: 2014-06-17; **Record Level:** collectionID: Roma3_5.8

##### Distribution

From Italy and Balkans to China. Cetralasiatic-Europeo-Mediterranean (CEM) chorotype.

#### Marinarozelotes barbatus

(L. Koch, 1866)

23E843CF-ECB0-598A-A094-F6F797ECF7CF

##### Materials

- **Type status:** Other material. **Occurrence:** recordedBy: Fattorini S., Di Giulio A.; individualCount: 1; sex: male; lifeStage: adult; occurrenceID: 1135BC0A-88A4-5560-B9D8-5F3AE2EF2852; **Taxon:** scientificName: Marinarozelotesbarbatus (L. Koch, 1866); order: Araneae; family: Gnaphosidae; genus: Marinarozelotes; **Location:** country: Italy; countryCode: IT; stateProvince: Rome; county: Rome; municipality: Rome; locality: Appia Antica Regional Park, Rome; locationRemarks: Appia Antica; decimalLatitude: 41.812575; decimalLongitude: 12.564011; geodeticDatum: WGS84; **Identification:** identifiedBy: Tommaso Fusco; dateIdentified: 2022; **Event:** samplingProtocol: Pitfall traps; eventDate: 2014-06-13; **Record Level:** collectionID: Roma3_5.8
- **Type status:** Other material. **Occurrence:** recordedBy: Fattorini S., Di Giulio A.; individualCount: 1; sex: male; lifeStage: adult; occurrenceID: 6B1CED1E-3A1D-5772-A413-23822353E471; **Taxon:** scientificName: Marinarozelotesbarbatus (L. Koch, 1866); order: Araneae; family: Gnaphosidae; genus: Marinarozelotes; **Location:** country: Italy; countryCode: IT; stateProvince: Rome; county: Rome; municipality: Rome; locality: Appia Antica Regional Park, Rome; locationRemarks: Casal Verbeni; decimalLatitude: 41.815250; decimalLongitude: 12.552222; geodeticDatum: WGS84; **Identification:** identifiedBy: Tommaso Fusco; dateIdentified: 2022; **Event:** samplingProtocol: Pitfall traps; eventDate: 2014-05-26; **Record Level:** collectionID: Roma3_5.8
- **Type status:** Other material. **Occurrence:** recordedBy: Fattorini S., Di Giulio A.; individualCount: 2; sex: male; lifeStage: adult; occurrenceID: EB508DB1-3924-555C-843B-0B837E32E2F7; **Taxon:** scientificName: Marinarozelotesbarbatus (L. Koch, 1866); order: Araneae; family: Gnaphosidae; genus: Marinarozelotes; **Location:** country: Italy; countryCode: IT; stateProvince: Rome; county: Rome; municipality: Rome; locality: Appia Antica Regional Park, Rome; locationRemarks: Casal Verbeni; decimalLatitude: 41.815250; decimalLongitude: 12.552222; geodeticDatum: WGS84; **Identification:** identifiedBy: Tommaso Fusco; dateIdentified: 2022; **Event:** samplingProtocol: Pitfall traps; eventDate: 2014-06-05; **Record Level:** collectionID: Roma3_5.8
- **Type status:** Other material. **Occurrence:** recordedBy: Fattorini S., Di Giulio A.; individualCount: 5; sex: 4 male, 1 female; lifeStage: adult; occurrenceID: F13E2D72-2607-5F39-B8EE-619A922B47A5; **Taxon:** scientificName: Marinarozelotesbarbatus (L. Koch, 1866); order: Araneae; family: Gnaphosidae; genus: Marinarozelotes; **Location:** country: Italy; countryCode: IT; stateProvince: Rome; county: Rome; municipality: Rome; locality: Appia Antica Regional Park, Rome; locationRemarks: Casal Verbeni; decimalLatitude: 41.815250; decimalLongitude: 12.552222; geodeticDatum: WGS84; **Identification:** identifiedBy: Tommaso Fusco; dateIdentified: 2022; **Event:** samplingProtocol: Pitfall traps; eventDate: 2014-06-13; **Record Level:** collectionID: Roma3_5.8
- **Type status:** Other material. **Occurrence:** recordedBy: Fattorini S., Di Giulio A.; individualCount: 1; sex: male; lifeStage: adult; occurrenceID: F172E67B-D1BE-5F66-865B-8B399147C406; **Taxon:** scientificName: Marinarozelotesbarbatus (L. Koch, 1866); order: Araneae; family: Gnaphosidae; genus: Marinarozelotes; **Location:** country: Italy; countryCode: IT; stateProvince: Rome; county: Rome; municipality: Rome; locality: Appia Antica Regional Park, Rome; locationRemarks: Cava Fiorucci; decimalLatitude: 41.834106; decimalLongitude: 12.549264; geodeticDatum: WGS84; **Identification:** identifiedBy: Tommaso Fusco; dateIdentified: 2022; **Event:** samplingProtocol: Pitfall traps; eventDate: 2014-06-05; **Record Level:** collectionID: Roma3_5.8
- **Type status:** Other material. **Occurrence:** recordedBy: Fattorini S., Di Giulio A.; individualCount: 3; sex: 2 male, 1 female; lifeStage: adult; occurrenceID: B1F601D5-711E-5F98-BACB-416AB833B830; **Taxon:** scientificName: Marinarozelotesbarbatus (L. Koch, 1866); order: Araneae; family: Gnaphosidae; genus: Marinarozelotes; **Location:** country: Italy; countryCode: IT; stateProvince: Rome; county: Rome; municipality: Rome; locality: Appia Antica Regional Park, Rome; locationRemarks: Cava Fiorucci; decimalLatitude: 41.834106; decimalLongitude: 12.549264; geodeticDatum: WGS84; **Identification:** identifiedBy: Tommaso Fusco; dateIdentified: 2022; **Event:** samplingProtocol: Pitfall traps; eventDate: 2014-06-13; **Record Level:** collectionID: Roma3_5.8
- **Type status:** Other material. **Occurrence:** recordedBy: Fattorini S., Di Giulio A.; individualCount: 5; sex: 4 male, 1 female; lifeStage: adult; occurrenceID: 6372AF5F-AB2D-55C4-ACDA-EF0775361A25; **Taxon:** scientificName: Marinarozelotesbarbatus (L. Koch, 1866); order: Araneae; family: Gnaphosidae; genus: Marinarozelotes; **Location:** country: Italy; countryCode: IT; stateProvince: Rome; county: Rome; municipality: Rome; locality: Appia Antica Regional Park, Rome; locationRemarks: Cava Fiorucci; decimalLatitude: 41.834106; decimalLongitude: 12.549264; geodeticDatum: WGS84; **Identification:** identifiedBy: Tommaso Fusco; dateIdentified: 2022; **Event:** samplingProtocol: Pitfall traps; eventDate: 2014-06-24; **Record Level:** collectionID: Roma3_5.8
- **Type status:** Other material. **Occurrence:** recordedBy: Fattorini S., Di Giulio A.; individualCount: 3; sex: male; lifeStage: adult; occurrenceID: 3A14AD9C-EC44-506F-AB1F-5D22624DBCD7; **Taxon:** scientificName: Marinarozelotesbarbatus (L. Koch, 1866); order: Araneae; family: Gnaphosidae; genus: Marinarozelotes; **Location:** country: Italy; countryCode: IT; stateProvince: Rome; county: Rome; municipality: Rome; locality: Appia Antica Regional Park, Rome; locationRemarks: Farnesiana; decimalLatitude: 41.839667; decimalLongitude: 12.525528; geodeticDatum: WGS84; **Identification:** identifiedBy: Tommaso Fusco; dateIdentified: 2022; **Event:** samplingProtocol: Pitfall traps; eventDate: 2014-06-09; **Record Level:** collectionID: Roma3_5.8
- **Type status:** Other material. **Occurrence:** recordedBy: Fattorini S., Di Giulio A.; individualCount: 3; sex: 2 male, 1 female; lifeStage: adult; occurrenceID: 11EB8A9A-7A44-5817-A5BE-5B918D4544C8; **Taxon:** scientificName: Marinarozelotesbarbatus (L. Koch, 1866); order: Araneae; family: Gnaphosidae; genus: Marinarozelotes; **Location:** country: Italy; countryCode: IT; stateProvince: Rome; county: Rome; municipality: Rome; locality: Appia Antica Regional Park, Rome; locationRemarks: Farnesiana; decimalLatitude: 41.839667; decimalLongitude: 12.525528; geodeticDatum: WGS84; **Identification:** identifiedBy: Tommaso Fusco; dateIdentified: 2022; **Event:** samplingProtocol: Pitfall traps; eventDate: 2014-06-19; **Record Level:** collectionID: Roma3_5.8

##### Distribution

Mediterranean to Caucasus. Introduced to USA. Mediterranean (MED) chorotype.

#### Marinarozelotes huberti

(Platnick & Murphy, 1984)

717255E2-E842-53E9-89AF-068A9202C5BA

##### Materials

- **Type status:** Other material. **Occurrence:** recordedBy: Fattorini S., Di Giulio A.; individualCount: 1; sex: male; lifeStage: adult; occurrenceID: E35C4885-A42C-5E48-9155-7207282ED4D3; **Taxon:** scientificName: Marinarozeloteshuberti (Platnick & Murphy, 1984); order: Araneae; family: Gnaphosidae; genus: Marinarozelotes; **Location:** country: Italy; countryCode: IT; stateProvince: Rome; county: Rome; municipality: Rome; locality: Appia Antica Regional Park, Rome; locationRemarks: Cava Fiorucci; decimalLatitude: 41.834106; decimalLongitude: 12.549264; geodeticDatum: WGS84; **Identification:** identifiedBy: Tommaso Fusco; dateIdentified: 2022; **Event:** samplingProtocol: Pitfall traps; eventDate: 2014-05-26; **Record Level:** collectionID: Roma3_5.8
- **Type status:** Other material. **Occurrence:** recordedBy: Fattorini S., Di Giulio A.; individualCount: 1; sex: male; lifeStage: adult; occurrenceID: B343064F-6823-5579-81AF-8F0CBD5AA598; **Taxon:** scientificName: Marinarozeloteshuberti (Platnick & Murphy, 1984); order: Araneae; family: Gnaphosidae; genus: Marinarozelotes; **Location:** country: Italy; countryCode: IT; stateProvince: Rome; county: Rome; municipality: Rome; locality: Appia Antica Regional Park, Rome; locationRemarks: Torre Selce; decimalLatitude: 41.816611; decimalLongitude: 12.560667; geodeticDatum: WGS84; **Identification:** identifiedBy: Tommaso Fusco; dateIdentified: 2022; **Event:** samplingProtocol: Pitfall traps; eventDate: 2014-06-05; **Record Level:** collectionID: Roma3_5.8
- **Type status:** Other material. **Occurrence:** recordedBy: Fattorini S., Di Giulio A.; individualCount: 1; sex: male; lifeStage: adult; occurrenceID: 382C661F-17E7-57BF-9E2D-986F9018800C; **Taxon:** scientificName: Marinarozeloteshuberti (Platnick & Murphy, 1984); order: Araneae; family: Gnaphosidae; genus: Marinarozelotes; **Location:** country: Italy; countryCode: IT; stateProvince: Rome; county: Rome; municipality: Rome; locality: Appia Antica Regional Park, Rome; locationRemarks: Cava Fiorucci; decimalLatitude: 41.834106; decimalLongitude: 12.549264; geodeticDatum: WGS84; **Identification:** identifiedBy: Tommaso Fusco; dateIdentified: 2022; **Event:** samplingProtocol: Pitfall traps; eventDate: 2014-06-24; **Record Level:** collectionID: Roma3_5.8
- **Type status:** Other material. **Occurrence:** recordedBy: Fattorini S., Di Giulio A.; individualCount: 1; sex: male; lifeStage: adult; occurrenceID: 2A2F7BB2-BE98-52C9-AF9D-8A7B24B813FE; **Taxon:** scientificName: Marinarozeloteshuberti (Platnick & Murphy, 1984); order: Araneae; family: Gnaphosidae; genus: Marinarozelotes; **Location:** country: Italy; countryCode: IT; stateProvince: Rome; county: Rome; municipality: Rome; locality: Appia Antica Regional Park, Rome; locationRemarks: Tor Marancia; decimalLatitude: 41.850308; decimalLongitude: 12.503178; geodeticDatum: WGS84; **Identification:** identifiedBy: Tommaso Fusco; dateIdentified: 2022; **Event:** samplingProtocol: Pitfall traps; eventDate: 2014-06-17; **Record Level:** collectionID: Roma3_5.8
- **Type status:** Other material. **Occurrence:** recordedBy: Fattorini S., Di Giulio A.; individualCount: 1; sex: male; lifeStage: adult; occurrenceID: A264B4E1-A1D6-513E-9BC7-3142CA18424C; **Taxon:** scientificName: Marinarozeloteshuberti (Platnick & Murphy, 1984); order: Araneae; family: Gnaphosidae; genus: Marinarozelotes; **Location:** country: Italy; countryCode: IT; stateProvince: Rome; county: Rome; municipality: Rome; locality: Appia Antica Regional Park, Rome; locationRemarks: Appia Antica; decimalLatitude: 41.812575; decimalLongitude: 12.564011; geodeticDatum: WGS84; **Identification:** identifiedBy: Tommaso Fusco; dateIdentified: 2022; **Event:** samplingProtocol: Pitfall traps; eventDate: 2014-06-13; **Record Level:** collectionID: Roma3_5.8

##### Distribution

Algeria, Italy, Albania. Mediterranean (MED) chorotype.

#### Micaria micans

(Blackwall, 1858)

52F0D6E3-DEC7-5AFC-BC28-5F77EFD518AC

##### Materials

- **Type status:** Other material. **Occurrence:** recordedBy: Fattorini S., Di Giulio A.; individualCount: 1; sex: female; lifeStage: adult; occurrenceID: 4541DF0E-8466-5892-98C1-FC8BC2D8AFF6; **Taxon:** scientificName: Micariamicans (Blackwall, 1858); order: Araneae; family: Gnaphosidae; genus: Micaria; **Location:** country: Italy; countryCode: IT; stateProvince: Rome; county: Rome; municipality: Rome; locality: Appia Antica Regional Park, Rome; locationRemarks: Tor Marancia; decimalLatitude: 41.850308; decimalLongitude: 12.503178; geodeticDatum: WGS84; **Identification:** identifiedBy: Tommaso Fusco; dateIdentified: 2022; **Event:** samplingProtocol: Pitfall traps; eventDate: 2014-05-26; **Record Level:** collectionID: Roma3_5.8

##### Distribution

Europe, Caucasus, Russia (Europe to South Siberia), Central Asia. Asiatic-European (ASE) chorotype.

#### Micaria pallipes

(Lucas, 1846)

E93414FE-D403-55D1-9293-4319B3B6D064

##### Materials

- **Type status:** Other material. **Occurrence:** recordedBy: Fattorini S., Di Giulio A.; individualCount: 1; sex: female; lifeStage: adult; occurrenceID: 18400A8C-E5BD-5076-95BC-E8EC2BFDD784; **Taxon:** scientificName: Micariapallipes (Lucas, 1846); order: Araneae; family: Gnaphosidae; genus: Micaria; **Location:** country: Italy; countryCode: IT; stateProvince: Rome; county: Rome; municipality: Rome; locality: Appia Antica Regional Park, Rome; locationRemarks: Acqua Santa; decimalLatitude: 41.850308; decimalLongitude: 12.503178; geodeticDatum: WGS84; **Identification:** identifiedBy: Tommaso Fusco; dateIdentified: 2022; **Event:** samplingProtocol: Pitfall traps; eventDate: 2014-06-19; **Record Level:** collectionID: Roma3_5.8

##### Distribution

Mediterranean, Russia (Europe), Caucasus, Kazakhstan, Iran, Turkmenistan. Turano-Mediterranean (TUM) chorotype.

#### Nomisia exornata

(C. L. Koch, 1839)

F88E8C8C-F62E-5A83-BD1A-FCEF582B2FFA

##### Materials

- **Type status:** Other material. **Occurrence:** recordedBy: Fattorini S., Di Giulio A.; individualCount: 2; sex: male; lifeStage: adult; occurrenceID: D6BF8900-3CA6-5881-A5DB-EC4386643AD0; **Taxon:** scientificName: Nomisiaexornata (C. L. Koch, 1839); order: Araneae; family: Gnaphosidae; genus: Nomisia; **Location:** country: Italy; countryCode: IT; stateProvince: Rome; county: Rome; municipality: Rome; locality: Appia Antica Regional Park, Rome; locationRemarks: Appia Antica; decimalLatitude: 41.812575; decimalLongitude: 12.564011; geodeticDatum: WGS84; **Identification:** identifiedBy: Tommaso Fusco; dateIdentified: 2022; **Event:** samplingProtocol: Pitfall traps; eventDate: 2014-06-13; **Record Level:** collectionID: Roma3_5.8
- **Type status:** Other material. **Occurrence:** recordedBy: Fattorini S., Di Giulio A.; individualCount: 1; sex: male; lifeStage: adult; occurrenceID: E48457F6-DF76-59AB-90B1-F46C7C9BF9C3; **Taxon:** scientificName: Nomisiaexornata (C. L. Koch, 1839); order: Araneae; family: Gnaphosidae; genus: Nomisia; **Location:** country: Italy; countryCode: IT; stateProvince: Rome; county: Rome; municipality: Rome; locality: Appia Antica Regional Park, Rome; locationRemarks: Caffarella Sud 3; decimalLatitude: 41.856928; decimalLongitude: 12.528406; geodeticDatum: WGS84; **Identification:** identifiedBy: Tommaso Fusco; dateIdentified: 2022; **Event:** samplingProtocol: Pitfall traps; eventDate: 2014-06-06; **Record Level:** collectionID: Roma3_5.8
- **Type status:** Other material. **Occurrence:** recordedBy: Fattorini S., Di Giulio A.; individualCount: 1; sex: male; lifeStage: adult; occurrenceID: 8E14D815-9B01-5650-890B-8A43CC9E58FA; **Taxon:** scientificName: Nomisiaexornata (C. L. Koch, 1839); order: Araneae; family: Gnaphosidae; genus: Nomisia; **Location:** country: Italy; countryCode: IT; stateProvince: Rome; county: Rome; municipality: Rome; locality: Appia Antica Regional Park, Rome; locationRemarks: Cava Fiorucci; decimalLatitude: 41.834106; decimalLongitude: 12.549264; geodeticDatum: WGS84; **Identification:** identifiedBy: Tommaso Fusco; dateIdentified: 2022; **Event:** samplingProtocol: Pitfall traps; eventDate: 2014-05-26; **Record Level:** collectionID: Roma3_5.8
- **Type status:** Other material. **Occurrence:** recordedBy: Fattorini S., Di Giulio A.; individualCount: 1; sex: male; lifeStage: adult; occurrenceID: 7896B1FD-4FE3-51CD-ADDB-628FF91CE3F5; **Taxon:** scientificName: Nomisiaexornata (C. L. Koch, 1839); order: Araneae; family: Gnaphosidae; genus: Nomisia; **Location:** country: Italy; countryCode: IT; stateProvince: Rome; county: Rome; municipality: Rome; locality: Appia Antica Regional Park, Rome; locationRemarks: Cava Fiorucci; decimalLatitude: 41.834106; decimalLongitude: 12.549264; geodeticDatum: WGS84; **Identification:** identifiedBy: Tommaso Fusco; dateIdentified: 2022; **Event:** samplingProtocol: Pitfall traps; eventDate: 2014-06-13; **Record Level:** collectionID: Roma3_5.8
- **Type status:** Other material. **Occurrence:** recordedBy: Fattorini S., Di Giulio A.; individualCount: 1; sex: male; lifeStage: adult; occurrenceID: 7FDA6513-17D2-5393-8E06-01B120D91141; **Taxon:** scientificName: Nomisiaexornata (C. L. Koch, 1839); order: Araneae; family: Gnaphosidae; genus: Nomisia; **Location:** country: Italy; countryCode: IT; stateProvince: Rome; county: Rome; municipality: Rome; locality: Appia Antica Regional Park, Rome; locationRemarks: Farnesiana; decimalLatitude: 41.839667; decimalLongitude: 12.525528; geodeticDatum: WGS84; **Identification:** identifiedBy: Tommaso Fusco; dateIdentified: 2022; **Event:** samplingProtocol: Pitfall traps; eventDate: 2014-05-20; **Record Level:** collectionID: Roma3_5.8
- **Type status:** Other material. **Occurrence:** recordedBy: Fattorini S., Di Giulio A.; individualCount: 2; sex: male; lifeStage: adult; occurrenceID: 57F4C3EF-6F97-5954-9838-6B0B115B87A1; **Taxon:** scientificName: Nomisiaexornata (C. L. Koch, 1839); order: Araneae; family: Gnaphosidae; genus: Nomisia; **Location:** country: Italy; countryCode: IT; stateProvince: Rome; county: Rome; municipality: Rome; locality: Appia Antica Regional Park, Rome; locationRemarks: Farnesiana; decimalLatitude: 41.839667; decimalLongitude: 12.525528; geodeticDatum: WGS84; **Identification:** identifiedBy: Tommaso Fusco; dateIdentified: 2022; **Event:** samplingProtocol: Pitfall traps; eventDate: 2014-06-09; **Record Level:** collectionID: Roma3_5.8
- **Type status:** Other material. **Occurrence:** recordedBy: Fattorini S., Di Giulio A.; individualCount: 1; sex: female; lifeStage: adult; occurrenceID: 84F130EB-8D67-5B02-BC28-F6663D1D1E3F; **Taxon:** scientificName: Nomisiaexornata (C. L. Koch, 1839); order: Araneae; family: Gnaphosidae; genus: Nomisia; **Location:** country: Italy; countryCode: IT; stateProvince: Rome; county: Rome; municipality: Rome; locality: Appia Antica Regional Park, Rome; locationRemarks: Farnesiana; decimalLatitude: 41.839667; decimalLongitude: 12.525528; geodeticDatum: WGS84; **Identification:** identifiedBy: Tommaso Fusco; dateIdentified: 2022; **Event:** samplingProtocol: Pitfall traps; eventDate: 2014-06-19; **Record Level:** collectionID: Roma3_5.8
- **Type status:** Other material. **Occurrence:** recordedBy: Fattorini S., Di Giulio A.; individualCount: 1; sex: male; lifeStage: adult; occurrenceID: 10900E01-E0A3-578B-B7AA-1A25FE9C3126; **Taxon:** scientificName: Nomisiaexornata (C. L. Koch, 1839); order: Araneae; family: Gnaphosidae; genus: Nomisia; **Location:** country: Italy; countryCode: IT; stateProvince: Rome; county: Rome; municipality: Rome; locality: Appia Antica Regional Park, Rome; locationRemarks: San Sebastiano; decimalLatitude: 41.855733; decimalLongitude: 12.515114; geodeticDatum: WGS84; **Identification:** identifiedBy: Tommaso Fusco; dateIdentified: 2022; **Event:** samplingProtocol: Pitfall traps; eventDate: 2014-05-28; **Record Level:** collectionID: Roma3_5.8

##### Distribution

Europe, North Africa, Turkey, Caucasus, Central Asia. Turano-Europeo-Mediterranean (TEM) chorotype.

#### Phaeocedus braccatus

(L. Koch, 1866)

48C8A567-E119-5B9C-A3D7-D82A681DFF95

##### Materials

- **Type status:** Other material. **Occurrence:** recordedBy: Fattorini S., Di Giulio A.; individualCount: 1; sex: male; lifeStage: adult; occurrenceID: AAA0A64F-BB62-56EB-AC3D-DD36DB05C677; **Taxon:** scientificName: Phaeocedusbraccatus (L. Koch, 1866); order: Araneae; family: Gnaphosidae; genus: Phaeocedus; **Location:** country: Italy; countryCode: IT; stateProvince: Rome; county: Rome; municipality: Rome; locality: Appia Antica Regional Park, Rome; locationRemarks: Farnesiana; decimalLatitude: 41.839667; decimalLongitude: 12.525528; geodeticDatum: WGS84; **Identification:** identifiedBy: Tommaso Fusco; dateIdentified: 2022; **Event:** samplingProtocol: Pitfall traps; eventDate: 2014-06-19; **Record Level:** collectionID: Roma3_5.8

##### Distribution

Morocco, Europe, Turkey, Caucasus, Russia (Europe to Far East) to China, Japan. Palaearctic (PAL) chorotype.

#### Setaphis carmeli

(O. Pickard-Cambridge, 1872)

166343B8-C8B7-599C-81B4-0CE7C8E2EA37

##### Materials

- **Type status:** Other material. **Occurrence:** recordedBy: Fattorini S., Di Giulio A.; individualCount: 1; sex: male; lifeStage: adult; occurrenceID: C6E46CCA-171F-5764-B8CC-21E6DF4FB3D9; **Taxon:** scientificName: Setaphiscarmeli (O. Pickard-Cambridge, 1872); order: Araneae; family: Gnaphosidae; genus: Setaphis; **Location:** country: Italy; countryCode: IT; stateProvince: Rome; county: Rome; municipality: Rome; locality: Appia Antica Regional Park, Rome; locationRemarks: Acqua Santa; decimalLatitude: 41.850561; decimalLongitude: 12.530861; geodeticDatum: WGS84; **Identification:** identifiedBy: Tommaso Fusco; dateIdentified: 2022; **Event:** samplingProtocol: Pitfall traps; eventDate: 2014-06-09; **Record Level:** collectionID: Roma3_5.8
- **Type status:** Other material. **Occurrence:** recordedBy: Fattorini S., Di Giulio A.; individualCount: 1; sex: male; lifeStage: adult; occurrenceID: C3EF4810-9F55-5362-A1DD-765FFA76800C; **Taxon:** scientificName: Setaphiscarmeli (O. Pickard-Cambridge, 1872); order: Araneae; family: Gnaphosidae; genus: Setaphis; **Location:** country: Italy; countryCode: IT; stateProvince: Rome; county: Rome; municipality: Rome; locality: Appia Antica Regional Park, Rome; locationRemarks: Appia Antica; decimalLatitude: 41.812575; decimalLongitude: 12.564011; geodeticDatum: WGS84; **Identification:** identifiedBy: Tommaso Fusco; dateIdentified: 2022; **Event:** samplingProtocol: Pitfall traps; eventDate: 2014-06-05; **Record Level:** collectionID: Roma3_5.8
- **Type status:** Other material. **Occurrence:** recordedBy: Fattorini S., Di Giulio A.; individualCount: 3; sex: male; lifeStage: adult; occurrenceID: 80CFEAF3-4C9B-5C02-AEBA-6AAF37E5DF09; **Taxon:** scientificName: Setaphiscarmeli (O. Pickard-Cambridge, 1872); order: Araneae; family: Gnaphosidae; genus: Setaphis; **Location:** country: Italy; countryCode: IT; stateProvince: Rome; county: Rome; municipality: Rome; locality: Appia Antica Regional Park, Rome; locationRemarks: Caffarella Sud; decimalLatitude: 41.857247; decimalLongitude: 12.529211; geodeticDatum: WGS84; **Identification:** identifiedBy: Tommaso Fusco; dateIdentified: 2022; **Event:** samplingProtocol: Pitfall traps; eventDate: 2014-06-06; **Record Level:** collectionID: Roma3_5.8
- **Type status:** Other material. **Occurrence:** recordedBy: Fattorini S., Di Giulio A.; individualCount: 1; sex: male; lifeStage: adult; occurrenceID: F3C945EF-51EB-5B1C-AE56-5E9FD36FE6E2; **Taxon:** scientificName: Setaphiscarmeli (O. Pickard-Cambridge, 1872); order: Araneae; family: Gnaphosidae; genus: Setaphis; **Location:** country: Italy; countryCode: IT; stateProvince: Rome; county: Rome; municipality: Rome; locality: Appia Antica Regional Park, Rome; locationRemarks: Casal Verbeni; decimalLatitude: 41.815250; decimalLongitude: 12.552222; geodeticDatum: WGS84; **Identification:** identifiedBy: Tommaso Fusco; dateIdentified: 2022; **Event:** samplingProtocol: Pitfall traps; eventDate: 2014-05-26; **Record Level:** collectionID: Roma3_5.8
- **Type status:** Other material. **Occurrence:** recordedBy: Fattorini S., Di Giulio A.; individualCount: 1; sex: male; lifeStage: adult; occurrenceID: 6838A2A9-08A2-5EF8-86B5-CC453F211BD2; **Taxon:** scientificName: Setaphiscarmeli (O. Pickard-Cambridge, 1872); order: Araneae; family: Gnaphosidae; genus: Setaphis; **Location:** country: Italy; countryCode: IT; stateProvince: Rome; county: Rome; municipality: Rome; locality: Appia Antica Regional Park, Rome; locationRemarks: Casal Verbeni; decimalLatitude: 41.815250; decimalLongitude: 12.552222; geodeticDatum: WGS84; **Identification:** identifiedBy: Tommaso Fusco; dateIdentified: 2022; **Event:** samplingProtocol: Pitfall traps; eventDate: 2014-06-13; **Record Level:** collectionID: Roma3_5.8
- **Type status:** Other material. **Occurrence:** recordedBy: Fattorini S., Di Giulio A.; individualCount: 1; sex: male; lifeStage: adult; occurrenceID: 05DD60D8-8AB8-572A-B2AA-926DBABA67FC; **Taxon:** scientificName: Setaphiscarmeli (O. Pickard-Cambridge, 1872); order: Araneae; family: Gnaphosidae; genus: Setaphis; **Location:** country: Italy; countryCode: IT; stateProvince: Rome; county: Rome; municipality: Rome; locality: Appia Antica Regional Park, Rome; locationRemarks: Farnesiana; decimalLatitude: 41.839667; decimalLongitude: 12.525528; geodeticDatum: WGS84; **Identification:** identifiedBy: Tommaso Fusco; dateIdentified: 2022; **Event:** samplingProtocol: Pitfall traps; eventDate: 2014-05-26; **Record Level:** collectionID: Roma3_5.8
- **Type status:** Other material. **Occurrence:** recordedBy: Fattorini S., Di Giulio A.; individualCount: 2; sex: male; lifeStage: adult; occurrenceID: C68311A1-A774-5D0A-A84E-0BE0F9CBAD9F; **Taxon:** scientificName: Setaphiscarmeli (O. Pickard-Cambridge, 1872); order: Araneae; family: Gnaphosidae; genus: Setaphis; **Location:** country: Italy; countryCode: IT; stateProvince: Rome; county: Rome; municipality: Rome; locality: Appia Antica Regional Park, Rome; locationRemarks: Farnesiana; decimalLatitude: 41.839667; decimalLongitude: 12.525528; geodeticDatum: WGS84; **Identification:** identifiedBy: Tommaso Fusco; dateIdentified: 2022; **Event:** samplingProtocol: Pitfall traps; eventDate: 2014-05-28; **Record Level:** collectionID: Roma3_5.8
- **Type status:** Other material. **Occurrence:** recordedBy: Fattorini S., Di Giulio A.; individualCount: 1; sex: female; lifeStage: adult; occurrenceID: D49101C2-6406-5DA8-9800-8CC68EC1C57D; **Taxon:** scientificName: Setaphiscarmeli (O. Pickard-Cambridge, 1872); order: Araneae; family: Gnaphosidae; genus: Setaphis; **Location:** country: Italy; countryCode: IT; stateProvince: Rome; county: Rome; municipality: Rome; locality: Appia Antica Regional Park, Rome; locationRemarks: Farnesiana; decimalLatitude: 41.839667; decimalLongitude: 12.525528; geodeticDatum: WGS84; **Identification:** identifiedBy: Tommaso Fusco; dateIdentified: 2022; **Event:** samplingProtocol: Pitfall traps; eventDate: 2013-11-25; **Record Level:** collectionID: Roma3_5.8
- **Type status:** Other material. **Occurrence:** recordedBy: Fattorini S., Di Giulio A.; individualCount: 3; sex: male; lifeStage: adult; occurrenceID: D5889FD5-04C1-5EB4-BA27-ABCEEDFAD33B; **Taxon:** scientificName: Setaphiscarmeli (O. Pickard-Cambridge, 1872); order: Araneae; family: Gnaphosidae; genus: Setaphis; **Location:** country: Italy; countryCode: IT; stateProvince: Rome; county: Rome; municipality: Rome; locality: Appia Antica Regional Park, Rome; locationRemarks: Farnesiana; decimalLatitude: 41.839667; decimalLongitude: 12.525528; geodeticDatum: WGS84; **Identification:** identifiedBy: Tommaso Fusco; dateIdentified: 2022; **Event:** samplingProtocol: Pitfall traps; eventDate: 2014-06-09; **Record Level:** collectionID: Roma3_5.8
- **Type status:** Other material. **Occurrence:** recordedBy: Fattorini S., Di Giulio A.; individualCount: 5; sex: 1 female, 4 male; lifeStage: adult; occurrenceID: BA73F19A-5506-5DB4-A610-01F317572C87; **Taxon:** scientificName: Setaphiscarmeli (O. Pickard-Cambridge, 1872); order: Araneae; family: Gnaphosidae; genus: Setaphis; **Location:** country: Italy; countryCode: IT; stateProvince: Rome; county: Rome; municipality: Rome; locality: Appia Antica Regional Park, Rome; locationRemarks: Farnesiana; decimalLatitude: 41.839667; decimalLongitude: 12.525528; geodeticDatum: WGS84; **Identification:** identifiedBy: Tommaso Fusco; dateIdentified: 2022; **Event:** samplingProtocol: Pitfall traps; eventDate: 2014-06-19; **Record Level:** collectionID: Roma3_5.8
- **Type status:** Other material. **Occurrence:** recordedBy: Fattorini S., Di Giulio A.; individualCount: 2; sex: male; lifeStage: adult; occurrenceID: 43CF6902-AF7F-54F0-A35C-9C6AC34B5843; **Taxon:** scientificName: Setaphiscarmeli (O. Pickard-Cambridge, 1872); order: Araneae; family: Gnaphosidae; genus: Setaphis; **Location:** country: Italy; countryCode: IT; stateProvince: Rome; county: Rome; municipality: Rome; locality: Appia Antica Regional Park, Rome; locationRemarks: Tor Marancia; decimalLatitude: 41.850308; decimalLongitude: 12.503178; geodeticDatum: WGS84; **Identification:** identifiedBy: Tommaso Fusco; dateIdentified: 2022; **Event:** samplingProtocol: Pitfall traps; eventDate: 2014-05-27; **Record Level:** collectionID: Roma3_5.8
- **Type status:** Other material. **Occurrence:** recordedBy: Fattorini S., Di Giulio A.; individualCount: 2; sex: male; lifeStage: adult; occurrenceID: FF592B62-6B3A-57FC-8C2E-A4518015DD1F; **Taxon:** scientificName: Setaphiscarmeli (O. Pickard-Cambridge, 1872); order: Araneae; family: Gnaphosidae; genus: Setaphis; **Location:** country: Italy; countryCode: IT; stateProvince: Rome; county: Rome; municipality: Rome; locality: Appia Antica Regional Park, Rome; locationRemarks: Tor Marancia; decimalLatitude: 41.850308; decimalLongitude: 12.503178; geodeticDatum: WGS84; **Identification:** identifiedBy: Tommaso Fusco; dateIdentified: 2022; **Event:** samplingProtocol: Pitfall traps; eventDate: 2014-06-05; **Record Level:** collectionID: Roma3_5.8
- **Type status:** Other material. **Occurrence:** recordedBy: Fattorini S., Di Giulio A.; individualCount: 1; sex: female; lifeStage: adult; occurrenceID: 4E1DEF03-F48F-5A06-9C63-6A762F7B4AC5; **Taxon:** scientificName: Setaphiscarmeli (O. Pickard-Cambridge, 1872); order: Araneae; family: Gnaphosidae; genus: Setaphis; **Location:** country: Italy; countryCode: IT; stateProvince: Rome; county: Rome; municipality: Rome; locality: Appia Antica Regional Park, Rome; locationRemarks: Torre Selce; decimalLatitude: 41.816611; decimalLongitude: 12.560667; geodeticDatum: WGS84; **Identification:** identifiedBy: Tommaso Fusco; dateIdentified: 2022; **Event:** samplingProtocol: Pitfall traps; eventDate: 2014-06-05; **Record Level:** collectionID: Roma3_5.8

##### Distribution

Widespread throughout the Mediterranean area. Mediterranean (MED) chorotype.

#### Trachyzelotes pedestris

(C. L. Koch, 1837)

D9D7A9D3-772D-56AA-831C-0287E06A400A

##### Materials

- **Type status:** Other material. **Occurrence:** recordedBy: Fattorini S., Di Giulio A.; individualCount: 1; sex: female; lifeStage: adult; occurrenceID: 397E3EAA-43E6-5BE9-90BE-178BC8415FCD; **Taxon:** scientificName: Trachyzelotespedestris (C. L. Koch, 1837); order: Araneae; family: Gnaphosidae; genus: Trachyzelotes; **Location:** country: Italy; countryCode: IT; stateProvince: Rome; county: Rome; municipality: Rome; locality: Appia Antica Regional Park, Rome; locationRemarks: Tor Marancia; decimalLatitude: 41.850308; decimalLongitude: 12.503178; geodeticDatum: WGS84; **Identification:** identifiedBy: Tommaso Fusco; dateIdentified: 2022; **Event:** samplingProtocol: Pitfall traps; eventDate: 2014-05-26; **Record Level:** collectionID: Roma3_5.8
- **Type status:** Other material. **Occurrence:** recordedBy: Fattorini S., Di Giulio A.; individualCount: 1; sex: male; lifeStage: adult; occurrenceID: AE9856B6-B6E6-5871-B068-6DF03CD84A38; **Taxon:** scientificName: Trachyzelotespedestris (C. L. Koch, 1837); order: Araneae; family: Gnaphosidae; genus: Trachyzelotes; **Location:** country: Italy; countryCode: IT; stateProvince: Rome; county: Rome; municipality: Rome; locality: Appia Antica Regional Park, Rome; locationRemarks: Tor Marancia; decimalLatitude: 41.850308; decimalLongitude: 12.503178; geodeticDatum: WGS84; **Identification:** identifiedBy: Tommaso Fusco; dateIdentified: 2022; **Event:** samplingProtocol: Pitfall traps; eventDate: 2014-06-05; **Record Level:** collectionID: Roma3_5.8
- **Type status:** Other material. **Occurrence:** recordedBy: Fattorini S., Di Giulio A.; individualCount: 1; sex: male; lifeStage: adult; occurrenceID: 10D52DC8-767D-52B6-A39D-F7B5B20DF1A9; **Taxon:** scientificName: Trachyzelotespedestris (C. L. Koch, 1837); order: Araneae; family: Gnaphosidae; genus: Trachyzelotes; **Location:** country: Italy; countryCode: IT; stateProvince: Rome; county: Rome; municipality: Rome; locality: Appia Antica Regional Park, Rome; locationRemarks: Caffarella Nord; decimalLatitude: 41.867753; decimalLongitude: 12.512414; geodeticDatum: WGS84; **Identification:** identifiedBy: Tommaso Fusco; dateIdentified: 2022; **Event:** samplingProtocol: Pitfall traps; eventDate: 2014-05-19; **Record Level:** collectionID: Roma3_5.8
- **Type status:** Other material. **Occurrence:** recordedBy: Fattorini S., Di Giulio A.; individualCount: 1; sex: female; lifeStage: adult; occurrenceID: 063BF45F-546D-51E3-BCF1-5AC241FECF20; **Taxon:** scientificName: Trachyzelotespedestris (C. L. Koch, 1837); order: Araneae; family: Gnaphosidae; genus: Trachyzelotes; **Location:** country: Italy; countryCode: IT; stateProvince: Rome; county: Rome; municipality: Rome; locality: Appia Antica Regional Park, Rome; locationRemarks: Caffarella Centro; decimalLatitude: 41.864889; decimalLongitude: 12.516389; geodeticDatum: WGS84; **Identification:** identifiedBy: Tommaso Fusco; dateIdentified: 2022; **Event:** samplingProtocol: Pitfall traps; eventDate: 2014-06-18; **Record Level:** collectionID: Roma3_5.8

##### Distribution

Europe, Caucasus, Turkey, Iran. Turano-European (TUE) chorotype.

#### Turkozelotes noname

Mazzia & Cornic, 2020

1797A8C2-7150-55A1-9F5E-E19911951AEE

##### Materials

- **Type status:** Other material. **Occurrence:** recordedBy: Fattorini S., Di Giulio A.; individualCount: 1; sex: female; lifeStage: adult; occurrenceID: 88F79B3E-C600-5768-8D05-506FEE26637D; **Taxon:** scientificName: Turkozelotesnoname Mazzia & Cornic, 2020; order: Araneae; family: Gnaphosidae; genus: Turkozelotes; **Location:** country: Italy; countryCode: IT; stateProvince: Rome; county: Rome; municipality: Rome; locality: Appia Antica Regional Park, Rome; locationRemarks: Farnesiana; decimalLatitude: 41.839667; decimalLongitude: 12.525528; geodeticDatum: WGS84; **Identification:** identifiedBy: Tommaso Fusco; dateIdentified: 2022; **Event:** samplingProtocol: Pitfall traps; eventDate: 2014-06-19; **Record Level:** collectionID: Roma3_5.8

##### Distribution

Italy and France. W-Mediterranean (WME) chorotype.

##### Notes

Habitus in Fig. 6.

#### Urozelotes rusticus

(L. Koch, 1872)

3657EC8B-D6C2-532A-9917-D0D77EDDF5AF

##### Materials

- **Type status:** Other material. **Occurrence:** recordedBy: Fattorini S., Di Giulio A.; individualCount: 1; sex: male; lifeStage: adult; occurrenceID: D0731A64-ED0B-51C3-9184-B67182341570; **Taxon:** scientificName: Urozelotesrusticus (L. Koch, 1872); order: Araneae; family: Gnaphosidae; genus: Urozelotes; **Location:** country: Italy; countryCode: IT; stateProvince: Rome; county: Rome; municipality: Rome; locality: Appia Antica Regional Park, Rome; locationRemarks: Casal Verbeni; decimalLatitude: 41.815250; decimalLongitude: 12.552222; geodeticDatum: WGS84; **Identification:** identifiedBy: Tommaso Fusco; dateIdentified: 2022; **Event:** samplingProtocol: Pitfall traps; eventDate: 2014-05-26; **Record Level:** collectionID: Roma3_5.8
- **Type status:** Other material. **Occurrence:** recordedBy: Fattorini S., Di Giulio A.; individualCount: 1; sex: male; lifeStage: adult; occurrenceID: DBB2B545-94B8-50F9-BFD3-EC65C4D2E4AC; **Taxon:** scientificName: Urozelotesrusticus (L. Koch, 1872); order: Araneae; family: Gnaphosidae; genus: Urozelotes; **Location:** country: Italy; countryCode: IT; stateProvince: Rome; county: Rome; municipality: Rome; locality: Appia Antica Regional Park, Rome; locationRemarks: Casal Verbeni; decimalLatitude: 41.815250; decimalLongitude: 12.552222; geodeticDatum: WGS84; **Identification:** identifiedBy: Tommaso Fusco; dateIdentified: 2022; **Event:** samplingProtocol: Pitfall traps; eventDate: 2014-06-13; **Record Level:** collectionID: Roma3_5.8

##### Distribution

Native to Europe/Mediterranean to temperate Asia. Introduced to North and South America, tropical Africa, Australia. Cosmopolitan (COS) chorotype.

#### Zelotes atrocaeruleus

(Simon, 1878)

AB84E98C-EF5C-56FC-8BFC-39E6AE9FD9A0

##### Materials

- **Type status:** Other material. **Occurrence:** recordedBy: Fattorini S., Di Giulio A.; individualCount: 2; sex: male; lifeStage: adult; occurrenceID: C9C0C54F-5B04-5D06-9DAE-1B6267E33CD1; **Taxon:** scientificName: Zelotesatrocaeruleus (Simon, 1878); order: Araneae; family: Gnaphosidae; genus: Zelotes; **Location:** country: Italy; countryCode: IT; stateProvince: Rome; county: Rome; municipality: Rome; locality: Appia Antica Regional Park, Rome; locationRemarks: Cava Fiorucci; decimalLatitude: 41.834106; decimalLongitude: 12.549264; geodeticDatum: WGS84; **Identification:** identifiedBy: Tommaso Fusco; dateIdentified: 2022; **Event:** samplingProtocol: Pitfall traps; eventDate: 2014-06-05; **Record Level:** collectionID: Roma3_5.8
- **Type status:** Other material. **Occurrence:** recordedBy: Fattorini S., Di Giulio A.; individualCount: 1; sex: female; lifeStage: adult; occurrenceID: 81654F56-E36F-558E-BE49-AA376ABC364B; **Taxon:** scientificName: Zelotesatrocaeruleus (Simon, 1878); order: Araneae; family: Gnaphosidae; genus: Zelotes; **Location:** country: Italy; countryCode: IT; stateProvince: Rome; county: Rome; municipality: Rome; locality: Appia Antica Regional Park, Rome; locationRemarks: Cava Fiorucci; decimalLatitude: 41.834106; decimalLongitude: 12.549264; geodeticDatum: WGS84; **Identification:** identifiedBy: Tommaso Fusco; dateIdentified: 2022; **Event:** samplingProtocol: Pitfall traps; eventDate: 2014-06-24; **Record Level:** collectionID: Roma3_5.8
- **Type status:** Other material. **Occurrence:** recordedBy: Fattorini S., Di Giulio A.; individualCount: 4; sex: 3 male, 1 female; lifeStage: adult; occurrenceID: 5AE2C26D-040F-5E7D-A520-E9BA34FFCE25; **Taxon:** scientificName: Zelotesatrocaeruleus (Simon, 1878); order: Araneae; family: Gnaphosidae; genus: Zelotes; **Location:** country: Italy; countryCode: IT; stateProvince: Rome; county: Rome; municipality: Rome; locality: Appia Antica Regional Park, Rome; locationRemarks: Farnesiana; decimalLatitude: 41.839667; decimalLongitude: 12.525528; geodeticDatum: WGS84; **Identification:** identifiedBy: Tommaso Fusco; dateIdentified: 2022; **Event:** samplingProtocol: Pitfall traps; eventDate: 2014-05-28; **Record Level:** collectionID: Roma3_5.8
- **Type status:** Other material. **Occurrence:** recordedBy: Fattorini S., Di Giulio A.; individualCount: 8; sex: 7 male, 1 female; lifeStage: adult; occurrenceID: 62256B62-E2EA-5B7D-A413-F358A4A9B08F; **Taxon:** scientificName: Zelotesatrocaeruleus (Simon, 1878); order: Araneae; family: Gnaphosidae; genus: Zelotes; **Location:** country: Italy; countryCode: IT; stateProvince: Rome; county: Rome; municipality: Rome; locality: Appia Antica Regional Park, Rome; locationRemarks: Farnesiana; decimalLatitude: 41.839667; decimalLongitude: 12.525528; geodeticDatum: WGS84; **Identification:** identifiedBy: Tommaso Fusco; dateIdentified: 2022; **Event:** samplingProtocol: Pitfall traps; eventDate: 2014-06-09; **Record Level:** collectionID: Roma3_5.8
- **Type status:** Other material. **Occurrence:** recordedBy: Fattorini S., Di Giulio A.; individualCount: 11; sex: 7 male, 4 female; lifeStage: adult; occurrenceID: 752767F1-AA54-5F52-882D-0AD1455DE57C; **Taxon:** scientificName: Zelotesatrocaeruleus (Simon, 1878); order: Araneae; family: Gnaphosidae; genus: Zelotes; **Location:** country: Italy; countryCode: IT; stateProvince: Rome; county: Rome; municipality: Rome; locality: Appia Antica Regional Park, Rome; locationRemarks: Farnesiana; decimalLatitude: 41.839667; decimalLongitude: 12.525528; geodeticDatum: WGS84; **Identification:** identifiedBy: Tommaso Fusco; dateIdentified: 2022; **Event:** samplingProtocol: Pitfall traps; eventDate: 2014-06-19; **Record Level:** collectionID: Roma3_5.8

##### Distribution

Europe, Turkey, Caucasus, from Russia (Europe) to China. Centralasiatic-European (CAE) chorotype.

#### Zelotes femellus

(L. Koch, 1866)

56119316-59D3-5F56-8C4F-F6591E2B0797

##### Materials

- **Type status:** Other material. **Occurrence:** recordedBy: Fattorini S., Di Giulio A.; individualCount: 1; sex: male; lifeStage: adult; occurrenceID: 836D867C-A914-51FA-80F5-AD44A91B5E8B; **Taxon:** scientificName: Zelotesfemellus (L. Koch, 1866); order: Araneae; family: Gnaphosidae; genus: Zelotes; **Location:** country: Italy; countryCode: IT; stateProvince: Rome; county: Rome; municipality: Rome; locality: Appia Antica Regional Park, Rome; locationRemarks: Farnesiana; decimalLatitude: 41.839667; decimalLongitude: 12.525528; geodeticDatum: WGS84; **Identification:** identifiedBy: Tommaso Fusco; dateIdentified: 2022; **Event:** samplingProtocol: Pitfall traps; eventDate: 2013-11-04; **Record Level:** collectionID: Roma3_5.8

##### Distribution

Southern Europe. S-European (SEU) chorotype.

#### Zelotes tenuis

(L. Koch, 1866)

7B5AF186-E501-594D-8FAB-2252A1223D20

##### Materials

- **Type status:** Other material. **Occurrence:** recordedBy: Fattorini S., Di Giulio A.; individualCount: 1; sex: male; lifeStage: adult; occurrenceID: 45BE1749-25B9-574E-8C97-5D433AE97B18; **Taxon:** scientificName: Zelotestenuis (L. Koch, 1866); order: Araneae; family: Gnaphosidae; genus: Zelotes; **Location:** country: Italy; countryCode: IT; stateProvince: Rome; county: Rome; municipality: Rome; locality: Appia Antica Regional Park, Rome; locationRemarks: Appia Antica; decimalLatitude: 41.812575; decimalLongitude: 12.564011; geodeticDatum: WGS84; **Identification:** identifiedBy: Tommaso Fusco; dateIdentified: 2022; **Event:** samplingProtocol: Pitfall traps; eventDate: 2014-06-13; **Record Level:** collectionID: Roma3_5.8
- **Type status:** Other material. **Occurrence:** recordedBy: Fattorini S., Di Giulio A.; individualCount: 1; sex: male; lifeStage: adult; occurrenceID: C74353A9-90DF-5993-84C8-151B8EE4EFCD; **Taxon:** scientificName: Zelotestenuis (L. Koch, 1866); order: Araneae; family: Gnaphosidae; genus: Zelotes; **Location:** country: Italy; countryCode: IT; stateProvince: Rome; county: Rome; municipality: Rome; locality: Appia Antica Regional Park, Rome; locationRemarks: Cava Fiorucci; decimalLatitude: 41.834106; decimalLongitude: 12.549264; geodeticDatum: WGS84; **Identification:** identifiedBy: Tommaso Fusco; dateIdentified: 2022; **Event:** samplingProtocol: Pitfall traps; eventDate: 2014-06-24; **Record Level:** collectionID: Roma3_5.8
- **Type status:** Other material. **Occurrence:** recordedBy: Fattorini S., Di Giulio A.; individualCount: 2; sex: 1 male, 1 female; lifeStage: adult; occurrenceID: C8FB965B-76FE-5DDB-9EB9-812FC83E9D2F; **Taxon:** scientificName: Zelotestenuis (L. Koch, 1866); order: Araneae; family: Gnaphosidae; genus: Zelotes; **Location:** country: Italy; countryCode: IT; stateProvince: Rome; county: Rome; municipality: Rome; locality: Appia Antica Regional Park, Rome; locationRemarks: Tor Marancia; decimalLatitude: 41.850308; decimalLongitude: 12.503178; geodeticDatum: WGS84; **Identification:** identifiedBy: Tommaso Fusco; dateIdentified: 2022; **Event:** samplingProtocol: Pitfall traps; eventDate: 2014-06-17; **Record Level:** collectionID: Roma3_5.8

##### Distribution

Mediterranean and central Europe to Caucasus. Introduced to Galapagos Is., USA. Europeo-Mediterranean (EUM) chorotype.

#### Hahniidae

Bertkau, 1878

B99D02BF-74E4-5F9A-BACA-BE4FDB97F7F4

#### Iberina candida

(Simon, 1875)

3ADB421F-4424-5C09-B9AA-9CCB2E416083

##### Materials

- **Type status:** Other material. **Occurrence:** recordedBy: Fattorini S., Di Giulio A.; individualCount: 1; sex: female; lifeStage: adult; occurrenceID: 672C29EE-07B8-5038-AD1F-1216E547092B; **Taxon:** scientificName: Iberinacandida (Simon, 1875); order: Araneae; family: Hahniidae; genus: Iberina; **Location:** country: Italy; countryCode: IT; stateProvince: Rome; county: Rome; municipality: Rome; locality: Appia Antica Regional Park, Rome; locationRemarks: San Sebastiano; decimalLatitude: 41.855733; decimalLongitude: 12.515114; geodeticDatum: WGS84; **Identification:** identifiedBy: Tommaso Fusco; dateIdentified: 2022; **Event:** samplingProtocol: Pitfall traps; eventDate: 2014-05-20; **Record Level:** collectionID: Roma3_5.8
- **Type status:** Other material. **Occurrence:** recordedBy: Fattorini S., Di Giulio A.; individualCount: 1; sex: female; lifeStage: adult; occurrenceID: 13840468-9BE4-5A92-8B73-27CD03F88C87; **Taxon:** scientificName: Iberinacandida (Simon, 1875); order: Araneae; family: Hahniidae; genus: Iberina; **Location:** country: Italy; countryCode: IT; stateProvince: Rome; county: Rome; municipality: Rome; locality: Appia Antica Regional Park, Rome; locationRemarks: Caffarella Sud 3; decimalLatitude: 41.856928; decimalLongitude: 12.528406; geodeticDatum: WGS84; **Identification:** identifiedBy: Tommaso Fusco; dateIdentified: 2022; **Event:** samplingProtocol: Pitfall traps; eventDate: 2014-05-19; **Record Level:** collectionID: Roma3_5.8

##### Distribution

North Africa, Europe, Turkey, Israel. Europeo-Mediterranean (EUM) chorotype.

#### Linyphiidae

Blackwall, 1859

0A51FA18-2720-5BF9-A75C-12795E9BE02A

#### Agyneta fuscipalpus

(C. L. Koch, 1836)

7CEE7B58-5A7C-5422-86F8-F1214FB9F350

##### Materials

- **Type status:** Other material. **Occurrence:** recordedBy: Fattorini S., Di Giulio A.; individualCount: 1; sex: male; lifeStage: adult; occurrenceID: 08A03C9B-7D43-5328-982B-BC5C57CD55DB; **Taxon:** scientificName: Agynetafuscipalpus (C. L. Koch, 1836); order: Araneae; family: Linyphiidae; genus: Agyneta; **Location:** country: Italy; countryCode: IT; stateProvince: Rome; county: Rome; municipality: Rome; locality: Appia Antica Regional Park, Rome; locationRemarks: Casal Verbeni; decimalLatitude: 41.815250; decimalLongitude: 12.552222; geodeticDatum: WGS84; **Identification:** identifiedBy: Tommaso Fusco; dateIdentified: 2022; **Event:** samplingProtocol: Pitfall traps; eventDate: 2014-06-13; **Record Level:** collectionID: Roma3_5.8

##### Distribution

Cabo Verde, Azores, Europe, North Africa, Caucasus, Russia (Europe to south Siberia), Iran, Central Asia. Palaearctic (PAL) chorotype.

#### Agyneta mollis

(O. Pickard-Cambridge, 1871)

27BE48A7-0926-53A7-9DED-AA6088048342

##### Materials

- **Type status:** Other material. **Occurrence:** recordedBy: Fattorini S., Di Giulio A.; individualCount: 1; sex: male; lifeStage: adult; occurrenceID: 368810F8-AECD-5DC5-BC42-118E839490BB; **Taxon:** scientificName: Agynetamollis (O. Pickard-Cambridge, 1871); order: Araneae; family: Linyphiidae; genus: Agyneta; **Location:** country: Italy; countryCode: IT; stateProvince: Rome; county: Rome; municipality: Rome; locality: Appia Antica Regional Park, Rome; locationRemarks: Caffarella Sud; decimalLatitude: 41.857247; decimalLongitude: 12.529211; geodeticDatum: WGS84; **Identification:** identifiedBy: Tommaso Fusco; dateIdentified: 2022; **Event:** samplingProtocol: Pitfall traps; eventDate: 2014-05-27; **Record Level:** collectionID: Roma3_5.8

##### Distribution

USA (Alaska), Canada, Europe, Morocco, Caucasus, Russia (Europe to Far East), Iran, China, Japan. Holarctic (OLA) chorotype.

#### Alioranus pauper

(Simon, 1881)

FD347E8A-DFAC-580F-A340-4658B27CDA55

##### Materials

- **Type status:** Other material. **Occurrence:** recordedBy: Fattorini S., Di Giulio A.; individualCount: 1; sex: male; lifeStage: adult; occurrenceID: A54B80C6-4972-5192-AF89-1D0372FA5791; **Taxon:** scientificName: Alioranuspauper (Simon, 1881); order: Araneae; family: Linyphiidae; genus: Alioranus; **Location:** country: Italy; countryCode: IT; stateProvince: Rome; county: Rome; municipality: Rome; locality: Appia Antica Regional Park, Rome; locationRemarks: Farnesiana; decimalLatitude: 41.839667; decimalLongitude: 12.525528; geodeticDatum: WGS84; **Identification:** identifiedBy: Tommaso Fusco; dateIdentified: 2022; **Event:** samplingProtocol: Pitfall traps; eventDate: 2014-05-28; **Record Level:** collectionID: Roma3_5.8
- **Type status:** Other material. **Occurrence:** recordedBy: Fattorini S., Di Giulio A.; individualCount: 1; sex: male; lifeStage: adult; occurrenceID: 8C0FCBE3-2934-5D41-85C2-D3DFB140E06B; **Taxon:** scientificName: Alioranuspauper (Simon, 1881); order: Araneae; family: Linyphiidae; genus: Alioranus; **Location:** country: Italy; countryCode: IT; stateProvince: Rome; county: Rome; municipality: Rome; locality: Appia Antica Regional Park, Rome; locationRemarks: Acqua Santa; decimalLatitude: 41.850561; decimalLongitude: 12.530861; geodeticDatum: WGS84; **Identification:** identifiedBy: Tommaso Fusco; dateIdentified: 2022; **Event:** samplingProtocol: Pitfall traps; eventDate: 2013-11-25; **Record Level:** collectionID: Roma3_5.8

##### Distribution

West Mediterranean from Portugal to Italy. W-Mediterranean (WME) chorotype.

#### Araeoncus humilis

(Blackwall, 1841)

A433A17E-2F76-527C-AFC2-8334DA891C2A

##### Materials

- **Type status:** Other material. **Occurrence:** recordedBy: Fattorini S., Di Giulio A.; individualCount: 1; sex: female; lifeStage: adult; occurrenceID: C3115BCE-E4DD-5596-8271-9CD7A40D8441; **Taxon:** scientificName: Araeoncushumilis (Blackwall, 1841); order: Araneae; family: Linyphiidae; genus: Araeoncus; **Location:** country: Italy; countryCode: IT; stateProvince: Rome; county: Rome; municipality: Rome; locality: Appia Antica Regional Park, Rome; locationRemarks: Farnesiana; decimalLatitude: 41.839667; decimalLongitude: 12.525528; geodeticDatum: WGS84; **Identification:** identifiedBy: Tommaso Fusco; dateIdentified: 2022; **Event:** samplingProtocol: Pitfall traps; eventDate: 2014-06-19; **Record Level:** collectionID: Roma3_5.8

##### Distribution

Europe, North Africa, Russia (Europe to south Siberia), Iran, Japan. Palaearctic (PAL) chorotype.

#### Araeoncus longiusculus

(O. Pickard-Cambridge, 1875)

5A5D3F48-522B-5D4E-A950-B06122D60291

##### Materials

- **Type status:** Other material. **Occurrence:** recordedBy: Fattorini S., Di Giulio A.; individualCount: 1; sex: female; lifeStage: adult; occurrenceID: 0F40AC79-1B8F-5F19-8F56-2519B71809E8; **Taxon:** scientificName: Araeoncuslongiusculus (O. Pickard-Cambridge, 1875); order: Araneae; family: Linyphiidae; genus: Araeoncus; **Location:** country: Italy; countryCode: IT; stateProvince: Rome; county: Rome; municipality: Rome; locality: Appia Antica Regional Park, Rome; locationRemarks: Acqua Santa; decimalLatitude: 41.850561; decimalLongitude: 12.530861; geodeticDatum: WGS84; **Identification:** identifiedBy: Tommaso Fusco; dateIdentified: 2022; **Event:** samplingProtocol: Pitfall traps; eventDate: 2014-06-19; **Record Level:** collectionID: Roma3_5.8
- **Type status:** Other material. **Occurrence:** recordedBy: Fattorini S., Di Giulio A.; individualCount: 3; sex: male; lifeStage: adult; occurrenceID: F82C0E7B-75A4-592C-B85F-37C92A3285D5; **Taxon:** scientificName: Araeoncuslongiusculus (O. Pickard-Cambridge, 1875); order: Araneae; family: Linyphiidae; genus: Araeoncus; **Location:** country: Italy; countryCode: IT; stateProvince: Rome; county: Rome; municipality: Rome; locality: Appia Antica Regional Park, Rome; locationRemarks: Appia Antica; decimalLatitude: 41.812575; decimalLongitude: 12.564011; geodeticDatum: WGS84; **Identification:** identifiedBy: Tommaso Fusco; dateIdentified: 2022; **Event:** samplingProtocol: Pitfall traps; eventDate: 2013-11-27; **Record Level:** collectionID: Roma3_5.8
- **Type status:** Other material. **Occurrence:** recordedBy: Fattorini S., Di Giulio A.; individualCount: 1; sex: male; lifeStage: adult; occurrenceID: 284F665B-97E3-5FD2-924C-4609989108F4; **Taxon:** scientificName: Araeoncuslongiusculus (O. Pickard-Cambridge, 1875); order: Araneae; family: Linyphiidae; genus: Araeoncus; **Location:** country: Italy; countryCode: IT; stateProvince: Rome; county: Rome; municipality: Rome; locality: Appia Antica Regional Park, Rome; locationRemarks: Appia Antica; decimalLatitude: 41.812575; decimalLongitude: 12.564011; geodeticDatum: WGS84; **Identification:** identifiedBy: Tommaso Fusco; dateIdentified: 2022; **Event:** samplingProtocol: Pitfall traps; eventDate: 2013-12-06; **Record Level:** collectionID: Roma3_5.8
- **Type status:** Other material. **Occurrence:** recordedBy: Fattorini S., Di Giulio A.; individualCount: 1; sex: male; lifeStage: adult; occurrenceID: F74F54C2-1863-5205-A47B-079AA5CC5E41; **Taxon:** scientificName: Araeoncuslongiusculus (O. Pickard-Cambridge, 1875); order: Araneae; family: Linyphiidae; genus: Araeoncus; **Location:** country: Italy; countryCode: IT; stateProvince: Rome; county: Rome; municipality: Rome; locality: Appia Antica Regional Park, Rome; locationRemarks: Caffarella Sud; decimalLatitude: 41.857247; decimalLongitude: 12.529211; geodeticDatum: WGS84; **Identification:** identifiedBy: Tommaso Fusco; dateIdentified: 2022; **Event:** samplingProtocol: Pitfall traps; eventDate: 2013-10-31; **Record Level:** collectionID: Roma3_5.8
- **Type status:** Other material. **Occurrence:** recordedBy: Fattorini S., Di Giulio A.; individualCount: 2; sex: 1 male, 1 female; lifeStage: adult; occurrenceID: 3FC48372-62DF-5BFD-B774-F7B5A39A7B45; **Taxon:** scientificName: Araeoncuslongiusculus (O. Pickard-Cambridge, 1875); order: Araneae; family: Linyphiidae; genus: Araeoncus; **Location:** country: Italy; countryCode: IT; stateProvince: Rome; county: Rome; municipality: Rome; locality: Appia Antica Regional Park, Rome; locationRemarks: Caffarella Sud; decimalLatitude: 41.857247; decimalLongitude: 12.529211; geodeticDatum: WGS84; **Identification:** identifiedBy: Tommaso Fusco; dateIdentified: 2022; **Event:** samplingProtocol: Pitfall traps; eventDate: 2013-11-30; **Record Level:** collectionID: Roma3_5.8
- **Type status:** Other material. **Occurrence:** recordedBy: Fattorini S., Di Giulio A.; individualCount: 1; sex: male; lifeStage: adult; occurrenceID: A4D3CC27-3178-5C27-BDF0-204FF875C770; **Taxon:** scientificName: Araeoncuslongiusculus (O. Pickard-Cambridge, 1875); order: Araneae; family: Linyphiidae; genus: Araeoncus; **Location:** country: Italy; countryCode: IT; stateProvince: Rome; county: Rome; municipality: Rome; locality: Appia Antica Regional Park, Rome; locationRemarks: Caffarella Sud; decimalLatitude: 41.857247; decimalLongitude: 12.529211; geodeticDatum: WGS84; **Identification:** identifiedBy: Tommaso Fusco; dateIdentified: 2022; **Event:** samplingProtocol: Pitfall traps; eventDate: 2013-11-12; **Record Level:** collectionID: Roma3_5.8
- **Type status:** Other material. **Occurrence:** recordedBy: Fattorini S., Di Giulio A.; individualCount: 1; sex: male; lifeStage: adult; occurrenceID: F933F55A-8214-5D9E-8297-7AA0A19F3567; **Taxon:** scientificName: Araeoncuslongiusculus (O. Pickard-Cambridge, 1875); order: Araneae; family: Linyphiidae; genus: Araeoncus; **Location:** country: Italy; countryCode: IT; stateProvince: Rome; county: Rome; municipality: Rome; locality: Appia Antica Regional Park, Rome; locationRemarks: Caffarella Sud 3; decimalLatitude: 41.856928; decimalLongitude: 12.528406; geodeticDatum: WGS84; **Identification:** identifiedBy: Tommaso Fusco; dateIdentified: 2022; **Event:** samplingProtocol: Pitfall traps; eventDate: 2014-05-27; **Record Level:** collectionID: Roma3_5.8
- **Type status:** Other material. **Occurrence:** recordedBy: Fattorini S., Di Giulio A.; individualCount: 1; sex: male; lifeStage: adult; occurrenceID: 76553039-3AA0-5373-B858-398FDD775273; **Taxon:** scientificName: Araeoncuslongiusculus (O. Pickard-Cambridge, 1875); order: Araneae; family: Linyphiidae; genus: Araeoncus; **Location:** country: Italy; countryCode: IT; stateProvince: Rome; county: Rome; municipality: Rome; locality: Appia Antica Regional Park, Rome; locationRemarks: Tor Marancia; decimalLatitude: 41.850308; decimalLongitude: 12.503178; geodeticDatum: WGS84; **Identification:** identifiedBy: Tommaso Fusco; dateIdentified: 2022; **Event:** samplingProtocol: Pitfall traps; eventDate: 2014-06-05; **Record Level:** collectionID: Roma3_5.8
- **Type status:** Other material. **Occurrence:** recordedBy: Fattorini S., Di Giulio A.; individualCount: 1; sex: male; lifeStage: adult; occurrenceID: 4A47837C-294A-5E8D-BE0B-8F81BCF2DC8C; **Taxon:** scientificName: Araeoncuslongiusculus (O. Pickard-Cambridge, 1875); order: Araneae; family: Linyphiidae; genus: Araeoncus; **Location:** country: Italy; countryCode: IT; stateProvince: Rome; county: Rome; municipality: Rome; locality: Appia Antica Regional Park, Rome; locationRemarks: Torre Selce; decimalLatitude: 41.816611; decimalLongitude: 12.560667; geodeticDatum: WGS84; **Identification:** identifiedBy: Tommaso Fusco; dateIdentified: 2022; **Event:** samplingProtocol: Pitfall traps; eventDate: 2013-12-06; **Record Level:** collectionID: Roma3_5.8

##### Distribution

Only found in Corsica, Sardinia and mainland Italy. W-Mediterranean (WME) chorotype.

#### Centromerus sylvaticus

(Blackwall, 1841)

D10B3157-549A-5090-A6EE-A5092C53384E

##### Materials

- **Type status:** Other material. **Occurrence:** recordedBy: Fattorini S., Di Giulio A.; individualCount: 1; sex: female; lifeStage: adult; occurrenceID: C37B005A-C2A2-5B0F-86A7-E1A72766C828; **Taxon:** scientificName: Centromerussylvaticus (Blackwall, 1841); order: Araneae; family: Linyphiidae; genus: Centromerus; **Location:** country: Italy; countryCode: IT; stateProvince: Rome; county: Rome; municipality: Rome; locality: Appia Antica Regional Park, Rome; locationRemarks: Tor Marancia; decimalLatitude: 41.850308; decimalLongitude: 12.503178; geodeticDatum: WGS84; **Identification:** identifiedBy: Tommaso Fusco; dateIdentified: 2022; **Event:** samplingProtocol: Pitfall traps; eventDate: 2014-05-26; **Record Level:** collectionID: Roma3_5.8

##### Distribution

North America, Europe, Russia (Europe to Far East), Turkey, Caucasus, Cina, Korea, Japan. Holarctic (OLA) chorotype.

#### Centromerus tongiorgii

Ballarin & Pantini, 2020

2A9BA29C-E83B-56BC-A84F-2D36AC96A777

##### Materials

- **Type status:** Other material. **Occurrence:** recordedBy: Fattorini S., Di Giulio A.; individualCount: 1; sex: female; lifeStage: adult; occurrenceID: 07B1CA73-F7DA-598F-A948-C99C6DCCAC91; **Taxon:** scientificName: Centromerustongiorgii Ballarin & Pantini, 2020; order: Araneae; family: Linyphiidae; genus: Centromerus; **Location:** country: Italy; countryCode: IT; stateProvince: Rome; county: Rome; municipality: Rome; locality: Appia Antica Regional Park, Rome; locationRemarks: Tor Marancia; decimalLatitude: 41.850308; decimalLongitude: 12.503178; geodeticDatum: WGS84; **Identification:** identifiedBy: Tommaso Fusco; dateIdentified: 2022; **Event:** samplingProtocol: Pitfall traps; eventDate: 2014-05-27; **Record Level:** collectionID: Roma3_5.8
- **Type status:** Other material. **Occurrence:** recordedBy: Fattorini S., Di Giulio A.; individualCount: 2; sex: female; lifeStage: adult; occurrenceID: 9E561AA1-C435-5EB7-9992-CC9EC2FEEE9F; **Taxon:** scientificName: Centromerustongiorgii Ballarin & Pantini, 2020; order: Araneae; family: Linyphiidae; genus: Centromerus; **Location:** country: Italy; countryCode: IT; stateProvince: Rome; county: Rome; municipality: Rome; locality: Appia Antica Regional Park, Rome; locationRemarks: Caffarella Centro; decimalLatitude: 41.864889; decimalLongitude: 12.516389; geodeticDatum: WGS84; **Identification:** identifiedBy: Tommaso Fusco; dateIdentified: 2022; **Event:** samplingProtocol: Pitfall traps; eventDate: 2014-05-27; **Record Level:** collectionID: Roma3_5.8
- **Type status:** Other material. **Occurrence:** recordedBy: Fattorini S., Di Giulio A.; individualCount: 1; sex: female; lifeStage: adult; occurrenceID: C066E781-52DF-587A-BEAE-66C996ACD854; **Taxon:** scientificName: Centromerustongiorgii Ballarin & Pantini, 2020; order: Araneae; family: Linyphiidae; genus: Centromerus; **Location:** country: Italy; countryCode: IT; stateProvince: Rome; county: Rome; municipality: Rome; locality: Appia Antica Regional Park, Rome; locationRemarks: Caffarella Centro; decimalLatitude: 41.864889; decimalLongitude: 12.516389; geodeticDatum: WGS84; **Identification:** identifiedBy: Tommaso Fusco; dateIdentified: 2022; **Event:** samplingProtocol: Pitfall traps; eventDate: 2014-05-19; **Record Level:** collectionID: Roma3_5.8

##### Distribution

Italian Endemic (END) from north to central Italy (Ballarin and Pantini 2020).

#### Ceratinella brevis

(Wider, 1834)

7C4F8AE0-FAF1-5B89-AD96-22607AA19DB2

##### Materials

- **Type status:** Other material. **Occurrence:** recordedBy: Fattorini S., Di Giulio A.; individualCount: 1; sex: female; lifeStage: adult; occurrenceID: D823EE4B-776E-5C2A-B51E-AC84A9151969; **Taxon:** scientificName: Ceratinellabrevis (Wider, 1834); order: Araneae; family: Linyphiidae; genus: Ceratinella; **Location:** country: Italy; countryCode: IT; stateProvince: Rome; county: Rome; municipality: Rome; locality: Appia Antica Regional Park, Rome; locationRemarks: Caffarella Nord; decimalLatitude: 41.867753; decimalLongitude: 12.512414; geodeticDatum: WGS84; **Identification:** identifiedBy: Tommaso Fusco; dateIdentified: 2022; **Event:** samplingProtocol: Pitfall traps; eventDate: 2014-05-27; **Record Level:** collectionID: Roma3_5.8

##### Distribution

Europe, Russia (Europe to Far East), Caucasus, Turkey, Iran, Central Asia, China, Korea, Japan. Palaearctic (PAL) chorotype.

#### Diplocephalus graecus

(O. Pickard-Cambridge, 1873)

86A81186-DBF5-5047-A1E3-6AD4DE0840ED

##### Materials

- **Type status:** Other material. **Occurrence:** recordedBy: Fattorini S., Di Giulio A.; individualCount: 1; sex: female; lifeStage: adult; occurrenceID: 2C2FF12E-82DB-5022-9AB3-DBE1999FB27C; **Taxon:** scientificName: Diplocephalusgraecus (O. Pickard-Cambridge, 1873); order: Araneae; family: Linyphiidae; genus: Diplocephalus; **Location:** country: Italy; countryCode: IT; stateProvince: Rome; county: Rome; municipality: Rome; locality: Appia Antica Regional Park, Rome; locationRemarks: Tor Marancia; decimalLatitude: 41.850308; decimalLongitude: 12.503178; geodeticDatum: WGS84; **Identification:** identifiedBy: Tommaso Fusco; dateIdentified: 2022; **Event:** samplingProtocol: Pitfall traps; eventDate: 2014-05-26; **Record Level:** collectionID: Roma3_5.8
- **Type status:** Other material. **Occurrence:** recordedBy: Fattorini S., Di Giulio A.; individualCount: 1; sex: male; lifeStage: adult; occurrenceID: E8A68361-152D-5057-9BA9-75518256521D; **Taxon:** scientificName: Diplocephalusgraecus (O. Pickard-Cambridge, 1873); order: Araneae; family: Linyphiidae; genus: Diplocephalus; **Location:** country: Italy; countryCode: IT; stateProvince: Rome; county: Rome; municipality: Rome; locality: Appia Antica Regional Park, Rome; locationRemarks: Caffarella Sud 3; decimalLatitude: 41.856928; decimalLongitude: 12.528406; geodeticDatum: WGS84; **Identification:** identifiedBy: Tommaso Fusco; dateIdentified: 2022; **Event:** samplingProtocol: Pitfall traps; eventDate: 2014-05-19; **Record Level:** collectionID: Roma3_5.8

##### Distribution

Europe, North Africa, Turkey, Israel. Europeo-Mediterranean (EUM) chorotype.

#### Diplostyla concolor

(Wider, 1834)

8AD02196-0070-56DB-B779-FAE1D6C98140

##### Materials

- **Type status:** Other material. **Occurrence:** recordedBy: Fattorini S., Di Giulio A.; individualCount: 1; sex: female; lifeStage: adult; occurrenceID: 0E8DDDCD-67EE-59F7-A800-27150A1661BD; **Taxon:** scientificName: Diplostylaconcolor (Wider, 1834); order: Araneae; family: Linyphiidae; genus: Diplostyla; **Location:** country: Italy; countryCode: IT; stateProvince: Rome; county: Rome; municipality: Rome; locality: Appia Antica Regional Park, Rome; locationRemarks: Acqua Santa; decimalLatitude: 41.850561; decimalLongitude: 12.530861; geodeticDatum: WGS84; **Identification:** identifiedBy: Tommaso Fusco; dateIdentified: 2022; **Event:** samplingProtocol: Pitfall traps; eventDate: 2014-05-20; **Record Level:** collectionID: Roma3_5.8
- **Type status:** Other material. **Occurrence:** recordedBy: Fattorini S., Di Giulio A.; individualCount: 3; sex: 2 male, 1 female; lifeStage: adult; occurrenceID: 314C9325-5EE2-53C6-A36B-EB523BFB307B; **Taxon:** scientificName: Diplostylaconcolor (Wider, 1834); order: Araneae; family: Linyphiidae; genus: Diplostyla; **Location:** country: Italy; countryCode: IT; stateProvince: Rome; county: Rome; municipality: Rome; locality: Appia Antica Regional Park, Rome; locationRemarks: Acqua Santa; decimalLatitude: 41.850561; decimalLongitude: 12.530861; geodeticDatum: WGS84; **Identification:** identifiedBy: Tommaso Fusco; dateIdentified: 2022; **Event:** samplingProtocol: Pitfall traps; eventDate: 2014-05-28; **Record Level:** collectionID: Roma3_5.8
- **Type status:** Other material. **Occurrence:** recordedBy: Fattorini S., Di Giulio A.; individualCount: 16; sex: 8 male, 8 female; lifeStage: adult; occurrenceID: 1A5827AC-6E18-545A-95D2-296FF0797F12; **Taxon:** scientificName: Diplostylaconcolor (Wider, 1834); order: Araneae; family: Linyphiidae; genus: Diplostyla; **Location:** country: Italy; countryCode: IT; stateProvince: Rome; county: Rome; municipality: Rome; locality: Appia Antica Regional Park, Rome; locationRemarks: Acqua Santa; decimalLatitude: 41.850561; decimalLongitude: 12.530861; geodeticDatum: WGS84; **Identification:** identifiedBy: Tommaso Fusco; dateIdentified: 2022; **Event:** samplingProtocol: Pitfall traps; eventDate: 2014-06-09; **Record Level:** collectionID: Roma3_5.8
- **Type status:** Other material. **Occurrence:** recordedBy: Fattorini S., Di Giulio A.; individualCount: 2; sex: 1 male, 1 female; lifeStage: adult; occurrenceID: DB160545-1831-5772-A50D-B624633C1F58; **Taxon:** scientificName: Diplostylaconcolor (Wider, 1834); order: Araneae; family: Linyphiidae; genus: Diplostyla; **Location:** country: Italy; countryCode: IT; stateProvince: Rome; county: Rome; municipality: Rome; locality: Appia Antica Regional Park, Rome; locationRemarks: Acqua Santa; decimalLatitude: 41.850561; decimalLongitude: 12.530861; geodeticDatum: WGS84; **Identification:** identifiedBy: Tommaso Fusco; dateIdentified: 2022; **Event:** samplingProtocol: Pitfall traps; eventDate: 2014-06-19; **Record Level:** collectionID: Roma3_5.8
- **Type status:** Other material. **Occurrence:** recordedBy: Fattorini S., Di Giulio A.; individualCount: 1; sex: female; lifeStage: adult; occurrenceID: 093C948A-3735-54C8-82D2-86B707583A66; **Taxon:** scientificName: Diplostylaconcolor (Wider, 1834); order: Araneae; family: Linyphiidae; genus: Diplostyla; **Location:** country: Italy; countryCode: IT; stateProvince: Rome; county: Rome; municipality: Rome; locality: Appia Antica Regional Park, Rome; locationRemarks: Caffarella Nord; decimalLatitude: 41.867753; decimalLongitude: 12.512414; geodeticDatum: WGS84; **Identification:** identifiedBy: Tommaso Fusco; dateIdentified: 2022; **Event:** samplingProtocol: Pitfall traps; eventDate: 2013-10-31; **Record Level:** collectionID: Roma3_5.8
- **Type status:** Other material. **Occurrence:** recordedBy: Fattorini S., Di Giulio A.; individualCount: 3; sex: 2 male, 1 female; lifeStage: adult; occurrenceID: D227FDFD-9494-5CDC-98E8-13C61874CA97; **Taxon:** scientificName: Diplostylaconcolor (Wider, 1834); order: Araneae; family: Linyphiidae; genus: Diplostyla; **Location:** country: Italy; countryCode: IT; stateProvince: Rome; county: Rome; municipality: Rome; locality: Appia Antica Regional Park, Rome; locationRemarks: Caffarella Nord; decimalLatitude: 41.867753; decimalLongitude: 12.512414; geodeticDatum: WGS84; **Identification:** identifiedBy: Tommaso Fusco; dateIdentified: 2022; **Event:** samplingProtocol: Pitfall traps; eventDate: 2014-05-27; **Record Level:** collectionID: Roma3_5.8
- **Type status:** Other material. **Occurrence:** recordedBy: Fattorini S., Di Giulio A.; individualCount: 2; sex: male; lifeStage: adult; occurrenceID: DE7EBE8B-9D72-5E24-B735-37B5F8D68D29; **Taxon:** scientificName: Diplostylaconcolor (Wider, 1834); order: Araneae; family: Linyphiidae; genus: Diplostyla; **Location:** country: Italy; countryCode: IT; stateProvince: Rome; county: Rome; municipality: Rome; locality: Appia Antica Regional Park, Rome; locationRemarks: Caffarella Nord; decimalLatitude: 41.867753; decimalLongitude: 12.512414; geodeticDatum: WGS84; **Identification:** identifiedBy: Tommaso Fusco; dateIdentified: 2022; **Event:** samplingProtocol: Pitfall traps; eventDate: 2014-06-06; **Record Level:** collectionID: Roma3_5.8
- **Type status:** Other material. **Occurrence:** recordedBy: Fattorini S., Di Giulio A.; individualCount: 6; sex: 3 male, 3 female; lifeStage: adult; occurrenceID: 318E1CB6-C836-5783-A18D-B1AAA553A1D2; **Taxon:** scientificName: Diplostylaconcolor (Wider, 1834); order: Araneae; family: Linyphiidae; genus: Diplostyla; **Location:** country: Italy; countryCode: IT; stateProvince: Rome; county: Rome; municipality: Rome; locality: Appia Antica Regional Park, Rome; locationRemarks: Caffarella Nord; decimalLatitude: 41.867753; decimalLongitude: 12.512414; geodeticDatum: WGS84; **Identification:** identifiedBy: Tommaso Fusco; dateIdentified: 2022; **Event:** samplingProtocol: Pitfall traps; eventDate: 2014-06-18; **Record Level:** collectionID: Roma3_5.8
- **Type status:** Other material. **Occurrence:** recordedBy: Fattorini S., Di Giulio A.; individualCount: 1; sex: female; lifeStage: adult; occurrenceID: 24DBC1CC-D368-5936-ABFC-3F62A0818104; **Taxon:** scientificName: Diplostylaconcolor (Wider, 1834); order: Araneae; family: Linyphiidae; genus: Diplostyla; **Location:** country: Italy; countryCode: IT; stateProvince: Rome; county: Rome; municipality: Rome; locality: Appia Antica Regional Park, Rome; locationRemarks: Caffarella Sud 1; decimalLatitude: 41.857247; decimalLongitude: 12.529211; geodeticDatum: WGS84; **Identification:** identifiedBy: Tommaso Fusco; dateIdentified: 2022; **Event:** samplingProtocol: Pitfall traps; eventDate: 2013-10-31; **Record Level:** collectionID: Roma3_5.8
- **Type status:** Other material. **Occurrence:** recordedBy: Fattorini S., Di Giulio A.; individualCount: 2; sex: 1 male, 1 female; lifeStage: adult; occurrenceID: C0674BE3-5835-5FA3-AE9D-9C8981D17130; **Taxon:** scientificName: Diplostylaconcolor (Wider, 1834); order: Araneae; family: Linyphiidae; genus: Diplostyla; **Location:** country: Italy; countryCode: IT; stateProvince: Rome; county: Rome; municipality: Rome; locality: Appia Antica Regional Park, Rome; locationRemarks: Caffarella Sud 1; decimalLatitude: 41.857247; decimalLongitude: 12.529211; geodeticDatum: WGS84; **Identification:** identifiedBy: Tommaso Fusco; dateIdentified: 2022; **Event:** samplingProtocol: Pitfall traps; eventDate: 2014-06-18; **Record Level:** collectionID: Roma3_5.8
- **Type status:** Other material. **Occurrence:** recordedBy: Fattorini S., Di Giulio A.; individualCount: 2; sex: 1 male, 1 female; lifeStage: adult; occurrenceID: EA2D254B-5F61-5576-A2FF-E0626F5A0FAC; **Taxon:** scientificName: Diplostylaconcolor (Wider, 1834); order: Araneae; family: Linyphiidae; genus: Diplostyla; **Location:** country: Italy; countryCode: IT; stateProvince: Rome; county: Rome; municipality: Rome; locality: Appia Antica Regional Park, Rome; locationRemarks: Caffarella Sud 2; decimalLatitude: 41.856742; decimalLongitude: 12.529453; geodeticDatum: WGS84; **Identification:** identifiedBy: Tommaso Fusco; dateIdentified: 2022; **Event:** samplingProtocol: Pitfall traps; eventDate: 2014-05-19; **Record Level:** collectionID: Roma3_5.8
- **Type status:** Other material. **Occurrence:** recordedBy: Fattorini S., Di Giulio A.; individualCount: 5; sex: 2 male, 3 female; lifeStage: adult; occurrenceID: C90A1470-5DB2-5E36-90A4-429BD4A9E36C; **Taxon:** scientificName: Diplostylaconcolor (Wider, 1834); order: Araneae; family: Linyphiidae; genus: Diplostyla; **Location:** country: Italy; countryCode: IT; stateProvince: Rome; county: Rome; municipality: Rome; locality: Appia Antica Regional Park, Rome; locationRemarks: Caffarella Sud 2; decimalLatitude: 41.856742; decimalLongitude: 12.529453; geodeticDatum: WGS84; **Identification:** identifiedBy: Tommaso Fusco; dateIdentified: 2022; **Event:** samplingProtocol: Pitfall traps; eventDate: 2014-05-27; **Record Level:** collectionID: Roma3_5.8
- **Type status:** Other material. **Occurrence:** recordedBy: Fattorini S., Di Giulio A.; individualCount: 2; sex: 2 male, 2 female; lifeStage: adult; occurrenceID: 290ADCA4-E645-52B7-B05D-1179B05855DF; **Taxon:** scientificName: Diplostylaconcolor (Wider, 1834); order: Araneae; family: Linyphiidae; genus: Diplostyla; **Location:** country: Italy; countryCode: IT; stateProvince: Rome; county: Rome; municipality: Rome; locality: Appia Antica Regional Park, Rome; locationRemarks: Caffarella Sud 2; decimalLatitude: 41.856742; decimalLongitude: 12.529453; geodeticDatum: WGS84; **Identification:** identifiedBy: Tommaso Fusco; dateIdentified: 2022; **Event:** samplingProtocol: Pitfall traps; eventDate: 2014-06-06; **Record Level:** collectionID: Roma3_5.8
- **Type status:** Other material. **Occurrence:** recordedBy: Fattorini S., Di Giulio A.; individualCount: 3; sex: 1 male, 2 female; lifeStage: adult; occurrenceID: 9C591C05-DB44-5624-B5CD-B7DAEAA0C83B; **Taxon:** scientificName: Diplostylaconcolor (Wider, 1834); order: Araneae; family: Linyphiidae; genus: Diplostyla; **Location:** country: Italy; countryCode: IT; stateProvince: Rome; county: Rome; municipality: Rome; locality: Appia Antica Regional Park, Rome; locationRemarks: Caffarella Sud 2; decimalLatitude: 41.856742; decimalLongitude: 12.529453; geodeticDatum: WGS84; **Identification:** identifiedBy: Tommaso Fusco; dateIdentified: 2022; **Event:** samplingProtocol: Pitfall traps; eventDate: 2014-06-18; **Record Level:** collectionID: Roma3_5.8
- **Type status:** Other material. **Occurrence:** recordedBy: Fattorini S., Di Giulio A.; individualCount: 1; sex: female; lifeStage: adult; occurrenceID: D2400B26-3899-5BED-B5E0-616C9C1C1260; **Taxon:** scientificName: Diplostylaconcolor (Wider, 1834); order: Araneae; family: Linyphiidae; genus: Diplostyla; **Location:** country: Italy; countryCode: IT; stateProvince: Rome; county: Rome; municipality: Rome; locality: Appia Antica Regional Park, Rome; locationRemarks: Caffarella Sud 3; decimalLatitude: 41.856928; decimalLongitude: 12.528406; geodeticDatum: WGS84; **Identification:** identifiedBy: Tommaso Fusco; dateIdentified: 2022; **Event:** samplingProtocol: Pitfall traps; eventDate: 2014-05-19; **Record Level:** collectionID: Roma3_5.8
- **Type status:** Other material. **Occurrence:** recordedBy: Fattorini S., Di Giulio A.; individualCount: 1; sex: female; lifeStage: adult; occurrenceID: 963CBDE4-9C5A-553B-9321-51F5A71E6CAC; **Taxon:** scientificName: Diplostylaconcolor (Wider, 1834); order: Araneae; family: Linyphiidae; genus: Diplostyla; **Location:** country: Italy; countryCode: IT; stateProvince: Rome; county: Rome; municipality: Rome; locality: Appia Antica Regional Park, Rome; locationRemarks: Caffarella Sud 3; decimalLatitude: 41.856928; decimalLongitude: 12.528406; geodeticDatum: WGS84; **Identification:** identifiedBy: Tommaso Fusco; dateIdentified: 2022; **Event:** samplingProtocol: Pitfall traps; eventDate: 2014-06-18; **Record Level:** collectionID: Roma3_5.8
- **Type status:** Other material. **Occurrence:** recordedBy: Fattorini S., Di Giulio A.; individualCount: 1; sex: male; lifeStage: adult; occurrenceID: A1A20A30-CE32-56DB-BD97-E21609D592B1; **Taxon:** scientificName: Diplostylaconcolor (Wider, 1834); order: Araneae; family: Linyphiidae; genus: Diplostyla; **Location:** country: Italy; countryCode: IT; stateProvince: Rome; county: Rome; municipality: Rome; locality: Appia Antica Regional Park, Rome; locationRemarks: Casal Verbeni; decimalLatitude: 41.815250; decimalLongitude: 12.552222; geodeticDatum: WGS84; **Identification:** identifiedBy: Tommaso Fusco; dateIdentified: 2022; **Event:** samplingProtocol: Pitfall traps; eventDate: 2014-05-26; **Record Level:** collectionID: Roma3_5.8
- **Type status:** Other material. **Occurrence:** recordedBy: Fattorini S., Di Giulio A.; individualCount: 1; sex: female; lifeStage: adult; occurrenceID: 0B87C85C-27AE-5D1F-A7B6-BDC12C09C9CA; **Taxon:** scientificName: Diplostylaconcolor (Wider, 1834); order: Araneae; family: Linyphiidae; genus: Diplostyla; **Location:** country: Italy; countryCode: IT; stateProvince: Rome; county: Rome; municipality: Rome; locality: Appia Antica Regional Park, Rome; locationRemarks: Casal Verbeni; decimalLatitude: 41.815250; decimalLongitude: 12.552222; geodeticDatum: WGS84; **Identification:** identifiedBy: Tommaso Fusco; dateIdentified: 2022; **Event:** samplingProtocol: Pitfall traps; eventDate: 2013-11-18; **Record Level:** collectionID: Roma3_5.8
- **Type status:** Other material. **Occurrence:** recordedBy: Fattorini S., Di Giulio A.; individualCount: 1; sex: female; lifeStage: adult; occurrenceID: 853E13CB-9698-5F93-89A0-A748C5C2FB8D; **Taxon:** scientificName: Diplostylaconcolor (Wider, 1834); order: Araneae; family: Linyphiidae; genus: Diplostyla; **Location:** country: Italy; countryCode: IT; stateProvince: Rome; county: Rome; municipality: Rome; locality: Appia Antica Regional Park, Rome; locationRemarks: Cava Fiorucci; decimalLatitude: 41.834106; decimalLongitude: 12.549264; geodeticDatum: WGS84; **Identification:** identifiedBy: Tommaso Fusco; dateIdentified: 2022; **Event:** samplingProtocol: Pitfall traps; eventDate: 2014-06-13; **Record Level:** collectionID: Roma3_5.8
- **Type status:** Other material. **Occurrence:** recordedBy: Fattorini S., Di Giulio A.; individualCount: 1; sex: male; lifeStage: adult; occurrenceID: 11E3293B-AA2D-55D7-9BEA-4A838465BA09; **Taxon:** scientificName: Diplostylaconcolor (Wider, 1834); order: Araneae; family: Linyphiidae; genus: Diplostyla; **Location:** country: Italy; countryCode: IT; stateProvince: Rome; county: Rome; municipality: Rome; locality: Appia Antica Regional Park, Rome; locationRemarks: Farnesiana; decimalLatitude: 41.839667; decimalLongitude: 12.525528; geodeticDatum: WGS84; **Identification:** identifiedBy: Tommaso Fusco; dateIdentified: 2022; **Event:** samplingProtocol: Pitfall traps; eventDate: 2014-06-19; **Record Level:** collectionID: Roma3_5.8

##### Distribution

North America, Europe, Turkey, Caucasus, Russia (Europe to Far East), Iran, Korea. Holarctic (OLA) chorotype.

#### Erigone autumnalis

Emerton, 1882

C4E6996C-B286-5FC6-A4A2-880D507A064B

##### Materials

- **Type status:** Other material. **Occurrence:** recordedBy: Fattorini S., Di Giulio A.; individualCount: 1; sex: female; lifeStage: adult; occurrenceID: 825BC6F5-89C9-54F1-B915-9612EEA06397; **Taxon:** scientificName: Erigoneautumnalis Emerton, 1882; order: Araneae; family: Linyphiidae; genus: Erigone; **Location:** country: Italy; countryCode: IT; stateProvince: Rome; county: Rome; municipality: Rome; locality: Appia Antica Regional Park, Rome; locationRemarks: Casal Verbeni; decimalLatitude: 41.815250; decimalLongitude: 12.552222; geodeticDatum: WGS84; **Identification:** identifiedBy: Tommaso Fusco; dateIdentified: 2022; **Event:** samplingProtocol: Pitfall traps; eventDate: 2014-06-05; **Record Level:** collectionID: Roma3_5.8
- **Type status:** Other material. **Occurrence:** recordedBy: Fattorini S., Di Giulio A.; individualCount: 1; sex: male; lifeStage: adult; occurrenceID: 012C41C4-453B-520A-82BA-C14ABB142D52; **Taxon:** scientificName: Erigoneautumnalis Emerton, 1882; order: Araneae; family: Linyphiidae; genus: Erigone; **Location:** country: Italy; countryCode: IT; stateProvince: Rome; county: Rome; municipality: Rome; locality: Appia Antica Regional Park, Rome; locationRemarks: Acqua Santa; decimalLatitude: 41.850561; decimalLongitude: 12.530861; geodeticDatum: WGS84; **Identification:** identifiedBy: Tommaso Fusco; dateIdentified: 2022; **Event:** samplingProtocol: Pitfall traps; eventDate: 2014-05-28; **Record Level:** collectionID: Roma3_5.8
- **Type status:** Other material. **Occurrence:** recordedBy: Fattorini S., Di Giulio A.; individualCount: 1; sex: male; lifeStage: adult; occurrenceID: 702CA147-7A92-5965-A9D6-8DCB756A9734; **Taxon:** scientificName: Erigoneautumnalis Emerton, 1882; order: Araneae; family: Linyphiidae; genus: Erigone; **Location:** country: Italy; countryCode: IT; stateProvince: Rome; county: Rome; municipality: Rome; locality: Appia Antica Regional Park, Rome; locationRemarks: Caffarella Sud 3; decimalLatitude: 41.856928; decimalLongitude: 12.528406; geodeticDatum: WGS84; **Identification:** identifiedBy: Tommaso Fusco; dateIdentified: 2022; **Event:** samplingProtocol: Pitfall traps; eventDate: 2014-05-19; **Record Level:** collectionID: Roma3_5.8

##### Distribution

Introduced species from North and Central America (Nentwig et al. 2024).

#### Erigone dentipalpis

(Wider, 1834)

DE361090-87FD-57AE-9958-84DBDB8D322C

##### Materials

- **Type status:** Other material. **Occurrence:** recordedBy: Fattorini S., Di Giulio A.; individualCount: 1; sex: male; lifeStage: adult; occurrenceID: 3BF5660B-695F-5E5A-BB25-AC6502072803; **Taxon:** scientificName: Erigonedentipalpis (Wider, 1834); order: Araneae; family: Linyphiidae; genus: Erigone; **Location:** country: Italy; countryCode: IT; stateProvince: Rome; county: Rome; municipality: Rome; locality: Appia Antica Regional Park, Rome; locationRemarks: Caffarella Sud 2; decimalLatitude: 41.856742; decimalLongitude: 12.529453; geodeticDatum: WGS84; **Identification:** identifiedBy: Tommaso Fusco; dateIdentified: 2022; **Event:** samplingProtocol: Pitfall traps; eventDate: 2014-06-06; **Record Level:** collectionID: Roma3_5.8
- **Type status:** Other material. **Occurrence:** recordedBy: Fattorini S., Di Giulio A.; individualCount: 1; sex: female; lifeStage: adult; occurrenceID: DC64D0A5-8C20-5F89-A6B7-A257304D1B75; **Taxon:** scientificName: Erigonedentipalpis (Wider, 1834); order: Araneae; family: Linyphiidae; genus: Erigone; **Location:** country: Italy; countryCode: IT; stateProvince: Rome; county: Rome; municipality: Rome; locality: Appia Antica Regional Park, Rome; locationRemarks: Tor Marancia; decimalLatitude: 41.850308; decimalLongitude: 12.503178; geodeticDatum: WGS84; **Identification:** identifiedBy: Tommaso Fusco; dateIdentified: 2022; **Event:** samplingProtocol: Pitfall traps; eventDate: 2013-11-27; **Record Level:** collectionID: Roma3_5.8
- **Type status:** Other material. **Occurrence:** recordedBy: Fattorini S., Di Giulio A.; individualCount: 1; sex: female; lifeStage: adult; occurrenceID: C81CD207-6E71-5794-AAB7-0ABD6910CEC8; **Taxon:** scientificName: Erigonedentipalpis (Wider, 1834); order: Araneae; family: Linyphiidae; genus: Erigone; **Location:** country: Italy; countryCode: IT; stateProvince: Rome; county: Rome; municipality: Rome; locality: Appia Antica Regional Park, Rome; locationRemarks: Casal Verbeni; decimalLatitude: 41.815250; decimalLongitude: 12.552222; geodeticDatum: WGS84; **Identification:** identifiedBy: Tommaso Fusco; dateIdentified: 2022; **Event:** samplingProtocol: Pitfall traps; eventDate: 2014-06-05; **Record Level:** collectionID: Roma3_5.8
- **Type status:** Other material. **Occurrence:** recordedBy: Fattorini S., Di Giulio A.; individualCount: 1; sex: female; lifeStage: adult; occurrenceID: C762D6CB-A771-5AEE-AA70-FEF528C185FB; **Taxon:** scientificName: Erigonedentipalpis (Wider, 1834); order: Araneae; family: Linyphiidae; genus: Erigone; **Location:** country: Italy; countryCode: IT; stateProvince: Rome; county: Rome; municipality: Rome; locality: Appia Antica Regional Park, Rome; locationRemarks: San Sebastiano; decimalLatitude: 41.855733; decimalLongitude: 12.515114; geodeticDatum: WGS84; **Identification:** identifiedBy: Tommaso Fusco; dateIdentified: 2022; **Event:** samplingProtocol: Pitfall traps; eventDate: 2014-05-28; **Record Level:** collectionID: Roma3_5.8
- **Type status:** Other material. **Occurrence:** recordedBy: Fattorini S., Di Giulio A.; individualCount: 2; sex: 1 male, 1 female; lifeStage: adult; occurrenceID: 9E149BD1-882A-548C-AF43-F85B5C427632; **Taxon:** scientificName: Erigonedentipalpis (Wider, 1834); order: Araneae; family: Linyphiidae; genus: Erigone; **Location:** country: Italy; countryCode: IT; stateProvince: Rome; county: Rome; municipality: Rome; locality: Appia Antica Regional Park, Rome; locationRemarks: Acqua santa; decimalLatitude: 41.850561; decimalLongitude: 12.530861; geodeticDatum: WGS84; **Identification:** identifiedBy: Tommaso Fusco; dateIdentified: 2022; **Event:** samplingProtocol: Pitfall traps; eventDate: 2014-06-19; **Record Level:** collectionID: Roma3_5.8
- **Type status:** Other material. **Occurrence:** recordedBy: Fattorini S., Di Giulio A.; individualCount: 1; sex: male; lifeStage: adult; occurrenceID: 68A8FE61-223D-559F-935E-5ADFF2C9196F; **Taxon:** scientificName: Erigonedentipalpis (Wider, 1834); order: Araneae; family: Linyphiidae; genus: Erigone; **Location:** country: Italy; countryCode: IT; stateProvince: Rome; county: Rome; municipality: Rome; locality: Appia Antica Regional Park, Rome; locationRemarks: Caffarella Sud 2; decimalLatitude: 41.856742; decimalLongitude: 12.529453; geodeticDatum: WGS84; **Identification:** identifiedBy: Tommaso Fusco; dateIdentified: 2022; **Event:** samplingProtocol: Pitfall traps; eventDate: 2014-06-18; **Record Level:** collectionID: Roma3_5.8

##### Distribution

Europe, North Africa, Turkey, Caucasus, Russia (Europe to Far East), Kazakhstan, Iran to China. Introduced to Canada. Palaearctic (PAL) chorotype.

#### Gonatium biimpressum

Simon, 1884

86986D4C-FF82-55F4-84E6-A21F63ED4C3B

##### Materials

- **Type status:** Other material. **Occurrence:** recordedBy: Fattorini S., Di Giulio A.; individualCount: 1; sex: male; lifeStage: adult; occurrenceID: 885A79C3-FE1D-5B06-9EEB-3D447D41A81D; **Taxon:** scientificName: Gonatiumbiimpressum Simon, 1884; order: Araneae; family: Linyphiidae; genus: Gonatium; **Location:** country: Italy; countryCode: IT; stateProvince: Rome; county: Rome; municipality: Rome; locality: Appia Antica Regional Park, Rome; locationRemarks: Acqua Santa; decimalLatitude: 41.850561; decimalLongitude: 12.530861; geodeticDatum: WGS84; **Identification:** identifiedBy: Tommaso Fusco; dateIdentified: 2022; **Event:** samplingProtocol: Pitfall traps; eventDate: 2014-06-19; **Record Level:** collectionID: Roma3_5.8

##### Distribution

Only found in Corsica, Sardinia and mainland Italy. W-Mediterranean (WME) chorotype.

#### Mecopisthes latinus

Millidge, 1978

A30C9D61-1F48-57F6-A13D-88BD52A0CE52

##### Materials

- **Type status:** Other material. **Occurrence:** recordedBy: Fattorini S., Di Giulio A.; individualCount: 1; sex: male; lifeStage: adult; occurrenceID: BC998F16-4EDC-521E-AB99-9596C53E0A67; **Taxon:** scientificName: Mecopistheslatinus Millidge, 1978; order: Araneae; family: Linyphiidae; genus: Mecopisthes; **Location:** country: Italy; countryCode: IT; stateProvince: Rome; county: Rome; municipality: Rome; locality: Appia Antica Regional Park, Rome; locationRemarks: Caffarella Nord; decimalLatitude: 41.867753; decimalLongitude: 12.512414; geodeticDatum: WGS84; **Identification:** identifiedBy: Tommaso Fusco; dateIdentified: 2022; **Event:** samplingProtocol: Pitfall; eventDate: 2013-10-31; **Record Level:** collectionID: Roma3_5.8
- **Type status:** Other material. **Occurrence:** recordedBy: Fattorini S., Di Giulio A.; individualCount: 1; sex: male; lifeStage: adult; occurrenceID: DE832FCA-A8D2-5E8B-873C-58C47880FC9B; **Taxon:** scientificName: Mecopistheslatinus Millidge, 1978; order: Araneae; family: Linyphiidae; genus: Mecopisthes; **Location:** country: Italy; countryCode: IT; stateProvince: Rome; county: Rome; municipality: Rome; locality: Appia Antica Regional Park, Rome; locationRemarks: Caffarella Nord; decimalLatitude: 41.867753; decimalLongitude: 12.512414; geodeticDatum: WGS84; **Identification:** identifiedBy: Tommaso Fusco; dateIdentified: 2022; **Event:** samplingProtocol: Pitfall; eventDate: 2013-11-29; **Record Level:** collectionID: Roma3_5.8
- **Type status:** Other material. **Occurrence:** recordedBy: Fattorini S., Di Giulio A.; individualCount: 1; sex: male; lifeStage: adult; occurrenceID: 6832266E-31B7-5F76-B0EE-5DE66212BFFE; **Taxon:** scientificName: Mecopistheslatinus Millidge, 1978; order: Araneae; family: Linyphiidae; genus: Mecopisthes; **Location:** country: Italy; countryCode: IT; stateProvince: Rome; county: Rome; municipality: Rome; locality: Appia Antica Regional Park, Rome; locationRemarks: Farnesiana; decimalLatitude: 41.839667; decimalLongitude: 12.525528; geodeticDatum: WGS84; **Identification:** identifiedBy: Tommaso Fusco; dateIdentified: 2022; **Event:** samplingProtocol: Pitfall; eventDate: 2013-11-25; **Record Level:** collectionID: Roma3_5.8
- **Type status:** Other material. **Occurrence:** recordedBy: Fattorini S., Di Giulio A.; individualCount: 1; sex: female; lifeStage: adult; occurrenceID: 1628F6AB-1319-5EA8-870F-387CCC53AE5F; **Taxon:** scientificName: Mecopistheslatinus Millidge, 1978; order: Araneae; family: Linyphiidae; genus: Mecopisthes; **Location:** country: Italy; countryCode: IT; stateProvince: Rome; county: Rome; municipality: Rome; locality: Appia Antica Regional Park, Rome; locationRemarks: Tor Marancia; decimalLatitude: 41.850308; decimalLongitude: 12.503178; geodeticDatum: WGS84; **Identification:** identifiedBy: Tommaso Fusco; dateIdentified: 2022; **Event:** samplingProtocol: Pitfall; eventDate: 2014-05-26; **Record Level:** collectionID: Roma3_5.8
- **Type status:** Other material. **Occurrence:** recordedBy: Fattorini S., Di Giulio A.; individualCount: 1; sex: female; lifeStage: adult; occurrenceID: CC6AA190-29DB-5A25-B46D-C3772CB0DEE4; **Taxon:** scientificName: Mecopistheslatinus Millidge, 1978; order: Araneae; family: Linyphiidae; genus: Mecopisthes; **Location:** country: Italy; countryCode: IT; stateProvince: Rome; county: Rome; municipality: Rome; locality: Appia Antica Regional Park, Rome; locationRemarks: Caffarella Nord; decimalLatitude: 41.867753; decimalLongitude: 12.512414; geodeticDatum: WGS84; **Identification:** identifiedBy: Tommaso Fusco; dateIdentified: 2022; **Event:** samplingProtocol: Pitfall; eventDate: 2014-06-06; **Record Level:** collectionID: Roma3_5.8

##### Distribution

Only found in southern Switzerland, in northern and central Italy. S-European (SEU) chorotype.

#### Microctenonyx subitaneus

(O. Pickard-Cambridge, 1875)

7A5BB375-3DA3-551A-9B2F-C6B70A90DECE

##### Materials

- **Type status:** Other material. **Occurrence:** recordedBy: Fattorini S., Di Giulio A.; individualCount: 3; sex: male; lifeStage: adult; occurrenceID: 40F7EA54-5E7E-5695-837A-AB50347C58DF; **Taxon:** scientificName: Microctenonyxsubitaneus (O. Pickard-Cambridge, 1875); order: Araneae; family: Linyphiidae; genus: Microctenonyx; **Location:** country: Italy; countryCode: IT; stateProvince: Rome; county: Rome; municipality: Rome; locality: Appia Antica Regional Park, Rome; locationRemarks: Appia Antica; decimalLatitude: 41.812575; decimalLongitude: 12.564011; geodeticDatum: WGS84; **Identification:** identifiedBy: Tommaso Fusco; dateIdentified: 2022; **Event:** samplingProtocol: Pitfall traps; eventDate: 2014-06-13; **Record Level:** collectionID: Roma3_5.8
- **Type status:** Other material. **Occurrence:** recordedBy: Fattorini S., Di Giulio A.; individualCount: 1; sex: male; lifeStage: adult; occurrenceID: B0B5D85D-CFEB-5A19-ADC3-ADB4E83EBFCA; **Taxon:** scientificName: Microctenonyxsubitaneus (O. Pickard-Cambridge, 1875); order: Araneae; family: Linyphiidae; genus: Microctenonyx; **Location:** country: Italy; countryCode: IT; stateProvince: Rome; county: Rome; municipality: Rome; locality: Appia Antica Regional Park, Rome; locationRemarks: Caffarella Centro; decimalLatitude: 41.864889; decimalLongitude: 12.516389; geodeticDatum: WGS84; **Identification:** identifiedBy: Tommaso Fusco; dateIdentified: 2022; **Event:** samplingProtocol: Pitfall traps; eventDate: 2014-05-27; **Record Level:** collectionID: Roma3_5.8
- **Type status:** Other material. **Occurrence:** recordedBy: Fattorini S., Di Giulio A.; individualCount: 1; sex: male; lifeStage: adult; occurrenceID: DB0A26C8-C3AD-5CC1-9EFA-DED0112FDBA5; **Taxon:** scientificName: Microctenonyxsubitaneus (O. Pickard-Cambridge, 1875); order: Araneae; family: Linyphiidae; genus: Microctenonyx; **Location:** country: Italy; countryCode: IT; stateProvince: Rome; county: Rome; municipality: Rome; locality: Appia Antica Regional Park, Rome; locationRemarks: Caffarella Sud 1; decimalLatitude: 41.857247; decimalLongitude: 12.529211; geodeticDatum: WGS84; **Identification:** identifiedBy: Tommaso Fusco; dateIdentified: 2022; **Event:** samplingProtocol: Pitfall traps; eventDate: 2014-06-18; **Record Level:** collectionID: Roma3_5.8
- **Type status:** Other material. **Occurrence:** recordedBy: Fattorini S., Di Giulio A.; individualCount: 1; sex: female; lifeStage: adult; occurrenceID: 192BBA0F-7C61-53E0-9B02-E66639AC79AF; **Taxon:** scientificName: Microctenonyxsubitaneus (O. Pickard-Cambridge, 1875); order: Araneae; family: Linyphiidae; genus: Microctenonyx; **Location:** countryCode: IT; stateProvince: Rome; county: Rome; municipality: Rome; locality: Appia Antica Regional Park, Rome; locationRemarks: Casal Verbeni; decimalLatitude: 41.815250; decimalLongitude: 12.552222; geodeticDatum: WGS84; **Identification:** identifiedBy: Tommaso Fusco; dateIdentified: 2022; **Event:** samplingProtocol: Pitfall traps; eventDate: 2013-11-18; **Record Level:** collectionID: Roma3_5.8
- **Type status:** Other material. **Occurrence:** recordedBy: Fattorini S., Di Giulio A.; individualCount: 2; sex: 1 male, 1 female; lifeStage: adult; occurrenceID: 55BEA564-5F02-51D0-843C-EEC0F36CF7A1; **Taxon:** scientificName: Microctenonyxsubitaneus (O. Pickard-Cambridge, 1875); order: Araneae; family: Linyphiidae; genus: Microctenonyx; **Location:** countryCode: IT; stateProvince: Rome; county: Rome; municipality: Rome; locality: Appia Antica Regional Park, Rome; locationRemarks: Casal Verbeni; decimalLatitude: 41.815250; decimalLongitude: 12.552222; geodeticDatum: WGS84; **Identification:** identifiedBy: Tommaso Fusco; dateIdentified: 2022; **Event:** samplingProtocol: Pitfall traps; eventDate: 2013-12-06; **Record Level:** collectionID: Roma3_5.8
- **Type status:** Other material. **Occurrence:** recordedBy: Fattorini S., Di Giulio A.; individualCount: 1; sex: male; lifeStage: adult; occurrenceID: 54748125-E83B-594F-8F73-E0AC38ABCF35; **Taxon:** scientificName: Microctenonyxsubitaneus (O. Pickard-Cambridge, 1875); order: Araneae; family: Linyphiidae; genus: Microctenonyx; **Location:** countryCode: IT; stateProvince: Rome; county: Rome; municipality: Rome; locality: Appia Antica Regional Park, Rome; locationRemarks: Farnesiana; decimalLatitude: 41.839667; decimalLongitude: 12.525528; geodeticDatum: WGS84; **Identification:** identifiedBy: Tommaso Fusco; dateIdentified: 2022; **Event:** samplingProtocol: Pitfall traps; eventDate: 2013-11-13; **Record Level:** collectionID: Roma3_5.8
- **Type status:** Other material. **Occurrence:** recordedBy: Fattorini S., Di Giulio A.; individualCount: 1; sex: male; lifeStage: adult; occurrenceID: 00456B43-E5FD-58F0-83B7-42B6D2331C56; **Taxon:** scientificName: Microctenonyxsubitaneus (O. Pickard-Cambridge, 1875); order: Araneae; family: Linyphiidae; genus: Microctenonyx; **Location:** countryCode: IT; stateProvince: Rome; county: Rome; municipality: Rome; locality: Appia Antica Regional Park, Rome; locationRemarks: San Sebastiano; decimalLatitude: 41.855733; decimalLongitude: 12.515114; geodeticDatum: WGS84; **Identification:** identifiedBy: Tommaso Fusco; dateIdentified: 2022; **Event:** samplingProtocol: Pitfall traps; eventDate: 2013-11-12; **Record Level:** collectionID: Roma3_5.8
- **Type status:** Other material. **Occurrence:** recordedBy: Fattorini S., Di Giulio A.; individualCount: 1; sex: female; lifeStage: adult; occurrenceID: 4CCFFF14-2A19-567F-99E8-C39FBDA8F180; **Taxon:** scientificName: Microctenonyxsubitaneus (O. Pickard-Cambridge, 1875); order: Araneae; family: Linyphiidae; genus: Microctenonyx; **Location:** countryCode: IT; stateProvince: Rome; county: Rome; municipality: Rome; locality: Appia Antica Regional Park, Rome; locationRemarks: San Sebastiano; decimalLatitude: 41.855733; decimalLongitude: 12.515114; geodeticDatum: WGS84; **Identification:** identifiedBy: Tommaso Fusco; dateIdentified: 2022; **Event:** samplingProtocol: Pitfall traps; eventDate: 2013-11-25; **Record Level:** collectionID: Roma3_5.8
- **Type status:** Other material. **Occurrence:** recordedBy: Fattorini S., Di Giulio A.; individualCount: 1; sex: male; lifeStage: adult; occurrenceID: D8AAF807-C3DB-5697-A1BF-C4657F8F3BA0; **Taxon:** scientificName: Microctenonyxsubitaneus (O. Pickard-Cambridge, 1875); order: Araneae; family: Linyphiidae; genus: Microctenonyx; **Location:** countryCode: IT; stateProvince: Rome; county: Rome; municipality: Rome; locality: Appia Antica Regional Park, Rome; locationRemarks: Tor Marancia; decimalLatitude: 41.850308; decimalLongitude: 12.503178; geodeticDatum: WGS84; **Identification:** identifiedBy: Tommaso Fusco; dateIdentified: 2022; **Event:** samplingProtocol: Pitfall traps; eventDate: 2013-11-07; **Record Level:** collectionID: Roma3_5.8
- **Type status:** Other material. **Occurrence:** recordedBy: Fattorini S., Di Giulio A.; individualCount: 1; sex: female; lifeStage: adult; occurrenceID: 1BE692C5-0915-5CD4-8C8F-8BCDE4F68A93; **Taxon:** scientificName: Microctenonyxsubitaneus (O. Pickard-Cambridge, 1875); order: Araneae; family: Linyphiidae; genus: Microctenonyx; **Location:** countryCode: IT; stateProvince: Rome; county: Rome; municipality: Rome; locality: Appia Antica Regional Park, Rome; locationRemarks: Torre Selce; decimalLatitude: 41.816611; decimalLongitude: 12.560667; geodeticDatum: WGS84; **Identification:** identifiedBy: Tommaso Fusco; dateIdentified: 2022; **Event:** samplingProtocol: Pitfall traps; eventDate: 2013-11-18; **Record Level:** collectionID: Roma3_5.8
- **Type status:** Other material. **Occurrence:** recordedBy: Fattorini S., Di Giulio A.; individualCount: 3; sex: 2 male, 1 female; lifeStage: adult; occurrenceID: DF40F813-BB65-586A-87EB-F76A4FAF1B74; **Taxon:** scientificName: Microctenonyxsubitaneus (O. Pickard-Cambridge, 1875); order: Araneae; family: Linyphiidae; genus: Microctenonyx; **Location:** countryCode: IT; stateProvince: Rome; county: Rome; municipality: Rome; locality: Appia Antica Regional Park, Rome; locationRemarks: Torre Selce; decimalLatitude: 41.816611; decimalLongitude: 12.560667; geodeticDatum: WGS84; **Identification:** identifiedBy: Tommaso Fusco; dateIdentified: 2022; **Event:** samplingProtocol: Pitfall traps; eventDate: 2013-11-27; **Record Level:** collectionID: Roma3_5.8

##### Distribution

Europe, Macaronesia, North Africa to Kyrgyzstan. Introduced to USA, Chile, Argentina, Kenya, South Africa, Australia, New Zealand. Cosmopolitan (COS) chorotype.

#### Microneta viaria

(Blackwall, 1841)

F9A0D095-B983-5B28-A737-73A6DEE5CD61

##### Materials

- **Type status:** Other material. **Occurrence:** recordedBy: Fattorini S., Di Giulio A.; individualCount: 1; sex: female; lifeStage: adult; occurrenceID: 036D68B4-95FE-56CB-9555-EE2CAD385C68; **Taxon:** scientificName: Micronetaviaria (Blackwall, 1841); order: Araneae; family: Linyphiidae; genus: Microneta; **Location:** country: Italy; countryCode: IT; stateProvince: Rome; county: Rome; municipality: Rome; locality: Appia Antica Regional Park, Rome; locationRemarks: Tor Marancia; decimalLatitude: 41.850308; decimalLongitude: 12.503178; geodeticDatum: WGS84; **Identification:** identifiedBy: Tommaso Fusco; dateIdentified: 2022; **Event:** samplingProtocol: Pitfall traps; eventDate: 2014-05-26; **Record Level:** collectionID: Roma3_5.8

##### Distribution

North America, Europe, Turkey, North Africa, Caucasus, Russia (Europe to Far East), Kazakhstan, Iran, Kyrgyzstan, China, Mongolia, Korea, Japan. Holarctic (OLA) chorotype.

#### Oedothorax paludigena

Simon, 1926

B2BC1687-26F1-525E-8FB4-4F40466C51CD

##### Materials

- **Type status:** Other material. **Occurrence:** recordedBy: Fattorini S., Di Giulio A.; individualCount: 1; sex: male; lifeStage: adult; occurrenceID: A55EADD7-9ED5-5A58-B593-8734BCB48984; **Taxon:** scientificName: Oedothoraxpaludigena Simon, 1926; order: Araneae; family: Linyphiidae; genus: Oedothorax; **Location:** country: Italy; countryCode: IT; stateProvince: Rome; county: Rome; municipality: Rome; locality: Appia Antica Regional Park, Rome; locationRemarks: Cava Fiorucci; decimalLatitude: 41.834106; decimalLongitude: 12.549264; geodeticDatum: WGS84; **Identification:** identifiedBy: Tommaso Fusco; dateIdentified: 2022; **Event:** samplingProtocol: Pitfall traps; eventDate: 2014-06-13; **Record Level:** collectionID: Roma3_5.8

##### Distribution

Spain, France (including Corsica), Italy (including Sardinia), Albania, Greece. Mediterranean (MED) chorotype.

#### Ostearius melanopygius

(O. Pickard-Cambridge, 1880)

C2ACB1B6-06FC-58BC-A9A4-5DBCF97712DE

##### Materials

- **Type status:** Other material. **Occurrence:** recordedBy: Fattorini S., Di Giulio A.; individualCount: 1; sex: female; lifeStage: adult; occurrenceID: 2AB19325-EBFE-56FD-AD8C-C06E54135389; **Taxon:** scientificName: Osteariusmelanopygius (O. Pickard-Cambridge, 1880); order: Araneae; family: Linyphiidae; genus: Ostearius; **Location:** country: Italy; countryCode: IT; stateProvince: Rome; county: Rome; municipality: Rome; locality: Appia Antica Regional Park, Rome; locationRemarks: Casal Verbeni; decimalLatitude: 41.815250; decimalLongitude: 12.552222; geodeticDatum: WGS84; **Identification:** identifiedBy: Tommaso Fusco; dateIdentified: 2022; **Event:** samplingProtocol: Pitfall traps; eventDate: 2013-12-06; **Record Level:** collectionID: Roma3_5.8

##### Distribution

Cosmopolitan. Cosmopolitan (COS) chorotype.

##### Notes

Species of South American origin that has established in Europe (Nentwig et al. 2024).

#### Ouedia rufithorax

(Simon, 1881)

8526B047-8176-5A64-A954-F8B60550B802

##### Materials

- **Type status:** Other material. **Occurrence:** recordedBy: Fattorini S., Di Giulio A.; individualCount: 1; sex: male; lifeStage: adult; occurrenceID: A0D3A3D7-0A0F-5781-89D4-CD8439455BDF; **Taxon:** scientificName: Ouediarufithorax (Simon, 1881); order: Araneae; family: Linyphiidae; genus: Ouedia; **Location:** country: Italy; countryCode: IT; stateProvince: Rome; county: Rome; municipality: Rome; locality: Appia Antica Regional Park, Rome; locationRemarks: Casal Verbeni; decimalLatitude: 41.815250; decimalLongitude: 12.552222; geodeticDatum: WGS84; **Identification:** identifiedBy: Tommaso Fusco; dateIdentified: 2022; **Event:** samplingProtocol: Pitfall traps; eventDate: 2013-11-27; **Record Level:** collectionID: Roma3_5.8
- **Type status:** Other material. **Occurrence:** recordedBy: Fattorini S., Di Giulio A.; individualCount: 1; sex: female; lifeStage: adult; occurrenceID: F00B3023-109D-5B28-A2FE-21A72A5D95E2; **Taxon:** scientificName: Ouediarufithorax (Simon, 1881); order: Araneae; family: Linyphiidae; genus: Ouedia; **Location:** country: Italy; countryCode: IT; stateProvince: Rome; county: Rome; municipality: Rome; locality: Appia Antica Regional Park, Rome; locationRemarks: Caffarella Sud; decimalLatitude: 41.856742; decimalLongitude: 12.529453; geodeticDatum: WGS84; **Identification:** identifiedBy: Tommaso Fusco; dateIdentified: 2022; **Event:** samplingProtocol: Pitfall traps; eventDate: 2013-11-20; **Record Level:** collectionID: Roma3_5.8
- **Type status:** Other material. **Occurrence:** recordedBy: Fattorini S., Di Giulio A.; individualCount: 1; sex: male; lifeStage: adult; occurrenceID: 5728F98D-D3C2-5FB9-89BE-2C0629116AA0; **Taxon:** scientificName: Ouediarufithorax (Simon, 1881); order: Araneae; family: Linyphiidae; genus: Ouedia; **Location:** country: Italy; countryCode: IT; stateProvince: Rome; county: Rome; municipality: Rome; locality: Appia Antica Regional Park, Rome; locationRemarks: Casal Verbeni; decimalLatitude: 41.815250; decimalLongitude: 12.552222; geodeticDatum: WGS84; **Identification:** identifiedBy: Tommaso Fusco; dateIdentified: 2022; **Event:** samplingProtocol: Pitfall traps; eventDate: 2013-11-07; **Record Level:** collectionID: Roma3_5.8

##### Distribution

Portugal, Spain, France, Italy, Algeria, Tunisia. W-Mediterranean (WME) chorotype.

#### Palliduphantes arenicola

(Denis, 1964)

B37754A7-9D24-5DA4-8358-2136F46CF39F

##### Materials

- **Type status:** Other material. **Occurrence:** recordedBy: Fattorini S., Di Giulio A.; individualCount: 1; sex: female; lifeStage: adult; occurrenceID: 116A9BD3-5CA6-591D-A2A6-6D70A0D559FC; **Taxon:** scientificName: Palliduphantesarenicola (Denis, 1964); order: Araneae; family: Linyphiidae; genus: Palliduphantes; **Location:** country: Italy; countryCode: IT; stateProvince: Rome; county: Rome; municipality: Rome; locality: Appia Antica Regional Park, Rome; locationRemarks: Casal Verbeni; decimalLatitude: 41.815250; decimalLongitude: 12.552222; geodeticDatum: WGS84; **Identification:** identifiedBy: Tommaso Fusco; dateIdentified: 2022; **Event:** samplingProtocol: Pitfall traps; eventDate: 2013-11-27; **Record Level:** collectionID: Roma3_5.8
- **Type status:** Other material. **Occurrence:** recordedBy: Fattorini S., Di Giulio A.; individualCount: 1; sex: female; lifeStage: adult; occurrenceID: 4543D6F7-9542-516A-B204-6CABE152282A; **Taxon:** scientificName: Palliduphantesarenicola (Denis, 1964); order: Araneae; family: Linyphiidae; genus: Palliduphantes; **Location:** country: Italy; countryCode: IT; stateProvince: Rome; county: Rome; municipality: Rome; locality: Appia Antica Regional Park, Rome; locationRemarks: Farnesiana; decimalLatitude: 41.839667; decimalLongitude: 12.525528; geodeticDatum: WGS84; **Identification:** identifiedBy: Tommaso Fusco; dateIdentified: 2022; **Event:** samplingProtocol: Pitfall traps; eventDate: 2013-11-13; **Record Level:** collectionID: Roma3_5.8
- **Type status:** Other material. **Occurrence:** recordedBy: Fattorini S., Di Giulio A.; individualCount: 2; sex: male; lifeStage: adult; occurrenceID: F551358C-9177-584A-8047-3AD765F5CCEE; **Taxon:** scientificName: Palliduphantesarenicola (Denis, 1964); order: Araneae; family: Linyphiidae; genus: Palliduphantes; **Location:** country: Italy; countryCode: IT; stateProvince: Rome; county: Rome; municipality: Rome; locality: Appia Antica Regional Park, Rome; locationRemarks: Farnesiana; decimalLatitude: 41.839667; decimalLongitude: 12.525528; geodeticDatum: WGS84; **Identification:** identifiedBy: Tommaso Fusco; dateIdentified: 2022; **Event:** samplingProtocol: Pitfall traps; eventDate: 2013-12-02; **Record Level:** collectionID: Roma3_5.8
- **Type status:** Other material. **Occurrence:** recordedBy: Fattorini S., Di Giulio A.; individualCount: 1; sex: male; lifeStage: adult; occurrenceID: D9E35F5C-71CC-5A9F-8E31-558B62ADB551; **Taxon:** scientificName: Palliduphantesarenicola (Denis, 1964); order: Araneae; family: Linyphiidae; genus: Palliduphantes; **Location:** country: Italy; countryCode: IT; stateProvince: Rome; county: Rome; municipality: Rome; locality: Appia Antica Regional Park, Rome; locationRemarks: Casal Verbeni; decimalLatitude: 41.815250; decimalLongitude: 12.552222; geodeticDatum: WGS84; **Identification:** identifiedBy: Tommaso Fusco; dateIdentified: 2022; **Event:** samplingProtocol: Pitfall traps; eventDate: 2013-11-07; **Record Level:** collectionID: Roma3_5.8

##### Distribution

France (Denis 1964), Switzerland (Pozzi and Hänggi 1998), Italy. S-European (SEU) chorotype.

##### Notes

New record for Italy

#### Palliduphantes byzantinus

(Fage, 1931)

F76D4339-3704-5C55-B860-110355CD1CE8

##### Materials

- **Type status:** Other material. **Occurrence:** recordedBy: Fattorini S., Di Giulio A.; individualCount: 1; sex: male; lifeStage: adult; occurrenceID: A34248CF-CCAE-5D14-83CC-593A4D129D7C; **Taxon:** scientificName: Palliduphantesbyzantinus (Fage, 1931); order: Araneae; family: Linyphiidae; genus: Palliduphantes; **Location:** country: Italy; countryCode: IT; stateProvince: Rome; county: Rome; municipality: Rome; locality: Appia Antica Regional Park, Rome; locationRemarks: Appia Antica; decimalLatitude: 41.812575; decimalLongitude: 12.564011; geodeticDatum: WGS84; **Identification:** identifiedBy: Tommaso Fusco; dateIdentified: 2022; **Event:** samplingProtocol: Pitfall traps; eventDate: 2014-05-26; **Record Level:** collectionID: Roma3_5.8
- **Type status:** Other material. **Occurrence:** recordedBy: Fattorini S., Di Giulio A.; individualCount: 1; sex: male; lifeStage: adult; occurrenceID: 775B4BF3-33DD-5462-9054-401CAC48BBC3; **Taxon:** scientificName: Palliduphantesbyzantinus (Fage, 1931); order: Araneae; family: Linyphiidae; genus: Palliduphantes; **Location:** country: Italy; countryCode: IT; stateProvince: Rome; county: Rome; municipality: Rome; locality: Appia Antica Regional Park, Rome; locationRemarks: Torre Selce; decimalLatitude: 41.816611; decimalLongitude: 12.560667; geodeticDatum: WGS84; **Identification:** identifiedBy: Tommaso Fusco; dateIdentified: 2022; **Event:** samplingProtocol: Pitfall traps; eventDate: 2014-05-26; **Record Level:** collectionID: Roma3_5.8
- **Type status:** Other material. **Occurrence:** recordedBy: Fattorini S., Di Giulio A.; individualCount: 2; sex: male; lifeStage: adult; occurrenceID: 92B322EC-0A59-5C77-8679-5861C3A8946A; **Taxon:** scientificName: Palliduphantesbyzantinus (Fage, 1931); order: Araneae; family: Linyphiidae; genus: Palliduphantes; **Location:** country: Italy; countryCode: IT; stateProvince: Rome; county: Rome; municipality: Rome; locality: Appia Antica Regional Park, Rome; locationRemarks: Farnesiana; decimalLatitude: 41.839667; decimalLongitude: 12.525528; geodeticDatum: WGS84; **Identification:** identifiedBy: Tommaso Fusco; dateIdentified: 2022; **Event:** samplingProtocol: Pitfall traps; eventDate: 2014-05-28; **Record Level:** collectionID: Roma3_5.8
- **Type status:** Other material. **Occurrence:** recordedBy: Fattorini S., Di Giulio A.; individualCount: 1; sex: male; lifeStage: adult; occurrenceID: CAEEC67F-2336-5F5B-8124-7B91CDA44261; **Taxon:** scientificName: Palliduphantesbyzantinus (Fage, 1931); order: Araneae; family: Linyphiidae; genus: Palliduphantes; **Location:** country: Italy; countryCode: IT; stateProvince: Rome; county: Rome; municipality: Rome; locality: Appia Antica Regional Park, Rome; locationRemarks: Farnesiana; decimalLatitude: 41.839667; decimalLongitude: 12.525528; geodeticDatum: WGS84; **Identification:** identifiedBy: Tommaso Fusco; dateIdentified: 2022; **Event:** samplingProtocol: Pitfall traps; eventDate: 2014-06-09; **Record Level:** collectionID: Roma3_5.8

##### Distribution

Italy, Romania, Bulgaria, North Macedonia, Greece, Turkey. S-European (SEU) chorotype.

#### Palliduphantes istrianus

(Kulczyński, 1914)

64397E20-382F-5B4B-A1C2-C5E6990AA09D

##### Materials

- **Type status:** Other material. **Occurrence:** recordedBy: Fattorini S., Di Giulio A.; individualCount: 1; sex: female; lifeStage: adult; occurrenceID: 3BE57980-1319-53E7-B177-4CE00887740D; **Taxon:** scientificName: Palliduphantesistrianus (Kulczyński, 1914); order: Araneae; family: Linyphiidae; genus: Palliduphantes; **Location:** country: Italy; countryCode: IT; stateProvince: Rome; county: Rome; municipality: Rome; locality: Appia Antica Regional Park, Rome; locationRemarks: Tor Marancia; decimalLatitude: 41.850308; decimalLongitude: 12.503178; geodeticDatum: WGS84; **Identification:** identifiedBy: Tommaso Fusco; dateIdentified: 2022; **Event:** samplingProtocol: Pitfall traps; eventDate: 2014-06-05; **Record Level:** collectionID: Roma3_5.8
- **Type status:** Other material. **Occurrence:** recordedBy: Fattorini S., Di Giulio A.; individualCount: 2; sex: male; lifeStage: adult; occurrenceID: 078C6694-3E1C-55E8-ADEF-DDD3CD1E53F8; **Taxon:** scientificName: Palliduphantesistrianus (Kulczyński, 1914); order: Araneae; family: Linyphiidae; genus: Palliduphantes; **Location:** country: Italy; countryCode: IT; stateProvince: Rome; county: Rome; municipality: Rome; locality: Appia Antica Regional Park, Rome; locationRemarks: Tor Marancia; decimalLatitude: 41.850308; decimalLongitude: 12.503178; geodeticDatum: WGS84; **Identification:** identifiedBy: Tommaso Fusco; dateIdentified: 2022; **Event:** samplingProtocol: Pitfall traps; eventDate: 2014-06-05; **Record Level:** collectionID: Roma3_5.8
- **Type status:** Other material. **Occurrence:** recordedBy: Fattorini S., Di Giulio A.; individualCount: 1; sex: male; lifeStage: adult; occurrenceID: 134FBBEB-38B3-559B-A883-2A5699CC6897; **Taxon:** scientificName: Palliduphantesistrianus (Kulczyński, 1914); order: Araneae; family: Linyphiidae; genus: Palliduphantes; **Location:** country: Italy; countryCode: IT; stateProvince: Rome; county: Rome; municipality: Rome; locality: Appia Antica Regional Park, Rome; locationRemarks: Tor Marancia; decimalLatitude: 41.850308; decimalLongitude: 12.503178; geodeticDatum: WGS84; **Identification:** identifiedBy: Tommaso Fusco; dateIdentified: 2022; **Event:** samplingProtocol: Pitfall traps; eventDate: 2014-05-15; **Record Level:** collectionID: Roma3_5.8
- **Type status:** Other material. **Occurrence:** recordedBy: Fattorini S., Di Giulio A.; individualCount: 1; sex: female; lifeStage: adult; occurrenceID: CF170022-4638-522D-AF3E-D33AB48025CC; **Taxon:** scientificName: Palliduphantesistrianus (Kulczyński, 1914); order: Araneae; family: Linyphiidae; genus: Palliduphantes; **Location:** country: Italy; countryCode: IT; stateProvince: Rome; county: Rome; municipality: Rome; locality: Appia Antica Regional Park, Rome; locationRemarks: Tor Marancia; decimalLatitude: 41.850308; decimalLongitude: 12.503178; geodeticDatum: WGS84; **Identification:** identifiedBy: Tommaso Fusco; dateIdentified: 2022; **Event:** samplingProtocol: Pitfall traps; eventDate: 2014-05-15; **Record Level:** collectionID: Roma3_5.8

##### Distribution

Italy, Balkans. S-European (SEU) chorotype.

#### Pelecopsis digitulus

Bosmans & Abrous, 1992

9BF2E451-A4E8-56DC-9B3E-9AD1B2C547A5

##### Materials

- **Type status:** Other material. **Occurrence:** recordedBy: Fattorini S., Di Giulio A.; individualCount: 1; sex: female; lifeStage: adult; occurrenceID: D6240784-F008-517C-8F3C-84500C0FA691; **Taxon:** scientificName: Pelecopsisdigitulus Bosmans & Abrous, 1992; order: Araneae; family: Linyphiidae; genus: Pelecopsis; **Location:** country: Italy; countryCode: IT; stateProvince: Rome; county: Rome; municipality: Rome; locality: Appia Antica Regional Park, Rome; locationRemarks: Caffarella Nord; decimalLatitude: 41.867753; decimalLongitude: 12.512414; geodeticDatum: WGS84; **Identification:** identifiedBy: Tommaso Fusco; dateIdentified: 2022; **Event:** samplingProtocol: Pitfall traps; eventDate: 2013-11-29; **Record Level:** collectionID: Roma3_5.8

##### Distribution

Algeria (Bosmans and Abrous 1992), Corsica (Lissner 2016), Italy. W-Mediterranean (WME) chorotype.

##### Notes

**Figure 8.**
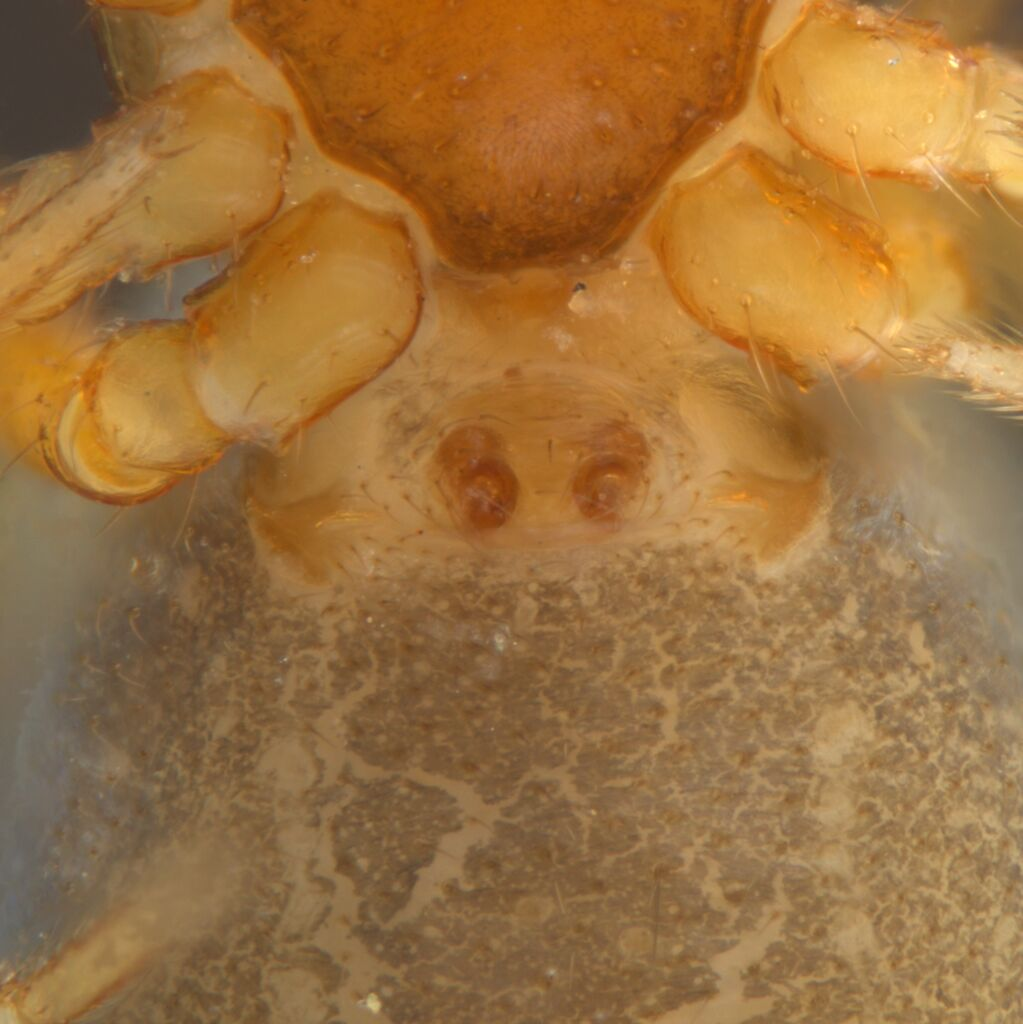
*Pelecopsisdigitulus*, epigyne. Specimen collected in a protected green space in urban Rome, Italy (the Appia Antica Regional Park).

New record for Italy (Figs 7, 8). Although only a single female of this species was found, the epigyne matches that of *E.dentigera* illustrated by Oger (2024) and described by Bosmans and Abrous (1992). The presence of this species in Italy is further confirmed by its occurrence in Umbria and Apulia (Umbria, Perugia, Castiglione del Lago, Isola Polvese 300 m alt., 8.V.2013, P. Salerno leg., 1 male; Apulia, Bari, Andria, Castel del Monte 500 m alt., 4.I-2.II.2005 R. Addante leg., 1 male; Museo Civico di Scienze Naturali "Enrico Caffi", Bergamo; Paolo Pantini, personal communication).

#### Prinerigone vagans

(Audouin, 1826)

F3D2909F-8317-5AB4-92CF-45766A53A1AA

##### Materials

- **Type status:** Other material. **Occurrence:** recordedBy: Fattorini S., Di Giulio A.; individualCount: 1; sex: male; lifeStage: adult; occurrenceID: B5BACF9C-359C-5E22-911C-5973528B4603; **Taxon:** scientificName: Prinerigonevagans (Audouin, 1826); order: Araneae; family: Linyphiidae; genus: Prinerigone; **Location:** country: Italy; countryCode: IT; stateProvince: Rome; county: Rome; municipality: Rome; locality: Appia Antica Regional Park, Rome; locationRemarks: Farnesiana; decimalLatitude: 41.839667; decimalLongitude: 12.525528; geodeticDatum: WGS84; **Identification:** identifiedBy: Tommaso Fusco; dateIdentified: 2022; **Event:** samplingProtocol: Pitfall traps; eventDate: 2013-11-25; **Record Level:** collectionID: Roma3_5.8

##### Distribution

Europe, North Africa, Turkey, Caucasus, Middle East, Iran, Central Asia, China. Palaearctic (PAL) chorotype.

#### Scutpelecopsis krausi

(Wunderlich, 1980)

32EEE86E-B20A-5AF8-8050-912E7CA341E4

##### Materials

- **Type status:** Other material. **Occurrence:** recordedBy: Fattorini S., Di Giulio A.; individualCount: 1; sex: male; lifeStage: adult; occurrenceID: 1A18BEA9-C2BA-598D-AE27-F61B3EE99802; **Taxon:** scientificName: Scutpelecopsiskrausi (Wunderlich, 1980); order: Araneae; family: Linyphiidae; genus: Scutpelecopsis; **Location:** country: Italy; countryCode: IT; stateProvince: Rome; county: Rome; municipality: Rome; locality: Appia Antica Regional Park, Rome; locationRemarks: Caffarella Sud; decimalLatitude: 41.857247; decimalLongitude: 12.529211; geodeticDatum: WGS84; **Identification:** identifiedBy: Tommaso Fusco; dateIdentified: 2022; **Event:** samplingProtocol: Pitfall traps; eventDate: 2013-11-20; **Record Level:** collectionID: Roma3_5.8
- **Type status:** Other material. **Occurrence:** recordedBy: Fattorini S., Di Giulio A.; individualCount: 4; sex: female; lifeStage: adult; occurrenceID: 160CD9C1-9109-528F-9F9A-364CA88E50D0; **Taxon:** scientificName: Scutpelecopsiskrausi (Wunderlich, 1980); order: Araneae; family: Linyphiidae; genus: Scutpelecopsis; **Location:** country: Italy; countryCode: IT; stateProvince: Rome; county: Rome; municipality: Rome; locality: Appia Antica Regional Park, Rome; locationRemarks: Caffarella Sud; decimalLatitude: 41.857247; decimalLongitude: 12.529211; geodeticDatum: WGS84; **Identification:** identifiedBy: Tommaso Fusco; dateIdentified: 2022; **Event:** samplingProtocol: Pitfall traps; eventDate: 2013-11-20; **Record Level:** collectionID: Roma3_5.8

##### Distribution

Italy, Balkans. S-European (SEU) chorotype.

#### Sintula retroversus

(O. Pickard-Cambridge, 1875)

13A6081F-3645-57CB-8E48-650EC61EE9C1

##### Materials

- **Type status:** Other material. **Occurrence:** recordedBy: Fattorini S., Di Giulio A.; individualCount: 1; sex: male; lifeStage: adult; occurrenceID: B8B28700-E018-514B-8EDD-3873A90E6C14; **Taxon:** scientificName: Sintularetroversus (O. Pickard-Cambridge, 1875); order: Araneae; family: Linyphiidae; genus: Sintula; **Location:** country: Italy; countryCode: IT; stateProvince: Rome; county: Rome; municipality: Rome; locality: Appia Antica Regional Park, Rome; locationRemarks: Caffarella Sud; decimalLatitude: 41.857247; decimalLongitude: 12.529211; geodeticDatum: WGS84; **Identification:** identifiedBy: Tommaso Fusco; dateIdentified: 2022; **Event:** samplingProtocol: Pitfall traps; eventDate: 2013-10-31; **Record Level:** collectionID: Roma3_5.8
- **Type status:** Other material. **Occurrence:** recordedBy: Fattorini S., Di Giulio A.; individualCount: 1; sex: male; lifeStage: adult; occurrenceID: 080C942F-445E-5FB0-94F5-D755DEE0AFE1; **Taxon:** scientificName: Sintularetroversus (O. Pickard-Cambridge, 1875); order: Araneae; family: Linyphiidae; genus: Sintula; **Location:** country: Italy; countryCode: IT; stateProvince: Rome; county: Rome; municipality: Rome; locality: Appia Antica Regional Park, Rome; locationRemarks: Casal Verbeni; decimalLatitude: 41.815250; decimalLongitude: 12.552222; geodeticDatum: WGS84; **Identification:** identifiedBy: Tommaso Fusco; dateIdentified: 2022; **Event:** samplingProtocol: Pitfall traps; eventDate: 2013-11-18; **Record Level:** collectionID: Roma3_5.8
- **Type status:** Other material. **Occurrence:** recordedBy: Fattorini S., Di Giulio A.; individualCount: 1; sex: male; lifeStage: adult; occurrenceID: 85656C64-9277-5DE4-A1F6-FD804537582C; **Taxon:** scientificName: Sintularetroversus (O. Pickard-Cambridge, 1875); order: Araneae; family: Linyphiidae; genus: Sintula; **Location:** country: Italy; countryCode: IT; stateProvince: Rome; county: Rome; municipality: Rome; locality: Appia Antica Regional Park, Rome; locationRemarks: Casal Verbeni; decimalLatitude: 41.815250; decimalLongitude: 12.552222; geodeticDatum: WGS84; **Identification:** identifiedBy: Tommaso Fusco; dateIdentified: 2022; **Event:** samplingProtocol: Pitfall traps; eventDate: 2013-12-06; **Record Level:** collectionID: Roma3_5.8
- **Type status:** Other material. **Occurrence:** recordedBy: Fattorini S., Di Giulio A.; individualCount: 3; sex: 2 male, 1 female; lifeStage: adult; occurrenceID: 9747F2A6-C1F0-564B-A835-C3536F6C8DE6; **Taxon:** scientificName: Sintularetroversus (O. Pickard-Cambridge, 1875); order: Araneae; family: Linyphiidae; genus: Sintula; **Location:** country: Italy; countryCode: IT; stateProvince: Rome; county: Rome; municipality: Rome; locality: Appia Antica Regional Park, Rome; locationRemarks: Cava Fiorucci; decimalLatitude: 41.834106; decimalLongitude: 12.549264; geodeticDatum: WGS84; **Identification:** identifiedBy: Tommaso Fusco; dateIdentified: 2022; **Event:** samplingProtocol: Pitfall traps; eventDate: 2013-11-18; **Record Level:** collectionID: Roma3_5.8
- **Type status:** Other material. **Occurrence:** recordedBy: Fattorini S., Di Giulio A.; individualCount: 1; sex: male; lifeStage: adult; occurrenceID: F3C29AC5-ED7F-5EBA-B7A4-4C74669F485C; **Taxon:** scientificName: Sintularetroversus (O. Pickard-Cambridge, 1875); order: Araneae; family: Linyphiidae; genus: Sintula; **Location:** country: Italy; countryCode: IT; stateProvince: Rome; county: Rome; municipality: Rome; locality: Appia Antica Regional Park, Rome; locationRemarks: Cava Fiorucci; decimalLatitude: 41.834106; decimalLongitude: 12.549264; geodeticDatum: WGS84; **Identification:** identifiedBy: Tommaso Fusco; dateIdentified: 2022; **Event:** samplingProtocol: Pitfall traps; eventDate: 2013-11-27; **Record Level:** collectionID: Roma3_5.8
- **Type status:** Other material. **Occurrence:** recordedBy: Fattorini S., Di Giulio A.; individualCount: 1; sex: male; lifeStage: adult; occurrenceID: F4E63534-797F-59D3-86A7-DE07B40A27CC; **Taxon:** scientificName: Sintularetroversus (O. Pickard-Cambridge, 1875); order: Araneae; family: Linyphiidae; genus: Sintula; **Location:** country: Italy; countryCode: IT; stateProvince: Rome; county: Rome; municipality: Rome; locality: Appia Antica Regional Park, Rome; locationRemarks: Cava Fiorucci; decimalLatitude: 41.834106; decimalLongitude: 12.549264; geodeticDatum: WGS84; **Identification:** identifiedBy: Tommaso Fusco; dateIdentified: 2022; **Event:** samplingProtocol: Pitfall traps; eventDate: 2013-12-06; **Record Level:** collectionID: Roma3_5.8
- **Type status:** Other material. **Occurrence:** recordedBy: Fattorini S., Di Giulio A.; individualCount: 2; sex: male; lifeStage: adult; occurrenceID: 7404C9A7-B74F-5472-9148-9F4FC619A28E; **Taxon:** scientificName: Sintularetroversus (O. Pickard-Cambridge, 1875); order: Araneae; family: Linyphiidae; genus: Sintula; **Location:** country: Italy; countryCode: IT; stateProvince: Rome; county: Rome; municipality: Rome; locality: Appia Antica Regional Park, Rome; locationRemarks: Farnesiana; decimalLatitude: 41.839667; decimalLongitude: 12.525528; geodeticDatum: WGS84; **Identification:** identifiedBy: Tommaso Fusco; dateIdentified: 2022; **Event:** samplingProtocol: Pitfall traps; eventDate: 2013-11-25; **Record Level:** collectionID: Roma3_5.8
- **Type status:** Other material. **Occurrence:** recordedBy: Fattorini S., Di Giulio A.; individualCount: 2; sex: male; lifeStage: adult; occurrenceID: 0642582A-7B47-5BBE-BE5B-B0D030FB949D; **Taxon:** scientificName: Sintularetroversus (O. Pickard-Cambridge, 1875); order: Araneae; family: Linyphiidae; genus: Sintula; **Location:** country: Italy; countryCode: IT; stateProvince: Rome; county: Rome; municipality: Rome; locality: Appia Antica Regional Park, Rome; locationRemarks: Farnesiana; decimalLatitude: 41.839667; decimalLongitude: 12.525528; geodeticDatum: WGS84; **Identification:** identifiedBy: Tommaso Fusco; dateIdentified: 2022; **Event:** samplingProtocol: Pitfall traps; eventDate: 2013-12-02; **Record Level:** collectionID: Roma3_5.8
- **Type status:** Other material. **Occurrence:** recordedBy: Fattorini S., Di Giulio A.; individualCount: 2; sex: male; lifeStage: adult; occurrenceID: C7741BFE-2815-5CEC-8CB6-5C54673A86A7; **Taxon:** scientificName: Sintularetroversus (O. Pickard-Cambridge, 1875); order: Araneae; family: Linyphiidae; genus: Sintula; **Location:** country: Italy; countryCode: IT; stateProvince: Rome; county: Rome; municipality: Rome; locality: Appia Antica Regional Park, Rome; locationRemarks: Torre Selce; decimalLatitude: 41.816611; decimalLongitude: 12.560667; geodeticDatum: WGS84; **Identification:** identifiedBy: Tommaso Fusco; dateIdentified: 2022; **Event:** samplingProtocol: Pitfall traps; eventDate: 2013-11-27; **Record Level:** collectionID: Roma3_5.8

##### Distribution

Europe, Turkey, Caucasus. European (EUR) chorotype.

#### Syedra nigrotibialis

Simon, 1884

010BD4E2-B82B-54F9-8A36-833718AE343E

##### Materials

- **Type status:** Other material. **Occurrence:** recordedBy: Fattorini S., Di Giulio A.; individualCount: 2; sex: 1 male, 1 female; lifeStage: adult; occurrenceID: E2FC32F9-E705-59AA-8486-2F3D8E205BE6; **Taxon:** scientificName: Syedranigrotibialis Simon, 1884; order: Araneae; family: Linyphiidae; genus: Syedra; **Location:** country: Italy; countryCode: IT; stateProvince: Rome; county: Rome; municipality: Rome; locality: Appia Antica Regional Park, Rome; locationRemarks: Tor Marancia; decimalLatitude: 41.850308; decimalLongitude: 12.503178; geodeticDatum: WGS84; **Identification:** identifiedBy: Tommaso Fusco; dateIdentified: 2022; **Event:** samplingProtocol: Pitfall traps; eventDate: 2014-06-05; **Record Level:** collectionID: Roma3_5.8

##### Distribution

Only found in Corsica, Sardinia and mainland Italy. W-Mediterranean (WME) chorotype.

#### Tenuiphantes herbicola

(Simon, 1884)

290E74C6-9763-56BF-AEB4-81F3A4217911

##### Materials

- **Type status:** Other material. **Occurrence:** recordedBy: Fattorini S., Di Giulio A.; individualCount: 1; sex: male; lifeStage: adult; occurrenceID: 652BBA63-4F86-5ADF-B52B-834CC305BE32; **Taxon:** scientificName: Tenuiphantesherbicola (Simon, 1884); order: Araneae; family: Linyphiidae; genus: Tenuiphantes; **Location:** country: Italy; countryCode: IT; stateProvince: Rome; county: Rome; municipality: Rome; locality: Appia Antica Regional Park, Rome; locationRemarks: Acqua Santa; decimalLatitude: 41.850561; decimalLongitude: 12.530861; geodeticDatum: WGS84; **Identification:** identifiedBy: Tommaso Fusco; dateIdentified: 2022; **Event:** samplingProtocol: Pitfall traps; eventDate: 2014-05-28; **Record Level:** collectionID: Roma3_5.8
- **Type status:** Other material. **Occurrence:** recordedBy: Fattorini S., Di Giulio A.; individualCount: 1; sex: female; lifeStage: adult; occurrenceID: 1E3E2672-DB14-5B5D-BC78-2E5230D439F2; **Taxon:** scientificName: Tenuiphantesherbicola (Simon, 1884); order: Araneae; family: Linyphiidae; genus: Tenuiphantes; **Location:** country: Italy; countryCode: IT; stateProvince: Rome; county: Rome; municipality: Rome; locality: Appia Antica Regional Park, Rome; locationRemarks: Caffarella Centro; decimalLatitude: 41.864889; decimalLongitude: 12.516389; geodeticDatum: WGS84; **Identification:** identifiedBy: Tommaso Fusco; dateIdentified: 2022; **Event:** samplingProtocol: Pitfall traps; eventDate: 2013-11-13; **Record Level:** collectionID: Roma3_5.8
- **Type status:** Other material. **Occurrence:** recordedBy: Fattorini S., Di Giulio A.; individualCount: 1; sex: male; lifeStage: adult; occurrenceID: 8C5972AB-1EC6-53A3-91F2-422A3743654E; **Taxon:** scientificName: Tenuiphantesherbicola (Simon, 1884); order: Araneae; family: Linyphiidae; genus: Tenuiphantes; **Location:** country: Italy; countryCode: IT; stateProvince: Rome; county: Rome; municipality: Rome; locality: Appia Antica Regional Park, Rome; locationRemarks: Caffarella Centro; decimalLatitude: 41.864889; decimalLongitude: 12.516389; geodeticDatum: WGS84; **Identification:** identifiedBy: Tommaso Fusco; dateIdentified: 2022; **Event:** samplingProtocol: Pitfall traps; eventDate: 2014-06-06; **Record Level:** collectionID: Roma3_5.8
- **Type status:** Other material. **Occurrence:** recordedBy: Fattorini S., Di Giulio A.; individualCount: 3; sex: 2 male, 1 female; lifeStage: adult; occurrenceID: 5CA1D5CC-7045-5733-8684-5CDEF9C9DBFA; **Taxon:** scientificName: Tenuiphantesherbicola (Simon, 1884); order: Araneae; family: Linyphiidae; genus: Tenuiphantes; **Location:** country: Italy; countryCode: IT; stateProvince: Rome; county: Rome; municipality: Rome; locality: Appia Antica Regional Park, Rome; locationRemarks: Caffarella Centro; decimalLatitude: 41.864889; decimalLongitude: 12.516389; geodeticDatum: WGS84; **Identification:** identifiedBy: Tommaso Fusco; dateIdentified: 2022; **Event:** samplingProtocol: Pitfall traps; eventDate: 2014-05-27; **Record Level:** collectionID: Roma3_5.8
- **Type status:** Other material. **Occurrence:** recordedBy: Fattorini S., Di Giulio A.; individualCount: 2; sex: 1 male, 1 female; lifeStage: adult; occurrenceID: F2BEFCA4-5CF0-5FDC-BA9A-282BC5A446DE; **Taxon:** scientificName: Tenuiphantesherbicola (Simon, 1884); order: Araneae; family: Linyphiidae; genus: Tenuiphantes; **Location:** country: Italy; countryCode: IT; stateProvince: Rome; county: Rome; municipality: Rome; locality: Appia Antica Regional Park, Rome; locationRemarks: Caffarella Centro; decimalLatitude: 41.864889; decimalLongitude: 12.516389; geodeticDatum: WGS84; **Identification:** identifiedBy: Tommaso Fusco; dateIdentified: 2022; **Event:** samplingProtocol: Pitfall traps; eventDate: 2014-06-06; **Record Level:** collectionID: Roma3_5.8
- **Type status:** Other material. **Occurrence:** recordedBy: Fattorini S., Di Giulio A.; individualCount: 2; sex: female; lifeStage: adult; occurrenceID: 3B48CD30-014F-526D-956D-B11201B49F49; **Taxon:** scientificName: Tenuiphantesherbicola (Simon, 1884); order: Araneae; family: Linyphiidae; genus: Tenuiphantes; **Location:** country: Italy; countryCode: IT; stateProvince: Rome; county: Rome; municipality: Rome; locality: Appia Antica Regional Park, Rome; locationRemarks: Caffarella Centro; decimalLatitude: 41.864889; decimalLongitude: 12.516389; geodeticDatum: WGS84; **Identification:** identifiedBy: Tommaso Fusco; dateIdentified: 2022; **Event:** samplingProtocol: Pitfall traps; eventDate: 2014-06-18; **Record Level:** collectionID: Roma3_5.8
- **Type status:** Other material. **Occurrence:** recordedBy: Fattorini S., Di Giulio A.; individualCount: 1; sex: male; lifeStage: adult; occurrenceID: 8D3E7F18-3765-57D3-BE40-B81705293170; **Taxon:** scientificName: Tenuiphantesherbicola (Simon, 1884); order: Araneae; family: Linyphiidae; genus: Tenuiphantes; **Location:** country: Italy; countryCode: IT; stateProvince: Rome; county: Rome; municipality: Rome; locality: Appia Antica Regional Park, Rome; locationRemarks: Caffarella Nord; decimalLatitude: 41.867753; decimalLongitude: 12.512414; geodeticDatum: WGS84; **Identification:** identifiedBy: Tommaso Fusco; dateIdentified: 2022; **Event:** samplingProtocol: Pitfall traps; eventDate: 2013-10-31; **Record Level:** collectionID: Roma3_5.8
- **Type status:** Other material. **Occurrence:** recordedBy: Fattorini S., Di Giulio A.; individualCount: 1; sex: male; lifeStage: adult; occurrenceID: 31908B03-865F-5566-863D-A6DF451D1955; **Taxon:** scientificName: Tenuiphantesherbicola (Simon, 1884); order: Araneae; family: Linyphiidae; genus: Tenuiphantes; **Location:** country: Italy; countryCode: IT; stateProvince: Rome; county: Rome; municipality: Rome; locality: Appia Antica Regional Park, Rome; locationRemarks: Caffarella Nord; decimalLatitude: 41.867753; decimalLongitude: 12.512414; geodeticDatum: WGS84; **Identification:** identifiedBy: Tommaso Fusco; dateIdentified: 2022; **Event:** samplingProtocol: Pitfall traps; eventDate: 2014-05-19; **Record Level:** collectionID: Roma3_5.8
- **Type status:** Other material. **Occurrence:** recordedBy: Fattorini S., Di Giulio A.; individualCount: 1; sex: female; lifeStage: adult; occurrenceID: 6CDB53D8-8B34-56AA-A321-BCC8331909B2; **Taxon:** scientificName: Tenuiphantesherbicola (Simon, 1884); order: Araneae; family: Linyphiidae; genus: Tenuiphantes; **Location:** country: Italy; countryCode: IT; stateProvince: Rome; county: Rome; municipality: Rome; locality: Appia Antica Regional Park, Rome; locationRemarks: Caffarella Nord; decimalLatitude: 41.867753; decimalLongitude: 12.512414; geodeticDatum: WGS84; **Identification:** identifiedBy: Tommaso Fusco; dateIdentified: 2022; **Event:** samplingProtocol: Pitfall traps; eventDate: 2013-11-13; **Record Level:** collectionID: Roma3_5.8
- **Type status:** Other material. **Occurrence:** recordedBy: Fattorini S., Di Giulio A.; individualCount: 2; sex: 1 male, 1 female; lifeStage: adult; occurrenceID: 386DE50A-A0EA-51F2-910D-FA3FFA506544; **Taxon:** scientificName: Tenuiphantesherbicola (Simon, 1884); order: Araneae; family: Linyphiidae; genus: Tenuiphantes; **Location:** country: Italy; countryCode: IT; stateProvince: Rome; county: Rome; municipality: Rome; locality: Appia Antica Regional Park, Rome; locationRemarks: Caffarella Nord; decimalLatitude: 41.867753; decimalLongitude: 12.512414; geodeticDatum: WGS84; **Identification:** identifiedBy: Tommaso Fusco; dateIdentified: 2022; **Event:** samplingProtocol: Pitfall traps; eventDate: 2014-06-06; **Record Level:** collectionID: Roma3_5.8
- **Type status:** Other material. **Occurrence:** recordedBy: Fattorini S., Di Giulio A.; individualCount: 1; sex: female; lifeStage: adult; occurrenceID: DF253D3D-3390-5E9A-A7C5-75962A8B22D5; **Taxon:** scientificName: Tenuiphantesherbicola (Simon, 1884); order: Araneae; family: Linyphiidae; genus: Tenuiphantes; **Location:** country: Italy; countryCode: IT; stateProvince: Rome; county: Rome; municipality: Rome; locality: Appia Antica Regional Park, Rome; locationRemarks: Caffarella Nord; decimalLatitude: 41.867753; decimalLongitude: 12.512414; geodeticDatum: WGS84; **Identification:** identifiedBy: Tommaso Fusco; dateIdentified: 2022; **Event:** samplingProtocol: Pitfall traps; eventDate: 2013-11-29; **Record Level:** collectionID: Roma3_5.8
- **Type status:** Other material. **Occurrence:** recordedBy: Fattorini S., Di Giulio A.; individualCount: 1; sex: female; lifeStage: adult; occurrenceID: 60552B90-0914-5134-B4D0-F3B8DB314484; **Taxon:** scientificName: Tenuiphantesherbicola (Simon, 1884); order: Araneae; family: Linyphiidae; genus: Tenuiphantes; **Location:** country: Italy; countryCode: IT; stateProvince: Rome; county: Rome; municipality: Rome; locality: Appia Antica Regional Park, Rome; locationRemarks: Cava Fiorucci; decimalLatitude: 41.834106; decimalLongitude: 12.549264; geodeticDatum: WGS84; **Identification:** identifiedBy: Tommaso Fusco; dateIdentified: 2022; **Event:** samplingProtocol: Pitfall traps; eventDate: 2014-05-26; **Record Level:** collectionID: Roma3_5.8
- **Type status:** Other material. **Occurrence:** recordedBy: Fattorini S., Di Giulio A.; individualCount: 1; sex: female; lifeStage: adult; occurrenceID: E2C9E41A-84CF-54BA-9F0C-BE7EDC7DB4C2; **Taxon:** scientificName: Tenuiphantesherbicola (Simon, 1884); order: Araneae; family: Linyphiidae; genus: Tenuiphantes; **Location:** country: Italy; countryCode: IT; stateProvince: Rome; county: Rome; municipality: Rome; locality: Appia Antica Regional Park, Rome; locationRemarks: Cava Fiorucci; decimalLatitude: 41.834106; decimalLongitude: 12.549264; geodeticDatum: WGS84; **Identification:** identifiedBy: Tommaso Fusco; dateIdentified: 2022; **Event:** samplingProtocol: Pitfall traps; eventDate: 2013-11-18; **Record Level:** collectionID: Roma3_5.8
- **Type status:** Other material. **Occurrence:** recordedBy: Fattorini S., Di Giulio A.; individualCount: 1; sex: male; lifeStage: adult; occurrenceID: 660C2C79-4CFF-5D0B-BDC9-DA276E57468A; **Taxon:** scientificName: Tenuiphantesherbicola (Simon, 1884); order: Araneae; family: Linyphiidae; genus: Tenuiphantes; **Location:** country: Italy; countryCode: IT; stateProvince: Rome; county: Rome; municipality: Rome; locality: Appia Antica Regional Park, Rome; locationRemarks: Cava Fiorucci; decimalLatitude: 41.834106; decimalLongitude: 12.549264; geodeticDatum: WGS84; **Identification:** identifiedBy: Tommaso Fusco; dateIdentified: 2022; **Event:** samplingProtocol: Pitfall traps; eventDate: 2013-12-06; **Record Level:** collectionID: Roma3_5.8
- **Type status:** Other material. **Occurrence:** recordedBy: Fattorini S., Di Giulio A.; individualCount: 1; sex: female; lifeStage: adult; occurrenceID: A2C6ECC1-920F-550A-A620-077319ECC29A; **Taxon:** scientificName: Tenuiphantesherbicola (Simon, 1884); order: Araneae; family: Linyphiidae; genus: Tenuiphantes; **Location:** country: Italy; countryCode: IT; stateProvince: Rome; county: Rome; municipality: Rome; locality: Appia Antica Regional Park, Rome; locationRemarks: San Sebastiano; decimalLatitude: 41.855733; decimalLongitude: 12.515114; geodeticDatum: WGS84; **Identification:** identifiedBy: Tommaso Fusco; dateIdentified: 2022; **Event:** samplingProtocol: Pitfall traps; eventDate: 2014-05-20; **Record Level:** collectionID: Roma3_5.8
- **Type status:** Other material. **Occurrence:** recordedBy: Fattorini S., Di Giulio A.; individualCount: 1; sex: female; lifeStage: adult; occurrenceID: 2DDBCDF8-1B64-597F-BDF2-0F6A1D633564; **Taxon:** scientificName: Tenuiphantesherbicola (Simon, 1884); order: Araneae; family: Linyphiidae; genus: Tenuiphantes; **Location:** country: Italy; countryCode: IT; stateProvince: Rome; county: Rome; municipality: Rome; locality: Appia Antica Regional Park, Rome; locationRemarks: San Sebastiano; decimalLatitude: 41.855733; decimalLongitude: 12.515114; geodeticDatum: WGS84; **Identification:** identifiedBy: Tommaso Fusco; dateIdentified: 2022; **Event:** samplingProtocol: Pitfall traps; eventDate: 2013-11-12; **Record Level:** collectionID: Roma3_5.8
- **Type status:** Other material. **Occurrence:** recordedBy: Fattorini S., Di Giulio A.; individualCount: 2; sex: 1 male, 1 female; lifeStage: adult; occurrenceID: EA6CFD1E-6A3B-58FF-A321-AD9B10FD5055; **Taxon:** scientificName: Tenuiphantesherbicola (Simon, 1884); order: Araneae; family: Linyphiidae; genus: Tenuiphantes; **Location:** country: Italy; countryCode: IT; stateProvince: Rome; county: Rome; municipality: Rome; locality: Appia Antica Regional Park, Rome; locationRemarks: San Sebastiano; decimalLatitude: 41.855733; decimalLongitude: 12.515114; geodeticDatum: WGS84; **Identification:** identifiedBy: Tommaso Fusco; dateIdentified: 2022; **Event:** samplingProtocol: Pitfall traps; eventDate: 2014-05-28; **Record Level:** collectionID: Roma3_5.8
- **Type status:** Other material. **Occurrence:** recordedBy: Fattorini S., Di Giulio A.; individualCount: 1; sex: male; lifeStage: adult; occurrenceID: CAA5136E-6757-50F2-8D35-81066900ECDC; **Taxon:** scientificName: Tenuiphantesherbicola (Simon, 1884); order: Araneae; family: Linyphiidae; genus: Tenuiphantes; **Location:** country: Italy; countryCode: IT; stateProvince: Rome; county: Rome; municipality: Rome; locality: Appia Antica Regional Park, Rome; locationRemarks: San Sebastiano; decimalLatitude: 41.855733; decimalLongitude: 12.515114; geodeticDatum: WGS84; **Identification:** identifiedBy: Tommaso Fusco; dateIdentified: 2022; **Event:** samplingProtocol: Pitfall traps; eventDate: 2013-12-02; **Record Level:** collectionID: Roma3_5.8
- **Type status:** Other material. **Occurrence:** recordedBy: Fattorini S., Di Giulio A.; individualCount: 1; sex: male; lifeStage: adult; occurrenceID: 5092DD79-6B78-5FCE-9022-BD74533E7263; **Taxon:** scientificName: Tenuiphantesherbicola (Simon, 1884); order: Araneae; family: Linyphiidae; genus: Tenuiphantes; **Location:** country: Italy; countryCode: IT; stateProvince: Rome; county: Rome; municipality: Rome; locality: Appia Antica Regional Park, Rome; locationRemarks: San Sebastiano; decimalLatitude: 41.855733; decimalLongitude: 12.515114; geodeticDatum: WGS84; **Identification:** identifiedBy: Tommaso Fusco; dateIdentified: 2022; **Event:** samplingProtocol: Pitfall traps; eventDate: 2014-06-19; **Record Level:** collectionID: Roma3_5.8
- **Type status:** Other material. **Occurrence:** recordedBy: Fattorini S., Di Giulio A.; individualCount: 1; sex: female; lifeStage: adult; occurrenceID: D6A8F994-C704-5308-AA32-021D349AA386; **Taxon:** scientificName: Tenuiphantesherbicola (Simon, 1884); order: Araneae; family: Linyphiidae; genus: Tenuiphantes; **Location:** country: Italy; countryCode: IT; stateProvince: Rome; county: Rome; municipality: Rome; locality: Appia Antica Regional Park, Rome; locationRemarks: Tor Marancia; decimalLatitude: 41.850308; decimalLongitude: 12.503178; geodeticDatum: WGS84; **Identification:** identifiedBy: Tommaso Fusco; dateIdentified: 2022; **Event:** samplingProtocol: Pitfall traps; eventDate: 2014-06-05; **Record Level:** collectionID: Roma3_5.8
- **Type status:** Other material. **Occurrence:** recordedBy: Fattorini S., Di Giulio A.; individualCount: 1; sex: female; lifeStage: adult; occurrenceID: 32316F0D-0A5C-5E97-AA67-234A5E033539; **Taxon:** scientificName: Tenuiphantesherbicola (Simon, 1884); order: Araneae; family: Linyphiidae; genus: Tenuiphantes; **Location:** country: Italy; countryCode: IT; stateProvince: Rome; county: Rome; municipality: Rome; locality: Appia Antica Regional Park, Rome; locationRemarks: Tor Marancia; decimalLatitude: 41.850308; decimalLongitude: 12.503178; geodeticDatum: WGS84; **Identification:** identifiedBy: Tommaso Fusco; dateIdentified: 2022; **Event:** samplingProtocol: Pitfall traps; eventDate: 2013-11-27; **Record Level:** collectionID: Roma3_5.8

##### Distribution

Spain, France (including Corsica), Italy, Croatia, Albania, Greece, Algeria. Mediterranean (MED) chorotype.

#### Tenuiphantes tenuis

(Blackwall, 1852)

D61CFAEE-4D47-5661-9545-D4B4B56CFD2A

##### Materials

- **Type status:** Other material. **Occurrence:** recordedBy: Fattorini S., Di Giulio A.; individualCount: 1; sex: female; lifeStage: adult; occurrenceID: 2C7207AA-7AA7-5751-972E-AAA61B090AF5; **Taxon:** scientificName: Tenuiphantestenuis (Blackwall, 1852); order: Araneae; family: Linyphiidae; genus: Tenuiphantes; **Location:** country: Italy; countryCode: IT; stateProvince: Rome; county: Rome; municipality: Rome; locality: Appia Antica Regional Park, Rome; locationRemarks: Acqua Santa; decimalLatitude: 41.850561; decimalLongitude: 12.530861; geodeticDatum: WGS84; **Identification:** identifiedBy: Tommaso Fusco; dateIdentified: 2022; **Event:** samplingProtocol: Pitfall traps; eventDate: 2014-06-09; **Record Level:** collectionID: Roma3_5.8
- **Type status:** Other material. **Occurrence:** recordedBy: Fattorini S., Di Giulio A.; individualCount: 1; sex: male; lifeStage: adult; occurrenceID: 381A5C72-81C1-503E-961C-4F486D543F24; **Taxon:** scientificName: Tenuiphantestenuis (Blackwall, 1852); order: Araneae; family: Linyphiidae; genus: Tenuiphantes; **Location:** country: Italy; countryCode: IT; stateProvince: Rome; county: Rome; municipality: Rome; locality: Appia Antica Regional Park, Rome; locationRemarks: Acqua Santa; decimalLatitude: 41.850561; decimalLongitude: 12.530861; geodeticDatum: WGS84; **Identification:** identifiedBy: Tommaso Fusco; dateIdentified: 2022; **Event:** samplingProtocol: Pitfall traps; eventDate: 2013-12-02; **Record Level:** collectionID: Roma3_5.8
- **Type status:** Other material. **Occurrence:** recordedBy: Fattorini S., Di Giulio A.; individualCount: 1; sex: female; lifeStage: adult; occurrenceID: 35BACFC2-94F3-5D0E-88FD-AD7941530DAA; **Taxon:** scientificName: Tenuiphantestenuis (Blackwall, 1852); order: Araneae; family: Linyphiidae; genus: Tenuiphantes; **Location:** country: Italy; countryCode: IT; stateProvince: Rome; county: Rome; municipality: Rome; locality: Appia Antica Regional Park, Rome; locationRemarks: Acqua Santa; decimalLatitude: 41.850561; decimalLongitude: 12.530861; geodeticDatum: WGS84; **Identification:** identifiedBy: Tommaso Fusco; dateIdentified: 2022; **Event:** samplingProtocol: Pitfall traps; eventDate: 2014-06-19; **Record Level:** collectionID: Roma3_5.8
- **Type status:** Other material. **Occurrence:** recordedBy: Fattorini S., Di Giulio A.; individualCount: 2; sex: male; lifeStage: adult; occurrenceID: BE2015C5-017F-521D-AD36-3BBE7303CC49; **Taxon:** scientificName: Tenuiphantestenuis (Blackwall, 1852); order: Araneae; family: Linyphiidae; genus: Tenuiphantes; **Location:** country: Italy; countryCode: IT; stateProvince: Rome; county: Rome; municipality: Rome; locality: Appia Antica Regional Park, Rome; locationRemarks: Caffarella Nord; decimalLatitude: 41.867753; decimalLongitude: 12.512414; geodeticDatum: WGS84; **Identification:** identifiedBy: Tommaso Fusco; dateIdentified: 2022; **Event:** samplingProtocol: Pitfall traps; eventDate: 2014-05-19; **Record Level:** collectionID: Roma3_5.8
- **Type status:** Other material. **Occurrence:** recordedBy: Fattorini S., Di Giulio A.; individualCount: 1; sex: female; lifeStage: adult; occurrenceID: 0C3E22F2-1CEC-5688-9CF3-5C36C33D6A6E; **Taxon:** scientificName: Tenuiphantestenuis (Blackwall, 1852); order: Araneae; family: Linyphiidae; genus: Tenuiphantes; **Location:** country: Italy; countryCode: IT; stateProvince: Rome; county: Rome; municipality: Rome; locality: Appia Antica Regional Park, Rome; locationRemarks: Caffarella Nord; decimalLatitude: 41.867753; decimalLongitude: 12.512414; geodeticDatum: WGS84; **Identification:** identifiedBy: Tommaso Fusco; dateIdentified: 2022; **Event:** samplingProtocol: Pitfall traps; eventDate: 2013-11-13; **Record Level:** collectionID: Roma3_5.8
- **Type status:** Other material. **Occurrence:** recordedBy: Fattorini S., Di Giulio A.; individualCount: 1; sex: male; lifeStage: adult; occurrenceID: 66D8D262-DB60-5B9A-AE84-2F8289A6B6D5; **Taxon:** scientificName: Tenuiphantestenuis (Blackwall, 1852); order: Araneae; family: Linyphiidae; genus: Tenuiphantes; **Location:** country: Italy; countryCode: IT; stateProvince: Rome; county: Rome; municipality: Rome; locality: Appia Antica Regional Park, Rome; locationRemarks: Caffarella Nord; decimalLatitude: 41.867753; decimalLongitude: 12.512414; geodeticDatum: WGS84; **Identification:** identifiedBy: Tommaso Fusco; dateIdentified: 2022; **Event:** samplingProtocol: Pitfall traps; eventDate: 2014-05-27; **Record Level:** collectionID: Roma3_5.8
- **Type status:** Other material. **Occurrence:** recordedBy: Fattorini S., Di Giulio A.; individualCount: 1; sex: male; lifeStage: adult; occurrenceID: 0AB7FEE7-58C3-5001-BBB1-12B8CC05A16B; **Taxon:** scientificName: Tenuiphantestenuis (Blackwall, 1852); order: Araneae; family: Linyphiidae; genus: Tenuiphantes; **Location:** country: Italy; countryCode: IT; stateProvince: Rome; county: Rome; municipality: Rome; locality: Appia Antica Regional Park, Rome; locationRemarks: Caffarella Sud; decimalLatitude: 41.857247; decimalLongitude: 12.529211; geodeticDatum: WGS84; **Identification:** identifiedBy: Tommaso Fusco; dateIdentified: 2022; **Event:** samplingProtocol: Pitfall traps; eventDate: 2014-06-06; **Record Level:** collectionID: Roma3_5.8
- **Type status:** Other material. **Occurrence:** recordedBy: Fattorini S., Di Giulio A.; individualCount: 1; sex: male; lifeStage: adult; occurrenceID: B10AE4FE-AA33-5D92-9966-C3CC6AEAA2C1; **Taxon:** scientificName: Tenuiphantestenuis (Blackwall, 1852); order: Araneae; family: Linyphiidae; genus: Tenuiphantes; **Location:** country: Italy; countryCode: IT; stateProvince: Rome; county: Rome; municipality: Rome; locality: Appia Antica Regional Park, Rome; locationRemarks: Caffarella Sud; decimalLatitude: 41.857247; decimalLongitude: 12.529211; geodeticDatum: WGS84; **Identification:** identifiedBy: Tommaso Fusco; dateIdentified: 2022; **Event:** samplingProtocol: Pitfall traps; eventDate: 2014-05-19; **Record Level:** collectionID: Roma3_5.8
- **Type status:** Other material. **Occurrence:** recordedBy: Fattorini S., Di Giulio A.; individualCount: 1; sex: female; lifeStage: adult; occurrenceID: 8725ACA6-94BB-5772-98B6-D838FCCEFA0D; **Taxon:** scientificName: Tenuiphantestenuis (Blackwall, 1852); order: Araneae; family: Linyphiidae; genus: Tenuiphantes; **Location:** country: Italy; countryCode: IT; stateProvince: Rome; county: Rome; municipality: Rome; locality: Appia Antica Regional Park, Rome; locationRemarks: Caffarella Sud; decimalLatitude: 41.857247; decimalLongitude: 12.529211; geodeticDatum: WGS84; **Identification:** identifiedBy: Tommaso Fusco; dateIdentified: 2022; **Event:** samplingProtocol: Pitfall traps; eventDate: 2014-05-27; **Record Level:** collectionID: Roma3_5.8
- **Type status:** Other material. **Occurrence:** recordedBy: Fattorini S., Di Giulio A.; individualCount: 1; sex: 2 male, 3 female; lifeStage: adult; occurrenceID: 4756EDD4-7F85-5294-9944-052A1CEF68D4; **Taxon:** scientificName: Tenuiphantestenuis (Blackwall, 1852); order: Araneae; family: Linyphiidae; genus: Tenuiphantes; **Location:** country: Italy; countryCode: IT; stateProvince: Rome; county: Rome; municipality: Rome; locality: Appia Antica Regional Park, Rome; locationRemarks: Casal Verbeni; decimalLatitude: 41.815250; decimalLongitude: 12.552222; geodeticDatum: WGS84; **Identification:** identifiedBy: Tommaso Fusco; dateIdentified: 2022; **Event:** samplingProtocol: Pitfall traps; eventDate: 2014-05-26; **Record Level:** collectionID: Roma3_5.8
- **Type status:** Other material. **Occurrence:** recordedBy: Fattorini S., Di Giulio A.; individualCount: 1; sex: male; lifeStage: adult; occurrenceID: 8147DCEE-0516-5106-B972-9B29FE3FBFCD; **Taxon:** scientificName: Tenuiphantestenuis (Blackwall, 1852); order: Araneae; family: Linyphiidae; genus: Tenuiphantes; **Location:** country: Italy; countryCode: IT; stateProvince: Rome; county: Rome; municipality: Rome; locality: Appia Antica Regional Park, Rome; locationRemarks: Casal Verbeni; decimalLatitude: 41.815250; decimalLongitude: 12.552222; geodeticDatum: WGS84; **Identification:** identifiedBy: Tommaso Fusco; dateIdentified: 2022; **Event:** samplingProtocol: Pitfall traps; eventDate: 2014-06-05; **Record Level:** collectionID: Roma3_5.8
- **Type status:** Other material. **Occurrence:** recordedBy: Fattorini S., Di Giulio A.; individualCount: 1; sex: female; lifeStage: adult; occurrenceID: 3FAABE82-38FA-518E-B8D3-6F08D63EED30; **Taxon:** scientificName: Tenuiphantestenuis (Blackwall, 1852); order: Araneae; family: Linyphiidae; genus: Tenuiphantes; **Location:** country: Italy; countryCode: IT; stateProvince: Rome; county: Rome; municipality: Rome; locality: Appia Antica Regional Park, Rome; locationRemarks: Cava Fiorucci; decimalLatitude: 41.834106; decimalLongitude: 12.549264; geodeticDatum: WGS84; **Identification:** identifiedBy: Tommaso Fusco; dateIdentified: 2022; **Event:** samplingProtocol: Pitfall traps; eventDate: 2014-05-26; **Record Level:** collectionID: Roma3_5.8
- **Type status:** Other material. **Occurrence:** recordedBy: Fattorini S., Di Giulio A.; individualCount: 1; sex: female; lifeStage: adult; occurrenceID: 500B98E8-E4F9-5724-8D77-8CB23223A4D8; **Taxon:** scientificName: Tenuiphantestenuis (Blackwall, 1852); order: Araneae; family: Linyphiidae; genus: Tenuiphantes; **Location:** country: Italy; countryCode: IT; stateProvince: Rome; county: Rome; municipality: Rome; locality: Appia Antica Regional Park, Rome; locationRemarks: Farnesiana; decimalLatitude: 41.839667; decimalLongitude: 12.525528; geodeticDatum: WGS84; **Identification:** identifiedBy: Tommaso Fusco; dateIdentified: 2022; **Event:** samplingProtocol: Pitfall traps; eventDate: 2014-05-28; **Record Level:** collectionID: Roma3_5.8
- **Type status:** Other material. **Occurrence:** recordedBy: Fattorini S., Di Giulio A.; individualCount: 1; sex: male; lifeStage: adult; occurrenceID: 102D0E81-ACCD-5651-91D2-EB2AF85DAAB1; **Taxon:** scientificName: Tenuiphantestenuis (Blackwall, 1852); order: Araneae; family: Linyphiidae; genus: Tenuiphantes; **Location:** country: Italy; countryCode: IT; stateProvince: Rome; county: Rome; municipality: Rome; locality: Appia Antica Regional Park, Rome; locationRemarks: Farnesiana; decimalLatitude: 41.839667; decimalLongitude: 12.525528; geodeticDatum: WGS84; **Identification:** identifiedBy: Tommaso Fusco; dateIdentified: 2022; **Event:** samplingProtocol: Pitfall traps; eventDate: 2013-12-02; **Record Level:** collectionID: Roma3_5.8
- **Type status:** Other material. **Occurrence:** recordedBy: Fattorini S., Di Giulio A.; individualCount: 1; sex: female; lifeStage: adult; occurrenceID: 51023C58-14AC-5E06-AAE8-EFF53CF66189; **Taxon:** scientificName: Tenuiphantestenuis (Blackwall, 1852); order: Araneae; family: Linyphiidae; genus: Tenuiphantes; **Location:** country: Italy; countryCode: IT; stateProvince: Rome; county: Rome; municipality: Rome; locality: Appia Antica Regional Park, Rome; locationRemarks: San Sebastiano; decimalLatitude: 41.855733; decimalLongitude: 12.515114; geodeticDatum: WGS84; **Identification:** identifiedBy: Tommaso Fusco; dateIdentified: 2022; **Event:** samplingProtocol: Pitfall traps; eventDate: 2013-11-25; **Record Level:** collectionID: Roma3_5.8
- **Type status:** Other material. **Occurrence:** recordedBy: Fattorini S., Di Giulio A.; individualCount: 2; sex: female; lifeStage: adult; occurrenceID: B4872D81-A6A2-5D4B-8C17-5448CDEADD1C; **Taxon:** scientificName: Tenuiphantestenuis (Blackwall, 1852); order: Araneae; family: Linyphiidae; genus: Tenuiphantes; **Location:** country: Italy; countryCode: IT; stateProvince: Rome; county: Rome; municipality: Rome; locality: Appia Antica Regional Park, Rome; locationRemarks: Tor Marancia; decimalLatitude: 41.850308; decimalLongitude: 12.503178; geodeticDatum: WGS84; **Identification:** identifiedBy: Tommaso Fusco; dateIdentified: 2022; **Event:** samplingProtocol: Pitfall traps; eventDate: 2014-05-15; **Record Level:** collectionID: Roma3_5.8
- **Type status:** Other material. **Occurrence:** recordedBy: Fattorini S., Di Giulio A.; individualCount: 2; sex: female; lifeStage: adult; occurrenceID: A6D378DB-CE1F-53D4-BD55-75E2E96421EF; **Taxon:** scientificName: Tenuiphantestenuis (Blackwall, 1852); order: Araneae; family: Linyphiidae; genus: Tenuiphantes; **Location:** country: Italy; countryCode: IT; stateProvince: Rome; county: Rome; municipality: Rome; locality: Appia Antica Regional Park, Rome; locationRemarks: Tor Marancia; decimalLatitude: 41.850308; decimalLongitude: 12.503178; geodeticDatum: WGS84; **Identification:** identifiedBy: Tommaso Fusco; dateIdentified: 2022; **Event:** samplingProtocol: Pitfall traps; eventDate: 2014-06-05; **Record Level:** collectionID: Roma3_5.8
- **Type status:** Other material. **Occurrence:** recordedBy: Fattorini S., Di Giulio A.; individualCount: 3; sex: male; lifeStage: adult; occurrenceID: 9FCC70DD-9F76-58F2-89B2-9655B68D96CD; **Taxon:** scientificName: Tenuiphantestenuis (Blackwall, 1852); order: Araneae; family: Linyphiidae; genus: Tenuiphantes; **Location:** country: Italy; countryCode: IT; stateProvince: Rome; county: Rome; municipality: Rome; locality: Appia Antica Regional Park, Rome; locationRemarks: Torre Selce; decimalLatitude: 41.816611; decimalLongitude: 12.560667; geodeticDatum: WGS84; **Identification:** identifiedBy: Tommaso Fusco; dateIdentified: 2022; **Event:** samplingProtocol: Pitfall traps; eventDate: 2014-05-26; **Record Level:** collectionID: Roma3_5.8
- **Type status:** Other material. **Occurrence:** recordedBy: Fattorini S., Di Giulio A.; individualCount: 2; sex: male; lifeStage: adult; occurrenceID: A27D0C1D-19F3-5EB9-941A-6F117796B18F; **Taxon:** scientificName: Tenuiphantestenuis (Blackwall, 1852); order: Araneae; family: Linyphiidae; genus: Tenuiphantes; **Location:** country: Italy; countryCode: IT; stateProvince: Rome; county: Rome; municipality: Rome; locality: Appia Antica Regional Park, Rome; locationRemarks: Torre Selce; decimalLatitude: 41.816611; decimalLongitude: 12.560667; geodeticDatum: WGS84; **Identification:** identifiedBy: Tommaso Fusco; dateIdentified: 2022; **Event:** samplingProtocol: Pitfall traps; eventDate: 2014-06-05; **Record Level:** collectionID: Roma3_5.8

##### Distribution

Macaronesia, northern Africa, Europe, Turkey, Caucasus, Russia (Europe to south Siberia), Iran, Kazakhstan, Central Asia. Introduced to Canada, USA, Chile, Argentina, Falkland Is., New Zealand. Cosmopolitan (COS) chorotype.

#### Trichoncus affinis

Kulczyński, 1894

C9242790-3CDA-50B4-8CE7-5F47BD828B19

##### Materials

- **Type status:** Other material. **Occurrence:** recordedBy: Fattorini S., Di Giulio A.; individualCount: 1; sex: female; lifeStage: adult; occurrenceID: 294BCFF1-36FE-5184-8E31-2BE44C2CE2DB; **Taxon:** scientificName: Trichoncusaffinis Kulczyński, 1894; order: Araneae; family: Linyphiidae; genus: Trichoncus; **Location:** country: Italy; countryCode: IT; stateProvince: Rome; county: Rome; municipality: Rome; locality: Appia Antica Regional Park, Rome; locationRemarks: Torre Selce; decimalLatitude: 41.816611; decimalLongitude: 12.560667; geodeticDatum: WGS84; **Identification:** identifiedBy: Tommaso Fusco; dateIdentified: 2022; **Event:** samplingProtocol: Pitfall traps; eventDate: 2014-06-05; **Record Level:** collectionID: Roma3_5.8
- **Type status:** Other material. **Occurrence:** recordedBy: Fattorini S., Di Giulio A.; individualCount: 2; sex: female; lifeStage: adult; occurrenceID: 4A14A4F5-989C-5110-B7E3-8C5FA726BBDC; **Taxon:** scientificName: Trichoncusaffinis Kulczyński, 1894; order: Araneae; family: Linyphiidae; genus: Trichoncus; **Location:** country: Italy; countryCode: IT; stateProvince: Rome; county: Rome; municipality: Rome; locality: Appia Antica Regional Park, Rome; locationRemarks: Farnesiana; decimalLatitude: 41.839667; decimalLongitude: 12.525528; geodeticDatum: WGS84; **Identification:** identifiedBy: Tommaso Fusco; dateIdentified: 2022; **Event:** samplingProtocol: Pitfall traps; eventDate: 2014-06-09; **Record Level:** collectionID: Roma3_5.8

##### Distribution

Europe, Caucasus. European (EUR) chorotype.

#### Trichoncus hackmani

Millidge, 1955

671AE0EF-5A40-5EB6-97B5-CEEC27A530C7

##### Materials

- **Type status:** Other material. **Occurrence:** recordedBy: Fattorini S., Di Giulio A.; individualCount: 1; sex: female; lifeStage: adult; occurrenceID: 5E440556-A09E-5112-9C09-D841BA57F783; **Taxon:** scientificName: Trichoncushackmani Millidge, 1955; order: Araneae; family: Linyphiidae; genus: Trichoncus; **Location:** country: Italy; countryCode: IT; stateProvince: Rome; county: Rome; municipality: Rome; locality: Appia Antica Regional Park, Rome; locationRemarks: Torre Selce; decimalLatitude: 41.816611; decimalLongitude: 12.560667; geodeticDatum: WGS84; **Identification:** identifiedBy: Tommaso Fusco; dateIdentified: 2022; **Event:** samplingProtocol: Pitfall traps; eventDate: 2014-06-24; **Record Level:** collectionID: Roma3_5.8

##### Distribution

Europe, Turkey. European (EUR) chorotype.

#### Trichoncus sordidus

Simon, 1884

BBFDCAF9-DA09-518F-B2CF-B1EDD3CE8F1F

##### Materials

- **Type status:** Other material. **Occurrence:** recordedBy: Fattorini S., Di Giulio A.; individualCount: 1; sex: male; lifeStage: adult; occurrenceID: 88CCDDAD-9AA7-5A52-8A95-8A2493F24FE4; **Taxon:** scientificName: Trichoncussordidus Simon, 1884; order: Araneae; family: Linyphiidae; genus: Trichoncus; **Location:** country: Italy; countryCode: IT; stateProvince: Rome; county: Rome; municipality: Rome; locality: Appia Antica Regional Park, Rome; locationRemarks: Cava Fiorucci; decimalLatitude: 41.834106; decimalLongitude: 12.549264; geodeticDatum: WGS84; **Identification:** identifiedBy: Tommaso Fusco; dateIdentified: 2022; **Event:** samplingProtocol: Pitfall traps; eventDate: 2014-05-26; **Record Level:** collectionID: Roma3_5.8
- **Type status:** Other material. **Occurrence:** recordedBy: Fattorini S., Di Giulio A.; individualCount: 2; sex: 1 female, 1 male; lifeStage: adult; occurrenceID: FBFD9436-9A3C-56E6-8618-361A6243479B; **Taxon:** scientificName: Trichoncussordidus Simon, 1884; order: Araneae; family: Linyphiidae; genus: Trichoncus; **Location:** country: Italy; countryCode: IT; stateProvince: Rome; county: Rome; municipality: Rome; locality: Appia Antica Regional Park, Rome; locationRemarks: Cava Fiorucci; decimalLatitude: 41.834106; decimalLongitude: 12.549264; geodeticDatum: WGS84; **Identification:** identifiedBy: Tommaso Fusco; dateIdentified: 2022; **Event:** samplingProtocol: Pitfall traps; eventDate: 2014-06-05; **Record Level:** collectionID: Roma3_5.8
- **Type status:** Other material. **Occurrence:** recordedBy: Fattorini S., Di Giulio A.; individualCount: 2; sex: female; lifeStage: adult; occurrenceID: B03A81B9-C569-51BC-AA5E-79648F547FB6; **Taxon:** scientificName: Trichoncussordidus Simon, 1884; order: Araneae; family: Linyphiidae; genus: Trichoncus; **Location:** country: Italy; countryCode: IT; stateProvince: Rome; county: Rome; municipality: Rome; locality: Appia Antica Regional Park, Rome; locationRemarks: Acqua Santa; decimalLatitude: 41.850561; decimalLongitude: 12.530861; geodeticDatum: WGS84; **Identification:** identifiedBy: Tommaso Fusco; dateIdentified: 2022; **Event:** samplingProtocol: Pitfall traps; eventDate: 2014-05-20; **Record Level:** collectionID: Roma3_5.8

##### Distribution

Most of southern Europe. S-European (SEU) chorotype.

#### Walckenaeria antica

(Wider, 1834)

316415A4-2ECC-59A3-A9D2-C93AD780024A

##### Materials

- **Type status:** Other material. **Occurrence:** recordedBy: Fattorini S., Di Giulio A.; individualCount: 3; sex: female; lifeStage: adult; occurrenceID: 9CE78AB7-10D3-59E4-91F7-78C9C8CB4B6E; **Taxon:** scientificName: Walckenaeriaantica (Wider, 1834); order: Araneae; family: Linyphiidae; genus: Walckenaeria; **Location:** country: Italy; countryCode: IT; stateProvince: Rome; county: Rome; municipality: Rome; locality: Appia Antica Regional Park, Rome; locationRemarks: Caffarella Sud; decimalLatitude: 41.857247; decimalLongitude: 12.529211; geodeticDatum: WGS84; **Identification:** identifiedBy: Tommaso Fusco; dateIdentified: 2022; **Event:** samplingProtocol: Pitfall traps; eventDate: 2014-06-06; **Record Level:** collectionID: Roma3_5.8
- **Type status:** Other material. **Occurrence:** recordedBy: Fattorini S., Di Giulio A.; individualCount: 1; sex: female; lifeStage: adult; occurrenceID: CC00C5AB-CB13-586A-9719-945303C1456F; **Taxon:** scientificName: Walckenaeriaantica (Wider, 1834); order: Araneae; family: Linyphiidae; genus: Walckenaeria; **Location:** country: Italy; countryCode: IT; stateProvince: Rome; county: Rome; municipality: Rome; locality: Appia Antica Regional Park, Rome; locationRemarks: Farnesiana; decimalLatitude: 41.839667; decimalLongitude: 12.525528; geodeticDatum: WGS84; **Identification:** identifiedBy: Tommaso Fusco; dateIdentified: 2022; **Event:** samplingProtocol: Pitfall traps; eventDate: 2014-06-19; **Record Level:** collectionID: Roma3_5.8
- **Type status:** Other material. **Occurrence:** recordedBy: Fattorini S., Di Giulio A.; individualCount: 1; sex: female; lifeStage: adult; occurrenceID: 6FBFC53D-1AD8-5822-95B3-DA0DA8878D02; **Taxon:** scientificName: Walckenaeriaantica (Wider, 1834); order: Araneae; family: Linyphiidae; genus: Walckenaeria; **Location:** country: Italy; countryCode: IT; stateProvince: Rome; county: Rome; municipality: Rome; locality: Appia Antica Regional Park, Rome; locationRemarks: Caffarella Nord; decimalLatitude: 41.867753; decimalLongitude: 12.512414; geodeticDatum: WGS84; **Identification:** identifiedBy: Tommaso Fusco; dateIdentified: 2022; **Event:** samplingProtocol: Pitfall traps; eventDate: 2014-05-27; **Record Level:** collectionID: Roma3_5.8

##### Distribution

Europe, Turkey, Caucasus, Russia (Europe to south Siberia), Kyrgyzstan, China, Korea, Japan. Asiatic-European (ASE) chorotype.

#### Liocranidae

Simon, 1897

B1D5248A-0BF3-5247-9A13-9E594B8F3F17

#### Agraecina lineata

(Simon, 1878)

D5277C7A-20C4-500C-8524-0E283F60176F

##### Materials

- **Type status:** Other material. **Occurrence:** recordedBy: Fattorini S., Di Giulio A.; individualCount: 1; sex: female; lifeStage: adult; occurrenceID: 30F0013E-2528-5564-B361-F85744B395B6; **Taxon:** scientificName: Agraecinalineata (Simon, 1878); order: Araneae; family: Liocranidae; genus: Agraecina; **Location:** country: Italy; countryCode: IT; stateProvince: Rome; county: Rome; municipality: Rome; locality: Appia Antica Regional Park, Rome; locationRemarks: Cava Fiorucci; decimalLatitude: 41.834106; decimalLongitude: 12.549264; geodeticDatum: WGS84; **Identification:** identifiedBy: Tommaso Fusco; dateIdentified: 2022; **Event:** samplingProtocol: Pitfall traps; eventDate: 2014-06-13; **Record Level:** collectionID: Roma3_5.8
- **Type status:** Other material. **Occurrence:** recordedBy: Fattorini S., Di Giulio A.; individualCount: 1; sex: male; lifeStage: adult; occurrenceID: 984E500B-E97D-57AC-984C-8FABF7C7DA4D; **Taxon:** scientificName: Agraecinalineata (Simon, 1878); order: Araneae; family: Liocranidae; genus: Agraecina; **Location:** country: Italy; countryCode: IT; stateProvince: Rome; county: Rome; municipality: Rome; locality: Appia Antica Regional Park, Rome; locationRemarks: Farnesiana; decimalLatitude: 41.839667; decimalLongitude: 12.525528; geodeticDatum: WGS84; **Identification:** identifiedBy: Tommaso Fusco; dateIdentified: 2022; **Event:** samplingProtocol: Pitfall traps; eventDate: 2014-06-19; **Record Level:** collectionID: Roma3_5.8

##### Distribution

Western Mediterranean to Kazakhstan. Turano-Europeo-Mediterranean (TEM) chorotype.

#### Agroeca cuprea

Menge, 1873

D4F90756-4C55-5E61-9BFC-AA3CF290FF77

##### Materials

- **Type status:** Other material. **Occurrence:** recordedBy: Fattorini S., Di Giulio A.; individualCount: 1; sex: female; lifeStage: adult; occurrenceID: A07A36FE-6B39-5110-AEB7-F9B819688783; **Taxon:** scientificName: Agroecacuprea Menge, 1873; order: Araneae; family: Liocranidae; genus: Agroeca; **Location:** country: Italy; countryCode: IT; stateProvince: Rome; county: Rome; municipality: Rome; locality: Appia Antica Regional Park, Rome; locationRemarks: Farnesiana; decimalLatitude: 41.839667; decimalLongitude: 12.525528; geodeticDatum: WGS84; **Identification:** identifiedBy: Tommaso Fusco; dateIdentified: 2022; **Event:** samplingProtocol: Pitfall traps; eventDate: 2014-05-26; **Record Level:** collectionID: Roma3_5.8
- **Type status:** Other material. **Occurrence:** recordedBy: Fattorini S., Di Giulio A.; individualCount: 2; sex: male; lifeStage: adult; occurrenceID: AA898849-1366-53D7-BC04-242CA6E5FE77; **Taxon:** scientificName: Agroecacuprea Menge, 1873; order: Araneae; family: Liocranidae; genus: Agroeca; **Location:** country: Italy; countryCode: IT; stateProvince: Rome; county: Rome; municipality: Rome; locality: Appia Antica Regional Park, Rome; locationRemarks: Caffarella Nord; decimalLatitude: 41.867753; decimalLongitude: 12.512414; geodeticDatum: WGS84; **Identification:** identifiedBy: Tommaso Fusco; dateIdentified: 2022; **Event:** samplingProtocol: Pitfall traps; eventDate: 2013-10-31; **Record Level:** collectionID: Roma3_5.8
- **Type status:** Other material. **Occurrence:** recordedBy: Fattorini S., Di Giulio A.; individualCount: 1; sex: female; lifeStage: adult; occurrenceID: 86C2264A-7EB2-54FC-877D-B067984E6001; **Taxon:** scientificName: Agroecacuprea Menge, 1873; order: Araneae; family: Liocranidae; genus: Agroeca; **Location:** country: Italy; countryCode: IT; stateProvince: Rome; county: Rome; municipality: Rome; locality: Appia Antica Regional Park, Rome; locationRemarks: Caffarella Nord; decimalLatitude: 41.867753; decimalLongitude: 12.512414; geodeticDatum: WGS84; **Identification:** identifiedBy: Tommaso Fusco; dateIdentified: 2022; **Event:** samplingProtocol: Pitfall traps; eventDate: 2014-05-19; **Record Level:** collectionID: Roma3_5.8

##### Distribution

Europe, Caucasus, Russia (Europe to south Siberia), Iran, Central Asia. Asiatic-European (ASE) chorotype.

#### Cybaeodes marinae

Di Franco, 1989

CCF5476B-498A-52DE-971D-413A0434E5D6

##### Materials

- **Type status:** Other material. **Occurrence:** recordedBy: Fattorini S., Di Giulio A.; individualCount: 1; sex: female; lifeStage: adult; occurrenceID: 21689743-8359-530D-A1D8-16E7FB0004BF; **Taxon:** scientificName: Cybaeodesmarinae Di Franco, 1989; order: Araneae; family: Liocranidae; genus: Cybaeodes; **Location:** country: Italy; countryCode: IT; stateProvince: Rome; county: Rome; municipality: Rome; locality: Appia Antica Regional Park, Rome; locationRemarks: Caffarella Nord; decimalLatitude: 41.867753; decimalLongitude: 12.512414; geodeticDatum: WGS84; **Identification:** identifiedBy: Tommaso Fusco; dateIdentified: 2022; **Event:** samplingProtocol: Pitfall traps; eventDate: 2013-10-31; **Record Level:** collectionID: Roma3_5.8
- **Type status:** Other material. **Occurrence:** recordedBy: Fattorini S., Di Giulio A.; individualCount: 1; sex: female; lifeStage: adult; occurrenceID: 3BFA2AB9-FB19-5EC0-A29C-EBA007605BE1; **Taxon:** scientificName: Cybaeodesmarinae Di Franco, 1989; order: Araneae; family: Liocranidae; genus: Cybaeodes; **Location:** country: Italy; countryCode: IT; stateProvince: Rome; county: Rome; municipality: Rome; locality: Appia Antica Regional Park, Rome; locationRemarks: Caffarella Sud 1; decimalLatitude: 41.857247; decimalLongitude: 12.529211; geodeticDatum: WGS84; **Identification:** identifiedBy: Tommaso Fusco; dateIdentified: 2022; **Event:** samplingProtocol: Pitfall traps; eventDate: 2014-05-27; **Record Level:** collectionID: Roma3_5.8
- **Type status:** Other material. **Occurrence:** recordedBy: Fattorini S., Di Giulio A.; individualCount: 1; sex: female; lifeStage: adult; occurrenceID: DC7B5315-CADC-574A-A85D-1AFB67794CBB; **Taxon:** scientificName: Cybaeodesmarinae Di Franco, 1989; order: Araneae; family: Liocranidae; genus: Cybaeodes; **Location:** country: Italy; countryCode: IT; stateProvince: Rome; county: Rome; municipality: Rome; locality: Appia Antica Regional Park, Rome; locationRemarks: Farnesiana; decimalLatitude: 41.839667; decimalLongitude: 12.525528; geodeticDatum: WGS84; **Identification:** identifiedBy: Tommaso Fusco; dateIdentified: 2022; **Event:** samplingProtocol: Pitfall traps; eventDate: 2014-06-19; **Record Level:** collectionID: Roma3_5.8
- **Type status:** Other material. **Occurrence:** recordedBy: Fattorini S., Di Giulio A.; individualCount: 1; sex: female; lifeStage: adult; occurrenceID: 4726911E-5369-5F5C-A4EC-A0FC541BAB3D; **Taxon:** scientificName: Cybaeodesmarinae Di Franco, 1989; order: Araneae; family: Liocranidae; genus: Cybaeodes; **Location:** country: Italy; countryCode: IT; stateProvince: Rome; county: Rome; municipality: Rome; locality: Appia Antica Regional Park, Rome; locationRemarks: Acqua Santa; decimalLatitude: 41.850561; decimalLongitude: 12.530861; geodeticDatum: WGS84; **Identification:** identifiedBy: Tommaso Fusco; dateIdentified: 2022; **Event:** samplingProtocol: Pitfall traps; eventDate: 2014-06-09; **Record Level:** collectionID: Roma3_5.8
- **Type status:** Other material. **Occurrence:** recordedBy: Fattorini S., Di Giulio A.; individualCount: 1; sex: male; lifeStage: adult; occurrenceID: 6E88B312-E15E-5F9A-81AC-FAC9E90BC05E; **Taxon:** scientificName: Cybaeodesmarinae Di Franco, 1989; order: Araneae; family: Liocranidae; genus: Cybaeodes; **Location:** country: Italy; countryCode: IT; stateProvince: Rome; county: Rome; municipality: Rome; locality: Appia Antica Regional Park, Rome; locationRemarks: Cava Fiorucci; decimalLatitude: 41.834106; decimalLongitude: 12.549264; geodeticDatum: WGS84; **Identification:** identifiedBy: Tommaso Fusco; dateIdentified: 2022; **Event:** samplingProtocol: Pitfall traps; eventDate: 2013-11-07; **Record Level:** collectionID: Roma3_5.8

##### Distribution

Italian Endemic (END) species from central and southern Italy (Di Franco 1989).

#### Lycosidae

Sundevall, 1833

63DE7B46-7DF9-5BD5-9A2D-BBB538590A5D

#### Alopecosa albofasciata

(Brullé, 1832)

83CB9376-4428-52BA-9B20-8E707A4D882D

##### Materials

- **Type status:** Other material. **Occurrence:** recordedBy: Fattorini S., Di Giulio A.; individualCount: 2; sex: 1 male, 1 female; lifeStage: adult; occurrenceID: 11A5EE27-94B3-5C82-AB5D-A4E54592ED84; **Taxon:** scientificName: Alopecosaalbofasciata (Brullé, 1832); order: Araneae; family: Lycosidae; genus: Alopecosa; **Location:** country: Italy; countryCode: IT; stateProvince: Rome; county: Rome; municipality: Rome; locality: Appia Antica Regional Park, Rome; locationRemarks: Acqua Santa; decimalLatitude: 41.850561; decimalLongitude: 12.530861; geodeticDatum: WGS84; **Identification:** identifiedBy: Tommaso Fusco; dateIdentified: 2022; **Event:** samplingProtocol: Pitfall traps; eventDate: 2014-05-28; **Record Level:** collectionID: Roma3_5.8
- **Type status:** Other material. **Occurrence:** recordedBy: Fattorini S., Di Giulio A.; individualCount: 4; sex: male; lifeStage: adult; occurrenceID: 594B6B3A-08C2-572B-B096-5D85D484FD84; **Taxon:** scientificName: Alopecosaalbofasciata (Brullé, 1832); order: Araneae; family: Lycosidae; genus: Alopecosa; **Location:** country: Italy; countryCode: IT; stateProvince: Rome; county: Rome; municipality: Rome; locality: Appia Antica Regional Park, Rome; locationRemarks: Acqua Santa; decimalLatitude: 41.850561; decimalLongitude: 12.530861; geodeticDatum: WGS84; **Identification:** identifiedBy: Tommaso Fusco; dateIdentified: 2022; **Event:** samplingProtocol: Pitfall traps; eventDate: 2014-05-20; **Record Level:** collectionID: Roma3_5.8
- **Type status:** Other material. **Occurrence:** recordedBy: Fattorini S., Di Giulio A.; individualCount: 2; sex: male; lifeStage: adult; occurrenceID: 56D11893-1FDC-5DEB-803B-CAD23DF36F00; **Taxon:** scientificName: Alopecosaalbofasciata (Brullé, 1832); order: Araneae; family: Lycosidae; genus: Alopecosa; **Location:** country: Italy; countryCode: IT; stateProvince: Rome; county: Rome; municipality: Rome; locality: Appia Antica Regional Park, Rome; locationRemarks: Acqua Santa; decimalLatitude: 41.850561; decimalLongitude: 12.530861; geodeticDatum: WGS84; **Identification:** identifiedBy: Tommaso Fusco; dateIdentified: 2022; **Event:** samplingProtocol: Pitfall traps; eventDate: 2014-06-09; **Record Level:** collectionID: Roma3_5.8
- **Type status:** Other material. **Occurrence:** recordedBy: Fattorini S., Di Giulio A.; individualCount: 1; sex: male; lifeStage: adult; occurrenceID: 7D0D8558-23A3-5EF8-B3FC-F45534A24FB0; **Taxon:** scientificName: Alopecosaalbofasciata (Brullé, 1832); order: Araneae; family: Lycosidae; genus: Alopecosa; **Location:** country: Italy; countryCode: IT; stateProvince: Rome; county: Rome; municipality: Rome; locality: Appia Antica Regional Park, Rome; locationRemarks: Appia Antica; decimalLatitude: 41.812575; decimalLongitude: 12.564011; geodeticDatum: WGS84; **Identification:** identifiedBy: Tommaso Fusco; dateIdentified: 2022; **Event:** samplingProtocol: Pitfall traps; eventDate: 2014-05-26; **Record Level:** collectionID: Roma3_5.8
- **Type status:** Other material. **Occurrence:** recordedBy: Fattorini S., Di Giulio A.; individualCount: 1; sex: male; lifeStage: adult; occurrenceID: 04D345ED-F13A-5BFF-8F99-BE806EEDC1A4; **Taxon:** scientificName: Alopecosaalbofasciata (Brullé, 1832); order: Araneae; family: Lycosidae; genus: Alopecosa; **Location:** country: Italy; countryCode: IT; stateProvince: Rome; county: Rome; municipality: Rome; locality: Appia Antica Regional Park, Rome; locationRemarks: Caffarella Nord; decimalLatitude: 41.867753; decimalLongitude: 12.512414; geodeticDatum: WGS84; **Identification:** identifiedBy: Tommaso Fusco; dateIdentified: 2022; **Event:** samplingProtocol: Pitfall traps; eventDate: 2014-06-06; **Record Level:** collectionID: Roma3_5.8
- **Type status:** Other material. **Occurrence:** recordedBy: Fattorini S., Di Giulio A.; individualCount: 1; sex: female; lifeStage: adult; occurrenceID: 81CB43A5-1586-5893-9B81-AE47E5E58568; **Taxon:** scientificName: Alopecosaalbofasciata (Brullé, 1832); order: Araneae; family: Lycosidae; genus: Alopecosa; **Location:** country: Italy; countryCode: IT; stateProvince: Rome; county: Rome; municipality: Rome; locality: Appia Antica Regional Park, Rome; locationRemarks: Caffarella Nord; decimalLatitude: 41.867753; decimalLongitude: 12.512414; geodeticDatum: WGS84; **Identification:** identifiedBy: Tommaso Fusco; dateIdentified: 2022; **Event:** samplingProtocol: Pitfall traps; eventDate: 2014-05-27; **Record Level:** collectionID: Roma3_5.8
- **Type status:** Other material. **Occurrence:** recordedBy: Fattorini S., Di Giulio A.; individualCount: 2; sex: male; lifeStage: adult; occurrenceID: C62A7C75-ECFB-522A-A390-EF13CD44361D; **Taxon:** scientificName: Alopecosaalbofasciata (Brullé, 1832); order: Araneae; family: Lycosidae; genus: Alopecosa; **Location:** country: Italy; countryCode: IT; stateProvince: Rome; county: Rome; municipality: Rome; locality: Appia Antica Regional Park, Rome; locationRemarks: Caffarella Sud; decimalLatitude: 41.857247; decimalLongitude: 12.529211; geodeticDatum: WGS84; **Identification:** identifiedBy: Tommaso Fusco; dateIdentified: 2022; **Event:** samplingProtocol: Pitfall traps; eventDate: 2014-05-27; **Record Level:** collectionID: Roma3_5.8
- **Type status:** Other material. **Occurrence:** recordedBy: Fattorini S., Di Giulio A.; individualCount: 4; sex: 2 male, 2 female; lifeStage: adult; occurrenceID: 69E39688-A15F-52D2-9AFC-0B9352233339; **Taxon:** scientificName: Alopecosaalbofasciata (Brullé, 1832); order: Araneae; family: Lycosidae; genus: Alopecosa; **Location:** country: Italy; countryCode: IT; stateProvince: Rome; county: Rome; municipality: Rome; locality: Appia Antica Regional Park, Rome; locationRemarks: Caffarella Sud; decimalLatitude: 41.857247; decimalLongitude: 12.529211; geodeticDatum: WGS84; **Identification:** identifiedBy: Tommaso Fusco; dateIdentified: 2022; **Event:** samplingProtocol: Pitfall traps; eventDate: 2014-05-19; **Record Level:** collectionID: Roma3_5.8
- **Type status:** Other material. **Occurrence:** recordedBy: Fattorini S., Di Giulio A.; individualCount: 1; sex: male; lifeStage: adult; occurrenceID: 13B4309A-679E-5BDA-BB97-1CBE7EAF7FA9; **Taxon:** scientificName: Alopecosaalbofasciata (Brullé, 1832); order: Araneae; family: Lycosidae; genus: Alopecosa; **Location:** country: Italy; countryCode: IT; stateProvince: Rome; county: Rome; municipality: Rome; locality: Appia Antica Regional Park, Rome; locationRemarks: Caffarella Sud 2; decimalLatitude: 41.856742; decimalLongitude: 12.529453; geodeticDatum: WGS84; **Identification:** identifiedBy: Tommaso Fusco; dateIdentified: 2022; **Event:** samplingProtocol: Pitfall traps; eventDate: 2014-06-06; **Record Level:** collectionID: Roma3_5.8
- **Type status:** Other material. **Occurrence:** recordedBy: Fattorini S., Di Giulio A.; individualCount: 6; sex: 3 male, 3 female; lifeStage: adult; occurrenceID: C7AB0FC7-89A5-57CF-8F04-9E2560A55599; **Taxon:** scientificName: Alopecosaalbofasciata (Brullé, 1832); order: Araneae; family: Lycosidae; genus: Alopecosa; **Location:** country: Italy; countryCode: IT; stateProvince: Rome; county: Rome; municipality: Rome; locality: Appia Antica Regional Park, Rome; locationRemarks: Caffarella Sud 3; decimalLatitude: 41.856928; decimalLongitude: 12.528406; geodeticDatum: WGS84; **Identification:** identifiedBy: Tommaso Fusco; dateIdentified: 2022; **Event:** samplingProtocol: Pitfall traps; eventDate: 2014-05-19; **Record Level:** collectionID: Roma3_5.8
- **Type status:** Other material. **Occurrence:** recordedBy: Fattorini S., Di Giulio A.; individualCount: 1; sex: female; lifeStage: adult; occurrenceID: 028EAB37-BFD6-595B-A25A-429052C919F6; **Taxon:** scientificName: Alopecosaalbofasciata (Brullé, 1832); order: Araneae; family: Lycosidae; genus: Alopecosa; **Location:** country: Italy; countryCode: IT; stateProvince: Rome; county: Rome; municipality: Rome; locality: Appia Antica Regional Park, Rome; locationRemarks: Casal Verbeni; decimalLatitude: 41.815250; decimalLongitude: 12.552222; geodeticDatum: WGS84; **Identification:** identifiedBy: Tommaso Fusco; dateIdentified: 2022; **Event:** samplingProtocol: Pitfall traps; eventDate: 2014-06-13; **Record Level:** collectionID: Roma3_5.8
- **Type status:** Other material. **Occurrence:** recordedBy: Fattorini S., Di Giulio A.; individualCount: 1; sex: male; lifeStage: adult; occurrenceID: 0C405A5C-9BBB-523A-AF8B-2DB4E38BD759; **Taxon:** scientificName: Alopecosaalbofasciata (Brullé, 1832); order: Araneae; family: Lycosidae; genus: Alopecosa; **Location:** country: Italy; countryCode: IT; stateProvince: Rome; county: Rome; municipality: Rome; locality: Appia Antica Regional Park, Rome; locationRemarks: Casal Verbeni; decimalLatitude: 41.815250; decimalLongitude: 12.552222; geodeticDatum: WGS84; **Identification:** identifiedBy: Tommaso Fusco; dateIdentified: 2022; **Event:** samplingProtocol: Pitfall traps; eventDate: 2014-05-26; **Record Level:** collectionID: Roma3_5.8
- **Type status:** Other material. **Occurrence:** recordedBy: Fattorini S., Di Giulio A.; individualCount: 1; sex: male; lifeStage: adult; occurrenceID: C91D55DA-8A85-5585-ADDF-3F7111F7155E; **Taxon:** scientificName: Alopecosaalbofasciata (Brullé, 1832); order: Araneae; family: Lycosidae; genus: Alopecosa; **Location:** country: Italy; countryCode: IT; stateProvince: Rome; county: Rome; municipality: Rome; locality: Appia Antica Regional Park, Rome; locationRemarks: Cava Fiorucci; decimalLatitude: 41.834106; decimalLongitude: 12.549264; geodeticDatum: WGS84; **Identification:** identifiedBy: Tommaso Fusco; dateIdentified: 2022; **Event:** samplingProtocol: Pitfall traps; eventDate: 2014-06-13; **Record Level:** collectionID: Roma3_5.8
- **Type status:** Other material. **Occurrence:** recordedBy: Fattorini S., Di Giulio A.; individualCount: 1; sex: male; lifeStage: adult; occurrenceID: 8EA884F1-78D2-5F1A-93E2-8D22EFC279EB; **Taxon:** scientificName: Alopecosaalbofasciata (Brullé, 1832); order: Araneae; family: Lycosidae; genus: Alopecosa; **Location:** country: Italy; countryCode: IT; stateProvince: Rome; county: Rome; municipality: Rome; locality: Appia Antica Regional Park, Rome; locationRemarks: Cava Fiorucci; decimalLatitude: 41.834106; decimalLongitude: 12.549264; geodeticDatum: WGS84; **Identification:** identifiedBy: Tommaso Fusco; dateIdentified: 2022; **Event:** samplingProtocol: Pitfall traps; eventDate: 2014-05-26; **Record Level:** collectionID: Roma3_5.8
- **Type status:** Other material. **Occurrence:** recordedBy: Fattorini S., Di Giulio A.; individualCount: 1; sex: male; lifeStage: adult; occurrenceID: DAE0404C-ADC4-553A-92BA-0546A74693B6; **Taxon:** scientificName: Alopecosaalbofasciata (Brullé, 1832); order: Araneae; family: Lycosidae; genus: Alopecosa; **Location:** country: Italy; countryCode: IT; stateProvince: Rome; county: Rome; municipality: Rome; locality: Appia Antica Regional Park, Rome; locationRemarks: Cava Fiorucci; decimalLatitude: 41.834106; decimalLongitude: 12.549264; geodeticDatum: WGS84; **Identification:** identifiedBy: Tommaso Fusco; dateIdentified: 2022; **Event:** samplingProtocol: Pitfall traps; eventDate: 2014-05-26; **Record Level:** collectionID: Roma3_5.8
- **Type status:** Other material. **Occurrence:** recordedBy: Fattorini S., Di Giulio A.; individualCount: 1; sex: female; lifeStage: adult; occurrenceID: 42701CFB-1033-5F9E-B2E2-695E24BB0F14; **Taxon:** scientificName: Alopecosaalbofasciata (Brullé, 1832); order: Araneae; family: Lycosidae; genus: Alopecosa; **Location:** country: Italy; countryCode: IT; stateProvince: Rome; county: Rome; municipality: Rome; locality: Appia Antica Regional Park, Rome; locationRemarks: Cava Fiorucci; decimalLatitude: 41.834106; decimalLongitude: 12.549264; geodeticDatum: WGS84; **Identification:** identifiedBy: Tommaso Fusco; dateIdentified: 2022; **Event:** samplingProtocol: Pitfall traps; eventDate: 2014-06-05; **Record Level:** collectionID: Roma3_5.8
- **Type status:** Other material. **Occurrence:** recordedBy: Fattorini S., Di Giulio A.; individualCount: 1; sex: male; lifeStage: adult; occurrenceID: 3E263521-2A85-5B97-8BF3-6F19B6F87BDE; **Taxon:** scientificName: Alopecosaalbofasciata (Brullé, 1832); order: Araneae; family: Lycosidae; genus: Alopecosa; **Location:** country: Italy; countryCode: IT; stateProvince: Rome; county: Rome; municipality: Rome; locality: Appia Antica Regional Park, Rome; locationRemarks: Farnesiana; decimalLatitude: 41.839667; decimalLongitude: 12.525528; geodeticDatum: WGS84; **Identification:** identifiedBy: Tommaso Fusco; dateIdentified: 2022; **Event:** samplingProtocol: Pitfall traps; eventDate: 2014-05-28; **Record Level:** collectionID: Roma3_5.8
- **Type status:** Other material. **Occurrence:** recordedBy: Fattorini S., Di Giulio A.; individualCount: 1; sex: male; lifeStage: adult; occurrenceID: CCC8448D-3D7F-5677-8219-E4EBD5B42AC4; **Taxon:** scientificName: Alopecosaalbofasciata (Brullé, 1832); order: Araneae; family: Lycosidae; genus: Alopecosa; **Location:** country: Italy; countryCode: IT; stateProvince: Rome; county: Rome; municipality: Rome; locality: Appia Antica Regional Park, Rome; locationRemarks: Farnesiana; decimalLatitude: 41.839667; decimalLongitude: 12.525528; geodeticDatum: WGS84; **Identification:** identifiedBy: Tommaso Fusco; dateIdentified: 2022; **Event:** samplingProtocol: Pitfall traps; eventDate: 2014-06-09; **Record Level:** collectionID: Roma3_5.8
- **Type status:** Other material. **Occurrence:** recordedBy: Fattorini S., Di Giulio A.; individualCount: 1; sex: female; lifeStage: adult; occurrenceID: 1C69760A-93D9-52AF-AD56-2296BE29B812; **Taxon:** scientificName: Alopecosaalbofasciata (Brullé, 1832); order: Araneae; family: Lycosidae; genus: Alopecosa; **Location:** country: Italy; countryCode: IT; stateProvince: Rome; county: Rome; municipality: Rome; locality: Appia Antica Regional Park, Rome; locationRemarks: San Sebastiano; decimalLatitude: 41.855733; decimalLongitude: 12.515114; geodeticDatum: WGS84; **Identification:** identifiedBy: Tommaso Fusco; dateIdentified: 2022; **Event:** samplingProtocol: Pitfall traps; eventDate: 2014-05-20; **Record Level:** collectionID: Roma3_5.8
- **Type status:** Other material. **Occurrence:** recordedBy: Fattorini S., Di Giulio A.; individualCount: 1; sex: male; lifeStage: adult; occurrenceID: 5695E9F4-996A-553D-B46D-34DF87E42FA5; **Taxon:** scientificName: Alopecosaalbofasciata (Brullé, 1832); order: Araneae; family: Lycosidae; genus: Alopecosa; **Location:** country: Italy; countryCode: IT; stateProvince: Rome; county: Rome; municipality: Rome; locality: Appia Antica Regional Park, Rome; locationRemarks: San Sebastiano; decimalLatitude: 41.855733; decimalLongitude: 12.515114; geodeticDatum: WGS84; **Identification:** identifiedBy: Tommaso Fusco; dateIdentified: 2022; **Event:** samplingProtocol: Pitfall traps; eventDate: 2014-06-09; **Record Level:** collectionID: Roma3_5.8
- **Type status:** Other material. **Occurrence:** recordedBy: Fattorini S., Di Giulio A.; individualCount: 1; sex: male; lifeStage: adult; occurrenceID: 30A79BF5-51D2-5CC9-AACC-718DEA292821; **Taxon:** scientificName: Alopecosaalbofasciata (Brullé, 1832); order: Araneae; family: Lycosidae; genus: Alopecosa; **Location:** country: Italy; countryCode: IT; stateProvince: Rome; county: Rome; municipality: Rome; locality: Appia Antica Regional Park, Rome; locationRemarks: Tor Marancia; decimalLatitude: 41.850308; decimalLongitude: 12.503178; geodeticDatum: WGS84; **Identification:** identifiedBy: Tommaso Fusco; dateIdentified: 2022; **Event:** samplingProtocol: Pitfall traps; eventDate: 2014-06-05; **Record Level:** collectionID: Roma3_5.8
- **Type status:** Other material. **Occurrence:** recordedBy: Fattorini S., Di Giulio A.; individualCount: 13; sex: 12 male, 1 female; lifeStage: adult; occurrenceID: BFB268CD-E018-5595-A5DC-D9C293639F4D; **Taxon:** scientificName: Alopecosaalbofasciata (Brullé, 1832); order: Araneae; family: Lycosidae; genus: Alopecosa; **Location:** country: Italy; countryCode: IT; stateProvince: Rome; county: Rome; municipality: Rome; locality: Appia Antica Regional Park, Rome; locationRemarks: Torre Selce; decimalLatitude: 41.816611; decimalLongitude: 12.560667; geodeticDatum: WGS84; **Identification:** identifiedBy: Tommaso Fusco; dateIdentified: 2022; **Event:** samplingProtocol: Pitfall traps; eventDate: 2014-05-26; **Record Level:** collectionID: Roma3_5.8
- **Type status:** Other material. **Occurrence:** recordedBy: Fattorini S., Di Giulio A.; individualCount: 1; sex: male; lifeStage: adult; occurrenceID: 5EB6DFDD-1841-5DF5-AA85-E45627823143; **Taxon:** scientificName: Alopecosaalbofasciata (Brullé, 1832); order: Araneae; family: Lycosidae; genus: Alopecosa; **Location:** country: Italy; countryCode: IT; stateProvince: Rome; county: Rome; municipality: Rome; locality: Appia Antica Regional Park, Rome; locationRemarks: Torre Selce; decimalLatitude: 41.816611; decimalLongitude: 12.560667; geodeticDatum: WGS84; **Identification:** identifiedBy: Tommaso Fusco; dateIdentified: 2022; **Event:** samplingProtocol: Pitfall traps; eventDate: 2014-06-05; **Record Level:** collectionID: Roma3_5.8
- **Type status:** Other material. **Occurrence:** recordedBy: Fattorini S., Di Giulio A.; individualCount: 4; sex: 2 male, 2 female; lifeStage: adult; occurrenceID: 34C94634-0A85-5BC8-9ED7-A2DAAD5279F5; **Taxon:** scientificName: Alopecosaalbofasciata (Brullé, 1832); order: Araneae; family: Lycosidae; genus: Alopecosa; **Location:** country: Italy; countryCode: IT; stateProvince: Rome; county: Rome; municipality: Rome; locality: Appia Antica Regional Park, Rome; locationRemarks: Torre Selce; decimalLatitude: 41.816611; decimalLongitude: 12.560667; geodeticDatum: WGS84; **Identification:** identifiedBy: Tommaso Fusco; dateIdentified: 2022; **Event:** samplingProtocol: Pitfall traps; eventDate: 2014-06-13; **Record Level:** collectionID: Roma3_5.8
- **Type status:** Other material. **Occurrence:** recordedBy: Fattorini S., Di Giulio A.; individualCount: 3; sex: male; lifeStage: adult; occurrenceID: FB0BD588-F14A-50AD-913E-FDE5FB59A8A3; **Taxon:** scientificName: Alopecosaalbofasciata (Brullé, 1832); order: Araneae; family: Lycosidae; genus: Alopecosa; **Location:** country: Italy; countryCode: IT; stateProvince: Rome; county: Rome; municipality: Rome; locality: Appia Antica Regional Park, Rome; locationRemarks: Torre Selce; decimalLatitude: 41.816611; decimalLongitude: 12.560667; geodeticDatum: WGS84; **Identification:** identifiedBy: Tommaso Fusco; dateIdentified: 2022; **Event:** samplingProtocol: Pitfall traps; eventDate: 2014-06-05; **Record Level:** collectionID: Roma3_5.8
- **Type status:** Other material. **Occurrence:** recordedBy: Fattorini S., Di Giulio A.; individualCount: 2; sex: female; lifeStage: adult; occurrenceID: F0B0D0ED-0C21-5225-BD7C-E43FCA98A4B9; **Taxon:** scientificName: Alopecosaalbofasciata (Brullé, 1832); order: Araneae; family: Lycosidae; genus: Alopecosa; **Location:** country: Italy; countryCode: IT; stateProvince: Rome; county: Rome; municipality: Rome; locality: Appia Antica Regional Park, Rome; locationRemarks: Torre Selce; decimalLatitude: 41.816611; decimalLongitude: 12.560667; geodeticDatum: WGS84; **Identification:** identifiedBy: Tommaso Fusco; dateIdentified: 2022; **Event:** samplingProtocol: Pitfall traps; eventDate: 2014-06-24; **Record Level:** collectionID: Roma3_5.8

##### Distribution

Mediterranean to Iraq. Turano-Mediterranean (TUM) chorotype.

#### Arctosa personata

(L. Koch, 1872)

4309AADF-A8D1-5DFD-95B5-DBAB8D12D578

##### Materials

- **Type status:** Other material. **Occurrence:** recordedBy: Fattorini S., Di Giulio A.; individualCount: 1; sex: male; lifeStage: adult; occurrenceID: 02A2BC33-D6E4-59B4-9A77-E710538886BE; **Taxon:** scientificName: Arctosapersonata (L. Koch, 1872); order: Araneae; family: Lycosidae; genus: Arctosa; **Location:** country: Italy; countryCode: IT; stateProvince: Rome; county: Rome; municipality: Rome; locality: Appia Antica Regional Park, Rome; locationRemarks: Acqua santa; decimalLatitude: 41.850561; decimalLongitude: 12.530861; geodeticDatum: WGS84; **Identification:** identifiedBy: Tommaso Fusco; dateIdentified: 2022; **Event:** samplingProtocol: Pitfall traps; eventDate: 2014-06-19; **Record Level:** collectionID: Roma3_5.8
- **Type status:** Other material. **Occurrence:** recordedBy: Fattorini S., Di Giulio A.; individualCount: 1; sex: male; lifeStage: adult; occurrenceID: C207A824-4B4A-535A-A752-82DBAA49D74C; **Taxon:** scientificName: Arctosapersonata (L. Koch, 1872); order: Araneae; family: Lycosidae; genus: Arctosa; **Location:** country: Italy; countryCode: IT; stateProvince: Rome; county: Rome; municipality: Rome; locality: Appia Antica Regional Park, Rome; locationRemarks: Appia Antica; decimalLatitude: 41.812575; decimalLongitude: 12.564011; geodeticDatum: WGS84; **Identification:** identifiedBy: Tommaso Fusco; dateIdentified: 2022; **Event:** samplingProtocol: Pitfall traps; eventDate: 2014-06-13; **Record Level:** collectionID: Roma3_5.8
- **Type status:** Other material. **Occurrence:** recordedBy: Fattorini S., Di Giulio A.; individualCount: 1; sex: female; lifeStage: adult; occurrenceID: BC6FBEEB-345E-5829-ABBC-02C808568A48; **Taxon:** scientificName: Arctosapersonata (L. Koch, 1872); order: Araneae; family: Lycosidae; genus: Arctosa; **Location:** country: Italy; countryCode: IT; stateProvince: Rome; county: Rome; municipality: Rome; locality: Appia Antica Regional Park, Rome; locationRemarks: Appia Antica; decimalLatitude: 41.812575; decimalLongitude: 12.564011; geodeticDatum: WGS84; **Identification:** identifiedBy: Tommaso Fusco; dateIdentified: 2022; **Event:** samplingProtocol: Pitfall traps; eventDate: 2013-12-06; **Record Level:** collectionID: Roma3_5.8
- **Type status:** Other material. **Occurrence:** recordedBy: Fattorini S., Di Giulio A.; individualCount: 1; sex: male; lifeStage: adult; occurrenceID: 85EDAE08-EE02-5577-A9C7-0DD08BF695C3; **Taxon:** scientificName: Arctosapersonata (L. Koch, 1872); order: Araneae; family: Lycosidae; genus: Arctosa; **Location:** country: Italy; countryCode: IT; stateProvince: Rome; county: Rome; municipality: Rome; locality: Appia Antica Regional Park, Rome; locationRemarks: Appia Antica; decimalLatitude: 41.812575; decimalLongitude: 12.564011; geodeticDatum: WGS84; **Identification:** identifiedBy: Tommaso Fusco; dateIdentified: 2022; **Event:** samplingProtocol: Pitfall traps; eventDate: 2014-06-13; **Record Level:** collectionID: Roma3_5.8
- **Type status:** Other material. **Occurrence:** recordedBy: Fattorini S., Di Giulio A.; individualCount: 1; sex: male; lifeStage: adult; occurrenceID: C1CAB047-5FF8-523D-96F6-80A212CEBE47; **Taxon:** scientificName: Arctosapersonata (L. Koch, 1872); order: Araneae; family: Lycosidae; genus: Arctosa; **Location:** country: Italy; countryCode: IT; stateProvince: Rome; county: Rome; municipality: Rome; locality: Appia Antica Regional Park, Rome; locationRemarks: Caffarella Centro; decimalLatitude: 41.864889; decimalLongitude: 12.516389; geodeticDatum: WGS84; **Identification:** identifiedBy: Tommaso Fusco; dateIdentified: 2022; **Event:** samplingProtocol: Pitfall traps; eventDate: 2013-11-13; **Record Level:** collectionID: Roma3_5.8
- **Type status:** Other material. **Occurrence:** recordedBy: Fattorini S., Di Giulio A.; individualCount: 1; sex: male; lifeStage: adult; occurrenceID: 0982F77D-AEA2-5A5E-BF30-6458B2341A02; **Taxon:** scientificName: Arctosapersonata (L. Koch, 1872); order: Araneae; family: Lycosidae; genus: Arctosa; **Location:** country: Italy; countryCode: IT; stateProvince: Rome; county: Rome; municipality: Rome; locality: Appia Antica Regional Park, Rome; locationRemarks: Caffarella Sud; decimalLatitude: 41.857247; decimalLongitude: 12.529211; geodeticDatum: WGS84; **Identification:** identifiedBy: Tommaso Fusco; dateIdentified: 2022; **Event:** samplingProtocol: Pitfall traps; eventDate: 2013-11-12; **Record Level:** collectionID: Roma3_5.8
- **Type status:** Other material. **Occurrence:** recordedBy: Fattorini S., Di Giulio A.; individualCount: 2; sex: 1 male, 1 female; lifeStage: adult; occurrenceID: 7C4F53A3-0DF3-55B9-93FE-9CF3A857CC9D; **Taxon:** scientificName: Arctosapersonata (L. Koch, 1872); order: Araneae; family: Lycosidae; genus: Arctosa; **Location:** country: Italy; countryCode: IT; stateProvince: Rome; county: Rome; municipality: Rome; locality: Appia Antica Regional Park, Rome; locationRemarks: Caffarella Sud; decimalLatitude: 41.857247; decimalLongitude: 12.529211; geodeticDatum: WGS84; **Identification:** identifiedBy: Tommaso Fusco; dateIdentified: 2022; **Event:** samplingProtocol: Pitfall traps; eventDate: 2014-05-19; **Record Level:** collectionID: Roma3_5.8
- **Type status:** Other material. **Occurrence:** recordedBy: Fattorini S., Di Giulio A.; individualCount: 1; sex: female; lifeStage: adult; occurrenceID: 0C16769D-DDC6-52CD-AC51-D391CA4629C8; **Taxon:** scientificName: Arctosapersonata (L. Koch, 1872); order: Araneae; family: Lycosidae; genus: Arctosa; **Location:** country: Italy; countryCode: IT; stateProvince: Rome; county: Rome; municipality: Rome; locality: Appia Antica Regional Park, Rome; locationRemarks: Caffarella Sud 2; decimalLatitude: 41.856742; decimalLongitude: 12.529453; geodeticDatum: WGS84; **Identification:** identifiedBy: Tommaso Fusco; dateIdentified: 2022; **Event:** samplingProtocol: Pitfall traps; eventDate: 2014-06-18; **Record Level:** collectionID: Roma3_5.8
- **Type status:** Other material. **Occurrence:** recordedBy: Fattorini S., Di Giulio A.; individualCount: 3; sex: male; lifeStage: adult; occurrenceID: E7C61EFA-91AA-5073-9708-F435B42E27D2; **Taxon:** scientificName: Arctosapersonata (L. Koch, 1872); order: Araneae; family: Lycosidae; genus: Arctosa; **Location:** country: Italy; countryCode: IT; stateProvince: Rome; county: Rome; municipality: Rome; locality: Appia Antica Regional Park, Rome; locationRemarks: Caffarella Sud 3; decimalLatitude: 41.856928; decimalLongitude: 12.528406; geodeticDatum: WGS84; **Identification:** identifiedBy: Tommaso Fusco; dateIdentified: 2022; **Event:** samplingProtocol: Pitfall traps; eventDate: 2014-05-19; **Record Level:** collectionID: Roma3_5.8
- **Type status:** Other material. **Occurrence:** recordedBy: Fattorini S., Di Giulio A.; individualCount: 7; sex: 6 male, 1 female; lifeStage: adult; occurrenceID: 19A24654-A8EC-51DE-8B10-AE352139B276; **Taxon:** scientificName: Arctosapersonata (L. Koch, 1872); order: Araneae; family: Lycosidae; genus: Arctosa; **Location:** country: Italy; countryCode: IT; stateProvince: Rome; county: Rome; municipality: Rome; locality: Appia Antica Regional Park, Rome; locationRemarks: Casal Verbeni; decimalLatitude: 41.815250; decimalLongitude: 12.552222; geodeticDatum: WGS84; **Identification:** identifiedBy: Tommaso Fusco; dateIdentified: 2022; **Event:** samplingProtocol: Pitfall traps; eventDate: 2014-05-26; **Record Level:** collectionID: Roma3_5.8
- **Type status:** Other material. **Occurrence:** recordedBy: Fattorini S., Di Giulio A.; individualCount: 1; sex: female; lifeStage: adult; occurrenceID: 6F03ED80-E0F3-5219-A688-E50746606B77; **Taxon:** scientificName: Arctosapersonata (L. Koch, 1872); order: Araneae; family: Lycosidae; genus: Arctosa; **Location:** country: Italy; countryCode: IT; stateProvince: Rome; county: Rome; municipality: Rome; locality: Appia Antica Regional Park, Rome; locationRemarks: Casal Verbeni; decimalLatitude: 41.815250; decimalLongitude: 12.552222; geodeticDatum: WGS84; **Identification:** identifiedBy: Tommaso Fusco; dateIdentified: 2022; **Event:** samplingProtocol: Pitfall traps; eventDate: 2014-06-13; **Record Level:** collectionID: Roma3_5.8
- **Type status:** Other material. **Occurrence:** recordedBy: Fattorini S., Di Giulio A.; individualCount: 1; sex: female; lifeStage: adult; occurrenceID: 49D79BF0-45E5-5B10-BD86-83A6AC360996; **Taxon:** scientificName: Arctosapersonata (L. Koch, 1872); order: Araneae; family: Lycosidae; genus: Arctosa; **Location:** country: Italy; countryCode: IT; stateProvince: Rome; county: Rome; municipality: Rome; locality: Appia Antica Regional Park, Rome; locationRemarks: Cava Fiorucci; decimalLatitude: 41.834106; decimalLongitude: 12.549264; geodeticDatum: WGS84; **Identification:** identifiedBy: Tommaso Fusco; dateIdentified: 2022; **Event:** samplingProtocol: Pitfall traps; eventDate: 2014-06-24; **Record Level:** collectionID: Roma3_5.8
- **Type status:** Other material. **Occurrence:** recordedBy: Fattorini S., Di Giulio A.; individualCount: 1; sex: male; lifeStage: adult; occurrenceID: E144E328-4219-50E1-8098-FC98ACCD9797; **Taxon:** scientificName: Arctosapersonata (L. Koch, 1872); order: Araneae; family: Lycosidae; genus: Arctosa; **Location:** country: Italy; countryCode: IT; stateProvince: Rome; county: Rome; municipality: Rome; locality: Appia Antica Regional Park, Rome; locationRemarks: Cava Fiorucci; decimalLatitude: 41.834106; decimalLongitude: 12.549264; geodeticDatum: WGS84; **Identification:** identifiedBy: Tommaso Fusco; dateIdentified: 2022; **Event:** samplingProtocol: Pitfall traps; eventDate: 2014-05-26; **Record Level:** collectionID: Roma3_5.8
- **Type status:** Other material. **Occurrence:** recordedBy: Fattorini S., Di Giulio A.; individualCount: 4; sex: 3 male, 1 female; lifeStage: adult; occurrenceID: 370AA4C4-EEF2-5A9D-BBB4-944B8D2AB260; **Taxon:** scientificName: Arctosapersonata (L. Koch, 1872); order: Araneae; family: Lycosidae; genus: Arctosa; **Location:** country: Italy; countryCode: IT; stateProvince: Rome; county: Rome; municipality: Rome; locality: Appia Antica Regional Park, Rome; locationRemarks: Cava Fiorucci; decimalLatitude: 41.834106; decimalLongitude: 12.549264; geodeticDatum: WGS84; **Identification:** identifiedBy: Tommaso Fusco; dateIdentified: 2022; **Event:** samplingProtocol: Pitfall traps; eventDate: 2014-06-13; **Record Level:** collectionID: Roma3_5.8
- **Type status:** Other material. **Occurrence:** recordedBy: Fattorini S., Di Giulio A.; individualCount: 2; sex: 1 male, 1 female; lifeStage: adult; occurrenceID: 4358C3A8-B286-5428-A0E4-F0359DCB58DA; **Taxon:** scientificName: Arctosapersonata (L. Koch, 1872); order: Araneae; family: Lycosidae; genus: Arctosa; **Location:** country: Italy; countryCode: IT; stateProvince: Rome; county: Rome; municipality: Rome; locality: Appia Antica Regional Park, Rome; locationRemarks: San Sebastiano; decimalLatitude: 41.855733; decimalLongitude: 12.515114; geodeticDatum: WGS84; **Identification:** identifiedBy: Tommaso Fusco; dateIdentified: 2022; **Event:** samplingProtocol: Pitfall traps; eventDate: 2014-05-28; **Record Level:** collectionID: Roma3_5.8
- **Type status:** Other material. **Occurrence:** recordedBy: Fattorini S., Di Giulio A.; individualCount: 3; sex: male; lifeStage: adult; occurrenceID: 9A8BC767-CA29-52A9-8341-B7E101273B02; **Taxon:** scientificName: Arctosapersonata (L. Koch, 1872); order: Araneae; family: Lycosidae; genus: Arctosa; **Location:** country: Italy; countryCode: IT; stateProvince: Rome; county: Rome; municipality: Rome; locality: Appia Antica Regional Park, Rome; locationRemarks: San Sebastiano; decimalLatitude: 41.855733; decimalLongitude: 12.515114; geodeticDatum: WGS84; **Identification:** identifiedBy: Tommaso Fusco; dateIdentified: 2022; **Event:** samplingProtocol: Pitfall traps; eventDate: 2014-05-20; **Record Level:** collectionID: Roma3_5.8
- **Type status:** Other material. **Occurrence:** recordedBy: Fattorini S., Di Giulio A.; individualCount: 2; sex: male; lifeStage: adult; occurrenceID: CE2E36D1-1E29-5778-989E-40AEFF85A847; **Taxon:** scientificName: Arctosapersonata (L. Koch, 1872); order: Araneae; family: Lycosidae; genus: Arctosa; **Location:** country: Italy; countryCode: IT; stateProvince: Rome; county: Rome; municipality: Rome; locality: Appia Antica Regional Park, Rome; locationRemarks: San Sebastiano; decimalLatitude: 41.855733; decimalLongitude: 12.515114; geodeticDatum: WGS84; **Identification:** identifiedBy: Tommaso Fusco; dateIdentified: 2022; **Event:** samplingProtocol: Pitfall traps; eventDate: 2014-06-09; **Record Level:** collectionID: Roma3_5.8
- **Type status:** Other material. **Occurrence:** recordedBy: Fattorini S., Di Giulio A.; individualCount: 2; sex: 1 male, 1 female; lifeStage: adult; occurrenceID: E5AEEA2D-FE81-523B-95A9-AF63FB80D5D9; **Taxon:** scientificName: Arctosapersonata (L. Koch, 1872); order: Araneae; family: Lycosidae; genus: Arctosa; **Location:** country: Italy; countryCode: IT; stateProvince: Rome; county: Rome; municipality: Rome; locality: Appia Antica Regional Park, Rome; locationRemarks: Tor Marancia; decimalLatitude: 41.850308; decimalLongitude: 12.503178; geodeticDatum: WGS84; **Identification:** identifiedBy: Tommaso Fusco; dateIdentified: 2022; **Event:** samplingProtocol: Pitfall traps; eventDate: 2014-05-27; **Record Level:** collectionID: Roma3_5.8
- **Type status:** Other material. **Occurrence:** recordedBy: Fattorini S., Di Giulio A.; individualCount: 11; sex: male; lifeStage: adult; occurrenceID: 928C6729-CE2C-556A-A006-5F2D31D773DF; **Taxon:** scientificName: Arctosapersonata (L. Koch, 1872); order: Araneae; family: Lycosidae; genus: Arctosa; **Location:** country: Italy; countryCode: IT; stateProvince: Rome; county: Rome; municipality: Rome; locality: Appia Antica Regional Park, Rome; locationRemarks: Torre Selce; decimalLatitude: 41.816611; decimalLongitude: 12.560667; geodeticDatum: WGS84; **Identification:** identifiedBy: Tommaso Fusco; dateIdentified: 2022; **Event:** samplingProtocol: Pitfall traps; eventDate: 2014-05-26; **Record Level:** collectionID: Roma3_5.8
- **Type status:** Other material. **Occurrence:** recordedBy: Fattorini S., Di Giulio A.; individualCount: 1; sex: male; lifeStage: adult; occurrenceID: BB83B31C-D57C-5FC5-9E07-EC137D6F5F62; **Taxon:** scientificName: Arctosapersonata (L. Koch, 1872); order: Araneae; family: Lycosidae; genus: Arctosa; **Location:** country: Italy; countryCode: IT; stateProvince: Rome; county: Rome; municipality: Rome; locality: Appia Antica Regional Park, Rome; locationRemarks: Torre Selce; decimalLatitude: 41.816611; decimalLongitude: 12.560667; geodeticDatum: WGS84; **Identification:** identifiedBy: Tommaso Fusco; dateIdentified: 2022; **Event:** samplingProtocol: Pitfall traps; eventDate: 2014-06-24; **Record Level:** collectionID: Roma3_5.8
- **Type status:** Other material. **Occurrence:** recordedBy: Fattorini S., Di Giulio A.; individualCount: 5; sex: male; lifeStage: adult; occurrenceID: 15E92E0A-6703-5F48-A522-25B209D5A27B; **Taxon:** scientificName: Arctosapersonata (L. Koch, 1872); order: Araneae; family: Lycosidae; genus: Arctosa; **Location:** country: Italy; countryCode: IT; stateProvince: Rome; county: Rome; municipality: Rome; locality: Appia Antica Regional Park, Rome; locationRemarks: Torre Selce; decimalLatitude: 41.816611; decimalLongitude: 12.560667; geodeticDatum: WGS84; **Identification:** identifiedBy: Tommaso Fusco; dateIdentified: 2022; **Event:** samplingProtocol: Pitfall traps; eventDate: 2014-06-05; **Record Level:** collectionID: Roma3_5.8
- **Type status:** Other material. **Occurrence:** recordedBy: Fattorini S., Di Giulio A.; individualCount: 1; sex: male; lifeStage: adult; occurrenceID: CD846C75-3D49-5364-B1AF-27F67BE14844; **Taxon:** scientificName: Arctosapersonata (L. Koch, 1872); order: Araneae; family: Lycosidae; genus: Arctosa; **Location:** country: Italy; countryCode: IT; stateProvince: Rome; county: Rome; municipality: Rome; locality: Appia Antica Regional Park, Rome; locationRemarks: Torre Selce; decimalLatitude: 41.816611; decimalLongitude: 12.560667; geodeticDatum: WGS84; **Identification:** identifiedBy: Tommaso Fusco; dateIdentified: 2022; **Event:** samplingProtocol: Pitfall traps; eventDate: 2014-06-13; **Record Level:** collectionID: Roma3_5.8
- **Type status:** Other material. **Occurrence:** recordedBy: Fattorini S., Di Giulio A.; individualCount: 1; sex: female; lifeStage: adult; occurrenceID: 82DF5251-ACC9-523E-BEA8-77BC842B8CE6; **Taxon:** scientificName: Arctosapersonata (L. Koch, 1872); order: Araneae; family: Lycosidae; genus: Arctosa; **Location:** country: Italy; countryCode: IT; stateProvince: Rome; county: Rome; municipality: Rome; locality: Appia Antica Regional Park, Rome; locationRemarks: Torre Selce; decimalLatitude: 41.816611; decimalLongitude: 12.560667; geodeticDatum: WGS84; **Identification:** identifiedBy: Tommaso Fusco; dateIdentified: 2022; **Event:** samplingProtocol: Pitfall traps; eventDate: 2013-11-07; **Record Level:** collectionID: Roma3_5.8

##### Distribution

Western Mediterranean to Slovenia. Mediterranean (MED) chorotype.

#### Aulonia albimana

(Walckenaer, 1805)

D589FE49-2FE9-538A-A5DE-B72E67AF0BC3

##### Materials

- **Type status:** Other material. **Occurrence:** recordedBy: Fattorini S., Di Giulio A.; individualCount: 2; sex: male; lifeStage: adult; occurrenceID: 942D1BAA-B8FA-5C47-A67C-AB2BB4B7421C; **Taxon:** scientificName: Auloniaalbimana (Walckenaer, 1805); order: Araneae; family: Lycosidae; genus: Aulonia; **Location:** country: Italy; countryCode: IT; stateProvince: Rome; county: Rome; municipality: Rome; locality: Appia Antica Regional Park, Rome; locationRemarks: Tor marancia; decimalLatitude: 41.850308; decimalLongitude: 12.503178; geodeticDatum: WGS84; **Identification:** identifiedBy: Tommaso Fusco; dateIdentified: 2022; **Event:** samplingProtocol: Pitfall traps; eventDate: 2014-05-26; **Record Level:** collectionID: Roma3_5.8
- **Type status:** Other material. **Occurrence:** recordedBy: Fattorini S., Di Giulio A.; individualCount: 2; sex: male; lifeStage: adult; occurrenceID: E22B1E9E-1D4E-561D-BE2B-CB11B39B4790; **Taxon:** scientificName: Auloniaalbimana (Walckenaer, 1805); order: Araneae; family: Lycosidae; genus: Aulonia; **Location:** country: Italy; countryCode: IT; stateProvince: Rome; county: Rome; municipality: Rome; locality: Appia Antica Regional Park, Rome; locationRemarks: Tor Marancia; decimalLatitude: 41.850308; decimalLongitude: 12.503178; geodeticDatum: WGS84; **Identification:** identifiedBy: Tommaso Fusco; dateIdentified: 2022; **Event:** samplingProtocol: Pitfall traps; eventDate: 2014-06-05; **Record Level:** collectionID: Roma3_5.8
- **Type status:** Other material. **Occurrence:** recordedBy: Fattorini S., Di Giulio A.; individualCount: 1; sex: male; lifeStage: adult; occurrenceID: 8A97B168-F007-53C3-9EA8-E8BF57585769; **Taxon:** scientificName: Auloniaalbimana (Walckenaer, 1805); order: Araneae; family: Lycosidae; genus: Aulonia; **Location:** country: Italy; countryCode: IT; stateProvince: Rome; county: Rome; municipality: Rome; locality: Appia Antica Regional Park, Rome; locationRemarks: Acqua Santa; decimalLatitude: 41.850561; decimalLongitude: 12.530861; geodeticDatum: WGS84; **Identification:** identifiedBy: Tommaso Fusco; dateIdentified: 2022; **Event:** samplingProtocol: Pitfall traps; eventDate: 2014-06-09; **Record Level:** collectionID: Roma3_5.8
- **Type status:** Other material. **Occurrence:** recordedBy: Fattorini S., Di Giulio A.; individualCount: 2; sex: male; lifeStage: adult; occurrenceID: 45FB97F3-6A9C-5E3A-958B-FB4D5EBEA503; **Taxon:** scientificName: Auloniaalbimana (Walckenaer, 1805); order: Araneae; family: Lycosidae; genus: Aulonia; **Location:** country: Italy; countryCode: IT; stateProvince: Rome; county: Rome; municipality: Rome; locality: Appia Antica Regional Park, Rome; locationRemarks: Tor Marancia; decimalLatitude: 41.850308; decimalLongitude: 12.503178; geodeticDatum: WGS84; **Identification:** identifiedBy: Tommaso Fusco; dateIdentified: 2022; **Event:** samplingProtocol: Pitfall traps; eventDate: 2014-06-17; **Record Level:** collectionID: Roma3_5.8
- **Type status:** Other material. **Occurrence:** recordedBy: Fattorini S., Di Giulio A.; individualCount: 4; sex: male; lifeStage: adult; occurrenceID: 9B072D2D-608D-5759-876A-E825C9FB870C; **Taxon:** scientificName: Auloniaalbimana (Walckenaer, 1805); order: Araneae; family: Lycosidae; genus: Aulonia; **Location:** country: Italy; countryCode: IT; stateProvince: Rome; county: Rome; municipality: Rome; locality: Appia Antica Regional Park, Rome; locationRemarks: Tor Marancia; decimalLatitude: 41.850308; decimalLongitude: 12.503178; geodeticDatum: WGS84; **Identification:** identifiedBy: Tommaso Fusco; dateIdentified: 2022; **Event:** samplingProtocol: Pitfall traps; eventDate: 2014-05-15; **Record Level:** collectionID: Roma3_5.8

##### Distribution

Europe, Turkey, Caucasus, Egypt. Europeo-Mediterranean (EUM) chorotype.

#### Pardosa prativaga

(L. Koch, 1870)

7BB6791A-FA48-53FA-B568-0F33BA57FA39

##### Materials

- **Type status:** Other material. **Occurrence:** recordedBy: Fattorini S., Di Giulio A.; individualCount: 4; sex: male; lifeStage: adult; occurrenceID: 14D724F8-78E3-5DE3-8328-1AC87E114280; **Taxon:** scientificName: Pardosaprativaga (L. Koch, 1870); order: Araneae; family: Lycosidae; genus: Pardosa; **Location:** country: Italy; countryCode: IT; stateProvince: Rome; county: Rome; municipality: Rome; locality: Appia Antica Regional Park, Rome; locationRemarks: Tor Marancia; decimalLatitude: 41.850308; decimalLongitude: 12.503178; geodeticDatum: WGS84; **Identification:** identifiedBy: Tommaso Fusco; dateIdentified: 2022; **Event:** samplingProtocol: Pitfall traps; eventDate: 2014-05-15; **Record Level:** collectionID: Roma3_5.8

##### Distribution

Europe, Turkey, Caucasus, Russia (Europe to south Siberia), Central Asia. Sibero-European (SIE) chorotype.

#### Pardosa proxima

(C. L. Koch, 1847)

FFA2DA3F-43DF-59D3-8804-ECDA8319B1A4

##### Materials

- **Type status:** Other material. **Occurrence:** recordedBy: Fattorini S., Di Giulio A.; individualCount: 17; sex: 15 male, 2 female; lifeStage: adult; occurrenceID: 8E39ECD6-9B3B-5F6A-8955-23048A075F4C; **Taxon:** scientificName: Pardosaproxima (C. L. Koch, 1847); order: Araneae; family: Lycosidae; genus: Pardosa; **Location:** country: Italy; countryCode: IT; stateProvince: Rome; county: Rome; municipality: Rome; locality: Appia Antica Regional Park, Rome; locationRemarks: Acqua Santa; decimalLatitude: 41.850561; decimalLongitude: 12.530861; geodeticDatum: WGS84; **Identification:** identifiedBy: Tommaso Fusco; dateIdentified: 2022; **Event:** samplingProtocol: Pitfall traps; eventDate: 2014-06-19; **Record Level:** collectionID: Roma3_5.8
- **Type status:** Other material. **Occurrence:** recordedBy: Fattorini S., Di Giulio A.; individualCount: 1; sex: female; lifeStage: adult; occurrenceID: 30777060-7BE4-5042-9664-DC0D264A80A7; **Taxon:** scientificName: Pardosaproxima (C. L. Koch, 1847); order: Araneae; family: Lycosidae; genus: Pardosa; **Location:** country: Italy; countryCode: IT; stateProvince: Rome; county: Rome; municipality: Rome; locality: Appia Antica Regional Park, Rome; locationRemarks: Acqua Santa; decimalLatitude: 41.850561; decimalLongitude: 12.530861; geodeticDatum: WGS84; **Identification:** identifiedBy: Tommaso Fusco; dateIdentified: 2022; **Event:** samplingProtocol: Pitfall traps; eventDate: 2014-06-09; **Record Level:** collectionID: Roma3_5.8
- **Type status:** Other material. **Occurrence:** recordedBy: Fattorini S., Di Giulio A.; individualCount: 2; sex: female; lifeStage: adult; occurrenceID: 4BF22FA3-4A72-5BC9-B089-2EC28C4FA47F; **Taxon:** scientificName: Pardosaproxima (C. L. Koch, 1847); order: Araneae; family: Lycosidae; genus: Pardosa; **Location:** country: Italy; countryCode: IT; stateProvince: Rome; county: Rome; municipality: Rome; locality: Appia Antica Regional Park, Rome; locationRemarks: Caffarella Nord; decimalLatitude: 41.867753; decimalLongitude: 12.512414; geodeticDatum: WGS84; **Identification:** identifiedBy: Tommaso Fusco; dateIdentified: 2022; **Event:** samplingProtocol: Pitfall traps; eventDate: 2014-06-18; **Record Level:** collectionID: Roma3_5.8
- **Type status:** Other material. **Occurrence:** recordedBy: Fattorini S., Di Giulio A.; individualCount: 1; sex: male; lifeStage: adult; occurrenceID: 7B3F9C59-80BB-5F9F-A00D-75625D8B6EB6; **Taxon:** scientificName: Pardosaproxima (C. L. Koch, 1847); order: Araneae; family: Lycosidae; genus: Pardosa; **Location:** country: Italy; countryCode: IT; stateProvince: Rome; county: Rome; municipality: Rome; locality: Appia Antica Regional Park, Rome; locationRemarks: Caffarella Sud; decimalLatitude: 41.857247; decimalLongitude: 12.529211; geodeticDatum: WGS84; **Identification:** identifiedBy: Tommaso Fusco; dateIdentified: 2022; **Event:** samplingProtocol: Pitfall traps; eventDate: 2014-06-18; **Record Level:** collectionID: Roma3_5.8
- **Type status:** Other material. **Occurrence:** recordedBy: Fattorini S., Di Giulio A.; individualCount: 1; sex: male; lifeStage: adult; occurrenceID: CAF9B5C6-841E-51D3-9157-F476A92DC838; **Taxon:** scientificName: Pardosaproxima (C. L. Koch, 1847); order: Araneae; family: Lycosidae; genus: Pardosa; **Location:** country: Italy; countryCode: IT; stateProvince: Rome; county: Rome; municipality: Rome; locality: Appia Antica Regional Park, Rome; locationRemarks: Casal Verbeni; decimalLatitude: 41.815250; decimalLongitude: 12.552222; geodeticDatum: WGS84; **Identification:** identifiedBy: Tommaso Fusco; dateIdentified: 2022; **Event:** samplingProtocol: Pitfall traps; eventDate: 2014-06-05; **Record Level:** collectionID: Roma3_5.8
- **Type status:** Other material. **Occurrence:** recordedBy: Fattorini S., Di Giulio A.; individualCount: 6; sex: 4 male, 2 female; lifeStage: adult; occurrenceID: 6D5C1432-8B2B-54D1-847A-EE53270C689B; **Taxon:** scientificName: Pardosaproxima (C. L. Koch, 1847); order: Araneae; family: Lycosidae; genus: Pardosa; **Location:** country: Italy; countryCode: IT; stateProvince: Rome; county: Rome; municipality: Rome; locality: Appia Antica Regional Park, Rome; locationRemarks: Cava Fiorucci; decimalLatitude: 41.834106; decimalLongitude: 12.549264; geodeticDatum: WGS84; **Identification:** identifiedBy: Tommaso Fusco; dateIdentified: 2022; **Event:** samplingProtocol: Pitfall traps; eventDate: 2014-06-13; **Record Level:** collectionID: Roma3_5.8
- **Type status:** Other material. **Occurrence:** recordedBy: Fattorini S., Di Giulio A.; individualCount: 5; sex: male; lifeStage: adult; occurrenceID: 3ACA5847-55B0-5CB7-8F73-42F5C95FB54B; **Taxon:** scientificName: Pardosaproxima (C. L. Koch, 1847); order: Araneae; family: Lycosidae; genus: Pardosa; **Location:** country: Italy; countryCode: IT; stateProvince: Rome; county: Rome; municipality: Rome; locality: Appia Antica Regional Park, Rome; locationRemarks: Cava Fiorucci; decimalLatitude: 41.834106; decimalLongitude: 12.549264; geodeticDatum: WGS84; **Identification:** identifiedBy: Tommaso Fusco; dateIdentified: 2022; **Event:** samplingProtocol: Pitfall traps; eventDate: 2014-06-24; **Record Level:** collectionID: Roma3_5.8
- **Type status:** Other material. **Occurrence:** recordedBy: Fattorini S., Di Giulio A.; individualCount: 3; sex: male; lifeStage: adult; occurrenceID: A2DDBB4B-2859-538D-B16A-1BFE234C286B; **Taxon:** scientificName: Pardosaproxima (C. L. Koch, 1847); order: Araneae; family: Lycosidae; genus: Pardosa; **Location:** country: Italy; countryCode: IT; stateProvince: Rome; county: Rome; municipality: Rome; locality: Appia Antica Regional Park, Rome; locationRemarks: Farnesiana; decimalLatitude: 41.839667; decimalLongitude: 12.525528; geodeticDatum: WGS84; **Identification:** identifiedBy: Tommaso Fusco; dateIdentified: 2022; **Event:** samplingProtocol: Pitfall traps; eventDate: 2014-06-19; **Record Level:** collectionID: Roma3_5.8
- **Type status:** Other material. **Occurrence:** recordedBy: Fattorini S., Di Giulio A.; individualCount: 1; sex: female; lifeStage: adult; occurrenceID: 719A264B-B6AB-5DD5-874D-23C6FBE4AF16; **Taxon:** scientificName: Pardosaproxima (C. L. Koch, 1847); order: Araneae; family: Lycosidae; genus: Pardosa; **Location:** country: Italy; countryCode: IT; stateProvince: Rome; county: Rome; municipality: Rome; locality: Appia Antica Regional Park, Rome; locationRemarks: Farnesiana; decimalLatitude: 41.839667; decimalLongitude: 12.525528; geodeticDatum: WGS84; **Identification:** identifiedBy: Tommaso Fusco; dateIdentified: 2022; **Event:** samplingProtocol: Pitfall traps; eventDate: 2014-05-28; **Record Level:** collectionID: Roma3_5.8
- **Type status:** Other material. **Occurrence:** recordedBy: Fattorini S., Di Giulio A.; individualCount: 1; sex: female; lifeStage: adult; occurrenceID: 7D4A62ED-33AA-5FC3-A3C6-E38526392C1D; **Taxon:** scientificName: Pardosaproxima (C. L. Koch, 1847); order: Araneae; family: Lycosidae; genus: Pardosa; **Location:** country: Italy; countryCode: IT; stateProvince: Rome; county: Rome; municipality: Rome; locality: Appia Antica Regional Park, Rome; locationRemarks: Tor Marancia; decimalLatitude: 41.850308; decimalLongitude: 12.503178; geodeticDatum: WGS84; **Identification:** identifiedBy: Tommaso Fusco; dateIdentified: 2022; **Event:** samplingProtocol: Pitfall traps; eventDate: 2014-05-27; **Record Level:** collectionID: Roma3_5.8
- **Type status:** Other material. **Occurrence:** recordedBy: Fattorini S., Di Giulio A.; individualCount: 1; sex: male; lifeStage: adult; occurrenceID: 9B46236E-CCF5-571B-BF23-87797439F834; **Taxon:** scientificName: Pardosaproxima (C. L. Koch, 1847); order: Araneae; family: Lycosidae; genus: Pardosa; **Location:** country: Italy; countryCode: IT; stateProvince: Rome; county: Rome; municipality: Rome; locality: Appia Antica Regional Park, Rome; locationRemarks: Tor Marancia; decimalLatitude: 41.850308; decimalLongitude: 12.503178; geodeticDatum: WGS84; **Identification:** identifiedBy: Tommaso Fusco; dateIdentified: 2022; **Event:** samplingProtocol: Pitfall traps; eventDate: 2014-06-05; **Record Level:** collectionID: Roma3_5.8
- **Type status:** Other material. **Occurrence:** recordedBy: Fattorini S., Di Giulio A.; individualCount: 1; sex: male; lifeStage: adult; occurrenceID: C0DE6200-E3EC-51A7-955B-2C3E8078949F; **Taxon:** scientificName: Pardosaproxima (C. L. Koch, 1847); order: Araneae; family: Lycosidae; genus: Pardosa; **Location:** country: Italy; countryCode: IT; stateProvince: Rome; county: Rome; municipality: Rome; locality: Appia Antica Regional Park, Rome; locationRemarks: Tor Marancia; decimalLatitude: 41.850308; decimalLongitude: 12.503178; geodeticDatum: WGS84; **Identification:** identifiedBy: Tommaso Fusco; dateIdentified: 2022; **Event:** samplingProtocol: Pitfall traps; eventDate: 2014-06-17; **Record Level:** collectionID: Roma3_5.8

##### Distribution

Macaronesia, northern Africa, Europe, Caucasus, Russia (Europe to Far East), Kazakhstan, Iran, Central Asia, China. Palaearctic (PAL) chorotype.

#### Trochosa hispanica

Simon, 1870

05249E58-3FD2-5EF4-B1B5-35F7941E5DF1

##### Materials

- **Type status:** Other material. **Occurrence:** recordedBy: Fattorini S., Di Giulio A.; individualCount: 7; sex: 6 male, 1 female; lifeStage: adult; occurrenceID: E665EA92-5FBD-509F-A61A-41BB022641C5; **Taxon:** scientificName: Trochosahispanica Simon, 1870; order: Araneae; family: Lycosidae; genus: Trochosa; **Location:** country: Italy; countryCode: IT; stateProvince: Rome; county: Rome; municipality: Rome; locality: Appia Antica Regional Park, Rome; locationRemarks: Acqua Santa; decimalLatitude: 41.850561; decimalLongitude: 12.530861; geodeticDatum: WGS84; **Identification:** identifiedBy: Tommaso Fusco; dateIdentified: 2022; **Event:** samplingProtocol: Pitfall traps; eventDate: 2014-05-20; **Record Level:** collectionID: Roma3_5.8
- **Type status:** Other material. **Occurrence:** recordedBy: Fattorini S., Di Giulio A.; individualCount: 7; sex: 5 male, 2 female; lifeStage: adult; occurrenceID: 156D27C3-3C9D-5F11-B6A7-76DC40C5AF17; **Taxon:** scientificName: Trochosahispanica Simon, 1870; order: Araneae; family: Lycosidae; genus: Trochosa; **Location:** country: Italy; countryCode: IT; stateProvince: Rome; county: Rome; municipality: Rome; locality: Appia Antica Regional Park, Rome; locationRemarks: Acqua Santa; decimalLatitude: 41.850561; decimalLongitude: 12.530861; geodeticDatum: WGS84; **Identification:** identifiedBy: Tommaso Fusco; dateIdentified: 2022; **Event:** samplingProtocol: Pitfall traps; eventDate: 2014-05-28; **Record Level:** collectionID: Roma3_5.8
- **Type status:** Other material. **Occurrence:** recordedBy: Fattorini S., Di Giulio A.; individualCount: 18; sex: 14 male, 4 female; lifeStage: adult; occurrenceID: 5CC3E061-B4B6-5864-92F4-093BD44CC248; **Taxon:** scientificName: Trochosahispanica Simon, 1870; order: Araneae; family: Lycosidae; genus: Trochosa; **Location:** country: Italy; countryCode: IT; stateProvince: Rome; county: Rome; municipality: Rome; locality: Appia Antica Regional Park, Rome; locationRemarks: Acqua Santa; decimalLatitude: 41.850561; decimalLongitude: 12.530861; geodeticDatum: WGS84; **Identification:** identifiedBy: Tommaso Fusco; dateIdentified: 2022; **Event:** samplingProtocol: Pitfall traps; eventDate: 2014-06-09; **Record Level:** collectionID: Roma3_5.8
- **Type status:** Other material. **Occurrence:** recordedBy: Fattorini S., Di Giulio A.; individualCount: 23; sex: 20 male, 3 female; lifeStage: adult; occurrenceID: 6A76341A-7B0A-5CF5-A507-7F2B3961C9A9; **Taxon:** scientificName: Trochosahispanica Simon, 1870; order: Araneae; family: Lycosidae; genus: Trochosa; **Location:** country: Italy; countryCode: IT; stateProvince: Rome; county: Rome; municipality: Rome; locality: Appia Antica Regional Park, Rome; locationRemarks: Acqua Santa; decimalLatitude: 41.850561; decimalLongitude: 12.530861; geodeticDatum: WGS84; **Identification:** identifiedBy: Tommaso Fusco; dateIdentified: 2022; **Event:** samplingProtocol: Pitfall traps; eventDate: 2014-06-19; **Record Level:** collectionID: Roma3_5.8
- **Type status:** Other material. **Occurrence:** recordedBy: Fattorini S., Di Giulio A.; individualCount: 3; sex: 2 male, 1 female; lifeStage: adult; occurrenceID: B65F4DF3-29F8-5FBF-8D2D-C6122884D60E; **Taxon:** scientificName: Trochosahispanica Simon, 1870; order: Araneae; family: Lycosidae; genus: Trochosa; **Location:** country: Italy; countryCode: IT; stateProvince: Rome; county: Rome; municipality: Rome; locality: Appia Antica Regional Park, Rome; locationRemarks: Appia Antica; decimalLatitude: 41.812575; decimalLongitude: 12.564011; geodeticDatum: WGS84; **Identification:** identifiedBy: Tommaso Fusco; dateIdentified: 2022; **Event:** samplingProtocol: Pitfall traps; eventDate: 2014-06-13; **Record Level:** collectionID: Roma3_5.8
- **Type status:** Other material. **Occurrence:** recordedBy: Fattorini S., Di Giulio A.; individualCount: 15; sex: 14 male, 1 female; lifeStage: adult; occurrenceID: E6A9363B-AFDF-55C5-88F0-A08D99AE3F09; **Taxon:** scientificName: Trochosahispanica Simon, 1870; order: Araneae; family: Lycosidae; genus: Trochosa; **Location:** country: Italy; countryCode: IT; stateProvince: Rome; county: Rome; municipality: Rome; locality: Appia Antica Regional Park, Rome; locationRemarks: Caffarella Centro; decimalLatitude: 41.864889; decimalLongitude: 12.516389; geodeticDatum: WGS84; **Identification:** identifiedBy: Tommaso Fusco; dateIdentified: 2022; **Event:** samplingProtocol: Pitfall traps; eventDate: 2014-05-19; **Record Level:** collectionID: Roma3_5.8
- **Type status:** Other material. **Occurrence:** recordedBy: Fattorini S., Di Giulio A.; individualCount: 4; sex: 1 male, 3 female; lifeStage: adult; occurrenceID: C8988CDF-2EC2-59D3-94EA-22A853382C72; **Taxon:** scientificName: Trochosahispanica Simon, 1870; order: Araneae; family: Lycosidae; genus: Trochosa; **Location:** country: Italy; countryCode: IT; stateProvince: Rome; county: Rome; municipality: Rome; locality: Appia Antica Regional Park, Rome; locationRemarks: Caffarella Centro; decimalLatitude: 41.864889; decimalLongitude: 12.516389; geodeticDatum: WGS84; **Identification:** identifiedBy: Tommaso Fusco; dateIdentified: 2022; **Event:** samplingProtocol: Pitfall traps; eventDate: 2014-05-27; **Record Level:** collectionID: Roma3_5.8
- **Type status:** Other material. **Occurrence:** recordedBy: Fattorini S., Di Giulio A.; individualCount: 5; sex: 4 male, 1 female; lifeStage: adult; occurrenceID: 84312CCF-7395-58CF-9B3F-1852DD12DE53; **Taxon:** scientificName: Trochosahispanica Simon, 1870; order: Araneae; family: Lycosidae; genus: Trochosa; **Location:** country: Italy; countryCode: IT; stateProvince: Rome; county: Rome; municipality: Rome; locality: Appia Antica Regional Park, Rome; locationRemarks: Caffarella Centro; decimalLatitude: 41.864889; decimalLongitude: 12.516389; geodeticDatum: WGS84; **Identification:** identifiedBy: Tommaso Fusco; dateIdentified: 2022; **Event:** samplingProtocol: Pitfall traps; eventDate: 2014-06-06; **Record Level:** collectionID: Roma3_5.8
- **Type status:** Other material. **Occurrence:** recordedBy: Fattorini S., Di Giulio A.; individualCount: 4; sex: 3 male, 1 female; lifeStage: adult; occurrenceID: F8A38A8C-4C72-5B43-8A7E-7B12DE8F6871; **Taxon:** scientificName: Trochosahispanica Simon, 1870; order: Araneae; family: Lycosidae; genus: Trochosa; **Location:** country: Italy; countryCode: IT; stateProvince: Rome; county: Rome; municipality: Rome; locality: Appia Antica Regional Park, Rome; locationRemarks: Caffarella Centro; decimalLatitude: 41.864889; decimalLongitude: 12.516389; geodeticDatum: WGS84; **Identification:** identifiedBy: Tommaso Fusco; dateIdentified: 2022; **Event:** samplingProtocol: Pitfall traps; eventDate: 2014-06-18; **Record Level:** collectionID: Roma3_5.8
- **Type status:** Other material. **Occurrence:** recordedBy: Fattorini S., Di Giulio A.; individualCount: 8; sex: 7 male, 1 female; lifeStage: adult; occurrenceID: 5ACBD112-3E3F-5E23-9BBD-5967FFA27727; **Taxon:** scientificName: Trochosahispanica Simon, 1870; order: Araneae; family: Lycosidae; genus: Trochosa; **Location:** country: Italy; countryCode: IT; stateProvince: Rome; county: Rome; municipality: Rome; locality: Appia Antica Regional Park, Rome; locationRemarks: Caffarella Centro; decimalLatitude: 41.864889; decimalLongitude: 12.516389; geodeticDatum: WGS84; **Identification:** identifiedBy: Tommaso Fusco; dateIdentified: 2022; **Event:** samplingProtocol: Pitfall traps; eventDate: 2014-05-27; **Record Level:** collectionID: Roma3_5.8
- **Type status:** Other material. **Occurrence:** recordedBy: Fattorini S., Di Giulio A.; individualCount: 5; sex: male; lifeStage: adult; occurrenceID: BF13B7A8-2CE7-54F3-B161-90A3237C276F; **Taxon:** scientificName: Trochosahispanica Simon, 1870; order: Araneae; family: Lycosidae; genus: Trochosa; **Location:** country: Italy; countryCode: IT; stateProvince: Rome; county: Rome; municipality: Rome; locality: Appia Antica Regional Park, Rome; locationRemarks: Caffarella Nord; decimalLatitude: 41.867753; decimalLongitude: 12.512414; geodeticDatum: WGS84; **Identification:** identifiedBy: Tommaso Fusco; dateIdentified: 2022; **Event:** samplingProtocol: Pitfall traps; eventDate: 2014-05-19; **Record Level:** collectionID: Roma3_5.8
- **Type status:** Other material. **Occurrence:** recordedBy: Fattorini S., Di Giulio A.; individualCount: 5; sex: 4 male, 1 female; lifeStage: adult; occurrenceID: 3DB96FEB-0FD6-539B-ACD5-7DF7A30F8B65; **Taxon:** scientificName: Trochosahispanica Simon, 1870; order: Araneae; family: Lycosidae; genus: Trochosa; **Location:** country: Italy; countryCode: IT; stateProvince: Rome; county: Rome; municipality: Rome; locality: Appia Antica Regional Park, Rome; locationRemarks: Caffarella Nord; decimalLatitude: 41.867753; decimalLongitude: 12.512414; geodeticDatum: WGS84; **Identification:** identifiedBy: Tommaso Fusco; dateIdentified: 2022; **Event:** samplingProtocol: Pitfall traps; eventDate: 2014-05-27; **Record Level:** collectionID: Roma3_5.8
- **Type status:** Other material. **Occurrence:** recordedBy: Fattorini S., Di Giulio A.; individualCount: 18; sex: 13 male, 5 female; lifeStage: adult; occurrenceID: 33FD1DCA-83EA-56F9-B6DB-C6A905917DA5; **Taxon:** scientificName: Trochosahispanica Simon, 1870; order: Araneae; family: Lycosidae; genus: Trochosa; **Location:** country: Italy; countryCode: IT; stateProvince: Rome; county: Rome; municipality: Rome; locality: Appia Antica Regional Park, Rome; locationRemarks: Caffarella Nord; decimalLatitude: 41.867753; decimalLongitude: 12.512414; geodeticDatum: WGS84; **Identification:** identifiedBy: Tommaso Fusco; dateIdentified: 2022; **Event:** samplingProtocol: Pitfall traps; eventDate: 2014-06-06; **Record Level:** collectionID: Roma3_5.8
- **Type status:** Other material. **Occurrence:** recordedBy: Fattorini S., Di Giulio A.; individualCount: 18; sex: 13 male, 5 female; lifeStage: adult; occurrenceID: C74F9F0B-9805-5271-AE1E-645EB3BB8343; **Taxon:** scientificName: Trochosahispanica Simon, 1870; order: Araneae; family: Lycosidae; genus: Trochosa; **Location:** country: Italy; countryCode: IT; stateProvince: Rome; county: Rome; municipality: Rome; locality: Appia Antica Regional Park, Rome; locationRemarks: Caffarella Nord; decimalLatitude: 41.867753; decimalLongitude: 12.512414; geodeticDatum: WGS84; **Identification:** identifiedBy: Tommaso Fusco; dateIdentified: 2022; **Event:** samplingProtocol: Pitfall traps; eventDate: 2014-06-18; **Record Level:** collectionID: Roma3_5.8
- **Type status:** Other material. **Occurrence:** recordedBy: Fattorini S., Di Giulio A.; individualCount: 3; sex: 1 male, 2 female; lifeStage: adult; occurrenceID: 3848E91D-6F92-5560-861A-529F2526AD15; **Taxon:** scientificName: Trochosahispanica Simon, 1870; order: Araneae; family: Lycosidae; genus: Trochosa; **Location:** country: Italy; countryCode: IT; stateProvince: Rome; county: Rome; municipality: Rome; locality: Appia Antica Regional Park, Rome; locationRemarks: Caffarella Sud; decimalLatitude: 41.857247; decimalLongitude: 12.529211; geodeticDatum: WGS84; **Identification:** identifiedBy: Tommaso Fusco; dateIdentified: 2022; **Event:** samplingProtocol: Pitfall traps; eventDate: 2013-11-12; **Record Level:** collectionID: Roma3_5.8
- **Type status:** Other material. **Occurrence:** recordedBy: Fattorini S., Di Giulio A.; individualCount: 1; sex: male; lifeStage: adult; occurrenceID: 86F8C27D-B1C1-5EF2-964D-B58B1442BA7B; **Taxon:** scientificName: Trochosahispanica Simon, 1870; order: Araneae; family: Lycosidae; genus: Trochosa; **Location:** country: Italy; countryCode: IT; stateProvince: Rome; county: Rome; municipality: Rome; locality: Appia Antica Regional Park, Rome; locationRemarks: Caffarella Sud; decimalLatitude: 41.857247; decimalLongitude: 12.529211; geodeticDatum: WGS84; **Identification:** identifiedBy: Tommaso Fusco; dateIdentified: 2022; **Event:** samplingProtocol: Pitfall traps; eventDate: 2013-11-29; **Record Level:** collectionID: Roma3_5.8
- **Type status:** Other material. **Occurrence:** recordedBy: Fattorini S., Di Giulio A.; individualCount: 3; sex: male; lifeStage: adult; occurrenceID: CB8C8773-E641-5FC9-80E5-6323E3CCE66E; **Taxon:** scientificName: Trochosahispanica Simon, 1870; order: Araneae; family: Lycosidae; genus: Trochosa; **Location:** country: Italy; countryCode: IT; stateProvince: Rome; county: Rome; municipality: Rome; locality: Appia Antica Regional Park, Rome; locationRemarks: Caffarella Sud; decimalLatitude: 41.857247; decimalLongitude: 12.529211; geodeticDatum: WGS84; **Identification:** identifiedBy: Tommaso Fusco; dateIdentified: 2022; **Event:** samplingProtocol: Pitfall traps; eventDate: 2014-05-19; **Record Level:** collectionID: Roma3_5.8
- **Type status:** Other material. **Occurrence:** recordedBy: Fattorini S., Di Giulio A.; individualCount: 7; sex: 5 male, 2 female; lifeStage: adult; occurrenceID: E630BA52-2DB9-54A4-B5AA-423AE0AFB3CC; **Taxon:** scientificName: Trochosahispanica Simon, 1870; order: Araneae; family: Lycosidae; genus: Trochosa; **Location:** country: Italy; countryCode: IT; stateProvince: Rome; county: Rome; municipality: Rome; locality: Appia Antica Regional Park, Rome; locationRemarks: Caffarella Sud; decimalLatitude: 41.857247; decimalLongitude: 12.529211; geodeticDatum: WGS84; **Identification:** identifiedBy: Tommaso Fusco; dateIdentified: 2022; **Event:** samplingProtocol: Pitfall traps; eventDate: 2014-05-27; **Record Level:** collectionID: Roma3_5.8
- **Type status:** Other material. **Occurrence:** recordedBy: Fattorini S., Di Giulio A.; individualCount: 2; sex: male; lifeStage: adult; occurrenceID: 5853A850-23A9-5966-8B5B-0196804FEB2F; **Taxon:** scientificName: Trochosahispanica Simon, 1870; order: Araneae; family: Lycosidae; genus: Trochosa; **Location:** country: Italy; countryCode: IT; stateProvince: Rome; county: Rome; municipality: Rome; locality: Appia Antica Regional Park, Rome; locationRemarks: Caffarella Sud; decimalLatitude: 41.857247; decimalLongitude: 12.529211; geodeticDatum: WGS84; **Identification:** identifiedBy: Tommaso Fusco; dateIdentified: 2022; **Event:** samplingProtocol: Pitfall traps; eventDate: 2014-06-06; **Record Level:** collectionID: Roma3_5.8
- **Type status:** Other material. **Occurrence:** recordedBy: Fattorini S., Di Giulio A.; individualCount: 2; sex: 2 male, 2 female; lifeStage: adult; occurrenceID: 42060B99-C984-56E5-82E5-5350DEF9CD14; **Taxon:** scientificName: Trochosahispanica Simon, 1870; order: Araneae; family: Lycosidae; genus: Trochosa; **Location:** country: Italy; countryCode: IT; stateProvince: Rome; county: Rome; municipality: Rome; locality: Appia Antica Regional Park, Rome; locationRemarks: Caffarella Sud; decimalLatitude: 41.857247; decimalLongitude: 12.529211; geodeticDatum: WGS84; **Identification:** identifiedBy: Tommaso Fusco; dateIdentified: 2022; **Event:** samplingProtocol: Pitfall traps; eventDate: 2014-06-18; **Record Level:** collectionID: Roma3_5.8
- **Type status:** Other material. **Occurrence:** recordedBy: Fattorini S., Di Giulio A.; individualCount: 15; sex: 13 male, 2 female; lifeStage: adult; occurrenceID: A7F6EF75-4B0E-542B-8CFD-1B2626DA2B9D; **Taxon:** scientificName: Trochosahispanica Simon, 1870; order: Araneae; family: Lycosidae; genus: Trochosa; **Location:** country: Italy; countryCode: IT; stateProvince: Rome; county: Rome; municipality: Rome; locality: Appia Antica Regional Park, Rome; locationRemarks: Caffarella Sud 2; decimalLatitude: 41.856742; decimalLongitude: 12.529453; geodeticDatum: WGS84; **Identification:** identifiedBy: Tommaso Fusco; dateIdentified: 2022; **Event:** samplingProtocol: Pitfall traps; eventDate: 2014-05-19; **Record Level:** collectionID: Roma3_5.8
- **Type status:** Other material. **Occurrence:** recordedBy: Fattorini S., Di Giulio A.; individualCount: 14; sex: 12 male, 2 female; lifeStage: adult; occurrenceID: 58B74BD3-061C-5160-A356-64D9097D5E88; **Taxon:** scientificName: Trochosahispanica Simon, 1870; order: Araneae; family: Lycosidae; genus: Trochosa; **Location:** country: Italy; countryCode: IT; stateProvince: Rome; county: Rome; municipality: Rome; locality: Appia Antica Regional Park, Rome; locationRemarks: Caffarella Sud 2; decimalLatitude: 41.856742; decimalLongitude: 12.529453; geodeticDatum: WGS84; **Identification:** identifiedBy: Tommaso Fusco; dateIdentified: 2022; **Event:** samplingProtocol: Pitfall traps; eventDate: 2014-05-27; **Record Level:** collectionID: Roma3_5.8
- **Type status:** Other material. **Occurrence:** recordedBy: Fattorini S., Di Giulio A.; individualCount: 12; sex: male; lifeStage: adult; occurrenceID: 35173D7D-A880-5442-AE0A-E5068CAB1A75; **Taxon:** scientificName: Trochosahispanica Simon, 1870; order: Araneae; family: Lycosidae; genus: Trochosa; **Location:** country: Italy; countryCode: IT; stateProvince: Rome; county: Rome; municipality: Rome; locality: Appia Antica Regional Park, Rome; locationRemarks: Caffarella Sud 2; decimalLatitude: 41.856742; decimalLongitude: 12.529453; geodeticDatum: WGS84; **Identification:** identifiedBy: Tommaso Fusco; dateIdentified: 2022; **Event:** samplingProtocol: Pitfall traps; eventDate: 2014-06-06; **Record Level:** collectionID: Roma3_5.8
- **Type status:** Other material. **Occurrence:** recordedBy: Fattorini S., Di Giulio A.; individualCount: 19; sex: 16 male, 3 female; lifeStage: adult; occurrenceID: 6230FC70-EF4C-529D-AEF0-B3E0B5785CD2; **Taxon:** scientificName: Trochosahispanica Simon, 1870; order: Araneae; family: Lycosidae; genus: Trochosa; **Location:** country: Italy; countryCode: IT; stateProvince: Rome; county: Rome; municipality: Rome; locality: Appia Antica Regional Park, Rome; locationRemarks: Caffarella Sud 2; decimalLatitude: 41.856742; decimalLongitude: 12.529453; geodeticDatum: WGS84; **Identification:** identifiedBy: Tommaso Fusco; dateIdentified: 2022; **Event:** samplingProtocol: Pitfall traps; eventDate: 2014-06-18; **Record Level:** collectionID: Roma3_5.8
- **Type status:** Other material. **Occurrence:** recordedBy: Fattorini S., Di Giulio A.; individualCount: 5; sex: male; lifeStage: adult; occurrenceID: 8A7FCD17-372A-582D-A423-481C119E039C; **Taxon:** scientificName: Trochosahispanica Simon, 1870; order: Araneae; family: Lycosidae; genus: Trochosa; **Location:** country: Italy; countryCode: IT; stateProvince: Rome; county: Rome; municipality: Rome; locality: Appia Antica Regional Park, Rome; locationRemarks: Caffarella Sud 3; decimalLatitude: 41.856928; decimalLongitude: 12.528406; geodeticDatum: WGS84; **Identification:** identifiedBy: Tommaso Fusco; dateIdentified: 2022; **Event:** samplingProtocol: Pitfall traps; eventDate: 2014-05-19; **Record Level:** collectionID: Roma3_5.8
- **Type status:** Other material. **Occurrence:** recordedBy: Fattorini S., Di Giulio A.; individualCount: 4; sex: male; lifeStage: adult; occurrenceID: 36C2D734-863E-5DA2-A472-C1A440BA5E65; **Taxon:** scientificName: Trochosahispanica Simon, 1870; order: Araneae; family: Lycosidae; genus: Trochosa; **Location:** country: Italy; countryCode: IT; stateProvince: Rome; county: Rome; municipality: Rome; locality: Appia Antica Regional Park, Rome; locationRemarks: Caffarella Sud 3; decimalLatitude: 41.856928; decimalLongitude: 12.528406; geodeticDatum: WGS84; **Identification:** identifiedBy: Tommaso Fusco; dateIdentified: 2022; **Event:** samplingProtocol: Pitfall traps; eventDate: 2014-05-27; **Record Level:** collectionID: Roma3_5.8
- **Type status:** Other material. **Occurrence:** recordedBy: Fattorini S., Di Giulio A.; individualCount: 2; sex: male; lifeStage: adult; occurrenceID: 5FBB7E74-B576-55ED-AEB5-B40049464E7E; **Taxon:** scientificName: Trochosahispanica Simon, 1870; order: Araneae; family: Lycosidae; genus: Trochosa; **Location:** country: Italy; countryCode: IT; stateProvince: Rome; county: Rome; municipality: Rome; locality: Appia Antica Regional Park, Rome; locationRemarks: Caffarella Sud 3; decimalLatitude: 41.856928; decimalLongitude: 12.528406; geodeticDatum: WGS84; **Identification:** identifiedBy: Tommaso Fusco; dateIdentified: 2022; **Event:** samplingProtocol: Pitfall traps; eventDate: 2014-06-18; **Record Level:** collectionID: Roma3_5.8
- **Type status:** Other material. **Occurrence:** recordedBy: Fattorini S., Di Giulio A.; individualCount: 5; sex: 4 male, 1 female; lifeStage: adult; occurrenceID: ECA80D92-4C9B-58D4-997E-E8A884A5F977; **Taxon:** scientificName: Trochosahispanica Simon, 1870; order: Araneae; family: Lycosidae; genus: Trochosa; **Location:** country: Italy; countryCode: IT; stateProvince: Rome; county: Rome; municipality: Rome; locality: Appia Antica Regional Park, Rome; locationRemarks: Casal Verbeni; decimalLatitude: 41.815250; decimalLongitude: 12.552222; geodeticDatum: WGS84; **Identification:** identifiedBy: Tommaso Fusco; dateIdentified: 2022; **Event:** samplingProtocol: Pitfall traps; eventDate: 2014-05-26; **Record Level:** collectionID: Roma3_5.8
- **Type status:** Other material. **Occurrence:** recordedBy: Fattorini S., Di Giulio A.; individualCount: 2; sex: male; lifeStage: adult; occurrenceID: 3D56B8CE-40C5-51F8-A6C8-F8649B6017F2; **Taxon:** scientificName: Trochosahispanica Simon, 1870; order: Araneae; family: Lycosidae; genus: Trochosa; **Location:** country: Italy; countryCode: IT; stateProvince: Rome; county: Rome; municipality: Rome; locality: Appia Antica Regional Park, Rome; locationRemarks: Casal Verbeni; decimalLatitude: 41.815250; decimalLongitude: 12.552222; geodeticDatum: WGS84; **Identification:** identifiedBy: Tommaso Fusco; dateIdentified: 2022; **Event:** samplingProtocol: Pitfall traps; eventDate: 2014-06-05; **Record Level:** collectionID: Roma3_5.8
- **Type status:** Other material. **Occurrence:** recordedBy: Fattorini S., Di Giulio A.; individualCount: 3; sex: 2 male, 1 female; lifeStage: adult; occurrenceID: FEAF6F0A-D912-5A51-900B-A536CB928E6F; **Taxon:** scientificName: Trochosahispanica Simon, 1870; order: Araneae; family: Lycosidae; genus: Trochosa; **Location:** country: Italy; countryCode: IT; stateProvince: Rome; county: Rome; municipality: Rome; locality: Appia Antica Regional Park, Rome; locationRemarks: Casal Verbeni; decimalLatitude: 41.815250; decimalLongitude: 12.552222; geodeticDatum: WGS84; **Identification:** identifiedBy: Tommaso Fusco; dateIdentified: 2022; **Event:** samplingProtocol: Pitfall traps; eventDate: 2014-06-13; **Record Level:** collectionID: Roma3_5.8
- **Type status:** Other material. **Occurrence:** recordedBy: Fattorini S., Di Giulio A.; individualCount: 1; sex: male; lifeStage: adult; occurrenceID: 4F724350-4CF3-5DA1-B6BE-8726E38816B4; **Taxon:** scientificName: Trochosahispanica Simon, 1870; order: Araneae; family: Lycosidae; genus: Trochosa; **Location:** country: Italy; countryCode: IT; stateProvince: Rome; county: Rome; municipality: Rome; locality: Appia Antica Regional Park, Rome; locationRemarks: Casal Verbeni; decimalLatitude: 41.815250; decimalLongitude: 12.552222; geodeticDatum: WGS84; **Identification:** identifiedBy: Tommaso Fusco; dateIdentified: 2022; **Event:** samplingProtocol: Pitfall traps; eventDate: 2014-06-24; **Record Level:** collectionID: Roma3_5.8
- **Type status:** Other material. **Occurrence:** recordedBy: Fattorini S., Di Giulio A.; individualCount: 16; sex: 11 male, 5 female; lifeStage: adult; occurrenceID: 8D06C8D0-44F0-5322-BD45-61215044089D; **Taxon:** scientificName: Trochosahispanica Simon, 1870; order: Araneae; family: Lycosidae; genus: Trochosa; **Location:** country: Italy; countryCode: IT; stateProvince: Rome; county: Rome; municipality: Rome; locality: Appia Antica Regional Park, Rome; locationRemarks: Cava Fiorucci; decimalLatitude: 41.834106; decimalLongitude: 12.549264; geodeticDatum: WGS84; **Identification:** identifiedBy: Tommaso Fusco; dateIdentified: 2022; **Event:** samplingProtocol: Pitfall traps; eventDate: 2014-05-26; **Record Level:** collectionID: Roma3_5.8
- **Type status:** Other material. **Occurrence:** recordedBy: Fattorini S., Di Giulio A.; individualCount: 6; sex: 4 male, 2 female; lifeStage: adult; occurrenceID: A80C00ED-E8F2-595B-8378-7AFB4FE6E488; **Taxon:** scientificName: Trochosahispanica Simon, 1870; order: Araneae; family: Lycosidae; genus: Trochosa; **Location:** country: Italy; countryCode: IT; stateProvince: Rome; county: Rome; municipality: Rome; locality: Appia Antica Regional Park, Rome; locationRemarks: Cava Fiorucci; decimalLatitude: 41.834106; decimalLongitude: 12.549264; geodeticDatum: WGS84; **Identification:** identifiedBy: Tommaso Fusco; dateIdentified: 2022; **Event:** samplingProtocol: Pitfall traps; eventDate: 2014-06-05; **Record Level:** collectionID: Roma3_5.8
- **Type status:** Other material. **Occurrence:** recordedBy: Fattorini S., Di Giulio A.; individualCount: 12; sex: 9 male, 3 female; lifeStage: adult; occurrenceID: 2CB4A6F5-2CA4-591A-B42B-F79DD03F4701; **Taxon:** scientificName: Trochosahispanica Simon, 1870; order: Araneae; family: Lycosidae; genus: Trochosa; **Location:** country: Italy; countryCode: IT; stateProvince: Rome; county: Rome; municipality: Rome; locality: Appia Antica Regional Park, Rome; locationRemarks: Cava Fiorucci; decimalLatitude: 41.834106; decimalLongitude: 12.549264; geodeticDatum: WGS84; **Identification:** identifiedBy: Tommaso Fusco; dateIdentified: 2022; **Event:** samplingProtocol: Pitfall traps; eventDate: 2014-06-13; **Record Level:** collectionID: Roma3_5.8
- **Type status:** Other material. **Occurrence:** recordedBy: Fattorini S., Di Giulio A.; individualCount: 7; sex: 5 male, 2 female; lifeStage: adult; occurrenceID: FF727391-BA73-526C-A16E-4CD5957C2691; **Taxon:** scientificName: Trochosahispanica Simon, 1870; order: Araneae; family: Lycosidae; genus: Trochosa; **Location:** country: Italy; countryCode: IT; stateProvince: Rome; county: Rome; municipality: Rome; locality: Appia Antica Regional Park, Rome; locationRemarks: Cava Fiorucci; decimalLatitude: 41.834106; decimalLongitude: 12.549264; geodeticDatum: WGS84; **Identification:** identifiedBy: Tommaso Fusco; dateIdentified: 2022; **Event:** samplingProtocol: Pitfall traps; eventDate: 2014-06-24; **Record Level:** collectionID: Roma3_5.8
- **Type status:** Other material. **Occurrence:** recordedBy: Fattorini S., Di Giulio A.; individualCount: 7; sex: 5 male, 2 female; lifeStage: adult; occurrenceID: B0DCA3AF-FBF6-54A1-8844-2CC19ACB4333; **Taxon:** scientificName: Trochosahispanica Simon, 1870; order: Araneae; family: Lycosidae; genus: Trochosa; **Location:** country: Italy; countryCode: IT; stateProvince: Rome; county: Rome; municipality: Rome; locality: Appia Antica Regional Park, Rome; locationRemarks: Farnesiana; decimalLatitude: 41.839667; decimalLongitude: 12.525528; geodeticDatum: WGS84; **Identification:** identifiedBy: Tommaso Fusco; dateIdentified: 2022; **Event:** samplingProtocol: Pitfall traps; eventDate: 2014-05-26; **Record Level:** collectionID: Roma3_5.8
- **Type status:** Other material. **Occurrence:** recordedBy: Fattorini S., Di Giulio A.; individualCount: 7; sex: 4 male, 3 female; lifeStage: adult; occurrenceID: DD553A1A-A46C-5ACC-8D93-FD68B2B5E897; **Taxon:** scientificName: Trochosahispanica Simon, 1870; order: Araneae; family: Lycosidae; genus: Trochosa; **Location:** country: Italy; countryCode: IT; stateProvince: Rome; county: Rome; municipality: Rome; locality: Appia Antica Regional Park, Rome; locationRemarks: Farnesiana; decimalLatitude: 41.839667; decimalLongitude: 12.525528; geodeticDatum: WGS84; **Identification:** identifiedBy: Tommaso Fusco; dateIdentified: 2022; **Event:** samplingProtocol: Pitfall traps; eventDate: 2014-05-28; **Record Level:** collectionID: Roma3_5.8
- **Type status:** Other material. **Occurrence:** recordedBy: Fattorini S., Di Giulio A.; individualCount: 6; sex: male; lifeStage: adult; occurrenceID: 3E0F4BD6-77C6-5FF0-9DA6-3BF321E76772; **Taxon:** scientificName: Trochosahispanica Simon, 1870; order: Araneae; family: Lycosidae; genus: Trochosa; **Location:** country: Italy; countryCode: IT; stateProvince: Rome; county: Rome; municipality: Rome; locality: Appia Antica Regional Park, Rome; locationRemarks: Farnesiana; decimalLatitude: 41.839667; decimalLongitude: 12.525528; geodeticDatum: WGS84; **Identification:** identifiedBy: Tommaso Fusco; dateIdentified: 2022; **Event:** samplingProtocol: Pitfall traps; eventDate: 2014-06-09; **Record Level:** collectionID: Roma3_5.8
- **Type status:** Other material. **Occurrence:** recordedBy: Fattorini S., Di Giulio A.; individualCount: 9; sex: 7 male, 2 female; lifeStage: adult; occurrenceID: 33D93DE0-AAAC-51BC-A89A-EC66D6C71C40; **Taxon:** scientificName: Trochosahispanica Simon, 1870; order: Araneae; family: Lycosidae; genus: Trochosa; **Location:** country: Italy; countryCode: IT; stateProvince: Rome; county: Rome; municipality: Rome; locality: Appia Antica Regional Park, Rome; locationRemarks: Farnesiana; decimalLatitude: 41.839667; decimalLongitude: 12.525528; geodeticDatum: WGS84; **Identification:** identifiedBy: Tommaso Fusco; dateIdentified: 2022; **Event:** samplingProtocol: Pitfall traps; eventDate: 2014-06-19; **Record Level:** collectionID: Roma3_5.8
- **Type status:** Other material. **Occurrence:** recordedBy: Fattorini S., Di Giulio A.; individualCount: 8; sex: male; lifeStage: adult; occurrenceID: 638CD026-A3DC-5AE5-8A83-D1ED189F17A8; **Taxon:** scientificName: Trochosahispanica Simon, 1870; order: Araneae; family: Lycosidae; genus: Trochosa; **Location:** country: Italy; countryCode: IT; stateProvince: Rome; county: Rome; municipality: Rome; locality: Appia Antica Regional Park, Rome; locationRemarks: Tor Marancia; decimalLatitude: 41.850308; decimalLongitude: 12.503178; geodeticDatum: WGS84; **Identification:** identifiedBy: Tommaso Fusco; dateIdentified: 2022; **Event:** samplingProtocol: Pitfall traps; eventDate: 2014-05-15; **Record Level:** collectionID: Roma3_5.8
- **Type status:** Other material. **Occurrence:** recordedBy: Fattorini S., Di Giulio A.; individualCount: 6; sex: male; lifeStage: adult; occurrenceID: 28AA1A52-8074-538E-9F8C-4BB9FC268E92; **Taxon:** scientificName: Trochosahispanica Simon, 1870; order: Araneae; family: Lycosidae; genus: Trochosa; **Location:** country: Italy; countryCode: IT; stateProvince: Rome; county: Rome; municipality: Rome; locality: Appia Antica Regional Park, Rome; locationRemarks: Tor marancia; decimalLatitude: 41.850308; decimalLongitude: 12.503178; geodeticDatum: WGS84; **Identification:** identifiedBy: Tommaso Fusco; dateIdentified: 2022; **Event:** samplingProtocol: Pitfall traps; eventDate: 2014-05-26; **Record Level:** collectionID: Roma3_5.8
- **Type status:** Other material. **Occurrence:** recordedBy: Fattorini S., Di Giulio A.; individualCount: 13; sex: 11 male, 2 female; lifeStage: adult; occurrenceID: 7A227C21-E1F0-54D2-8F81-75F500300BAE; **Taxon:** scientificName: Trochosahispanica Simon, 1870; order: Araneae; family: Lycosidae; genus: Trochosa; **Location:** country: Italy; countryCode: IT; stateProvince: Rome; county: Rome; municipality: Rome; locality: Appia Antica Regional Park, Rome; locationRemarks: Tor Marancia; decimalLatitude: 41.850308; decimalLongitude: 12.503178; geodeticDatum: WGS84; **Identification:** identifiedBy: Tommaso Fusco; dateIdentified: 2022; **Event:** samplingProtocol: Pitfall traps; eventDate: 2014-06-05; **Record Level:** collectionID: Roma3_5.8
- **Type status:** Other material. **Occurrence:** recordedBy: Fattorini S., Di Giulio A.; individualCount: 4; sex: 6 male, 4 female; lifeStage: adult; occurrenceID: 3DC09CAC-BBFE-506B-ABD1-903395EBA3AA; **Taxon:** scientificName: Trochosahispanica Simon, 1870; order: Araneae; family: Lycosidae; genus: Trochosa; **Location:** country: Italy; countryCode: IT; stateProvince: Rome; county: Rome; municipality: Rome; locality: Appia Antica Regional Park, Rome; locationRemarks: Tor Marancia; decimalLatitude: 41.850308; decimalLongitude: 12.503178; geodeticDatum: WGS84; **Identification:** identifiedBy: Tommaso Fusco; dateIdentified: 2022; **Event:** samplingProtocol: Pitfall traps; eventDate: 2014-06-17; **Record Level:** collectionID: Roma3_5.8

##### Distribution

Mediterranean to Iran. Turano-Mediterranean (TUM) chorotype.

#### Miturgidae

Simon, 1886

AA5371F7-6E6C-5EFA-8E45-FD7D3F74883D

#### Zora spinimana

(Sundevall, 1833)

A630BD0C-432B-54C8-AB6D-1FDD9BF7BCF3

##### Materials

- **Type status:** Other material. **Occurrence:** recordedBy: Fattorini S., Di Giulio A.; individualCount: 1; sex: male; lifeStage: adult; occurrenceID: 83578907-98DA-5206-B347-47BA3CC22ED6; **Taxon:** scientificName: Zoraspinimana (Sundevall, 1833); order: Araneae; family: Miturgidae; genus: Zora; **Location:** country: Italy; countryCode: IT; stateProvince: Rome; county: Rome; municipality: Rome; locality: Appia Antica Regional Park, Rome; locationRemarks: Tor Marancia; decimalLatitude: 41.850308; decimalLongitude: 12.503178; geodeticDatum: WGS84; **Identification:** identifiedBy: Tommaso Fusco; dateIdentified: 2022; **Event:** samplingProtocol: Pitfall traps; eventDate: 2014-05-26; **Record Level:** collectionID: Roma3_5.8

##### Distribution

Europe, Turkey, Caucasus, Russia (Europe to Far East), Kazakhstan, Iran, Central Asia, China, Japan. Palaearctic (PAL) chorotype.

#### Nemesiidae

Simon, 1889

AF0191E6-C737-5157-BCF3-B1D4E36478DB

#### Nemesia bosmansi

Decae, 2024

E95D07A1-B1EC-5333-9DFB-B41142ACF1C9

##### Materials

- **Type status:** Other material. **Occurrence:** recordedBy: Fattorini S., Di Giulio A.; individualCount: 1; sex: male; lifeStage: adult; occurrenceID: B53D23FE-1977-5E32-8813-113535DD5186; **Taxon:** scientificName: Nemesiabosmansi Decae, 2024; order: Araneae; family: Nemesiidae; genus: Nemesia; **Location:** country: Italy; countryCode: IT; stateProvince: Rome; county: Rome; municipality: Rome; locality: Appia Antica Regional Park, Rome; locationRemarks: Appia Antica; decimalLatitude: 41.812575; decimalLongitude: 12.564011; geodeticDatum: WGS84; **Identification:** identifiedBy: Tommaso Fusco; dateIdentified: 2022; **Event:** samplingProtocol: Pitfall traps; eventDate: 2014-05-26; **Record Level:** collectionID: Roma3_5.8
- **Type status:** Other material. **Occurrence:** recordedBy: Fattorini S., Di Giulio A.; individualCount: 3; sex: male; lifeStage: adult; occurrenceID: AABA8547-C1CD-55C4-9738-CB57F97E441F; **Taxon:** scientificName: Nemesiabosmansi Decae, 2024; order: Araneae; family: Nemesiidae; genus: Nemesia; **Location:** country: Italy; countryCode: IT; stateProvince: Rome; county: Rome; municipality: Rome; locality: Appia Antica Regional Park, Rome; locationRemarks: Appia Antica; decimalLatitude: 41.812575; decimalLongitude: 12.564011; geodeticDatum: WGS84; **Identification:** identifiedBy: Tommaso Fusco; dateIdentified: 2022; **Event:** samplingProtocol: Pitfall traps; eventDate: 2014-06-13; **Record Level:** collectionID: Roma3_5.8
- **Type status:** Other material. **Occurrence:** recordedBy: Fattorini S., Di Giulio A.; individualCount: 8; sex: male; lifeStage: adult; occurrenceID: 18792DA1-9AAF-51B2-A487-3A7C2B1CA3B8; **Taxon:** scientificName: Nemesiabosmansi Decae, 2024; order: Araneae; family: Nemesiidae; genus: Nemesia; **Location:** country: Italy; countryCode: IT; stateProvince: Rome; county: Rome; municipality: Rome; locality: Appia Antica Regional Park, Rome; locationRemarks: Caffarella Centro; decimalLatitude: 41.864889; decimalLongitude: 12.516389; geodeticDatum: WGS84; **Identification:** identifiedBy: Tommaso Fusco; dateIdentified: 2022; **Event:** samplingProtocol: Pitfall traps; eventDate: 2013-10-31; **Record Level:** collectionID: Roma3_5.8
- **Type status:** Other material. **Occurrence:** recordedBy: Fattorini S., Di Giulio A.; individualCount: 1; sex: male; lifeStage: adult; occurrenceID: 6B0C0D7F-FDE4-5B6D-89D2-D531EDABE66C; **Taxon:** scientificName: Nemesiabosmansi Decae, 2024; order: Araneae; family: Nemesiidae; genus: Nemesia; **Location:** country: Italy; countryCode: IT; stateProvince: Rome; county: Rome; municipality: Rome; locality: Appia Antica Regional Park, Rome; locationRemarks: Caffarella Centro; decimalLatitude: 41.864889; decimalLongitude: 12.516389; geodeticDatum: WGS84; **Identification:** identifiedBy: Tommaso Fusco; dateIdentified: 2022; **Event:** samplingProtocol: Pitfall traps; eventDate: 2014-05-19; **Record Level:** collectionID: Roma3_5.8
- **Type status:** Other material. **Occurrence:** recordedBy: Fattorini S., Di Giulio A.; individualCount: 11; sex: male; lifeStage: adult; occurrenceID: 9F6996B3-F1E3-5946-B294-E68E13BCAD03; **Taxon:** scientificName: Nemesiabosmansi Decae, 2024; order: Araneae; family: Nemesiidae; genus: Nemesia; **Location:** country: Italy; countryCode: IT; stateProvince: Rome; county: Rome; municipality: Rome; locality: Appia Antica Regional Park, Rome; locationRemarks: Caffarella Centro; decimalLatitude: 41.864889; decimalLongitude: 12.516389; geodeticDatum: WGS84; **Identification:** identifiedBy: Tommaso Fusco; dateIdentified: 2022; **Event:** samplingProtocol: Pitfall traps; eventDate: 2013-11-13; **Record Level:** collectionID: Roma3_5.8
- **Type status:** Other material. **Occurrence:** recordedBy: Fattorini S., Di Giulio A.; individualCount: 1; sex: male; lifeStage: adult; occurrenceID: A787CB14-E76C-5697-89B3-86CED2E0C86D; **Taxon:** scientificName: Nemesiabosmansi Decae, 2024; order: Araneae; family: Nemesiidae; genus: Nemesia; **Location:** country: Italy; countryCode: IT; stateProvince: Rome; county: Rome; municipality: Rome; locality: Appia Antica Regional Park, Rome; locationRemarks: Caffarella Centro; decimalLatitude: 41.864889; decimalLongitude: 12.516389; geodeticDatum: WGS84; **Identification:** identifiedBy: Tommaso Fusco; dateIdentified: 2022; **Event:** samplingProtocol: Pitfall traps; eventDate: 2013-11-12; **Record Level:** collectionID: Roma3_5.8
- **Type status:** Other material. **Occurrence:** recordedBy: Fattorini S., Di Giulio A.; individualCount: 4; sex: male; lifeStage: adult; occurrenceID: 6B84259F-40C4-5D1E-9D22-B371D7214716; **Taxon:** scientificName: Nemesiabosmansi Decae, 2024; order: Araneae; family: Nemesiidae; genus: Nemesia; **Location:** country: Italy; countryCode: IT; stateProvince: Rome; county: Rome; municipality: Rome; locality: Appia Antica Regional Park, Rome; locationRemarks: Caffarella Centro; decimalLatitude: 41.864889; decimalLongitude: 12.516389; geodeticDatum: WGS84; **Identification:** identifiedBy: Tommaso Fusco; dateIdentified: 2022; **Event:** samplingProtocol: Pitfall traps; eventDate: 2013-11-20; **Record Level:** collectionID: Roma3_5.8
- **Type status:** Other material. **Occurrence:** recordedBy: Fattorini S., Di Giulio A.; individualCount: 1; sex: male; lifeStage: adult; occurrenceID: 19921934-5A75-55A5-A937-CDC14E7838B8; **Taxon:** scientificName: Nemesiabosmansi Decae, 2024; order: Araneae; family: Nemesiidae; genus: Nemesia; **Location:** country: Italy; countryCode: IT; stateProvince: Rome; county: Rome; municipality: Rome; locality: Appia Antica Regional Park, Rome; locationRemarks: Caffarella Nord; decimalLatitude: 41.867753; decimalLongitude: 12.512414; geodeticDatum: WGS84; **Identification:** identifiedBy: Tommaso Fusco; dateIdentified: 2022; **Event:** samplingProtocol: Pitfall traps; eventDate: 2013-10-31; **Record Level:** collectionID: Roma3_5.8
- **Type status:** Other material. **Occurrence:** recordedBy: Fattorini S., Di Giulio A.; individualCount: 1; sex: male; lifeStage: adult; occurrenceID: 3D7F7DFF-EAFF-5A40-A3E3-5884DEC27AEA; **Taxon:** scientificName: Nemesiabosmansi Decae, 2024; order: Araneae; family: Nemesiidae; genus: Nemesia; **Location:** country: Italy; countryCode: IT; stateProvince: Rome; county: Rome; municipality: Rome; locality: Appia Antica Regional Park, Rome; locationRemarks: Caffarella Nord; decimalLatitude: 41.867753; decimalLongitude: 12.512414; geodeticDatum: WGS84; **Identification:** identifiedBy: Tommaso Fusco; dateIdentified: 2022; **Event:** samplingProtocol: Pitfall traps; eventDate: 2013-11-13; **Record Level:** collectionID: Roma3_5.8
- **Type status:** Other material. **Occurrence:** recordedBy: Fattorini S., Di Giulio A.; individualCount: 1; sex: male; lifeStage: adult; occurrenceID: 0650215C-28F7-53AE-B148-0EEF35D2E96E; **Taxon:** scientificName: Nemesiabosmansi Decae, 2024; order: Araneae; family: Nemesiidae; genus: Nemesia; **Location:** country: Italy; countryCode: IT; stateProvince: Rome; county: Rome; municipality: Rome; locality: Appia Antica Regional Park, Rome; locationRemarks: Caffarella Nord; decimalLatitude: 41.867753; decimalLongitude: 12.512414; geodeticDatum: WGS84; **Identification:** identifiedBy: Tommaso Fusco; dateIdentified: 2022; **Event:** samplingProtocol: Pitfall traps; eventDate: 2013-11-20; **Record Level:** collectionID: Roma3_5.8
- **Type status:** Other material. **Occurrence:** recordedBy: Fattorini S., Di Giulio A.; individualCount: 2; sex: male; lifeStage: adult; occurrenceID: 88D7B8F7-E877-5AE2-AA93-B95F8E24FAB1; **Taxon:** scientificName: Nemesiabosmansi Decae, 2024; order: Araneae; family: Nemesiidae; genus: Nemesia; **Location:** country: Italy; countryCode: IT; stateProvince: Rome; county: Rome; municipality: Rome; locality: Appia Antica Regional Park, Rome; locationRemarks: Caffarella Nord; decimalLatitude: 41.867753; decimalLongitude: 12.512414; geodeticDatum: WGS84; **Identification:** identifiedBy: Tommaso Fusco; dateIdentified: 2022; **Event:** samplingProtocol: Pitfall traps; eventDate: 2013-11-29; **Record Level:** collectionID: Roma3_5.8
- **Type status:** Other material. **Occurrence:** recordedBy: Fattorini S., Di Giulio A.; individualCount: 2; sex: male; lifeStage: adult; occurrenceID: 608E175C-D76A-53D1-A0B1-CABA02E16B07; **Taxon:** scientificName: Nemesiabosmansi Decae, 2024; order: Araneae; family: Nemesiidae; genus: Nemesia; **Location:** country: Italy; countryCode: IT; stateProvince: Rome; county: Rome; municipality: Rome; locality: Appia Antica Regional Park, Rome; locationRemarks: Caffarella Sud; decimalLatitude: 41.857247; decimalLongitude: 12.529211; geodeticDatum: WGS84; **Identification:** identifiedBy: Tommaso Fusco; dateIdentified: 2022; **Event:** samplingProtocol: Pitfall traps; eventDate: 2013-11-12; **Record Level:** collectionID: Roma3_5.8
- **Type status:** Other material. **Occurrence:** recordedBy: Fattorini S., Di Giulio A.; individualCount: 1; sex: male; lifeStage: adult; occurrenceID: 651F9D30-0FD0-5DC3-A0EB-2BA71D9F9F71; **Taxon:** scientificName: Nemesiabosmansi Decae, 2024; order: Araneae; family: Nemesiidae; genus: Nemesia; **Location:** country: Italy; countryCode: IT; stateProvince: Rome; county: Rome; municipality: Rome; locality: Appia Antica Regional Park, Rome; locationRemarks: Cava Fiorucci; decimalLatitude: 41.834106; decimalLongitude: 12.549264; geodeticDatum: WGS84; **Identification:** identifiedBy: Tommaso Fusco; dateIdentified: 2022; **Event:** samplingProtocol: Pitfall traps; eventDate: 2014-05-26; **Record Level:** collectionID: Roma3_5.8
- **Type status:** Other material. **Occurrence:** recordedBy: Fattorini S., Di Giulio A.; individualCount: 3; sex: male; lifeStage: adult; occurrenceID: 5DEBF83D-D469-56DB-9941-DCD49D2C16A3; **Taxon:** scientificName: Nemesiabosmansi Decae, 2024; order: Araneae; family: Nemesiidae; genus: Nemesia; **Location:** country: Italy; countryCode: IT; stateProvince: Rome; county: Rome; municipality: Rome; locality: Appia Antica Regional Park, Rome; locationRemarks: Cava Fiorucci; decimalLatitude: 41.834106; decimalLongitude: 12.549264; geodeticDatum: WGS84; **Identification:** identifiedBy: Tommaso Fusco; dateIdentified: 2022; **Event:** samplingProtocol: Pitfall traps; eventDate: 2013-11-07; **Record Level:** collectionID: Roma3_5.8
- **Type status:** Other material. **Occurrence:** recordedBy: Fattorini S., Di Giulio A.; individualCount: 3; sex: male; lifeStage: adult; occurrenceID: FF88BD70-3105-50A3-A1BE-75D018069E1E; **Taxon:** scientificName: Nemesiabosmansi Decae, 2024; order: Araneae; family: Nemesiidae; genus: Nemesia; **Location:** country: Italy; countryCode: IT; stateProvince: Rome; county: Rome; municipality: Rome; locality: Appia Antica Regional Park, Rome; locationRemarks: Cava Fiorucci; decimalLatitude: 41.834106; decimalLongitude: 12.549264; geodeticDatum: WGS84; **Identification:** identifiedBy: Tommaso Fusco; dateIdentified: 2022; **Event:** samplingProtocol: Pitfall traps; eventDate: 2013-11-18; **Record Level:** collectionID: Roma3_5.8
- **Type status:** Other material. **Occurrence:** recordedBy: Fattorini S., Di Giulio A.; individualCount: 1; sex: male; lifeStage: adult; occurrenceID: 35C077F3-D196-5267-AEC5-5371831C0AA0; **Taxon:** scientificName: Nemesiabosmansi Decae, 2024; order: Araneae; family: Nemesiidae; genus: Nemesia; **Location:** country: Italy; countryCode: IT; stateProvince: Rome; county: Rome; municipality: Rome; locality: Appia Antica Regional Park, Rome; locationRemarks: Cava Fiorucci; decimalLatitude: 41.834106; decimalLongitude: 12.549264; geodeticDatum: WGS84; **Identification:** identifiedBy: Tommaso Fusco; dateIdentified: 2022; **Event:** samplingProtocol: Pitfall traps; eventDate: 2014-05-26; **Record Level:** collectionID: Roma3_5.8
- **Type status:** Other material. **Occurrence:** recordedBy: Fattorini S., Di Giulio A.; individualCount: 4; sex: male; lifeStage: adult; occurrenceID: 7D8C2540-3305-5916-8144-9F0F95465413; **Taxon:** scientificName: Nemesiabosmansi Decae, 2024; order: Araneae; family: Nemesiidae; genus: Nemesia; **Location:** country: Italy; countryCode: IT; stateProvince: Rome; county: Rome; municipality: Rome; locality: Appia Antica Regional Park, Rome; locationRemarks: Farnesiana; decimalLatitude: 41.839667; decimalLongitude: 12.525528; geodeticDatum: WGS84; **Identification:** identifiedBy: Tommaso Fusco; dateIdentified: 2022; **Event:** samplingProtocol: Pitfall traps; eventDate: 2013-11-04; **Record Level:** collectionID: Roma3_5.8
- **Type status:** Other material. **Occurrence:** recordedBy: Fattorini S., Di Giulio A.; individualCount: 2; sex: male; lifeStage: adult; occurrenceID: 1D699510-3999-55AA-AB22-201CAA1C571C; **Taxon:** scientificName: Nemesiabosmansi Decae, 2024; order: Araneae; family: Nemesiidae; genus: Nemesia; **Location:** country: Italy; countryCode: IT; stateProvince: Rome; county: Rome; municipality: Rome; locality: Appia Antica Regional Park, Rome; locationRemarks: Farnesiana; decimalLatitude: 41.839667; decimalLongitude: 12.525528; geodeticDatum: WGS84; **Identification:** identifiedBy: Tommaso Fusco; dateIdentified: 2022; **Event:** samplingProtocol: Pitfall traps; eventDate: 2013-11-13; **Record Level:** collectionID: Roma3_5.8
- **Type status:** Other material. **Occurrence:** recordedBy: Fattorini S., Di Giulio A.; individualCount: 2; sex: male; lifeStage: adult; occurrenceID: AF8C6AC9-CECB-5058-A438-F53E35F5B82A; **Taxon:** scientificName: Nemesiabosmansi Decae, 2024; order: Araneae; family: Nemesiidae; genus: Nemesia; **Location:** country: Italy; countryCode: IT; stateProvince: Rome; county: Rome; municipality: Rome; locality: Appia Antica Regional Park, Rome; locationRemarks: Farnesiana; decimalLatitude: 41.839667; decimalLongitude: 12.525528; geodeticDatum: WGS84; **Identification:** identifiedBy: Tommaso Fusco; dateIdentified: 2022; **Event:** samplingProtocol: Pitfall traps; eventDate: 2013-11-25; **Record Level:** collectionID: Roma3_5.8
- **Type status:** Other material. **Occurrence:** recordedBy: Fattorini S., Di Giulio A.; individualCount: 1; sex: male; lifeStage: adult; occurrenceID: CA1FB5FD-63CA-59F2-BED7-8CFFB0770F3F; **Taxon:** scientificName: Nemesiabosmansi Decae, 2024; order: Araneae; family: Nemesiidae; genus: Nemesia; **Location:** country: Italy; countryCode: IT; stateProvince: Rome; county: Rome; municipality: Rome; locality: Appia Antica Regional Park, Rome; locationRemarks: Farnesiana; decimalLatitude: 41.839667; decimalLongitude: 12.525528; geodeticDatum: WGS84; **Identification:** identifiedBy: Tommaso Fusco; dateIdentified: 2022; **Event:** samplingProtocol: Pitfall traps; eventDate: 2014-06-19; **Record Level:** collectionID: Roma3_5.8
- **Type status:** Other material. **Occurrence:** recordedBy: Fattorini S., Di Giulio A.; individualCount: 1; sex: male; lifeStage: adult; occurrenceID: 27FA3C9A-2B6E-5CC6-A17A-F8E05BBE580B; **Taxon:** scientificName: Nemesiabosmansi Decae, 2024; order: Araneae; family: Nemesiidae; genus: Nemesia; **Location:** country: Italy; countryCode: IT; stateProvince: Rome; county: Rome; municipality: Rome; locality: Appia Antica Regional Park, Rome; locationRemarks: Farnesiana; decimalLatitude: 41.839667; decimalLongitude: 12.525528; geodeticDatum: WGS84; **Identification:** identifiedBy: Tommaso Fusco; dateIdentified: 2022; **Event:** samplingProtocol: Pitfall traps; eventDate: 2013-12-02; **Record Level:** collectionID: Roma3_5.8
- **Type status:** Other material. **Occurrence:** recordedBy: Fattorini S., Di Giulio A.; individualCount: 17; sex: male; lifeStage: adult; occurrenceID: E0222E05-BFDA-56CF-AD4A-C7DD1D6C2248; **Taxon:** scientificName: Nemesiabosmansi Decae, 2024; order: Araneae; family: Nemesiidae; genus: Nemesia; **Location:** country: Italy; countryCode: IT; stateProvince: Rome; county: Rome; municipality: Rome; locality: Appia Antica Regional Park, Rome; locationRemarks: San Sebastiano; decimalLatitude: 41.855733; decimalLongitude: 12.515114; geodeticDatum: WGS84; **Identification:** identifiedBy: Tommaso Fusco; dateIdentified: 2022; **Event:** samplingProtocol: Pitfall traps; eventDate: 2013-11-04; **Record Level:** collectionID: Roma3_5.8
- **Type status:** Other material. **Occurrence:** recordedBy: Fattorini S., Di Giulio A.; individualCount: 6; sex: male; lifeStage: adult; occurrenceID: CD35226A-3E83-5099-88AD-FE4F10A79AC0; **Taxon:** scientificName: Nemesiabosmansi Decae, 2024; order: Araneae; family: Nemesiidae; genus: Nemesia; **Location:** country: Italy; countryCode: IT; stateProvince: Rome; county: Rome; municipality: Rome; locality: Appia Antica Regional Park, Rome; locationRemarks: San Sebastiano; decimalLatitude: 41.855733; decimalLongitude: 12.515114; geodeticDatum: WGS84; **Identification:** identifiedBy: Tommaso Fusco; dateIdentified: 2022; **Event:** samplingProtocol: Pitfall traps; eventDate: 2013-11-12; **Record Level:** collectionID: Roma3_5.8
- **Type status:** Other material. **Occurrence:** recordedBy: Fattorini S., Di Giulio A.; individualCount: 5; sex: male; lifeStage: adult; occurrenceID: E45819B1-1F53-5207-8919-100936D48557; **Taxon:** scientificName: Nemesiabosmansi Decae, 2024; order: Araneae; family: Nemesiidae; genus: Nemesia; **Location:** country: Italy; countryCode: IT; stateProvince: Rome; county: Rome; municipality: Rome; locality: Appia Antica Regional Park, Rome; locationRemarks: San Sebastiano; decimalLatitude: 41.855733; decimalLongitude: 12.515114; geodeticDatum: WGS84; **Identification:** identifiedBy: Tommaso Fusco; dateIdentified: 2022; **Event:** samplingProtocol: Pitfall traps; eventDate: 2013-11-25; **Record Level:** collectionID: Roma3_5.8
- **Type status:** Other material. **Occurrence:** recordedBy: Fattorini S., Di Giulio A.; individualCount: 2; sex: male; lifeStage: adult; occurrenceID: F5906473-75CD-5D88-A2D5-6C39659BC8E3; **Taxon:** scientificName: Nemesiabosmansi Decae, 2024; order: Araneae; family: Nemesiidae; genus: Nemesia; **Location:** country: Italy; countryCode: IT; stateProvince: Rome; county: Rome; municipality: Rome; locality: Appia Antica Regional Park, Rome; locationRemarks: San Sebastiano; decimalLatitude: 41.855733; decimalLongitude: 12.515114; geodeticDatum: WGS84; **Identification:** identifiedBy: Tommaso Fusco; dateIdentified: 2022; **Event:** samplingProtocol: Pitfall traps; eventDate: 2014-06-19; **Record Level:** collectionID: Roma3_5.8
- **Type status:** Other material. **Occurrence:** recordedBy: Fattorini S., Di Giulio A.; individualCount: 1; sex: male; lifeStage: adult; occurrenceID: 2C9B411A-B4E9-57B5-A599-19E63BBD989E; **Taxon:** scientificName: Nemesiabosmansi Decae, 2024; order: Araneae; family: Nemesiidae; genus: Nemesia; **Location:** country: Italy; countryCode: IT; stateProvince: Rome; county: Rome; municipality: Rome; locality: Appia Antica Regional Park, Rome; locationRemarks: San Sebastiano; decimalLatitude: 41.855733; decimalLongitude: 12.515114; geodeticDatum: WGS84; **Identification:** identifiedBy: Tommaso Fusco; dateIdentified: 2022; **Event:** samplingProtocol: Pitfall traps; eventDate: 2013-12-02; **Record Level:** collectionID: Roma3_5.8
- **Type status:** Other material. **Occurrence:** recordedBy: Fattorini S., Di Giulio A.; individualCount: 1; sex: male; lifeStage: adult; occurrenceID: 5A903B33-74F1-5F98-A05F-5C39B8D4784E; **Taxon:** scientificName: Nemesiabosmansi Decae, 2024; order: Araneae; family: Nemesiidae; genus: Nemesia; **Location:** country: Italy; countryCode: IT; stateProvince: Rome; county: Rome; municipality: Rome; locality: Appia Antica Regional Park, Rome; locationRemarks: Tor Marancia; decimalLatitude: 41.850308; decimalLongitude: 12.503178; geodeticDatum: WGS84; **Identification:** identifiedBy: Tommaso Fusco; dateIdentified: 2022; **Event:** samplingProtocol: Pitfall traps; eventDate: 2013-10-28; **Record Level:** collectionID: Roma3_5.8
- **Type status:** Other material. **Occurrence:** recordedBy: Fattorini S., Di Giulio A.; individualCount: 1; sex: male; lifeStage: adult; occurrenceID: 6E292D6E-C450-577D-B1F4-AF381EF78143; **Taxon:** scientificName: Nemesiabosmansi Decae, 2024; order: Araneae; family: Nemesiidae; genus: Nemesia; **Location:** country: Italy; countryCode: IT; stateProvince: Rome; county: Rome; municipality: Rome; locality: Appia Antica Regional Park, Rome; locationRemarks: Tor Marancia; decimalLatitude: 41.850308; decimalLongitude: 12.503178; geodeticDatum: WGS84; **Identification:** identifiedBy: Tommaso Fusco; dateIdentified: 2022; **Event:** samplingProtocol: Pitfall traps; eventDate: 2014-05-27; **Record Level:** collectionID: Roma3_5.8
- **Type status:** Other material. **Occurrence:** recordedBy: Fattorini S., Di Giulio A.; individualCount: 1; sex: male; lifeStage: adult; occurrenceID: 952497FE-B29B-57DA-A71D-CA9C3F10E30D; **Taxon:** scientificName: Nemesiabosmansi Decae, 2024; order: Araneae; family: Nemesiidae; genus: Nemesia; **Location:** country: Italy; countryCode: IT; stateProvince: Rome; county: Rome; municipality: Rome; locality: Appia Antica Regional Park, Rome; locationRemarks: Tor Marancia; decimalLatitude: 41.850308; decimalLongitude: 12.503178; geodeticDatum: WGS84; **Identification:** identifiedBy: Tommaso Fusco; dateIdentified: 2022; **Event:** samplingProtocol: Pitfall traps; eventDate: 2013-11-18; **Record Level:** collectionID: Roma3_5.8
- **Type status:** Other material. **Occurrence:** recordedBy: Fattorini S., Di Giulio A.; individualCount: 1; sex: male; lifeStage: adult; occurrenceID: A2E3DB06-B12C-57E6-9909-14942C43229D; **Taxon:** scientificName: Nemesiabosmansi Decae, 2024; order: Araneae; family: Nemesiidae; genus: Nemesia; **Location:** country: Italy; countryCode: IT; stateProvince: Rome; county: Rome; municipality: Rome; locality: Appia Antica Regional Park, Rome; locationRemarks: Tor Marancia; decimalLatitude: 41.850308; decimalLongitude: 12.503178; geodeticDatum: WGS84; **Identification:** identifiedBy: Tommaso Fusco; dateIdentified: 2022; **Event:** samplingProtocol: Pitfall traps; eventDate: 2014-06-17; **Record Level:** collectionID: Roma3_5.8
- **Type status:** Other material. **Occurrence:** recordedBy: Fattorini S., Di Giulio A.; individualCount: 1; sex: male; lifeStage: adult; occurrenceID: 2E6564DA-0EFE-583B-BAB4-AF0CC78A3A79; **Taxon:** scientificName: Nemesiabosmansi Decae, 2024; order: Araneae; family: Nemesiidae; genus: Nemesia; **Location:** country: Italy; countryCode: IT; stateProvince: Rome; county: Rome; municipality: Rome; locality: Appia Antica Regional Park, Rome; locationRemarks: Torre Selce; decimalLatitude: 41.816611; decimalLongitude: 12.560667; geodeticDatum: WGS84; **Identification:** identifiedBy: Tommaso Fusco; dateIdentified: 2022; **Event:** samplingProtocol: Pitfall traps; eventDate: 2014-05-26; **Record Level:** collectionID: Roma3_5.8
- **Type status:** Other material. **Occurrence:** recordedBy: Fattorini S., Di Giulio A.; individualCount: 1; sex: male; lifeStage: adult; occurrenceID: 7A66B685-8DDC-52AB-AD53-52A5063C4052; **Taxon:** scientificName: Nemesiabosmansi Decae, 2024; order: Araneae; family: Nemesiidae; genus: Nemesia; **Location:** country: Italy; countryCode: IT; stateProvince: Rome; county: Rome; municipality: Rome; locality: Appia Antica Regional Park, Rome; locationRemarks: Torre Selce; decimalLatitude: 41.816611; decimalLongitude: 12.560667; geodeticDatum: WGS84; **Identification:** identifiedBy: Tommaso Fusco; dateIdentified: 2022; **Event:** samplingProtocol: Pitfall traps; eventDate: 2013-11-07; **Record Level:** collectionID: Roma3_5.8

##### Distribution

Italian Endemic (END) species only found in the Latium Region (Decae 2024).

#### Nesticidae

Simon, 1894

12DEA5FE-8C6A-598D-87C6-CDB331433F11

#### Kryptonesticus eremita

(Simon, 1880)

1BA0CAF9-18AB-5C2D-8811-FA6A1686F7E3

##### Materials

- **Type status:** Other material. **Occurrence:** recordedBy: Fattorini S., Di Giulio A.; individualCount: 1; sex: male; lifeStage: adult; occurrenceID: 259737DB-55BB-53AD-BDD0-C1DED03747DA; **Taxon:** scientificName: Kryptonesticuseremita (Simon, 1880); order: Araneae; family: Nesticidae; genus: Kryptonesticus; **Location:** country: Italy; countryCode: IT; stateProvince: Rome; county: Rome; municipality: Rome; locality: Appia Antica Regional Park, Rome; locationRemarks: Cava Fiorucci; decimalLatitude: 41.834106; decimalLongitude: 12.549264; geodeticDatum: WGS84; **Identification:** identifiedBy: Tommaso Fusco; dateIdentified: 2022; **Event:** samplingProtocol: Pitfall traps; eventDate: 2014-06-13; **Record Level:** collectionID: Roma3_5.8

##### Distribution

Europe, Turkey. European (EUR) chorotype.

#### Oecobiidae

Blackwall, 1862

2B571DFF-4414-58A4-BCB5-F4B84352A075

#### Oecobius maculatus

Simon, 1870

7ED75FD4-AA6B-526C-838D-246913F44826

##### Materials

- **Type status:** Other material. **Occurrence:** recordedBy: Fattorini S., Di Giulio A.; individualCount: 1; sex: male; lifeStage: adult; occurrenceID: EA438464-1DD3-5338-B845-64579E52D210; **Taxon:** scientificName: Oecobiusmaculatus Simon, 1870; order: Araneae; family: Oecobiidae; genus: Oecobius; **Location:** country: Italy; countryCode: IT; stateProvince: Rome; county: Rome; municipality: Rome; locality: Appia Antica Regional Park, Rome; locationRemarks: Cava Fiorucci; decimalLatitude: 41.834106; decimalLongitude: 12.549264; geodeticDatum: WGS84; **Identification:** identifiedBy: Tommaso Fusco; dateIdentified: 2022; **Event:** samplingProtocol: Pitfall traps; eventDate: 2014-06-13; **Record Level:** collectionID: Roma3_5.8
- **Type status:** Other material. **Occurrence:** recordedBy: Fattorini S., Di Giulio A.; individualCount: 1; sex: female; lifeStage: adult; occurrenceID: 8D7C5D8E-DFD3-59ED-9494-0E85331A97EF; **Taxon:** scientificName: Oecobiusmaculatus Simon, 1870; order: Araneae; family: Oecobiidae; genus: Oecobius; **Location:** country: Italy; countryCode: IT; stateProvince: Rome; county: Rome; municipality: Rome; locality: Appia Antica Regional Park, Rome; locationRemarks: Casal Verbeni; decimalLatitude: 41.815250; decimalLongitude: 12.552222; geodeticDatum: WGS84; **Identification:** identifiedBy: Tommaso Fusco; dateIdentified: 2022; **Event:** samplingProtocol: Pitfall traps; eventDate: 2014-06-13; **Record Level:** collectionID: Roma3_5.8

##### Distribution

Europe to Azerbaijan. Introduced to USA and Mexico. Europeo-Mediterranean (EUM) chorotype.

#### Oecobius navus

Blackwall, 1859

932986D7-73CC-5DFF-BE77-16F8F181C786

##### Materials

- **Type status:** Other material. **Occurrence:** recordedBy: Fattorini S., Di Giulio A.; individualCount: 1; sex: male; lifeStage: adult; occurrenceID: 58CC8F84-8A4C-5435-85BC-9B3CBE312942; **Taxon:** scientificName: Oecobiusnavus Blackwall, 1859; order: Araneae; family: Oecobiidae; genus: Oecobius; **Location:** country: Italy; countryCode: IT; stateProvince: Rome; county: Rome; municipality: Rome; locality: Appia Antica Regional Park, Rome; locationRemarks: San Sebastiano; decimalLatitude: 41.855733; decimalLongitude: 12.515114; geodeticDatum: WGS84; **Identification:** identifiedBy: Tommaso Fusco; dateIdentified: 2022; **Event:** samplingProtocol: Pitfall traps; eventDate: 2014-06-09; **Record Level:** collectionID: Roma3_5.8

##### Distribution

Europe, northern Africa, South Africa, Turkey, Caucasus. Introduced to South Africa, China, Korea, Japan, New Zealand, Canada, USA, South America. Cosmopolitan (COS) chorotype.

#### Oonopidae

Simon, 1890

83E9D3C1-7417-50EF-9415-54487E47C794

#### Orchestina longipes

Dalmas, 1922

BC044E71-87BC-5F39-BA38-3FB67893A7C7

##### Materials

- **Type status:** Other material. **Occurrence:** recordedBy: Fattorini S., Di Giulio A.; individualCount: 1; sex: female; lifeStage: adult; occurrenceID: D91E912A-840E-54F8-8155-B406BE14C516; **Taxon:** scientificName: Orchestinalongipes Dalmas, 1922; order: Araneae; family: Oonopidae; genus: Orchestina; **Location:** country: Italy; countryCode: IT; stateProvince: Rome; county: Rome; municipality: Rome; locality: Appia Antica Regional Park, Rome; locationRemarks: Cava Fiorucci; decimalLatitude: 41.834106; decimalLongitude: 12.549264; geodeticDatum: WGS84; **Identification:** identifiedBy: Tommaso Fusco; dateIdentified: 2022; **Event:** samplingProtocol: Pitfall traps; eventDate: 2014-06-13; **Record Level:** collectionID: Roma3_5.8
- **Type status:** Other material. **Occurrence:** recordedBy: Fattorini S., Di Giulio A.; individualCount: 1; sex: female; lifeStage: adult; occurrenceID: 92B6D307-3727-53B4-8144-B0CC9E4A5168; **Taxon:** scientificName: Orchestinalongipes Dalmas, 1922; order: Araneae; family: Oonopidae; genus: Orchestina; **Location:** country: Italy; countryCode: IT; stateProvince: Rome; county: Rome; municipality: Rome; locality: Appia Antica Regional Park, Rome; locationRemarks: Farnesiana; decimalLatitude: 41.839667; decimalLongitude: 12.525528; geodeticDatum: WGS84; **Identification:** identifiedBy: Tommaso Fusco; dateIdentified: 2022; **Event:** samplingProtocol: Pitfall traps; eventDate: 2014-06-19; **Record Level:** collectionID: Roma3_5.8
- **Type status:** Other material. **Occurrence:** recordedBy: Fattorini S., Di Giulio A.; individualCount: 2; sex: 1 male, 1 female; lifeStage: adult; occurrenceID: 2574A575-7EA9-5BE3-B0A8-DF5B93DBA5B4; **Taxon:** scientificName: Orchestinalongipes Dalmas, 1922; order: Araneae; family: Oonopidae; genus: Orchestina; **Location:** country: Italy; countryCode: IT; stateProvince: Rome; county: Rome; municipality: Rome; locality: Appia Antica Regional Park, Rome; locationRemarks: Torre Selce; decimalLatitude: 41.816611; decimalLongitude: 12.560667; geodeticDatum: WGS84; **Identification:** identifiedBy: Tommaso Fusco; dateIdentified: 2022; **Event:** samplingProtocol: Pitfall traps; eventDate: 2014-06-13; **Record Level:** collectionID: Roma3_5.8
- **Type status:** Other material. **Occurrence:** recordedBy: Fattorini S., Di Giulio A.; individualCount: 1; sex: female; lifeStage: adult; occurrenceID: 1ACB9AB4-9A97-5FE9-A875-6B9DD64A462B; **Taxon:** scientificName: Orchestinalongipes Dalmas, 1922; order: Araneae; family: Oonopidae; genus: Orchestina; **Location:** country: Italy; countryCode: IT; stateProvince: Rome; county: Rome; municipality: Rome; locality: Appia Antica Regional Park, Rome; locationRemarks: San Sebastiano; decimalLatitude: 41.855733; decimalLongitude: 12.515114; geodeticDatum: WGS84; **Identification:** identifiedBy: Tommaso Fusco; dateIdentified: 2022; **Event:** samplingProtocol: Pitfall traps; eventDate: 2014-06-09; **Record Level:** collectionID: Roma3_5.8
- **Type status:** Other material. **Occurrence:** recordedBy: Fattorini S., Di Giulio A.; individualCount: 1; sex: female; lifeStage: adult; occurrenceID: EAD5FCB6-A959-592D-BB87-B3F2A768B9D5; **Taxon:** scientificName: Orchestinalongipes Dalmas, 1922; order: Araneae; family: Oonopidae; genus: Orchestina; **Location:** country: Italy; countryCode: IT; stateProvince: Rome; county: Rome; municipality: Rome; locality: Appia Antica Regional Park, Rome; locationRemarks: Caffarella Centro; decimalLatitude: 41.864889; decimalLongitude: 12.516389; geodeticDatum: WGS84; **Identification:** identifiedBy: Tommaso Fusco; dateIdentified: 2022; **Event:** samplingProtocol: Pitfall traps; eventDate: 2014-06-18; **Record Level:** collectionID: Roma3_5.8

##### Distribution

Portugal, Spain (Balearic Is.), France (Corsica), Italy. W-Mediterranean (WME) chorotype.

#### Silhouettella loricatula

(Roewer, 1942)

45E4B0BC-6911-54B6-ABDF-50D165602594

##### Materials

- **Type status:** Other material. **Occurrence:** recordedBy: Fattorini S., Di Giulio A.; individualCount: 1; sex: male; lifeStage: adult; occurrenceID: B5E7C268-7DFF-50B3-BB87-CEC57ECBF769; **Taxon:** scientificName: Silhouettellaloricatula (Roewer, 1942); order: Araneae; family: Oonopidae; genus: Silhouettella; **Location:** country: Italy; countryCode: IT; stateProvince: Rome; county: Rome; municipality: Rome; locality: Appia Antica Regional Park, Rome; locationRemarks: Casal Verbeni; decimalLatitude: 41.815250; decimalLongitude: 12.552222; geodeticDatum: WGS84; **Identification:** identifiedBy: Tommaso Fusco; dateIdentified: 2022; **Event:** samplingProtocol: Pitfall traps; eventDate: 2014-06-13; **Record Level:** collectionID: Roma3_5.8
- **Type status:** Other material. **Occurrence:** recordedBy: Fattorini S., Di Giulio A.; individualCount: 1; sex: female; lifeStage: adult; occurrenceID: 6FDBEB5B-D809-5B0C-AE80-C080697C2B3D; **Taxon:** scientificName: Silhouettellaloricatula (Roewer, 1942); order: Araneae; family: Oonopidae; genus: Silhouettella; **Location:** country: Italy; countryCode: IT; stateProvince: Rome; county: Rome; municipality: Rome; locality: Appia Antica Regional Park, Rome; locationRemarks: Casal Verbeni; decimalLatitude: 41.815250; decimalLongitude: 12.552222; geodeticDatum: WGS84; **Identification:** identifiedBy: Tommaso Fusco; dateIdentified: 2022; **Event:** samplingProtocol: Pitfall traps; eventDate: 2014-06-05; **Record Level:** collectionID: Roma3_5.8
- **Type status:** Other material. **Occurrence:** recordedBy: Fattorini S., Di Giulio A.; individualCount: 1; sex: male; lifeStage: adult; occurrenceID: 74AD5016-7484-54CE-9065-7F4BA2873BF7; **Taxon:** scientificName: Silhouettellaloricatula (Roewer, 1942); order: Araneae; family: Oonopidae; genus: Silhouettella; **Location:** country: Italy; countryCode: IT; stateProvince: Rome; county: Rome; municipality: Rome; locality: Appia Antica Regional Park, Rome; locationRemarks: Farnesiana; decimalLatitude: 41.839667; decimalLongitude: 12.525528; geodeticDatum: WGS84; **Identification:** identifiedBy: Tommaso Fusco; dateIdentified: 2022; **Event:** samplingProtocol: Pitfall traps; eventDate: 2014-05-28; **Record Level:** collectionID: Roma3_5.8
- **Type status:** Other material. **Occurrence:** recordedBy: Fattorini S., Di Giulio A.; individualCount: 1; sex: female; lifeStage: adult; occurrenceID: C834D6D2-B60E-5784-8803-5D5CC4941323; **Taxon:** scientificName: Silhouettellaloricatula (Roewer, 1942); order: Araneae; family: Oonopidae; genus: Silhouettella; **Location:** country: Italy; countryCode: IT; stateProvince: Rome; county: Rome; municipality: Rome; locality: Appia Antica Regional Park, Rome; locationRemarks: Torre Selce; decimalLatitude: 41.816611; decimalLongitude: 12.560667; geodeticDatum: WGS84; **Identification:** identifiedBy: Tommaso Fusco; dateIdentified: 2022; **Event:** samplingProtocol: Pitfall traps; eventDate: 2014-06-24; **Record Level:** collectionID: Roma3_5.8

##### Distribution

Europe to Central Asia, North Africa. Turano-Europeo-Mediterranean (TEM) chorotype.

##### Notes

Habitus in Fig. 9.

#### Philodromidae

Thorell, 1870

E96D8C3B-25F9-570B-9667-477BFE707E7A

#### Philodromus rufus

Walckenaer, 1826

7C162DC3-1676-50C6-BDF4-E92E22092250

##### Materials

- **Type status:** Other material. **Occurrence:** recordedBy: Fattorini S., Di Giulio A.; individualCount: 1; sex: female; lifeStage: adult; occurrenceID: D5B984DE-B1E7-548F-912E-7C95C634A415; **Taxon:** scientificName: Philodromusrufus Walckenaer, 1826; order: Araneae; family: Philodromidae; genus: Philodromus; **Location:** country: Italy; countryCode: IT; stateProvince: Rome; county: Rome; municipality: Rome; locality: Appia Antica Regional Park, Rome; locationRemarks: Caffarella Nord; decimalLatitude: 41.867753; decimalLongitude: 12.512414; geodeticDatum: WGS84; **Identification:** identifiedBy: Tommaso Fusco; dateIdentified: 2022; **Event:** samplingProtocol: Pitfall traps; eventDate: 2014-06-06; **Record Level:** collectionID: Roma3_5.8

##### Distribution

North America, Europe, Turkey, Caucasus, Russia (Europe to Far East), Kazakhstan, Iran, Central Asia, Mongolia, China, Korea, Japan. Holarctic (OLA) chorotype.

#### Pulchellodromus bistigma

(Simon, 1870)

971821D9-8237-534B-AC65-24C9080FF3EB

##### Materials

- **Type status:** Other material. **Occurrence:** recordedBy: Fattorini S., Di Giulio A.; individualCount: 1; sex: female; lifeStage: adult; occurrenceID: 2F2EAAF5-BA5B-5CF5-8577-447D0476B8D0; **Taxon:** scientificName: Pulchellodromusbistigma (Simon, 1870); order: Araneae; family: Philodromidae; genus: Pulchellodromus; **Location:** country: Italy; countryCode: IT; stateProvince: Rome; county: Rome; municipality: Rome; locality: Appia Antica Regional Park, Rome; locationRemarks: Casal Verbeni; decimalLatitude: 41.815250; decimalLongitude: 12.552222; geodeticDatum: WGS84; **Identification:** identifiedBy: Tommaso Fusco; dateIdentified: 2022; **Event:** samplingProtocol: Pitfall traps; eventDate: 2014-06-24; **Record Level:** collectionID: Roma3_5.8
- **Type status:** Other material. **Occurrence:** recordedBy: Fattorini S., Di Giulio A.; individualCount: 1; sex: male; lifeStage: adult; occurrenceID: 1F6A0E07-3DDC-515B-A16C-687EA3061AFC; **Taxon:** scientificName: Pulchellodromusbistigma (Simon, 1870); order: Araneae; family: Philodromidae; genus: Pulchellodromus; **Location:** country: Italy; countryCode: IT; stateProvince: Rome; county: Rome; municipality: Rome; locality: Appia Antica Regional Park, Rome; locationRemarks: Farnesiana; decimalLatitude: 41.839667; decimalLongitude: 12.525528; geodeticDatum: WGS84; **Identification:** identifiedBy: Tommaso Fusco; dateIdentified: 2022; **Event:** samplingProtocol: Pitfall traps; eventDate: 2014-05-28; **Record Level:** collectionID: Roma3_5.8
- **Type status:** Other material. **Occurrence:** recordedBy: Fattorini S., Di Giulio A.; individualCount: 1; sex: male; lifeStage: adult; occurrenceID: A16F4D49-5A0E-551D-825A-959C0F8F9988; **Taxon:** scientificName: Pulchellodromusbistigma (Simon, 1870); order: Araneae; family: Philodromidae; genus: Pulchellodromus; **Location:** country: Italy; countryCode: IT; stateProvince: Rome; county: Rome; municipality: Rome; locality: Appia Antica Regional Park, Rome; locationRemarks: Torre Selce; decimalLatitude: 41.816611; decimalLongitude: 12.560667; geodeticDatum: WGS84; **Identification:** identifiedBy: Tommaso Fusco; dateIdentified: 2022; **Event:** samplingProtocol: Pitfall traps; eventDate: 2014-06-13; **Record Level:** collectionID: Roma3_5.8
- **Type status:** Other material. **Occurrence:** recordedBy: Fattorini S., Di Giulio A.; individualCount: 1; sex: female; lifeStage: adult; occurrenceID: 63EB568E-21C0-5144-A293-C83A1EF82205; **Taxon:** scientificName: Pulchellodromusbistigma (Simon, 1870); order: Araneae; family: Philodromidae; genus: Pulchellodromus; **Location:** country: Italy; countryCode: IT; stateProvince: Rome; county: Rome; municipality: Rome; locality: Appia Antica Regional Park, Rome; locationRemarks: Caffarella Centro; decimalLatitude: 41.864889; decimalLongitude: 12.516389; geodeticDatum: WGS84; **Identification:** identifiedBy: Tommaso Fusco; dateIdentified: 2022; **Event:** samplingProtocol: Pitfall traps; eventDate: 2014-06-18; **Record Level:** collectionID: Roma3_5.8

##### Distribution

Widespread in the Mediterranean area. Mediterranean (MED) chorotype.

#### Phrurolithidae

Banks, 1892

A8F3BA16-6D86-5EB9-B95E-AEA2EB6A9D28

#### Liophrurillus flavitarsis

(Lucas, 1846)

DC16D71F-B7F6-5C62-9262-CAC31FCA05CB

##### Materials

- **Type status:** Other material. **Occurrence:** recordedBy: Fattorini S., Di Giulio A.; individualCount: 2; sex: 1 male, 1 female; lifeStage: adult; occurrenceID: 08FA2AF7-176B-51D2-8146-4C71D6F4C2B2; **Taxon:** scientificName: Liophrurillusflavitarsis (Lucas, 1846); order: Araneae; family: Phrurolithidae; genus: Liophrurillus; **Location:** country: Italy; countryCode: IT; stateProvince: Rome; county: Rome; municipality: Rome; locality: Appia Antica Regional Park, Rome; locationRemarks: Caffarella Centro; decimalLatitude: 41.864889; decimalLongitude: 12.516389; geodeticDatum: WGS84; **Identification:** identifiedBy: Tommaso Fusco; dateIdentified: 2022; **Event:** samplingProtocol: Pitfall traps; eventDate: 2014-05-19; **Record Level:** collectionID: Roma3_5.8
- **Type status:** Other material. **Occurrence:** recordedBy: Fattorini S., Di Giulio A.; individualCount: 1; sex: male; lifeStage: adult; occurrenceID: 5DE8088C-1FE0-5263-9CF8-600EEFD197BF; **Taxon:** scientificName: Liophrurillusflavitarsis (Lucas, 1846); order: Araneae; family: Phrurolithidae; genus: Liophrurillus; **Location:** country: Italy; countryCode: IT; stateProvince: Rome; county: Rome; municipality: Rome; locality: Appia Antica Regional Park, Rome; locationRemarks: Caffarella Sud 1; decimalLatitude: 41.857247; decimalLongitude: 12.529211; geodeticDatum: WGS84; **Identification:** identifiedBy: Tommaso Fusco; dateIdentified: 2022; **Event:** samplingProtocol: Pitfall traps; eventDate: 2014-05-19; **Record Level:** collectionID: Roma3_5.8
- **Type status:** Other material. **Occurrence:** recordedBy: Fattorini S., Di Giulio A.; individualCount: 1; sex: female; lifeStage: adult; occurrenceID: F5449DA8-0B46-57F2-9421-EF809B465F25; **Taxon:** scientificName: Liophrurillusflavitarsis (Lucas, 1846); order: Araneae; family: Phrurolithidae; genus: Liophrurillus; **Location:** country: Italy; countryCode: IT; stateProvince: Rome; county: Rome; municipality: Rome; locality: Appia Antica Regional Park, Rome; locationRemarks: Caffarella Sud 2; decimalLatitude: 41.856742; decimalLongitude: 12.529453; geodeticDatum: WGS84; **Identification:** identifiedBy: Tommaso Fusco; dateIdentified: 2022; **Event:** samplingProtocol: Pitfall traps; eventDate: 2014-05-19; **Record Level:** collectionID: Roma3_5.8
- **Type status:** Other material. **Occurrence:** recordedBy: Fattorini S., Di Giulio A.; individualCount: 1; sex: female; lifeStage: adult; occurrenceID: 6E8F88D6-F58B-5663-B66F-A55282B3FA69; **Taxon:** scientificName: Liophrurillusflavitarsis (Lucas, 1846); order: Araneae; family: Phrurolithidae; genus: Liophrurillus; **Location:** country: Italy; countryCode: IT; stateProvince: Rome; county: Rome; municipality: Rome; locality: Appia Antica Regional Park, Rome; locationRemarks: Caffarella Sud 3; decimalLatitude: 41.856928; decimalLongitude: 12.528406; geodeticDatum: WGS84; **Identification:** identifiedBy: Tommaso Fusco; dateIdentified: 2022; **Event:** samplingProtocol: Pitfall traps; eventDate: 2014-05-19; **Record Level:** collectionID: Roma3_5.8
- **Type status:** Other material. **Occurrence:** recordedBy: Fattorini S., Di Giulio A.; individualCount: 3; sex: female; lifeStage: adult; occurrenceID: 07DCD9C0-F4D9-5D59-B96A-1633C69330C0; **Taxon:** scientificName: Liophrurillusflavitarsis (Lucas, 1846); order: Araneae; family: Phrurolithidae; genus: Liophrurillus; **Location:** country: Italy; countryCode: IT; stateProvince: Rome; county: Rome; municipality: Rome; locality: Appia Antica Regional Park, Rome; locationRemarks: Casal Verbeni; decimalLatitude: 41.815250; decimalLongitude: 12.552222; geodeticDatum: WGS84; **Identification:** identifiedBy: Tommaso Fusco; dateIdentified: 2022; **Event:** samplingProtocol: Pitfall traps; eventDate: 2014-05-26; **Record Level:** collectionID: Roma3_5.8
- **Type status:** Other material. **Occurrence:** recordedBy: Fattorini S., Di Giulio A.; individualCount: 3; sex: female; lifeStage: adult; occurrenceID: 14EE79F3-516E-5CB0-A4A8-8E04965755F3; **Taxon:** scientificName: Liophrurillusflavitarsis (Lucas, 1846); order: Araneae; family: Phrurolithidae; genus: Liophrurillus; **Location:** country: Italy; countryCode: IT; stateProvince: Rome; county: Rome; municipality: Rome; locality: Appia Antica Regional Park, Rome; locationRemarks: Farnesiana; decimalLatitude: 41.839667; decimalLongitude: 12.525528; geodeticDatum: WGS84; **Identification:** identifiedBy: Tommaso Fusco; dateIdentified: 2022; **Event:** samplingProtocol: Pitfall traps; eventDate: 2014-05-20; **Record Level:** collectionID: Roma3_5.8
- **Type status:** Other material. **Occurrence:** recordedBy: Fattorini S., Di Giulio A.; individualCount: 1; sex: female; lifeStage: adult; occurrenceID: 0FC07D93-5B7A-588F-BD55-5E1413DA7F17; **Taxon:** scientificName: Liophrurillusflavitarsis (Lucas, 1846); order: Araneae; family: Phrurolithidae; genus: Liophrurillus; **Location:** country: Italy; countryCode: IT; stateProvince: Rome; county: Rome; municipality: Rome; locality: Appia Antica Regional Park, Rome; locationRemarks: San Sebastiano; decimalLatitude: 41.855733; decimalLongitude: 12.515114; geodeticDatum: WGS84; **Identification:** identifiedBy: Tommaso Fusco; dateIdentified: 2022; **Event:** samplingProtocol: Pitfall traps; eventDate: 2014-05-20; **Record Level:** collectionID: Roma3_5.8
- **Type status:** Other material. **Occurrence:** recordedBy: Fattorini S., Di Giulio A.; individualCount: 2; sex: 1 male, 1 female; lifeStage: adult; occurrenceID: ABB73C77-E722-5DA4-AFB0-8023D1C3CD26; **Taxon:** scientificName: Liophrurillusflavitarsis (Lucas, 1846); order: Araneae; family: Phrurolithidae; genus: Liophrurillus; **Location:** country: Italy; countryCode: IT; stateProvince: Rome; county: Rome; municipality: Rome; locality: Appia Antica Regional Park, Rome; locationRemarks: Tor Marancia; decimalLatitude: 41.850308; decimalLongitude: 12.503178; geodeticDatum: WGS84; **Identification:** identifiedBy: Tommaso Fusco; dateIdentified: 2022; **Event:** samplingProtocol: Pitfall traps; eventDate: 2014-05-15; **Record Level:** collectionID: Roma3_5.8
- **Type status:** Other material. **Occurrence:** recordedBy: Fattorini S., Di Giulio A.; individualCount: 1; sex: female; lifeStage: adult; occurrenceID: 32C07185-84D3-5705-AD37-8600E7C31E6C; **Taxon:** scientificName: Liophrurillusflavitarsis (Lucas, 1846); order: Araneae; family: Phrurolithidae; genus: Liophrurillus; **Location:** country: Italy; countryCode: IT; stateProvince: Rome; county: Rome; municipality: Rome; locality: Appia Antica Regional Park, Rome; locationRemarks: Torre Selce; decimalLatitude: 41.816611; decimalLongitude: 12.560667; geodeticDatum: WGS84; **Identification:** identifiedBy: Tommaso Fusco; dateIdentified: 2022; **Event:** samplingProtocol: Pitfall traps; eventDate: 2014-05-26; **Record Level:** collectionID: Roma3_5.8
- **Type status:** Other material. **Occurrence:** recordedBy: Fattorini S., Di Giulio A.; individualCount: 1; sex: female; lifeStage: adult; occurrenceID: 6B8534F9-80EF-55D9-8215-B9697F196645; **Taxon:** scientificName: Liophrurillusflavitarsis (Lucas, 1846); order: Araneae; family: Phrurolithidae; genus: Liophrurillus; **Location:** country: Italy; countryCode: IT; stateProvince: Rome; county: Rome; municipality: Rome; locality: Appia Antica Regional Park, Rome; locationRemarks: Acqua Santa; decimalLatitude: 41.850561; decimalLongitude: 12.530861; geodeticDatum: WGS84; **Identification:** identifiedBy: Tommaso Fusco; dateIdentified: 2022; **Event:** samplingProtocol: Pitfall traps; eventDate: 2014-05-28; **Record Level:** collectionID: Roma3_5.8
- **Type status:** Other material. **Occurrence:** recordedBy: Fattorini S., Di Giulio A.; individualCount: 3; sex: 1 male, 2 female; lifeStage: adult; occurrenceID: 1232DD84-5705-50A5-B64A-A8989C9B348E; **Taxon:** scientificName: Liophrurillusflavitarsis (Lucas, 1846); order: Araneae; family: Phrurolithidae; genus: Liophrurillus; **Location:** country: Italy; countryCode: IT; stateProvince: Rome; county: Rome; municipality: Rome; locality: Appia Antica Regional Park, Rome; locationRemarks: Appia Antica; decimalLatitude: 41.812575; decimalLongitude: 12.564011; geodeticDatum: WGS84; **Identification:** identifiedBy: Tommaso Fusco; dateIdentified: 2022; **Event:** samplingProtocol: Pitfall traps; eventDate: 2014-06-05; **Record Level:** collectionID: Roma3_5.8
- **Type status:** Other material. **Occurrence:** recordedBy: Fattorini S., Di Giulio A.; individualCount: 2; sex: 1 male, 1 female; lifeStage: adult; occurrenceID: 026D17ED-5CEE-5210-AC8F-35E82F1F379C; **Taxon:** scientificName: Liophrurillusflavitarsis (Lucas, 1846); order: Araneae; family: Phrurolithidae; genus: Liophrurillus; **Location:** country: Italy; countryCode: IT; stateProvince: Rome; county: Rome; municipality: Rome; locality: Appia Antica Regional Park, Rome; locationRemarks: Caffarella Centro; decimalLatitude: 41.864889; decimalLongitude: 12.516389; geodeticDatum: WGS84; **Identification:** identifiedBy: Tommaso Fusco; dateIdentified: 2022; **Event:** samplingProtocol: Pitfall traps; eventDate: 2014-05-27; **Record Level:** collectionID: Roma3_5.8
- **Type status:** Other material. **Occurrence:** recordedBy: Fattorini S., Di Giulio A.; individualCount: 2; sex: female; lifeStage: adult; occurrenceID: 2B24D6FB-9913-597E-A133-6EE20B06C209; **Taxon:** scientificName: Liophrurillusflavitarsis (Lucas, 1846); order: Araneae; family: Phrurolithidae; genus: Liophrurillus; **Location:** country: Italy; countryCode: IT; stateProvince: Rome; county: Rome; municipality: Rome; locality: Appia Antica Regional Park, Rome; locationRemarks: Casal Verbeni; decimalLatitude: 41.815250; decimalLongitude: 12.552222; geodeticDatum: WGS84; **Identification:** identifiedBy: Tommaso Fusco; dateIdentified: 2022; **Event:** samplingProtocol: Pitfall traps; eventDate: 2014-06-05; **Record Level:** collectionID: Roma3_5.8
- **Type status:** Other material. **Occurrence:** recordedBy: Fattorini S., Di Giulio A.; individualCount: 2; sex: 1 male, 1 female; lifeStage: adult; occurrenceID: 75EB2700-69C9-5F95-A688-CF30589915BC; **Taxon:** scientificName: Liophrurillusflavitarsis (Lucas, 1846); order: Araneae; family: Phrurolithidae; genus: Liophrurillus; **Location:** country: Italy; countryCode: IT; stateProvince: Rome; county: Rome; municipality: Rome; locality: Appia Antica Regional Park, Rome; locationRemarks: Cava Fiorucci; decimalLatitude: 41.834106; decimalLongitude: 12.549264; geodeticDatum: WGS84; **Identification:** identifiedBy: Tommaso Fusco; dateIdentified: 2022; **Event:** samplingProtocol: Pitfall traps; eventDate: 2014-05-26; **Record Level:** collectionID: Roma3_5.8
- **Type status:** Other material. **Occurrence:** recordedBy: Fattorini S., Di Giulio A.; individualCount: 1; sex: male; lifeStage: adult; occurrenceID: 714D7E73-FF09-58D5-BC78-3C5703597A6B; **Taxon:** scientificName: Liophrurillusflavitarsis (Lucas, 1846); order: Araneae; family: Phrurolithidae; genus: Liophrurillus; **Location:** country: Italy; countryCode: IT; stateProvince: Rome; county: Rome; municipality: Rome; locality: Appia Antica Regional Park, Rome; locationRemarks: Cava Fiorucci; decimalLatitude: 41.834106; decimalLongitude: 12.549264; geodeticDatum: WGS84; **Identification:** identifiedBy: Tommaso Fusco; dateIdentified: 2022; **Event:** samplingProtocol: Pitfall traps; eventDate: 2013-11-18; **Record Level:** collectionID: Roma3_5.8
- **Type status:** Other material. **Occurrence:** recordedBy: Fattorini S., Di Giulio A.; individualCount: 4; sex: 1 male, 3 female; lifeStage: adult; occurrenceID: BB037ABC-42B5-582C-A805-C8B438446AC9; **Taxon:** scientificName: Liophrurillusflavitarsis (Lucas, 1846); order: Araneae; family: Phrurolithidae; genus: Liophrurillus; **Location:** country: Italy; countryCode: IT; stateProvince: Rome; county: Rome; municipality: Rome; locality: Appia Antica Regional Park, Rome; locationRemarks: Farnesiana; decimalLatitude: 41.839667; decimalLongitude: 12.525528; geodeticDatum: WGS84; **Identification:** identifiedBy: Tommaso Fusco; dateIdentified: 2022; **Event:** samplingProtocol: Pitfall traps; eventDate: 2014-05-28; **Record Level:** collectionID: Roma3_5.8
- **Type status:** Other material. **Occurrence:** recordedBy: Fattorini S., Di Giulio A.; individualCount: 2; sex: female; lifeStage: adult; occurrenceID: BC3A54F1-1CF1-5AF2-B652-1AC1C96ABB10; **Taxon:** scientificName: Liophrurillusflavitarsis (Lucas, 1846); order: Araneae; family: Phrurolithidae; genus: Liophrurillus; **Location:** country: Italy; countryCode: IT; stateProvince: Rome; county: Rome; municipality: Rome; locality: Appia Antica Regional Park, Rome; locationRemarks: San Sebastiano; decimalLatitude: 41.855733; decimalLongitude: 12.515114; geodeticDatum: WGS84; **Identification:** identifiedBy: Tommaso Fusco; dateIdentified: 2022; **Event:** samplingProtocol: Pitfall traps; eventDate: 2014-05-28; **Record Level:** collectionID: Roma3_5.8
- **Type status:** Other material. **Occurrence:** recordedBy: Fattorini S., Di Giulio A.; individualCount: 1; sex: female; lifeStage: adult; occurrenceID: 46145692-1B0C-5322-8A52-976E785DB5D3; **Taxon:** scientificName: Liophrurillusflavitarsis (Lucas, 1846); order: Araneae; family: Phrurolithidae; genus: Liophrurillus; **Location:** country: Italy; countryCode: IT; stateProvince: Rome; county: Rome; municipality: Rome; locality: Appia Antica Regional Park, Rome; locationRemarks: Tor marancia; decimalLatitude: 41.850308; decimalLongitude: 12.503178; geodeticDatum: WGS84; **Identification:** identifiedBy: Tommaso Fusco; dateIdentified: 2022; **Event:** samplingProtocol: Pitfall traps; eventDate: 2014-05-26; **Record Level:** collectionID: Roma3_5.8
- **Type status:** Other material. **Occurrence:** recordedBy: Fattorini S., Di Giulio A.; individualCount: 1; sex: female; lifeStage: adult; occurrenceID: 6225A477-6450-50D5-87BC-85DC8255E2E1; **Taxon:** scientificName: Liophrurillusflavitarsis (Lucas, 1846); order: Araneae; family: Phrurolithidae; genus: Liophrurillus; **Location:** country: Italy; countryCode: IT; stateProvince: Rome; county: Rome; municipality: Rome; locality: Appia Antica Regional Park, Rome; locationRemarks: Acqua santa; decimalLatitude: 41.850561; decimalLongitude: 12.530861; geodeticDatum: WGS84; **Identification:** identifiedBy: Tommaso Fusco; dateIdentified: 2022; **Event:** samplingProtocol: Pitfall traps; eventDate: 2014-06-09; **Record Level:** collectionID: Roma3_5.8
- **Type status:** Other material. **Occurrence:** recordedBy: Fattorini S., Di Giulio A.; individualCount: 2; sex: female; lifeStage: adult; occurrenceID: E8B3B229-09EB-5CEC-83F0-91053B298E70; **Taxon:** scientificName: Liophrurillusflavitarsis (Lucas, 1846); order: Araneae; family: Phrurolithidae; genus: Liophrurillus; **Location:** country: Italy; countryCode: IT; stateProvince: Rome; county: Rome; municipality: Rome; locality: Appia Antica Regional Park, Rome; locationRemarks: Appia Antica; decimalLatitude: 41.812575; decimalLongitude: 12.564011; geodeticDatum: WGS84; **Identification:** identifiedBy: Tommaso Fusco; dateIdentified: 2022; **Event:** samplingProtocol: Pitfall traps; eventDate: 2014-06-13; **Record Level:** collectionID: Roma3_5.8
- **Type status:** Other material. **Occurrence:** recordedBy: Fattorini S., Di Giulio A.; individualCount: 3; sex: female; lifeStage: adult; occurrenceID: 08AC8D15-9830-5DDF-8754-7BD3DBBB69DA; **Taxon:** scientificName: Liophrurillusflavitarsis (Lucas, 1846); order: Araneae; family: Phrurolithidae; genus: Liophrurillus; **Location:** country: Italy; countryCode: IT; stateProvince: Rome; county: Rome; municipality: Rome; locality: Appia Antica Regional Park, Rome; locationRemarks: Caffarella Centro; decimalLatitude: 41.864889; decimalLongitude: 12.516389; geodeticDatum: WGS84; **Identification:** identifiedBy: Tommaso Fusco; dateIdentified: 2022; **Event:** samplingProtocol: Pitfall traps; eventDate: 2014-06-06; **Record Level:** collectionID: Roma3_5.8
- **Type status:** Other material. **Occurrence:** recordedBy: Fattorini S., Di Giulio A.; individualCount: 1; sex: female; lifeStage: adult; occurrenceID: 73E1AF83-CE2E-5827-82A3-CE5386CA6B2C; **Taxon:** scientificName: Liophrurillusflavitarsis (Lucas, 1846); order: Araneae; family: Phrurolithidae; genus: Liophrurillus; **Location:** country: Italy; countryCode: IT; stateProvince: Rome; county: Rome; municipality: Rome; locality: Appia Antica Regional Park, Rome; locationRemarks: Farnesiana; decimalLatitude: 41.839667; decimalLongitude: 12.525528; geodeticDatum: WGS84; **Identification:** identifiedBy: Tommaso Fusco; dateIdentified: 2022; **Event:** samplingProtocol: Pitfall traps; eventDate: 2014-06-09; **Record Level:** collectionID: Roma3_5.8
- **Type status:** Other material. **Occurrence:** recordedBy: Fattorini S., Di Giulio A.; individualCount: 4; sex: female; lifeStage: adult; occurrenceID: DEAF81EF-FBFC-5DC6-B2FE-444FFF0229E6; **Taxon:** scientificName: Liophrurillusflavitarsis (Lucas, 1846); order: Araneae; family: Phrurolithidae; genus: Liophrurillus; **Location:** country: Italy; countryCode: IT; stateProvince: Rome; county: Rome; municipality: Rome; locality: Appia Antica Regional Park, Rome; locationRemarks: Tor Marancia; decimalLatitude: 41.850308; decimalLongitude: 12.503178; geodeticDatum: WGS84; **Identification:** identifiedBy: Tommaso Fusco; dateIdentified: 2022; **Event:** samplingProtocol: Pitfall traps; eventDate: 2014-06-05; **Record Level:** collectionID: Roma3_5.8
- **Type status:** Other material. **Occurrence:** recordedBy: Fattorini S., Di Giulio A.; individualCount: 2; sex: female; lifeStage: adult; occurrenceID: 34A4F131-C3A9-51C7-BEE8-03B7C512F6DA; **Taxon:** scientificName: Liophrurillusflavitarsis (Lucas, 1846); order: Araneae; family: Phrurolithidae; genus: Liophrurillus; **Location:** country: Italy; countryCode: IT; stateProvince: Rome; county: Rome; municipality: Rome; locality: Appia Antica Regional Park, Rome; locationRemarks: Acqua Santa; decimalLatitude: 41.850561; decimalLongitude: 12.530861; geodeticDatum: WGS84; **Identification:** identifiedBy: Tommaso Fusco; dateIdentified: 2022; **Event:** samplingProtocol: Pitfall traps; eventDate: 2014-06-19; **Record Level:** collectionID: Roma3_5.8
- **Type status:** Other material. **Occurrence:** recordedBy: Fattorini S., Di Giulio A.; individualCount: 2; sex: female; lifeStage: adult; occurrenceID: 2C254E41-6106-5E22-A33C-4CC542860963; **Taxon:** scientificName: Liophrurillusflavitarsis (Lucas, 1846); order: Araneae; family: Phrurolithidae; genus: Liophrurillus; **Location:** country: Italy; countryCode: IT; stateProvince: Rome; county: Rome; municipality: Rome; locality: Appia Antica Regional Park, Rome; locationRemarks: Caffarella Centro; decimalLatitude: 41.864889; decimalLongitude: 12.516389; geodeticDatum: WGS84; **Identification:** identifiedBy: Tommaso Fusco; dateIdentified: 2022; **Event:** samplingProtocol: Pitfall traps; eventDate: 2014-06-18; **Record Level:** collectionID: Roma3_5.8
- **Type status:** Other material. **Occurrence:** recordedBy: Fattorini S., Di Giulio A.; individualCount: 1; sex: female; lifeStage: adult; occurrenceID: BF5C8BC2-EB84-51C4-AAD3-454F0EC2195C; **Taxon:** scientificName: Liophrurillusflavitarsis (Lucas, 1846); order: Araneae; family: Phrurolithidae; genus: Liophrurillus; **Location:** country: Italy; countryCode: IT; stateProvince: Rome; county: Rome; municipality: Rome; locality: Appia Antica Regional Park, Rome; locationRemarks: Caffarella Sud 1; decimalLatitude: 41.857247; decimalLongitude: 12.529211; geodeticDatum: WGS84; **Identification:** identifiedBy: Tommaso Fusco; dateIdentified: 2022; **Event:** samplingProtocol: Pitfall traps; eventDate: 2014-06-18; **Record Level:** collectionID: Roma3_5.8
- **Type status:** Other material. **Occurrence:** recordedBy: Fattorini S., Di Giulio A.; individualCount: 1; sex: female; lifeStage: adult; occurrenceID: DD0FF776-244E-5E6C-A5EF-49A6D022DC77; **Taxon:** scientificName: Liophrurillusflavitarsis (Lucas, 1846); order: Araneae; family: Phrurolithidae; genus: Liophrurillus; **Location:** country: Italy; countryCode: IT; stateProvince: Rome; county: Rome; municipality: Rome; locality: Appia Antica Regional Park, Rome; locationRemarks: Caffarella Sud 2; decimalLatitude: 41.856742; decimalLongitude: 12.529453; geodeticDatum: WGS84; **Identification:** identifiedBy: Tommaso Fusco; dateIdentified: 2022; **Event:** samplingProtocol: Pitfall traps; eventDate: 2014-06-18; **Record Level:** collectionID: Roma3_5.8
- **Type status:** Other material. **Occurrence:** recordedBy: Fattorini S., Di Giulio A.; individualCount: 1; sex: female; lifeStage: adult; occurrenceID: 63D889EA-F685-5F31-98E4-02746D54FCB6; **Taxon:** scientificName: Liophrurillusflavitarsis (Lucas, 1846); order: Araneae; family: Phrurolithidae; genus: Liophrurillus; **Location:** country: Italy; countryCode: IT; stateProvince: Rome; county: Rome; municipality: Rome; locality: Appia Antica Regional Park, Rome; locationRemarks: Casal Verbeni; decimalLatitude: 41.815250; decimalLongitude: 12.552222; geodeticDatum: WGS84; **Identification:** identifiedBy: Tommaso Fusco; dateIdentified: 2022; **Event:** samplingProtocol: Pitfall traps; eventDate: 2014-06-24; **Record Level:** collectionID: Roma3_5.8
- **Type status:** Other material. **Occurrence:** recordedBy: Fattorini S., Di Giulio A.; individualCount: 2; sex: female; lifeStage: adult; occurrenceID: E1C6FE6B-9F6B-5D47-9428-96C20BE39BB0; **Taxon:** scientificName: Liophrurillusflavitarsis (Lucas, 1846); order: Araneae; family: Phrurolithidae; genus: Liophrurillus; **Location:** country: Italy; countryCode: IT; stateProvince: Rome; county: Rome; municipality: Rome; locality: Appia Antica Regional Park, Rome; locationRemarks: Farnesiana; decimalLatitude: 41.839667; decimalLongitude: 12.525528; geodeticDatum: WGS84; **Identification:** identifiedBy: Tommaso Fusco; dateIdentified: 2022; **Event:** samplingProtocol: Pitfall traps; eventDate: 2014-06-19; **Record Level:** collectionID: Roma3_5.8
- **Type status:** Other material. **Occurrence:** recordedBy: Fattorini S., Di Giulio A.; individualCount: 1; sex: female; lifeStage: adult; occurrenceID: 8BAD2C06-7627-5D4D-BECB-E438B8145B9C; **Taxon:** scientificName: Liophrurillusflavitarsis (Lucas, 1846); order: Araneae; family: Phrurolithidae; genus: Liophrurillus; **Location:** country: Italy; countryCode: IT; stateProvince: Rome; county: Rome; municipality: Rome; locality: Appia Antica Regional Park, Rome; locationRemarks: Tor Marancia; decimalLatitude: 41.850308; decimalLongitude: 12.503178; geodeticDatum: WGS84; **Identification:** identifiedBy: Tommaso Fusco; dateIdentified: 2022; **Event:** samplingProtocol: Pitfall traps; eventDate: 2014-06-17; **Record Level:** collectionID: Roma3_5.8

##### Distribution

SW Europe, North Africa; also quoted from Romania. W-Mediterranean (WME) chorotype.

#### Phrurolithus minimus

C. L. Koch, 1839

6042DEEA-E7AD-59C9-B16D-2CC754F2332F

##### Materials

- **Type status:** Other material. **Occurrence:** recordedBy: Fattorini S., Di Giulio A.; individualCount: 1; sex: male; lifeStage: adult; occurrenceID: 6480CD2F-DA50-51A5-BB76-C255E1BD4841; **Taxon:** scientificName: Phrurolithusminimus C. L. Koch, 1839; order: Araneae; family: Phrurolithidae; genus: Phrurolithus; **Location:** country: Italy; countryCode: IT; stateProvince: Rome; county: Rome; municipality: Rome; locality: Appia Antica Regional Park, Rome; locationRemarks: Caffarella Centro; decimalLatitude: 41.864889; decimalLongitude: 12.516389; geodeticDatum: WGS84; **Identification:** identifiedBy: Tommaso Fusco; dateIdentified: 2022; **Event:** samplingProtocol: Pitfall; eventDate: 2014-05-19; **Record Level:** collectionID: Roma3_5.8
- **Type status:** Other material. **Occurrence:** recordedBy: Fattorini S., Di Giulio A.; individualCount: 1; sex: male; lifeStage: adult; occurrenceID: E8E7F7E7-DFEE-5517-8FCC-68505C867A64; **Taxon:** scientificName: Phrurolithusminimus C. L. Koch, 1839; order: Araneae; family: Phrurolithidae; genus: Phrurolithus; **Location:** country: Italy; countryCode: IT; stateProvince: Rome; county: Rome; municipality: Rome; locality: Appia Antica Regional Park, Rome; locationRemarks: Caffarella Sud; decimalLatitude: 41.857247; decimalLongitude: 12.529211; geodeticDatum: WGS84; **Identification:** identifiedBy: Tommaso Fusco; dateIdentified: 2022; **Event:** samplingProtocol: Pitfall; eventDate: 2014-05-27; **Record Level:** collectionID: Roma3_5.8
- **Type status:** Other material. **Occurrence:** recordedBy: Fattorini S., Di Giulio A.; individualCount: 1; sex: male; lifeStage: adult; occurrenceID: 7DAD5818-2C45-5079-A8E0-1F99C0279251; **Taxon:** scientificName: Phrurolithusminimus C. L. Koch, 1839; order: Araneae; family: Phrurolithidae; genus: Phrurolithus; **Location:** country: Italy; countryCode: IT; stateProvince: Rome; county: Rome; municipality: Rome; locality: Appia Antica Regional Park, Rome; locationRemarks: Caffarella Sud 2; decimalLatitude: 41.856742; decimalLongitude: 12.529453; geodeticDatum: WGS84; **Identification:** identifiedBy: Tommaso Fusco; dateIdentified: 2022; **Event:** samplingProtocol: Pitfall; eventDate: 2014-06-06; **Record Level:** collectionID: Roma3_5.8
- **Type status:** Other material. **Occurrence:** recordedBy: Fattorini S., Di Giulio A.; individualCount: 3; sex: 2 male, 1 female; lifeStage: adult; occurrenceID: DC188292-4A7B-5704-8028-6006A1CAE64F; **Taxon:** scientificName: Phrurolithusminimus C. L. Koch, 1839; order: Araneae; family: Phrurolithidae; genus: Phrurolithus; **Location:** country: Italy; countryCode: IT; stateProvince: Rome; county: Rome; municipality: Rome; locality: Appia Antica Regional Park, Rome; locationRemarks: Caffarella Sud 2; decimalLatitude: 41.856742; decimalLongitude: 12.529453; geodeticDatum: WGS84; **Identification:** identifiedBy: Tommaso Fusco; dateIdentified: 2022; **Event:** samplingProtocol: Pitfall; eventDate: 2014-06-18; **Record Level:** collectionID: Roma3_5.8
- **Type status:** Other material. **Occurrence:** recordedBy: Fattorini S., Di Giulio A.; individualCount: 4; sex: 3 male, 1 female; lifeStage: adult; occurrenceID: DB7EAF66-84C4-5258-AF25-E712D9C5A487; **Taxon:** scientificName: Phrurolithusminimus C. L. Koch, 1839; order: Araneae; family: Phrurolithidae; genus: Phrurolithus; **Location:** country: Italy; countryCode: IT; stateProvince: Rome; county: Rome; municipality: Rome; locality: Appia Antica Regional Park, Rome; locationRemarks: Tor Marancia; decimalLatitude: 41.850308; decimalLongitude: 12.503178; geodeticDatum: WGS84; **Identification:** identifiedBy: Tommaso Fusco; dateIdentified: 2022; **Event:** samplingProtocol: Pitfall; eventDate: 2014-05-26; **Record Level:** collectionID: Roma3_5.8
- **Type status:** Other material. **Occurrence:** recordedBy: Fattorini S., Di Giulio A.; individualCount: 6; sex: 5 male, 1 female; lifeStage: adult; occurrenceID: 14D7024B-B58B-5252-BE81-36FD77D00EE3; **Taxon:** scientificName: Phrurolithusminimus C. L. Koch, 1839; order: Araneae; family: Phrurolithidae; genus: Phrurolithus; **Location:** country: Italy; countryCode: IT; stateProvince: Rome; county: Rome; municipality: Rome; locality: Appia Antica Regional Park, Rome; locationRemarks: Tor Marancia; decimalLatitude: 41.850308; decimalLongitude: 12.503178; geodeticDatum: WGS84; **Identification:** identifiedBy: Tommaso Fusco; dateIdentified: 2022; **Event:** samplingProtocol: Pitfall; eventDate: 2014-06-05; **Record Level:** collectionID: Roma3_5.8
- **Type status:** Other material. **Occurrence:** recordedBy: Fattorini S., Di Giulio A.; individualCount: 10; sex: 9 male, 1 female; lifeStage: adult; occurrenceID: 73C8F060-A9B1-5441-88DD-4BE42609AF62; **Taxon:** scientificName: Phrurolithusminimus C. L. Koch, 1839; order: Araneae; family: Phrurolithidae; genus: Phrurolithus; **Location:** country: Italy; countryCode: IT; stateProvince: Rome; county: Rome; municipality: Rome; locality: Appia Antica Regional Park, Rome; locationRemarks: Tor Marancia; decimalLatitude: 41.850308; decimalLongitude: 12.503178; geodeticDatum: WGS84; **Identification:** identifiedBy: Tommaso Fusco; dateIdentified: 2022; **Event:** samplingProtocol: Pitfall; eventDate: 2014-06-17; **Record Level:** collectionID: Roma3_5.8
- **Type status:** Other material. **Occurrence:** recordedBy: Fattorini S., Di Giulio A.; individualCount: 2; sex: male; lifeStage: adult; occurrenceID: 5635FAE5-70F6-5EBF-A9D4-65F0561056C6; **Taxon:** scientificName: Phrurolithusminimus C. L. Koch, 1839; order: Araneae; family: Phrurolithidae; genus: Phrurolithus; **Location:** country: Italy; countryCode: IT; stateProvince: Rome; county: Rome; municipality: Rome; locality: Appia Antica Regional Park, Rome; locationRemarks: Tor Marancia; decimalLatitude: 41.850308; decimalLongitude: 12.503178; geodeticDatum: WGS84; **Identification:** identifiedBy: Tommaso Fusco; dateIdentified: 2022; **Event:** samplingProtocol: Pitfall; eventDate: 2014-05-15; **Record Level:** collectionID: Roma3_5.8
- **Type status:** Other material. **Occurrence:** recordedBy: Fattorini S., Di Giulio A.; individualCount: 1; sex: female; lifeStage: adult; occurrenceID: 9644E5D0-1016-5AB8-B7D5-4FEBC4191FA0; **Taxon:** scientificName: Phrurolithusminimus C. L. Koch, 1839; order: Araneae; family: Phrurolithidae; genus: Phrurolithus; **Location:** country: Italy; countryCode: IT; stateProvince: Rome; county: Rome; municipality: Rome; locality: Appia Antica Regional Park, Rome; locationRemarks: Tor Marancia; decimalLatitude: 41.850308; decimalLongitude: 12.503178; geodeticDatum: WGS84; **Identification:** identifiedBy: Tommaso Fusco; dateIdentified: 2022; **Event:** samplingProtocol: Pitfall; eventDate: 2013-10-28; **Record Level:** collectionID: Roma3_5.8
- **Type status:** Other material. **Occurrence:** recordedBy: Fattorini S., Di Giulio A.; individualCount: 1; sex: female; lifeStage: adult; occurrenceID: 38A954E9-8189-5FE4-85B9-5D0E43862496; **Taxon:** scientificName: Phrurolithusminimus C. L. Koch, 1839; order: Araneae; family: Phrurolithidae; genus: Phrurolithus; **Location:** country: Italy; countryCode: IT; stateProvince: Rome; county: Rome; municipality: Rome; locality: Appia Antica Regional Park, Rome; locationRemarks: Tor Marancia; decimalLatitude: 41.850308; decimalLongitude: 12.503178; geodeticDatum: WGS84; **Identification:** identifiedBy: Tommaso Fusco; dateIdentified: 2022; **Event:** samplingProtocol: Pitfall; eventDate: 2014-05-26; **Record Level:** collectionID: Roma3_5.8
- **Type status:** Other material. **Occurrence:** recordedBy: Fattorini S., Di Giulio A.; individualCount: 1; sex: female; lifeStage: adult; occurrenceID: 0C726D05-5EEB-50F7-B3BD-7F15CB11F16C; **Taxon:** scientificName: Phrurolithusminimus C. L. Koch, 1839; order: Araneae; family: Phrurolithidae; genus: Phrurolithus; **Location:** country: Italy; countryCode: IT; stateProvince: Rome; county: Rome; municipality: Rome; locality: Appia Antica Regional Park, Rome; locationRemarks: Cava Fiorucci; decimalLatitude: 41.834106; decimalLongitude: 12.549264; geodeticDatum: WGS84; **Identification:** identifiedBy: Tommaso Fusco; dateIdentified: 2022; **Event:** samplingProtocol: Pitfall; eventDate: 2014-06-24; **Record Level:** collectionID: Roma3_5.8
- **Type status:** Other material. **Occurrence:** recordedBy: Fattorini S., Di Giulio A.; individualCount: 1; sex: female; lifeStage: adult; occurrenceID: 6F398022-756B-539E-8281-9BE8762C344D; **Taxon:** scientificName: Phrurolithusminimus C. L. Koch, 1839; order: Araneae; family: Phrurolithidae; genus: Phrurolithus; **Location:** country: Italy; countryCode: IT; stateProvince: Rome; county: Rome; municipality: Rome; locality: Appia Antica Regional Park, Rome; locationRemarks: Caffarella Nord; decimalLatitude: 41.867753; decimalLongitude: 12.512414; geodeticDatum: WGS84; **Identification:** identifiedBy: Tommaso Fusco; dateIdentified: 2022; **Event:** samplingProtocol: Pitfall; eventDate: 2014-06-18; **Record Level:** collectionID: Roma3_5.8

##### Distribution

Most of Europe. European (EUR) chorotype.

#### Salticidae

Blackwall, 1841

B4896C9E-DB6A-5D33-92EB-E11A3CEAF62B

#### Aelurilus v-insignitus

(Clerck, 1757)

65EA4B69-E99B-5296-9610-99082DAA7C67

##### Materials

- **Type status:** Other material. **Occurrence:** recordedBy: Fattorini S., Di Giulio A.; individualCount: 1; sex: male; lifeStage: adult; occurrenceID: 51E35146-FE0E-52C0-8586-46720AA7B2DB; **Taxon:** scientificName: Aelurillus v-insignitus (Clerck, 1757); order: Araneae; family: Salticidae; genus: Aelurillus; **Location:** country: Italy; countryCode: IT; stateProvince: Rome; county: Rome; municipality: Rome; locality: Appia Antica regional Park, Rome; locationRemarks: Casal Verbeni; decimalLatitude: 41.815250; decimalLongitude: 12.552222; geodeticDatum: WGS84; **Identification:** identifiedBy: Tommaso Fusco; dateIdentified: 2022; **Event:** samplingProtocol: Pitfall traps; eventDate: 2014-05-26; **Record Level:** collectionID: Roma3_5.8

##### Distribution

Europe, Turkey, Caucasus, Russia (Europe to Far East), Kazakhstan, Central Asia, China. Palaearctic (PAL) chorotype.

#### Euophrys frontalis

(Walckenaer, 1802)

4869B754-B552-5548-AEEF-44D6B5132345

##### Materials

- **Type status:** Other material. **Occurrence:** recordedBy: Fattorini S., Di Giulio A.; individualCount: 1; sex: male; lifeStage: adult; occurrenceID: AE5D0814-294F-5B3A-83C6-DE4BA3E09835; **Taxon:** scientificName: Euophrysfrontalis (Walckenaer, 1802); order: Araneae; family: Salticidae; genus: Euophrys; **Location:** country: Italy; countryCode: IT; stateProvince: Rome; county: Rome; municipality: Rome; locality: Appia Antica Regional Park, Rome; locationRemarks: Tor marancia; decimalLatitude: 41.850308; decimalLongitude: 12.503178; geodeticDatum: WGS84; **Identification:** identifiedBy: Tommaso Fusco; dateIdentified: 2022; **Event:** samplingProtocol: Pitfall traps; eventDate: 2014-05-26; **Record Level:** collectionID: Roma3_5.8
- **Type status:** Other material. **Occurrence:** recordedBy: Fattorini S., Di Giulio A.; individualCount: 2; sex: 1 male, 1 female; lifeStage: adult; occurrenceID: DC1D25A3-50F3-5C41-BAA9-A0443B64FF09; **Taxon:** scientificName: Euophrysfrontalis (Walckenaer, 1802); order: Araneae; family: Salticidae; genus: Euophrys; **Location:** country: Italy; countryCode: IT; stateProvince: Rome; county: Rome; municipality: Rome; locality: Appia Antica Regional Park, Rome; locationRemarks: Tor Marancia; decimalLatitude: 41.850308; decimalLongitude: 12.503178; geodeticDatum: WGS84; **Identification:** identifiedBy: Tommaso Fusco; dateIdentified: 2022; **Event:** samplingProtocol: Pitfall traps; eventDate: 2014-06-05; **Record Level:** collectionID: Roma3_5.8
- **Type status:** Other material. **Occurrence:** recordedBy: Fattorini S., Di Giulio A.; individualCount: 1; sex: female; lifeStage: adult; occurrenceID: E46A01FB-65B6-5075-B89D-1A0CF7DA34FA; **Taxon:** scientificName: Euophrysfrontalis (Walckenaer, 1802); order: Araneae; family: Salticidae; genus: Euophrys; **Location:** country: Italy; countryCode: IT; stateProvince: Rome; county: Rome; municipality: Rome; locality: Appia Antica Regional Park, Rome; locationRemarks: Farnesiana; decimalLatitude: 41.839667; decimalLongitude: 12.525528; geodeticDatum: WGS84; **Identification:** identifiedBy: Tommaso Fusco; dateIdentified: 2022; **Event:** samplingProtocol: Pitfall traps; eventDate: 2014-06-19; **Record Level:** collectionID: Roma3_5.8
- **Type status:** Other material. **Occurrence:** recordedBy: Fattorini S., Di Giulio A.; individualCount: 2; sex: male; lifeStage: adult; occurrenceID: 074E6FDB-D36A-531F-947B-F1997987FF1E; **Taxon:** scientificName: Euophrysfrontalis (Walckenaer, 1802); order: Araneae; family: Salticidae; genus: Euophrys; **Location:** country: Italy; countryCode: IT; stateProvince: Rome; county: Rome; municipality: Rome; locality: Appia Antica Regional Park, Rome; locationRemarks: Tor Marancia; decimalLatitude: 41.850308; decimalLongitude: 12.503178; geodeticDatum: WGS84; **Identification:** identifiedBy: Tommaso Fusco; dateIdentified: 2022; **Event:** samplingProtocol: Pitfall traps; eventDate: 2014-06-17; **Record Level:** collectionID: Roma3_5.8

##### Distribution

Europe, North Africa, Turkey, Caucasus, Russia (Europe to Far East), Kazakhstan, Iran, Central Asia, China, Korea, Japan. Palaearctic (PAL) chorotype.

#### Euophrys rufibarbis

(Simon, 1868)

92344ABD-2408-5B6F-A1C4-C8209C957A8F

##### Materials

- **Type status:** Other material. **Occurrence:** recordedBy: Fattorini S., Di Giulio A.; individualCount: 1; sex: female; lifeStage: adult; occurrenceID: E715625A-CA6C-5B97-83E6-F1D2E45B16FD; **Taxon:** scientificName: Euophrysrufibarbis (Simon, 1868); order: Araneae; family: Salticidae; genus: Euophrys; **Location:** country: Italy; countryCode: IT; stateProvince: Rome; county: Rome; municipality: Rome; locality: Appia Antica Regional Park, Rome; locationRemarks: Acqua Santa; decimalLatitude: 41.850561; decimalLongitude: 12.530861; geodeticDatum: WGS84; **Identification:** identifiedBy: Tommaso Fusco; dateIdentified: 2022; **Event:** samplingProtocol: Pitfall traps; eventDate: 2014-05-28; **Record Level:** collectionID: Roma3_5.8
- **Type status:** Other material. **Occurrence:** recordedBy: Fattorini S., Di Giulio A.; individualCount: 1; sex: female; lifeStage: adult; occurrenceID: 51F3A394-57FB-5CE1-8EC2-F7358BED32FA; **Taxon:** scientificName: Euophrysrufibarbis (Simon, 1868); order: Araneae; family: Salticidae; genus: Euophrys; **Location:** country: Italy; countryCode: IT; stateProvince: Rome; county: Rome; municipality: Rome; locality: Appia Antica Regional Park, Rome; locationRemarks: Cava Fiorucci; decimalLatitude: 41.834106; decimalLongitude: 12.549264; geodeticDatum: WGS84; **Identification:** identifiedBy: Tommaso Fusco; dateIdentified: 2022; **Event:** samplingProtocol: Pitfall traps; eventDate: 2014-05-26; **Record Level:** collectionID: Roma3_5.8
- **Type status:** Other material. **Occurrence:** recordedBy: Fattorini S., Di Giulio A.; individualCount: 1; sex: female; lifeStage: adult; occurrenceID: 185CEE36-B8C8-5C9B-8F3C-D73305B7BAD0; **Taxon:** scientificName: Euophrysrufibarbis (Simon, 1868); order: Araneae; family: Salticidae; genus: Euophrys; **Location:** country: Italy; countryCode: IT; stateProvince: Rome; county: Rome; municipality: Rome; locality: Appia Antica Regional Park, Rome; locationRemarks: Tor Marancia; decimalLatitude: 41.850308; decimalLongitude: 12.503178; geodeticDatum: WGS84; **Identification:** identifiedBy: Tommaso Fusco; dateIdentified: 2022; **Event:** samplingProtocol: Pitfall traps; eventDate: 2014-06-17; **Record Level:** collectionID: Roma3_5.8
- **Type status:** Other material. **Occurrence:** recordedBy: Fattorini S., Di Giulio A.; individualCount: 1; sex: female; lifeStage: adult; occurrenceID: 99F76D8A-681F-5317-91B3-D9312F6B8CF2; **Taxon:** scientificName: Euophrysrufibarbis (Simon, 1868); order: Araneae; family: Salticidae; genus: Euophrys; **Location:** country: Italy; countryCode: IT; stateProvince: Rome; county: Rome; municipality: Rome; locality: Appia Antica Regional Park, Rome; locationRemarks: Torre Selce; decimalLatitude: 41.816611; decimalLongitude: 12.560667; geodeticDatum: WGS84; **Identification:** identifiedBy: Tommaso Fusco; dateIdentified: 2022; **Event:** samplingProtocol: Pitfall traps; eventDate: 2014-05-26; **Record Level:** collectionID: Roma3_5.8
- **Type status:** Other material. **Occurrence:** recordedBy: Fattorini S., Di Giulio A.; individualCount: 1; sex: female; lifeStage: adult; occurrenceID: 2FAE796B-651D-5B46-9FDA-46FC19B72D0E; **Taxon:** scientificName: Euophrysrufibarbis (Simon, 1868); order: Araneae; family: Salticidae; genus: Euophrys; **Location:** country: Italy; countryCode: IT; stateProvince: Rome; county: Rome; municipality: Rome; locality: Appia Antica Regional Park, Rome; locationRemarks: Torre Selce; decimalLatitude: 41.816611; decimalLongitude: 12.560667; geodeticDatum: WGS84; **Identification:** identifiedBy: Tommaso Fusco; dateIdentified: 2022; **Event:** samplingProtocol: Pitfall traps; eventDate: 2014-06-24; **Record Level:** collectionID: Roma3_5.8
- **Type status:** Other material. **Occurrence:** recordedBy: Fattorini S., Di Giulio A.; individualCount: 1; sex: male; lifeStage: adult; occurrenceID: 02CBE51E-EE64-579A-92AE-74524EB28098; **Taxon:** scientificName: Euophrysrufibarbis (Simon, 1868); order: Araneae; family: Salticidae; genus: Euophrys; **Location:** country: Italy; countryCode: IT; stateProvince: Rome; county: Rome; municipality: Rome; locality: Appia Antica Regional Park, Rome; locationRemarks: Torre Selce; decimalLatitude: 41.816611; decimalLongitude: 12.560667; geodeticDatum: WGS84; **Identification:** identifiedBy: Tommaso Fusco; dateIdentified: 2022; **Event:** samplingProtocol: Pitfall traps; eventDate: 2014-06-13; **Record Level:** collectionID: Roma3_5.8

##### Distribution

North Africa, southern Europe, Turkey, China. Centralasiatic-Europeo-Mediterranean (CEM) chorotype.

#### Euophrys sulfurea

(L. Koch, 1867)

34CCB4ED-8954-51DB-85A0-9916EA7B4D42

##### Materials

- **Type status:** Other material. **Occurrence:** recordedBy: Fattorini S., Di Giulio A.; individualCount: 1; sex: female; lifeStage: adult; occurrenceID: 4ED6C59A-7456-5EB8-B0AA-18D02B3AE507; **Taxon:** scientificName: Euophrys sulphurea (L. Koch, 1867); order: Araneae; family: Salticidae; genus: Euophrys; **Location:** country: Italy; countryCode: IT; stateProvince: Rome; county: Rome; municipality: Rome; locality: Appia Antica Regional Park, Rome; locationRemarks: Cava Fiorucci; decimalLatitude: 41.834106; decimalLongitude: 12.549264; geodeticDatum: WGS84; **Identification:** identifiedBy: Tommaso Fusco; dateIdentified: 2022; **Event:** samplingProtocol: Pitfall; eventDate: 2014-06-24; **Record Level:** collectionID: Roma3_5.8
- **Type status:** Other material. **Occurrence:** recordedBy: Fattorini S., Di Giulio A.; individualCount: 1; sex: male; lifeStage: adult; occurrenceID: 9EB1F95B-DE84-5A56-A157-E9BF87937409; **Taxon:** scientificName: Euophrys sulphurea (L. Koch, 1867); order: Araneae; family: Salticidae; genus: Euophrys; **Location:** country: Italy; countryCode: IT; stateProvince: Rome; county: Rome; municipality: Rome; locality: Appia Antica Regional Park, Rome; locationRemarks: Farnesiana; decimalLatitude: 41.839667; decimalLongitude: 12.525528; geodeticDatum: WGS84; **Identification:** identifiedBy: Tommaso Fusco; dateIdentified: 2022; **Event:** samplingProtocol: Pitfall; eventDate: 2014-06-09; **Record Level:** collectionID: Roma3_5.8
- **Type status:** Other material. **Occurrence:** recordedBy: Fattorini S., Di Giulio A.; individualCount: 1; sex: male; lifeStage: adult; occurrenceID: 0ADDA6E3-1894-5351-861A-3EDF7B8A4850; **Taxon:** scientificName: Euophrys sulphurea (L. Koch, 1867); order: Araneae; family: Salticidae; genus: Euophrys; **Location:** country: Italy; countryCode: IT; stateProvince: Rome; county: Rome; municipality: Rome; locality: Appia Antica Regional Park, Rome; locationRemarks: Caffarella Sud 3; decimalLatitude: 41.856928; decimalLongitude: 12.528406; geodeticDatum: WGS84; **Identification:** identifiedBy: Tommaso Fusco; dateIdentified: 2022; **Event:** samplingProtocol: Pitfall; eventDate: 2014-05-19; **Record Level:** collectionID: Roma3_5.8
- **Type status:** Other material. **Occurrence:** recordedBy: Fattorini S., Di Giulio A.; individualCount: 1; sex: male; lifeStage: adult; occurrenceID: 5B0B2CB8-D6A2-5582-A89D-84750846F933; **Taxon:** scientificName: Euophrys sulphurea (L. Koch, 1867); order: Araneae; family: Salticidae; genus: Euophrys; **Location:** country: Italy; countryCode: IT; stateProvince: Rome; county: Rome; municipality: Rome; locality: Appia Antica Regional Park, Rome; locationRemarks: Cava Fiorucci; decimalLatitude: 41.834106; decimalLongitude: 12.549264; geodeticDatum: WGS84; **Identification:** identifiedBy: Tommaso Fusco; dateIdentified: 2022; **Event:** samplingProtocol: Pitfall; eventDate: 2014-05-26; **Record Level:** collectionID: Roma3_5.8
- **Type status:** Other material. **Occurrence:** recordedBy: Fattorini S., Di Giulio A.; individualCount: 1; sex: male; lifeStage: adult; occurrenceID: 97DECA1C-B084-5498-BF7B-3EB0B1AE0278; **Taxon:** scientificName: Euophrys sulphurea (L. Koch, 1867); order: Araneae; family: Salticidae; genus: Euophrys; **Location:** country: Italy; countryCode: IT; stateProvince: Rome; county: Rome; municipality: Rome; locality: Appia Antica Regional Park, Rome; locationRemarks: Cava Fiorucci; decimalLatitude: 41.834106; decimalLongitude: 12.549264; geodeticDatum: WGS84; **Identification:** identifiedBy: Tommaso Fusco; dateIdentified: 2022; **Event:** samplingProtocol: Pitfall; eventDate: 2014-06-24; **Record Level:** collectionID: Roma3_5.8
- **Type status:** Other material. **Occurrence:** recordedBy: Fattorini S., Di Giulio A.; individualCount: 1; sex: male; lifeStage: adult; occurrenceID: C356DD29-E2B5-57D2-90C5-0659294959D4; **Taxon:** scientificName: Euophrys sulphurea (L. Koch, 1867); order: Araneae; family: Salticidae; genus: Euophrys; **Location:** country: Italy; countryCode: IT; stateProvince: Rome; county: Rome; municipality: Rome; locality: Appia Antica Regional Park, Rome; locationRemarks: Farnesiana; decimalLatitude: 41.839667; decimalLongitude: 12.525528; geodeticDatum: WGS84; **Identification:** identifiedBy: Tommaso Fusco; dateIdentified: 2022; **Event:** samplingProtocol: Pitfall; eventDate: 2014-05-28; **Record Level:** collectionID: Roma3_5.8

##### Distribution

Southern Europe, Turkey, Syria. Mediterranean (MED) chorotype.

#### Euophrys terrestris

(Simon, 1871)

67ABEA78-C888-532E-AA5B-09CB166EAEBE

##### Materials

- **Type status:** Other material. **Occurrence:** recordedBy: Fattorini S., Di Giulio A.; individualCount: 1; sex: male; lifeStage: adult; occurrenceID: 1F57C2ED-4C21-5D51-BC85-90CD43462580; **Taxon:** scientificName: Euophrysterrestris (Simon, 1871); order: Araneae; family: Salticidae; genus: Euophrys; **Location:** country: Italy; countryCode: IT; stateProvince: Rome; county: Rome; municipality: Rome; locality: Appia Antica Regional Park, Rome; locationRemarks: San Sebastiano; decimalLatitude: 41.855733; decimalLongitude: 12.515114; geodeticDatum: WGS84; **Identification:** identifiedBy: Tommaso Fusco; dateIdentified: 2022; **Event:** samplingProtocol: Pitfall traps; eventDate: 2014-05-26; **Record Level:** collectionID: Roma3_5.8
- **Type status:** Other material. **Occurrence:** recordedBy: Fattorini S., Di Giulio A.; individualCount: 2; sex: male; lifeStage: adult; occurrenceID: 0D0A74F7-EFA0-53FE-BDF1-23055565EFD6; **Taxon:** scientificName: Euophrysterrestris (Simon, 1871); order: Araneae; family: Salticidae; genus: Euophrys; **Location:** country: Italy; countryCode: IT; stateProvince: Rome; county: Rome; municipality: Rome; locality: Appia Antica Regional Park, Rome; locationRemarks: Acqua Santa; decimalLatitude: 41.850561; decimalLongitude: 12.530861; geodeticDatum: WGS84; **Identification:** identifiedBy: Tommaso Fusco; dateIdentified: 2022; **Event:** samplingProtocol: Pitfall traps; eventDate: 2013-10-31
- **Type status:** Other material. **Occurrence:** recordedBy: Fattorini S., Di Giulio A.; individualCount: 1; sex: male; lifeStage: adult; occurrenceID: 6CF1EA0E-8BD1-54A2-92F3-BA71A064FE30; **Taxon:** scientificName: Euophrysterrestris (Simon, 1871); order: Araneae; family: Salticidae; genus: Euophrys; **Location:** country: Italy; countryCode: IT; stateProvince: Rome; county: Rome; municipality: Rome; locality: Appia Antica Regional Park, Rome; locationRemarks: Appia Antica; decimalLatitude: 41.812575; decimalLongitude: 12.564011; geodeticDatum: WGS84; **Identification:** identifiedBy: Tommaso Fusco; dateIdentified: 2022; **Event:** samplingProtocol: Pitfall traps; eventDate: 2014-05-19

##### Distribution

SW Europe, North Africa. W-Mediterranean (WME) chorotype.

#### Evarcha jucunda

(Lucas, 1846)

7A43197B-C80F-5FA7-A024-3A62E54C4E05

##### Materials

- **Type status:** Other material. **Occurrence:** recordedBy: Fattorini S., Di Giulio A.; individualCount: 1; sex: male; lifeStage: adult; occurrenceID: 8C8D86E7-B44F-5F2F-BD85-10F82F6001E1; **Taxon:** scientificName: Evarchajucunda (Lucas, 1846); order: Araneae; family: Salticidae; genus: Evarcha; **Location:** country: Italy; countryCode: IT; stateProvince: Rome; county: Rome; municipality: Rome; locality: Appia Antica Regional Park, Rome; locationRemarks: Casal Verbeni; decimalLatitude: 41.815250; decimalLongitude: 12.552222; geodeticDatum: WGS84; **Identification:** identifiedBy: Tommaso Fusco; dateIdentified: 2022; **Event:** samplingProtocol: Pitfall traps; eventDate: 2014-06-13; **Record Level:** collectionID: Roma3_5.8
- **Type status:** Other material. **Occurrence:** recordedBy: Fattorini S., Di Giulio A.; individualCount: 2; sex: 1 male, 1 female; lifeStage: adult; occurrenceID: 472B0EB5-E766-5D46-B826-BC7564FB26F5; **Taxon:** scientificName: Evarchajucunda (Lucas, 1846); order: Araneae; family: Salticidae; genus: Evarcha; **Location:** country: Italy; countryCode: IT; stateProvince: Rome; county: Rome; municipality: Rome; locality: Appia Antica Regional Park, Rome; locationRemarks: Appia Antica; decimalLatitude: 41.812575; decimalLongitude: 12.564011; geodeticDatum: WGS84; **Identification:** identifiedBy: Tommaso Fusco; dateIdentified: 2022; **Event:** samplingProtocol: Pitfall traps; eventDate: 2014-06-05; **Record Level:** collectionID: Roma3_5.8
- **Type status:** Other material. **Occurrence:** recordedBy: Fattorini S., Di Giulio A.; individualCount: 1; sex: male; lifeStage: adult; occurrenceID: F720015B-BAFE-53BE-8FE8-9025F1C685B3; **Taxon:** scientificName: Evarchajucunda (Lucas, 1846); order: Araneae; family: Salticidae; genus: Evarcha; **Location:** country: Italy; countryCode: IT; stateProvince: Rome; county: Rome; municipality: Rome; locality: Appia Antica Regional Park, Rome; locationRemarks: Appia Antica; decimalLatitude: 41.812575; decimalLongitude: 12.564011; geodeticDatum: WGS84; **Identification:** identifiedBy: Tommaso Fusco; dateIdentified: 2022; **Event:** samplingProtocol: Pitfall traps; eventDate: 2014-06-13; **Record Level:** collectionID: Roma3_5.8
- **Type status:** Other material. **Occurrence:** recordedBy: Fattorini S., Di Giulio A.; individualCount: 1; sex: male; lifeStage: adult; occurrenceID: 63E099CA-EC56-5ADC-8EF5-DF54B0C91343; **Taxon:** scientificName: Evarchajucunda (Lucas, 1846); order: Araneae; family: Salticidae; genus: Evarcha; **Location:** country: Italy; countryCode: IT; stateProvince: Rome; county: Rome; municipality: Rome; locality: Appia Antica Regional Park, Rome; locationRemarks: Caffarella Nord; decimalLatitude: 41.867753; decimalLongitude: 12.512414; geodeticDatum: WGS84; **Identification:** identifiedBy: Tommaso Fusco; dateIdentified: 2022; **Event:** samplingProtocol: Pitfall traps; eventDate: 2014-05-27; **Record Level:** collectionID: Roma3_5.8

##### Distribution

Widespread in the Mediterranean area. Mediterranean (MED) chorotype.

#### Philaeus chrysops

(Poda, 1761)

F8F0374A-B55E-53D8-9C6A-E74FE9CF2010

##### Materials

- **Type status:** Other material. **Occurrence:** recordedBy: Fattorini S., Di Giulio A.; individualCount: 1; sex: female; lifeStage: adult; occurrenceID: 288993EE-D553-54C2-A131-247CCE27083D; **Taxon:** scientificName: Philaeuschrysops (Poda, 1761); order: Araneae; family: Salticidae; genus: Philaeus; **Location:** country: Italy; countryCode: IT; stateProvince: Rome; county: Rome; municipality: Rome; locality: Appia Antica Regional Park, Rome; locationRemarks: Cava Fiorucci; decimalLatitude: 41.834106; decimalLongitude: 12.549264; geodeticDatum: WGS84; **Identification:** identifiedBy: Tommaso Fusco; dateIdentified: 2022; **Event:** samplingProtocol: Pitfall traps; eventDate: 2014-05-26; **Record Level:** collectionID: Roma3_5.8

##### Distribution

Europe, North Africa to Middle East, Turkey, Caucasus, Russia (Europe to Far East), Iran, Kazakhstan, Central Asia, Afghanistan, China, Mongolia, Korea. Palaearctic (PAL) chorotype.

#### Phlegra bresnieri

(Lucas, 1846)

FB4AE9C6-5F27-5BC1-9394-95592889666B

##### Materials

- **Type status:** Other material. **Occurrence:** recordedBy: Fattorini S., Di Giulio A.; individualCount: 1; sex: male; lifeStage: adult; occurrenceID: BEEE156D-4A78-53F2-8BEE-B5E5A857666E; **Taxon:** scientificName: Phlegrabresnieri (Lucas, 1846); order: Araneae; family: Salticidae; genus: Phlegra; **Location:** country: Italy; countryCode: IT; stateProvince: Rome; county: Rome; municipality: Rome; locality: Appia Antica Regional Park, Rome; locationRemarks: Torre Selce; decimalLatitude: 41.816611; decimalLongitude: 12.560667; geodeticDatum: WGS84; **Identification:** identifiedBy: Tommaso Fusco; dateIdentified: 2022; **Event:** samplingProtocol: Pitfall traps; eventDate: 2014-05-26; **Record Level:** collectionID: Roma3_5.8

##### Distribution

Southern Europe, Turkey, Azerbaijan, Iran, Yemen, northern Africa, Ivory Coast, Tanzania, South Africa. Afrotropico-Mediterranean (AFM) chorotype.

#### Pseudeuophrys perdifumo

van Helsdingen, 2015

09BD2E5D-84E0-5787-8B40-F9F372D4637D

##### Materials

- **Type status:** Other material. **Occurrence:** recordedBy: Fattorini S., Di Giulio A.; individualCount: 1; sex: female; lifeStage: adult; occurrenceID: 6FC042B8-741F-5D0A-AAF5-BF1FF8F369B3; **Taxon:** scientificName: Pseudeuophrysperdifumo van Helsdingen, 2015; order: Araneae; family: Salticidae; genus: Pseudeuophrys; **Location:** country: Italy; countryCode: IT; stateProvince: Rome; county: Rome; municipality: Rome; locality: Appia Antica Regional Park, Rome; locationRemarks: San Sebastiano; decimalLatitude: 41.855733; decimalLongitude: 12.515114; geodeticDatum: WGS84; **Identification:** identifiedBy: Tommaso Fusco; dateIdentified: 2022; **Event:** samplingProtocol: Pitfall traps; eventDate: 2014-06-09; **Record Level:** collectionID: Roma3_5.8

##### Distribution

Endemic (END) species only found in southern Italy (Van Helsdingen 2015)

##### Notes

A single female of this species was found. The epigyne resembles that of *P.perdifumo* illustrated and described by Van Helsdingen (2015). A male specimen is needed to confirm this species.

#### Pseudeuophrys vafra

(Blackwall, 1867)

CF635FFB-DBF8-531D-97B6-B6AB36D0123B

##### Materials

- **Type status:** Other material. **Occurrence:** recordedBy: Fattorini S., Di Giulio A.; individualCount: 1; sex: female; lifeStage: adult; occurrenceID: 08AA9092-5ACA-5BC4-BEE6-AC070A5E33EB; **Taxon:** scientificName: Pseudeuophrysvafra (Blackwall, 1867); order: Araneae; family: Salticidae; genus: Pseudeuophrys; **Location:** country: Italy; countryCode: IT; stateProvince: Rome; county: Rome; municipality: Rome; locality: Appia Antica Regional Park, Rome; locationRemarks: Caffarella Centro; decimalLatitude: 41.864889; decimalLongitude: 12.516389; geodeticDatum: WGS84; **Identification:** identifiedBy: Tommaso Fusco; dateIdentified: 2022; **Event:** samplingProtocol: Pitfall traps; eventDate: 2014-06-06; **Record Level:** collectionID: Roma3_5.8

##### Distribution

Azores, Madeira, North Africa, Europe (Portugal to Russia), Georgia. Europeo-Mediterranean (EUM) chorotype.

#### Scytodidae

Blackwall, 1864

59701501-96AC-5DE5-A9AE-989F0FA5ABAA

#### Scytodes thoracica

(Latreille, 1802)

AA5345FC-692D-53B2-9DF3-BA79003614B1

##### Materials

- **Type status:** Other material. **Occurrence:** recordedBy: Fattorini S., Di Giulio A.; individualCount: 1; sex: male; lifeStage: adult; occurrenceID: DAEA5AB8-379E-5729-929E-9204572FC7F3; **Taxon:** scientificName: Scytodesthoracica (Latreille, 1802); order: Araneae; family: Scytodidae; genus: Scytodes; **Location:** country: Italy; countryCode: IT; stateProvince: Rome; county: Rome; municipality: Rome; locality: Appia Antica Regional Park, Rome; locationRemarks: Acqua Santa; decimalLatitude: 41.850561; decimalLongitude: 12.530861; geodeticDatum: WGS84; **Identification:** identifiedBy: Tommaso Fusco; dateIdentified: 2022; **Event:** samplingProtocol: Pitfall traps; eventDate: 2014-06-19; **Record Level:** collectionID: Roma3_5.8
- **Type status:** Other material. **Occurrence:** recordedBy: Fattorini S., Di Giulio A.; individualCount: 1; sex: female; lifeStage: adult; occurrenceID: 34D83E76-356F-5A51-BDBB-E31C6A324E8D; **Taxon:** scientificName: Scytodesthoracica (Latreille, 1802); order: Araneae; family: Scytodidae; genus: Scytodes; **Location:** country: Italy; countryCode: IT; stateProvince: Rome; county: Rome; municipality: Rome; locality: Appia Antica Regional Park, Rome; locationRemarks: Acqua Santa; decimalLatitude: 41.850561; decimalLongitude: 12.530861; geodeticDatum: WGS84; **Identification:** identifiedBy: Tommaso Fusco; dateIdentified: 2022; **Event:** samplingProtocol: Pitfall traps; eventDate: 2014-05-20; **Record Level:** collectionID: Roma3_5.8
- **Type status:** Other material. **Occurrence:** recordedBy: Fattorini S., Di Giulio A.; individualCount: 2; sex: female; lifeStage: adult; occurrenceID: 2486CB37-EE24-5F7C-B064-725F15F9D219; **Taxon:** scientificName: Scytodesthoracica (Latreille, 1802); order: Araneae; family: Scytodidae; genus: Scytodes; **Location:** country: Italy; countryCode: IT; stateProvince: Rome; county: Rome; municipality: Rome; locality: Appia Antica Regional Park, Rome; locationRemarks: Acqua Santa; decimalLatitude: 41.850561; decimalLongitude: 12.530861; geodeticDatum: WGS84; **Identification:** identifiedBy: Tommaso Fusco; dateIdentified: 2022; **Event:** samplingProtocol: Pitfall traps; eventDate: 2014-05-28; **Record Level:** collectionID: Roma3_5.8
- **Type status:** Other material. **Occurrence:** recordedBy: Fattorini S., Di Giulio A.; individualCount: 2; sex: 1 male, 1 female; lifeStage: adult; occurrenceID: 622C8654-92E0-5FA5-A94E-71D928C3EAB9; **Taxon:** scientificName: Scytodesthoracica (Latreille, 1802); order: Araneae; family: Scytodidae; genus: Scytodes; **Location:** country: Italy; countryCode: IT; stateProvince: Rome; county: Rome; municipality: Rome; locality: Appia Antica Regional Park, Rome; locationRemarks: Acqua Santa; decimalLatitude: 41.850561; decimalLongitude: 12.530861; geodeticDatum: WGS84; **Identification:** identifiedBy: Tommaso Fusco; dateIdentified: 2022; **Event:** samplingProtocol: Pitfall traps; eventDate: 2014-06-09; **Record Level:** collectionID: Roma3_5.8
- **Type status:** Other material. **Occurrence:** recordedBy: Fattorini S., Di Giulio A.; individualCount: 3; sex: 1 male, 2 female; lifeStage: adult; occurrenceID: A9002645-8138-5614-A188-0CDA38F5348A; **Taxon:** scientificName: Scytodesthoracica (Latreille, 1802); order: Araneae; family: Scytodidae; genus: Scytodes; **Location:** country: Italy; countryCode: IT; stateProvince: Rome; county: Rome; municipality: Rome; locality: Appia Antica Regional Park, Rome; locationRemarks: Appia Antica; decimalLatitude: 41.812575; decimalLongitude: 12.564011; geodeticDatum: WGS84; **Identification:** identifiedBy: Tommaso Fusco; dateIdentified: 2022; **Event:** samplingProtocol: Pitfall traps; eventDate: 2014-06-05; **Record Level:** collectionID: Roma3_5.8
- **Type status:** Other material. **Occurrence:** recordedBy: Fattorini S., Di Giulio A.; individualCount: 2; sex: m; lifeStage: adult; occurrenceID: 276B6CE9-538E-5087-82CD-EA8C3F3A21D5; **Taxon:** scientificName: Scytodesthoracica (Latreille, 1802); order: Araneae; family: Scytodidae; genus: Scytodes; **Location:** country: Italy; countryCode: IT; stateProvince: Rome; county: Rome; municipality: Rome; locality: Appia Antica Regional Park, Rome; locationRemarks: Appia Antica; decimalLatitude: 41.812575; decimalLongitude: 12.564011; geodeticDatum: WGS84; **Identification:** identifiedBy: Tommaso Fusco; dateIdentified: 2022; **Event:** samplingProtocol: Pitfall traps; eventDate: 2014-06-05; **Record Level:** collectionID: Roma3_5.8
- **Type status:** Other material. **Occurrence:** recordedBy: Fattorini S., Di Giulio A.; individualCount: 6; sex: 3 male, 3 female; lifeStage: adult; occurrenceID: A532462C-3229-5423-A40D-696E70DA1277; **Taxon:** scientificName: Scytodesthoracica (Latreille, 1802); order: Araneae; family: Scytodidae; genus: Scytodes; **Location:** country: Italy; countryCode: IT; stateProvince: Rome; county: Rome; municipality: Rome; locality: Appia Antica Regional Park, Rome; locationRemarks: Appia Antica; decimalLatitude: 41.812575; decimalLongitude: 12.564011; geodeticDatum: WGS84; **Identification:** identifiedBy: Tommaso Fusco; dateIdentified: 2022; **Event:** samplingProtocol: Pitfall traps; eventDate: 2014-06-13; **Record Level:** collectionID: Roma3_5.8
- **Type status:** Other material. **Occurrence:** recordedBy: Fattorini S., Di Giulio A.; individualCount: 8; sex: 5 male, 3 female; lifeStage: adult; occurrenceID: 988408A9-507A-556A-8E36-44B55C1A992D; **Taxon:** scientificName: Scytodesthoracica (Latreille, 1802); order: Araneae; family: Scytodidae; genus: Scytodes; **Location:** country: Italy; countryCode: IT; stateProvince: Rome; county: Rome; municipality: Rome; locality: Appia Antica Regional Park, Rome; locationRemarks: Appia Antica; decimalLatitude: 41.812575; decimalLongitude: 12.564011; geodeticDatum: WGS84; **Identification:** identifiedBy: Tommaso Fusco; dateIdentified: 2022; **Event:** samplingProtocol: Pitfall traps; eventDate: 2014-06-24; **Record Level:** collectionID: Roma3_5.8
- **Type status:** Other material. **Occurrence:** recordedBy: Fattorini S., Di Giulio A.; individualCount: 1; sex: female; lifeStage: adult; occurrenceID: 91BFA75C-E8CE-5F9E-B456-F2A52C836C2A; **Taxon:** scientificName: Scytodesthoracica (Latreille, 1802); order: Araneae; family: Scytodidae; genus: Scytodes; **Location:** country: Italy; countryCode: IT; stateProvince: Rome; county: Rome; municipality: Rome; locality: Appia Antica Regional Park, Rome; locationRemarks: Caffarella Centro; decimalLatitude: 41.864889; decimalLongitude: 12.516389; geodeticDatum: WGS84; **Identification:** identifiedBy: Tommaso Fusco; dateIdentified: 2022; **Event:** samplingProtocol: Pitfall traps; eventDate: 2014-05-27; **Record Level:** collectionID: Roma3_5.8
- **Type status:** Other material. **Occurrence:** recordedBy: Fattorini S., Di Giulio A.; individualCount: 2; sex: 1 male, 1 female; lifeStage: adult; occurrenceID: 935B30DF-806E-51FA-8EB7-BCD93EB35150; **Taxon:** scientificName: Scytodesthoracica (Latreille, 1802); order: Araneae; family: Scytodidae; genus: Scytodes; **Location:** country: Italy; countryCode: IT; stateProvince: Rome; county: Rome; municipality: Rome; locality: Appia Antica Regional Park, Rome; locationRemarks: Caffarella Centro; decimalLatitude: 41.864889; decimalLongitude: 12.516389; geodeticDatum: WGS84; **Identification:** identifiedBy: Tommaso Fusco; dateIdentified: 2022; **Event:** samplingProtocol: Pitfall traps; eventDate: 2014-06-18; **Record Level:** collectionID: Roma3_5.8
- **Type status:** Other material. **Occurrence:** recordedBy: Fattorini S., Di Giulio A.; individualCount: 1; sex: female; lifeStage: adult; occurrenceID: 81E184A4-4683-5C72-A31E-46ACAC822AA0; **Taxon:** scientificName: Scytodesthoracica (Latreille, 1802); order: Araneae; family: Scytodidae; genus: Scytodes; **Location:** country: Italy; countryCode: IT; stateProvince: Rome; county: Rome; municipality: Rome; locality: Appia Antica Regional Park, Rome; locationRemarks: Caffarella Centro; decimalLatitude: 41.864889; decimalLongitude: 12.516389; geodeticDatum: WGS84; **Identification:** identifiedBy: Tommaso Fusco; dateIdentified: 2022; **Event:** samplingProtocol: Pitfall traps; eventDate: 2014-06-06; **Record Level:** collectionID: Roma3_5.8
- **Type status:** Other material. **Occurrence:** recordedBy: Fattorini S., Di Giulio A.; individualCount: 4; sex: 2 male, 2 female; lifeStage: adult; occurrenceID: 60A4685E-B044-5532-9308-00E4DDCC62F6; **Taxon:** scientificName: Scytodesthoracica (Latreille, 1802); order: Araneae; family: Scytodidae; genus: Scytodes; **Location:** country: Italy; countryCode: IT; stateProvince: Rome; county: Rome; municipality: Rome; locality: Appia Antica Regional Park, Rome; locationRemarks: Caffarella Centro; decimalLatitude: 41.864889; decimalLongitude: 12.516389; geodeticDatum: WGS84; **Identification:** identifiedBy: Tommaso Fusco; dateIdentified: 2022; **Event:** samplingProtocol: Pitfall traps; eventDate: 2014-06-18; **Record Level:** collectionID: Roma3_5.8
- **Type status:** Other material. **Occurrence:** recordedBy: Fattorini S., Di Giulio A.; individualCount: 1; sex: female; lifeStage: adult; occurrenceID: EA1F9E72-222E-5CFB-B0A3-FC87755411EF; **Taxon:** scientificName: Scytodesthoracica (Latreille, 1802); order: Araneae; family: Scytodidae; genus: Scytodes; **Location:** country: Italy; countryCode: IT; stateProvince: Rome; county: Rome; municipality: Rome; locality: Appia Antica Regional Park, Rome; locationRemarks: Caffarella Nord; decimalLatitude: 41.867753; decimalLongitude: 12.512414; geodeticDatum: WGS84; **Identification:** identifiedBy: Tommaso Fusco; dateIdentified: 2022; **Event:** samplingProtocol: Pitfall traps; eventDate: 2014-06-06; **Record Level:** collectionID: Roma3_5.8
- **Type status:** Other material. **Occurrence:** recordedBy: Fattorini S., Di Giulio A.; individualCount: 1; sex: male; lifeStage: adult; occurrenceID: 276F22E8-D937-5083-A412-E0E75BBC8459; **Taxon:** scientificName: Scytodesthoracica (Latreille, 1802); order: Araneae; family: Scytodidae; genus: Scytodes; **Location:** country: Italy; countryCode: IT; stateProvince: Rome; county: Rome; municipality: Rome; locality: Appia Antica Regional Park, Rome; locationRemarks: Caffarella Nord; decimalLatitude: 41.867753; decimalLongitude: 12.512414; geodeticDatum: WGS84; **Identification:** identifiedBy: Tommaso Fusco; dateIdentified: 2022; **Event:** samplingProtocol: Pitfall traps; eventDate: 2014-06-18; **Record Level:** collectionID: Roma3_5.8
- **Type status:** Other material. **Occurrence:** recordedBy: Fattorini S., Di Giulio A.; individualCount: 5; sex: 1 male, 4 female; lifeStage: adult; occurrenceID: 822217D3-EEFF-5FE5-9E88-E3ED7BD26131; **Taxon:** scientificName: Scytodesthoracica (Latreille, 1802); order: Araneae; family: Scytodidae; genus: Scytodes; **Location:** country: Italy; countryCode: IT; stateProvince: Rome; county: Rome; municipality: Rome; locality: Appia Antica Regional Park, Rome; locationRemarks: Casal Verbeni; decimalLatitude: 41.815250; decimalLongitude: 12.552222; geodeticDatum: WGS84; **Identification:** identifiedBy: Tommaso Fusco; dateIdentified: 2022; **Event:** samplingProtocol: Pitfall traps; eventDate: 2014-05-26; **Record Level:** collectionID: Roma3_5.8
- **Type status:** Other material. **Occurrence:** recordedBy: Fattorini S., Di Giulio A.; individualCount: 1; sex: female; lifeStage: adult; occurrenceID: DD14F20E-0062-5584-8393-97F282E49824; **Taxon:** scientificName: Scytodesthoracica (Latreille, 1802); order: Araneae; family: Scytodidae; genus: Scytodes; **Location:** country: Italy; countryCode: IT; stateProvince: Rome; county: Rome; municipality: Rome; locality: Appia Antica Regional Park, Rome; locationRemarks: Casal Verbeni; decimalLatitude: 41.815250; decimalLongitude: 12.552222; geodeticDatum: WGS84; **Identification:** identifiedBy: Tommaso Fusco; dateIdentified: 2022; **Event:** samplingProtocol: Pitfall traps; eventDate: 2014-06-24; **Record Level:** collectionID: Roma3_5.8
- **Type status:** Other material. **Occurrence:** recordedBy: Fattorini S., Di Giulio A.; individualCount: 3; sex: 1 male, 2 female; lifeStage: adult; occurrenceID: B117F4F5-4481-5206-A89E-D25B94078EA3; **Taxon:** scientificName: Scytodesthoracica (Latreille, 1802); order: Araneae; family: Scytodidae; genus: Scytodes; **Location:** country: Italy; countryCode: IT; stateProvince: Rome; county: Rome; municipality: Rome; locality: Appia Antica Regional Park, Rome; locationRemarks: Casal Verbeni; decimalLatitude: 41.815250; decimalLongitude: 12.552222; geodeticDatum: WGS84; **Identification:** identifiedBy: Tommaso Fusco; dateIdentified: 2022; **Event:** samplingProtocol: Pitfall traps; eventDate: 2014-06-13; **Record Level:** collectionID: Roma3_5.8
- **Type status:** Other material. **Occurrence:** recordedBy: Fattorini S., Di Giulio A.; individualCount: 1; sex: female; lifeStage: adult; occurrenceID: 0B2B2EAD-801B-5FF2-A544-D0E4A9C0925B; **Taxon:** scientificName: Scytodesthoracica (Latreille, 1802); order: Araneae; family: Scytodidae; genus: Scytodes; **Location:** country: Italy; countryCode: IT; stateProvince: Rome; county: Rome; municipality: Rome; locality: Appia Antica Regional Park, Rome; locationRemarks: Cava Fiorucci; decimalLatitude: 41.834106; decimalLongitude: 12.549264; geodeticDatum: WGS84; **Identification:** identifiedBy: Tommaso Fusco; dateIdentified: 2022; **Event:** samplingProtocol: Pitfall traps; eventDate: 2014-06-05; **Record Level:** collectionID: Roma3_5.8
- **Type status:** Other material. **Occurrence:** recordedBy: Fattorini S., Di Giulio A.; individualCount: 2; sex: 2 male, 1 female; lifeStage: adult; occurrenceID: 14F7A79B-32B1-5D0C-8ED5-C7B413A3E0A4; **Taxon:** scientificName: Scytodesthoracica (Latreille, 1802); order: Araneae; family: Scytodidae; genus: Scytodes; **Location:** country: Italy; countryCode: IT; stateProvince: Rome; county: Rome; municipality: Rome; locality: Appia Antica Regional Park, Rome; locationRemarks: Cava Fiorucci; decimalLatitude: 41.834106; decimalLongitude: 12.549264; geodeticDatum: WGS84; **Identification:** identifiedBy: Tommaso Fusco; dateIdentified: 2022; **Event:** samplingProtocol: Pitfall traps; eventDate: 2014-06-13; **Record Level:** collectionID: Roma3_5.8
- **Type status:** Other material. **Occurrence:** recordedBy: Fattorini S., Di Giulio A.; individualCount: 1; sex: female; lifeStage: adult; occurrenceID: 72F382CF-0CF8-55BE-90AC-F16952925AB3; **Taxon:** scientificName: Scytodesthoracica (Latreille, 1802); order: Araneae; family: Scytodidae; genus: Scytodes; **Location:** country: Italy; countryCode: IT; stateProvince: Rome; county: Rome; municipality: Rome; locality: Appia Antica Regional Park, Rome; locationRemarks: Cava Fiorucci; decimalLatitude: 41.834106; decimalLongitude: 12.549264; geodeticDatum: WGS84; **Identification:** identifiedBy: Tommaso Fusco; dateIdentified: 2022; **Event:** samplingProtocol: Pitfall traps; eventDate: 2014-06-24; **Record Level:** collectionID: Roma3_5.8
- **Type status:** Other material. **Occurrence:** recordedBy: Fattorini S., Di Giulio A.; individualCount: 1; sex: female; lifeStage: adult; occurrenceID: F458B420-D20E-5571-ADD1-2C7FA1482B22; **Taxon:** scientificName: Scytodesthoracica (Latreille, 1802); order: Araneae; family: Scytodidae; genus: Scytodes; **Location:** country: Italy; countryCode: IT; stateProvince: Rome; county: Rome; municipality: Rome; locality: Appia Antica Regional Park, Rome; locationRemarks: Cava Fiorucci; decimalLatitude: 41.834106; decimalLongitude: 12.549264; geodeticDatum: WGS84; **Identification:** identifiedBy: Tommaso Fusco; dateIdentified: 2022; **Event:** samplingProtocol: Pitfall traps; eventDate: 2013-12-06; **Record Level:** collectionID: Roma3_5.8
- **Type status:** Other material. **Occurrence:** recordedBy: Fattorini S., Di Giulio A.; individualCount: 2; sex: female; lifeStage: adult; occurrenceID: E7D20840-FA63-58F9-BE83-247A769DA6AC; **Taxon:** scientificName: Scytodesthoracica (Latreille, 1802); order: Araneae; family: Scytodidae; genus: Scytodes; **Location:** country: Italy; countryCode: IT; stateProvince: Rome; county: Rome; municipality: Rome; locality: Appia Antica Regional Park, Rome; locationRemarks: Farnesiana; decimalLatitude: 41.839667; decimalLongitude: 12.525528; geodeticDatum: WGS84; **Identification:** identifiedBy: Tommaso Fusco; dateIdentified: 2022; **Event:** samplingProtocol: Pitfall traps; eventDate: 2014-06-19; **Record Level:** collectionID: Roma3_5.8
- **Type status:** Other material. **Occurrence:** recordedBy: Fattorini S., Di Giulio A.; individualCount: 2; sex: female; lifeStage: adult; occurrenceID: F49A3837-B789-525C-A9B6-C58E16303A02; **Taxon:** scientificName: Scytodesthoracica (Latreille, 1802); order: Araneae; family: Scytodidae; genus: Scytodes; **Location:** country: Italy; countryCode: IT; stateProvince: Rome; county: Rome; municipality: Rome; locality: Appia Antica Regional Park, Rome; locationRemarks: Farnesiana; decimalLatitude: 41.839667; decimalLongitude: 12.525528; geodeticDatum: WGS84; **Identification:** identifiedBy: Tommaso Fusco; dateIdentified: 2022; **Event:** samplingProtocol: Pitfall traps; eventDate: 2014-05-28; **Record Level:** collectionID: Roma3_5.8
- **Type status:** Other material. **Occurrence:** recordedBy: Fattorini S., Di Giulio A.; individualCount: 1; sex: male; lifeStage: adult; occurrenceID: B5A8E48A-E579-5305-B8D3-D50F661CF858; **Taxon:** scientificName: Scytodesthoracica (Latreille, 1802); order: Araneae; family: Scytodidae; genus: Scytodes; **Location:** country: Italy; countryCode: IT; stateProvince: Rome; county: Rome; municipality: Rome; locality: Appia Antica Regional Park, Rome; locationRemarks: Farnesiana; decimalLatitude: 41.839667; decimalLongitude: 12.525528; geodeticDatum: WGS84; **Identification:** identifiedBy: Tommaso Fusco; dateIdentified: 2022; **Event:** samplingProtocol: Pitfall traps; eventDate: 2014-06-09; **Record Level:** collectionID: Roma3_5.8
- **Type status:** Other material. **Occurrence:** recordedBy: Fattorini S., Di Giulio A.; individualCount: 1; sex: female; lifeStage: adult; occurrenceID: 00E79236-2A57-51D1-A69F-B2D31350DDDA; **Taxon:** scientificName: Scytodesthoracica (Latreille, 1802); order: Araneae; family: Scytodidae; genus: Scytodes; **Location:** country: Italy; countryCode: IT; stateProvince: Rome; county: Rome; municipality: Rome; locality: Appia Antica Regional Park, Rome; locationRemarks: Farnesiana; decimalLatitude: 41.839667; decimalLongitude: 12.525528; geodeticDatum: WGS84; **Identification:** identifiedBy: Tommaso Fusco; dateIdentified: 2022; **Event:** samplingProtocol: Pitfall traps; eventDate: 2013-11-04; **Record Level:** collectionID: Roma3_5.8
- **Type status:** Other material. **Occurrence:** recordedBy: Fattorini S., Di Giulio A.; individualCount: 2; sex: female; lifeStage: adult; occurrenceID: C67BED60-2C75-5D38-BAA8-9F69D31E4E15; **Taxon:** scientificName: Scytodesthoracica (Latreille, 1802); order: Araneae; family: Scytodidae; genus: Scytodes; **Location:** country: Italy; countryCode: IT; stateProvince: Rome; county: Rome; municipality: Rome; locality: Appia Antica Regional Park, Rome; locationRemarks: San Sebastiano; decimalLatitude: 41.855733; decimalLongitude: 12.515114; geodeticDatum: WGS84; **Identification:** identifiedBy: Tommaso Fusco; dateIdentified: 2022; **Event:** samplingProtocol: Pitfall traps; eventDate: 2014-05-28; **Record Level:** collectionID: Roma3_5.8
- **Type status:** Other material. **Occurrence:** recordedBy: Fattorini S., Di Giulio A.; individualCount: 3; sex: female; lifeStage: adult; occurrenceID: FB40B88E-532D-5A6B-9B1F-CEF7F51C01F2; **Taxon:** scientificName: Scytodesthoracica (Latreille, 1802); order: Araneae; family: Scytodidae; genus: Scytodes; **Location:** country: Italy; countryCode: IT; stateProvince: Rome; county: Rome; municipality: Rome; locality: Appia Antica Regional Park, Rome; locationRemarks: San Sebastiano; decimalLatitude: 41.855733; decimalLongitude: 12.515114; geodeticDatum: WGS84; **Identification:** identifiedBy: Tommaso Fusco; dateIdentified: 2022; **Event:** samplingProtocol: Pitfall traps; eventDate: 2014-06-09; **Record Level:** collectionID: Roma3_5.8
- **Type status:** Other material. **Occurrence:** recordedBy: Fattorini S., Di Giulio A.; individualCount: 2; sex: 1 male, 1 female; lifeStage: adult; occurrenceID: 0FFD7C32-8F3A-5C7D-8DEB-A97AB0BD3679; **Taxon:** scientificName: Scytodesthoracica (Latreille, 1802); order: Araneae; family: Scytodidae; genus: Scytodes; **Location:** country: Italy; countryCode: IT; stateProvince: Rome; county: Rome; municipality: Rome; locality: Appia Antica Regional Park, Rome; locationRemarks: San Sebastiano; decimalLatitude: 41.855733; decimalLongitude: 12.515114; geodeticDatum: WGS84; **Identification:** identifiedBy: Tommaso Fusco; dateIdentified: 2022; **Event:** samplingProtocol: Pitfall traps; eventDate: 2014-06-19; **Record Level:** collectionID: Roma3_5.8
- **Type status:** Other material. **Occurrence:** recordedBy: Fattorini S., Di Giulio A.; individualCount: 4; sex: female; lifeStage: adult; occurrenceID: 25E4D451-8EF8-5445-8B3B-AAE1F1AE23F9; **Taxon:** scientificName: Scytodesthoracica (Latreille, 1802); order: Araneae; family: Scytodidae; genus: Scytodes; **Location:** country: Italy; countryCode: IT; stateProvince: Rome; county: Rome; municipality: Rome; locality: Appia Antica Regional Park, Rome; locationRemarks: Tor Marancia; decimalLatitude: 41.850308; decimalLongitude: 12.503178; geodeticDatum: WGS84; **Identification:** identifiedBy: Tommaso Fusco; dateIdentified: 2022; **Event:** samplingProtocol: Pitfall traps; eventDate: 2014-05-27; **Record Level:** collectionID: Roma3_5.8
- **Type status:** Other material. **Occurrence:** recordedBy: Fattorini S., Di Giulio A.; individualCount: 4; sex: 3 male, 1 female; lifeStage: adult; occurrenceID: 58EB3E76-2FAA-5414-8301-F73A486ED126; **Taxon:** scientificName: Scytodesthoracica (Latreille, 1802); order: Araneae; family: Scytodidae; genus: Scytodes; **Location:** country: Italy; countryCode: IT; stateProvince: Rome; county: Rome; municipality: Rome; locality: Appia Antica Regional Park, Rome; locationRemarks: Tor Marancia; decimalLatitude: 41.850308; decimalLongitude: 12.503178; geodeticDatum: WGS84; **Identification:** identifiedBy: Tommaso Fusco; dateIdentified: 2022; **Event:** samplingProtocol: Pitfall traps; eventDate: 2014-06-17; **Record Level:** collectionID: Roma3_5.8
- **Type status:** Other material. **Occurrence:** recordedBy: Fattorini S., Di Giulio A.; individualCount: 1; sex: 1 male, 1 female; lifeStage: adult; occurrenceID: 686DCF15-831A-5CE2-84C6-EDF1F2798748; **Taxon:** scientificName: Scytodesthoracica (Latreille, 1802); order: Araneae; family: Scytodidae; genus: Scytodes; **Location:** country: Italy; countryCode: IT; stateProvince: Rome; county: Rome; municipality: Rome; locality: Appia Antica Regional Park, Rome; locationRemarks: Torre Selce; decimalLatitude: 41.816611; decimalLongitude: 12.560667; geodeticDatum: WGS84; **Identification:** identifiedBy: Tommaso Fusco; dateIdentified: 2022; **Event:** samplingProtocol: Pitfall traps; eventDate: 2014-05-26; **Record Level:** collectionID: Roma3_5.8
- **Type status:** Other material. **Occurrence:** recordedBy: Fattorini S., Di Giulio A.; individualCount: 6; sex: 5 male, 1 female; lifeStage: adult; occurrenceID: 5333C031-F333-51C9-9BE2-1A6FCECE70F8; **Taxon:** scientificName: Scytodesthoracica (Latreille, 1802); order: Araneae; family: Scytodidae; genus: Scytodes; **Location:** country: Italy; countryCode: IT; stateProvince: Rome; county: Rome; municipality: Rome; locality: Appia Antica Regional Park, Rome; locationRemarks: Torre Selce; decimalLatitude: 41.816611; decimalLongitude: 12.560667; geodeticDatum: WGS84; **Identification:** identifiedBy: Tommaso Fusco; dateIdentified: 2022; **Event:** samplingProtocol: Pitfall traps; eventDate: 2014-06-24; **Record Level:** collectionID: Roma3_5.8
- **Type status:** Other material. **Occurrence:** recordedBy: Fattorini S., Di Giulio A.; individualCount: 3; sex: 2 male, 1 female; lifeStage: adult; occurrenceID: D63E97AB-E9F6-5CD8-A5C0-081DEA555F90; **Taxon:** scientificName: Scytodesthoracica (Latreille, 1802); order: Araneae; family: Scytodidae; genus: Scytodes; **Location:** country: Italy; countryCode: IT; stateProvince: Rome; county: Rome; municipality: Rome; locality: Appia Antica Regional Park, Rome; locationRemarks: Torre Selce; decimalLatitude: 41.816611; decimalLongitude: 12.560667; geodeticDatum: WGS84; **Identification:** identifiedBy: Tommaso Fusco; dateIdentified: 2022; **Event:** samplingProtocol: Pitfall traps; eventDate: 2014-06-05; **Record Level:** collectionID: Roma3_5.8
- **Type status:** Other material. **Occurrence:** recordedBy: Fattorini S., Di Giulio A.; individualCount: 3; sex: female; lifeStage: adult; occurrenceID: 97F01AC1-6339-559B-99B0-76D682299A28; **Taxon:** scientificName: Scytodesthoracica (Latreille, 1802); order: Araneae; family: Scytodidae; genus: Scytodes; **Location:** country: Italy; countryCode: IT; stateProvince: Rome; county: Rome; municipality: Rome; locality: Appia Antica Regional Park, Rome; locationRemarks: Torre Selce; decimalLatitude: 41.816611; decimalLongitude: 12.560667; geodeticDatum: WGS84; **Identification:** identifiedBy: Tommaso Fusco; dateIdentified: 2022; **Event:** samplingProtocol: Pitfall traps; eventDate: 2014-06-13; **Record Level:** collectionID: Roma3_5.8
- **Type status:** Other material. **Occurrence:** recordedBy: Fattorini S., Di Giulio A.; individualCount: 1; sex: female; lifeStage: adult; occurrenceID: B53E7D0F-BC8D-5281-8D4A-10426B078EA3; **Taxon:** scientificName: Scytodesthoracica (Latreille, 1802); order: Araneae; family: Scytodidae; genus: Scytodes; **Location:** country: Italy; countryCode: IT; stateProvince: Rome; county: Rome; municipality: Rome; locality: Appia Antica Regional Park, Rome; locationRemarks: Torre Selce; decimalLatitude: 41.816611; decimalLongitude: 12.560667; geodeticDatum: WGS84; **Identification:** identifiedBy: Tommaso Fusco; dateIdentified: 2022; **Event:** samplingProtocol: Pitfall traps; eventDate: 2013-11-07; **Record Level:** collectionID: Roma3_5.8

##### Distribution

Europe, North Africa, Turkey, Iran, temperate Asia to China, Korea, Japan. Introduced to North America, Argentina, South Africa, India, Australia, New Zealand. Cosmopolitan (COS) chorotype.

#### Sparassidae

Bertkau, 1872

FF74DDF5-2D51-5F06-8822-B56A4B617619

#### Olios argelasius

(Walckenaer, 1806)

C496E0DC-C4C6-5CA8-9AF1-402E83CAFC77

##### Materials

- **Type status:** Other material. **Occurrence:** recordedBy: Fattorini S., Di Giulio A.; individualCount: 1; sex: female; lifeStage: adult; occurrenceID: AF0C3A89-3F90-5E5E-B7F3-7AA8755AC227; **Taxon:** scientificName: Oliosargelasius (Walckenaer, 1806); order: Araneae; family: Sparassidae; genus: Olios; **Location:** country: Italy; countryCode: IT; stateProvince: Rome; county: Rome; municipality: Rome; locality: Appia Antica Regional Park, Rome; locationRemarks: Caffarella Centro; decimalLatitude: 41.864889; decimalLongitude: 12.516389; geodeticDatum: WGS84; **Identification:** identifiedBy: Tommaso Fusco; dateIdentified: 2022; **Event:** samplingProtocol: Pitfall traps; eventDate: 2014-06-06; **Record Level:** collectionID: Roma3_5.8

##### Distribution

Widespread in the Mediterranean area. Mediterranean (MED) chorotype.

#### Tetragnathidae

Menge, 1866

E60F138B-BD66-5C8C-ACA7-06BE84168E38

#### Pachygnatha degeeri

Sundevall, 1830

5442C346-4630-58A1-B26D-F2D0E4198DA6

##### Materials

- **Type status:** Other material. **Occurrence:** recordedBy: Fattorini S., Di Giulio A.; individualCount: 1; sex: male; lifeStage: adult; occurrenceID: 170F8D8E-F009-596C-888E-96C5F2B16889; **Taxon:** scientificName: Pachygnathadegeeri Sundevall, 1830; order: Araneae; family: Tetragnathidae; genus: Pachygnatha; **Location:** country: Italy; countryCode: IT; stateProvince: Rome; county: Rome; municipality: Rome; locality: Appia Antica Regional Park, Rome; locationRemarks: Farnesiana; decimalLatitude: 41.839667; decimalLongitude: 12.525528; geodeticDatum: WGS84; **Identification:** identifiedBy: Tommaso Fusco; dateIdentified: 2022; **Event:** samplingProtocol: Pitfall traps; eventDate: 2014-06-19; **Record Level:** collectionID: Roma3_5.8
- **Type status:** Other material. **Occurrence:** recordedBy: Fattorini S., Di Giulio A.; individualCount: 1; sex: female; lifeStage: adult; occurrenceID: CFF89C0C-8FA6-56F5-90CC-AB8F1B8EC443; **Taxon:** scientificName: Pachygnathadegeeri Sundevall, 1830; order: Araneae; family: Tetragnathidae; genus: Pachygnatha; **Location:** country: Italy; countryCode: IT; stateProvince: Rome; county: Rome; municipality: Rome; locality: Appia Antica Regional Park, Rome; locationRemarks: Casal Verbeni; decimalLatitude: 41.815250; decimalLongitude: 12.552222; geodeticDatum: WGS84; **Identification:** identifiedBy: Tommaso Fusco; dateIdentified: 2022; **Event:** samplingProtocol: Pitfall traps; eventDate: 2014-06-13; **Record Level:** collectionID: Roma3_5.8
- **Type status:** Other material. **Occurrence:** recordedBy: Fattorini S., Di Giulio A.; individualCount: 1; sex: male; lifeStage: adult; occurrenceID: 1D5DBB62-9363-5218-90EA-57D460392D8D; **Taxon:** scientificName: Pachygnathadegeeri Sundevall, 1830; order: Araneae; family: Tetragnathidae; genus: Pachygnatha; **Location:** country: Italy; countryCode: IT; stateProvince: Rome; county: Rome; municipality: Rome; locality: Appia Antica Regional Park, Rome; locationRemarks: Acqua Santa; decimalLatitude: 41.850561; decimalLongitude: 12.530861; geodeticDatum: WGS84; **Identification:** identifiedBy: Tommaso Fusco; dateIdentified: 2022; **Event:** samplingProtocol: Pitfall traps; eventDate: 2014-05-28; **Record Level:** collectionID: Roma3_5.8
- **Type status:** Other material. **Occurrence:** recordedBy: Fattorini S., Di Giulio A.; individualCount: 1; sex: female; lifeStage: adult; occurrenceID: EF79D5C8-0F68-54D2-A1E2-D91839DA23E5; **Taxon:** scientificName: Pachygnathadegeeri Sundevall, 1830; **Location:** country: Italy; countryCode: IT; stateProvince: Rome; county: Rome; municipality: Rome; locality: Appia Antica Regional Park, Rome; locationRemarks: Farnesiana; decimalLatitude: 41.839667; decimalLongitude: 12.525528; geodeticDatum: WGS84; **Identification:** identifiedBy: Tommaso Fusco; dateIdentified: 2022; **Event:** samplingProtocol: Pitfall traps; eventDate: 2014-06-09; **Record Level:** collectionID: Roma3_5.8
- **Type status:** Other material. **Occurrence:** recordedBy: Fattorini S., Di Giulio A.; individualCount: 1; sex: female; lifeStage: adult; occurrenceID: 25113A79-0059-56B2-B4A9-85C4375B93EB; **Taxon:** scientificName: Pachygnathadegeeri Sundevall, 1830; **Location:** country: Italy; countryCode: IT; stateProvince: Rome; county: Rome; municipality: Rome; locality: Appia Antica Regional Park, Rome; locationRemarks: Caffarella Sud 2; decimalLatitude: 41.856742; decimalLongitude: 12.529453; geodeticDatum: WGS84; **Identification:** identifiedBy: Tommaso Fusco; dateIdentified: 2022; **Event:** samplingProtocol: Pitfall traps; eventDate: 2014-05-19; **Record Level:** collectionID: Roma3_5.8

##### Distribution

Azores, North Africa, Europe, Turkey, Caucasus, Russia (Europe to Far East), Iran, Central Asia, China. Palaearctic (PAL) chorotype.

#### Theridiidae

Sundevall, 1833

CF6A808B-8412-565A-B769-5F79DFCAC5E8

#### Asagena italica

(Knoflach, 1996)

2FEA706E-EACF-5990-BA5D-76A28855863E

##### Materials

- **Type status:** Other material. **Occurrence:** recordedBy: Fattorini S., Di Giulio A.; individualCount: 1; sex: male; lifeStage: adult; occurrenceID: B63C07E6-C1F9-5BB9-A1DD-C8ACBAB3EDFD; **Taxon:** scientificName: Asagenaitalica (Knoflach, 1996); order: Araneae; family: Theridiidae; genus: Asagnea; **Location:** country: Italy; countryCode: IT; stateProvince: Rome; county: Rome; municipality: Rome; locality: Appia Antica Regional Park, Rome; locationRemarks: Caffarella Centro; decimalLatitude: 41.864889; decimalLongitude: 12.516389; geodeticDatum: WGS84; **Identification:** identifiedBy: Tommaso Fusco; dateIdentified: 2022; **Event:** samplingProtocol: Pitfall traps; eventDate: 2014-06-06; **Record Level:** collectionID: Roma3_5.8

##### Distribution

France (including Corsica), Switzerland, Italy, Algeria. W-Mediterranean (WME) chorotype.

#### Crustulina guttata

(Wider, 1834)

C0EEA7C1-5B11-5CD9-A7A4-295098A93B2A

##### Materials

- **Type status:** Other material. **Occurrence:** recordedBy: Fattorini S., Di Giulio A.; individualCount: 1; sex: Male; lifeStage: adult; occurrenceID: 8177331B-DFA6-5B73-9169-9C384FDADF16; **Taxon:** scientificName: Crustulinaguttata (Wider, 1834); order: Araneae; family: Theridiidae; genus: Crustulina; **Location:** country: Italy; countryCode: IT; stateProvince: Rome; county: Rome; municipality: Rome; locality: Appia Antica Regional Park, Rome; locationRemarks: Tor Marancia; decimalLatitude: 41.850308; decimalLongitude: 12.503178; geodeticDatum: WGS84; **Identification:** identifiedBy: Tommaso Fusco; dateIdentified: 2022; **Event:** samplingProtocol: Pitfall traps; eventDate: 2014-05-15; **Record Level:** collectionID: Roma3_5.8

##### Distribution

Canary Islands, Europe, Caucasus, Russia (Europe to south Siberia), Kazakhstan, Iran, Central Asia, China, Korea, Japan. Palaearctic (PAL) chorotype.

#### Crustulina scabripes

Simon, 1881

06BCF8F2-1E17-59F8-90BF-2290E9201310

##### Materials

- **Type status:** Other material. **Occurrence:** recordedBy: Fattorini S., Di Giulio A.; individualCount: 1; sex: male; lifeStage: adult; occurrenceID: 16114C2B-05AE-59C8-A80A-AD8A7B015BE6; **Taxon:** scientificName: Crustulinascabripes Simon, 1881; order: Araneae; family: Theridiidae; genus: Crustulina; **Location:** country: Italy; countryCode: IT; stateProvince: Rome; county: Rome; municipality: Rome; locality: Appia Antica Regional Park, Rome; locationRemarks: Caffarella centro; decimalLatitude: 41.864889; decimalLongitude: 12.516389; geodeticDatum: WGS84; **Identification:** identifiedBy: Tommaso Fusco; dateIdentified: 2022; **Event:** samplingProtocol: Pitfall traps; eventDate: 2014-06-06; **Record Level:** collectionID: Roma3_5.8
- **Type status:** Other material. **Occurrence:** recordedBy: Fattorini S., Di Giulio A.; individualCount: 1; sex: female; lifeStage: adult; occurrenceID: 527D845F-A7D2-5C52-B644-3C07530CB7DD; **Taxon:** scientificName: Crustulinascabripes Simon, 1881; order: Araneae; family: Theridiidae; genus: Crustulina; **Location:** country: Italy; countryCode: IT; stateProvince: Rome; county: Rome; municipality: Rome; locality: Appia Antica Regional Park, Rome; locationRemarks: Farnesiana; decimalLatitude: 41.839667; decimalLongitude: 12.525528; geodeticDatum: WGS84; **Identification:** identifiedBy: Tommaso Fusco; dateIdentified: 2022; **Event:** samplingProtocol: Pitfall traps; eventDate: 2014-06-09; **Record Level:** collectionID: Roma3_5.8

##### Distribution

Widespread in the Mediterranean area. Mediterranean (MED) chorotype.

#### Enoplognata mandibularis

(Lucas, 1846)

78599236-A8CE-5801-9432-D9A82EADA3C3

##### Materials

- **Type status:** Other material. **Occurrence:** recordedBy: Fattorini S., Di Giulio A.; individualCount: 2; sex: 1 male, 1 female; lifeStage: adult; occurrenceID: 004D3D88-7A98-5E53-A9D1-7783A3C6C27A; **Taxon:** scientificName: Enoplognatha mandibularis (Lucas, 1846); order: Araneae; family: Theridiidae; genus: Enoplognatha; **Location:** country: Italy; countryCode: IT; stateProvince: Rome; county: Rome; municipality: Rome; locality: Appia Antica Regional Park, Rome; locationRemarks: Acqua Santa; decimalLatitude: 41.850561; decimalLongitude: 12.530861; geodeticDatum: WGS84; **Identification:** identifiedBy: Tommaso Fusco; dateIdentified: 2022; **Event:** samplingProtocol: Pitfall traps; eventDate: 2013-12-02; **Record Level:** collectionID: Roma3_5.8
- **Type status:** Other material. **Occurrence:** recordedBy: Fattorini S., Di Giulio A.; individualCount: 2; sex: 1 male, 1 female; lifeStage: adult; occurrenceID: 6B39A710-F383-55B2-BB19-42833EAF5734; **Taxon:** scientificName: Enoplognatha mandibularis (Lucas, 1846); order: Araneae; family: Theridiidae; genus: Enoplognatha; **Location:** country: Italy; countryCode: IT; stateProvince: Rome; county: Rome; municipality: Rome; locality: Appia Antica Regional Park, Rome; locationRemarks: Caffarella Centro; decimalLatitude: 41.864889; decimalLongitude: 12.516389; geodeticDatum: WGS84; **Identification:** identifiedBy: Tommaso Fusco; dateIdentified: 2022; **Event:** samplingProtocol: Pitfall traps; eventDate: 2013-11-13; **Record Level:** collectionID: Roma3_5.8
- **Type status:** Other material. **Occurrence:** recordedBy: Fattorini S., Di Giulio A.; individualCount: 2; sex: 1 male, 1 female; lifeStage: adult; occurrenceID: B6F1B68C-7FF5-592D-8CDA-E1161826B5AF; **Taxon:** scientificName: Enoplognatha mandibularis (Lucas, 1846); order: Araneae; family: Theridiidae; genus: Enoplognatha; **Location:** country: Italy; countryCode: IT; stateProvince: Rome; county: Rome; municipality: Rome; locality: Appia Antica Regional Park, Rome; locationRemarks: Caffarella Sud; decimalLatitude: 41.857247; decimalLongitude: 12.529211; geodeticDatum: WGS84; **Identification:** identifiedBy: Tommaso Fusco; dateIdentified: 2022; **Event:** samplingProtocol: Pitfall traps; eventDate: 2013-11-20; **Record Level:** collectionID: Roma3_5.8
- **Type status:** Other material. **Occurrence:** recordedBy: Fattorini S., Di Giulio A.; individualCount: 1; sex: male; lifeStage: adult; occurrenceID: 84864C21-5012-51E7-9788-2F54793A5F17; **Taxon:** scientificName: Enoplognatha mandibularis (Lucas, 1846); order: Araneae; family: Theridiidae; genus: Enoplognatha; **Location:** country: Italy; countryCode: IT; stateProvince: Rome; county: Rome; municipality: Rome; locality: Appia Antica Regional Park, Rome; locationRemarks: Caffarella Sud; decimalLatitude: 41.857247; decimalLongitude: 12.529211; geodeticDatum: WGS84; **Identification:** identifiedBy: Tommaso Fusco; dateIdentified: 2022; **Event:** samplingProtocol: Pitfall traps; eventDate: 2013-11-30; **Record Level:** collectionID: Roma3_5.8
- **Type status:** Other material. **Occurrence:** recordedBy: Fattorini S., Di Giulio A.; individualCount: 2; sex: 1 male, 1 female; lifeStage: adult; occurrenceID: 5DD856BD-7D7D-5EB3-903D-9189A115B56E; **Taxon:** scientificName: Enoplognatha mandibularis (Lucas, 1846); order: Araneae; family: Theridiidae; genus: Enoplognatha; **Location:** country: Italy; countryCode: IT; stateProvince: Rome; county: Rome; municipality: Rome; locality: Appia Antica Regional Park, Rome; locationRemarks: Casal Verbeni; decimalLatitude: 41.815250; decimalLongitude: 12.552222; geodeticDatum: WGS84; **Identification:** identifiedBy: Tommaso Fusco; dateIdentified: 2022; **Event:** samplingProtocol: Pitfall traps; eventDate: 2013-11-18; **Record Level:** collectionID: Roma3_5.8
- **Type status:** Other material. **Occurrence:** recordedBy: Fattorini S., Di Giulio A.; individualCount: 1; sex: male; lifeStage: adult; occurrenceID: ED71DA00-2673-5D12-9A1E-D99967B2966C; **Taxon:** scientificName: Enoplognatha mandibularis (Lucas, 1846); order: Araneae; family: Theridiidae; genus: Enoplognatha; **Location:** country: Italy; countryCode: IT; stateProvince: Rome; county: Rome; municipality: Rome; locality: Appia Antica Regional Park, Rome; locationRemarks: Casal Verbeni; decimalLatitude: 41.815250; decimalLongitude: 12.552222; geodeticDatum: WGS84; **Identification:** identifiedBy: Tommaso Fusco; dateIdentified: 2022; **Event:** samplingProtocol: Pitfall traps; eventDate: 2013-11-27; **Record Level:** collectionID: Roma3_5.8
- **Type status:** Other material. **Occurrence:** recordedBy: Fattorini S., Di Giulio A.; individualCount: 1; sex: 1 male, 1 female; lifeStage: adult; occurrenceID: D7C25E4C-4021-512D-BCE9-AEDF039D6FB9; **Taxon:** scientificName: Enoplognatha mandibularis (Lucas, 1846); order: Araneae; family: Theridiidae; genus: Enoplognatha; **Location:** country: Italy; countryCode: IT; stateProvince: Rome; county: Rome; municipality: Rome; locality: Appia Antica Regional Park, Rome; locationRemarks: Casal Verbeni; decimalLatitude: 41.815250; decimalLongitude: 12.552222; geodeticDatum: WGS84; **Identification:** identifiedBy: Tommaso Fusco; dateIdentified: 2022; **Event:** samplingProtocol: Pitfall traps; eventDate: 2013-12-05; **Record Level:** collectionID: Roma3_5.8
- **Type status:** Other material. **Occurrence:** recordedBy: Fattorini S., Di Giulio A.; individualCount: 2; sex: male; lifeStage: adult; occurrenceID: 99BBA98C-9390-5048-B8A3-FD19B423EF8A; **Taxon:** scientificName: Enoplognatha mandibularis (Lucas, 1846); order: Araneae; family: Theridiidae; genus: Enoplognatha; **Location:** country: Italy; countryCode: IT; stateProvince: Rome; county: Rome; municipality: Rome; locality: Appia Antica Regional Park, Rome; locationRemarks: Cava Fiorucci; decimalLatitude: 41.834106; decimalLongitude: 12.549264; geodeticDatum: WGS84; **Identification:** identifiedBy: Tommaso Fusco; dateIdentified: 2022; **Event:** samplingProtocol: Pitfall traps; eventDate: 2013-11-27; **Record Level:** collectionID: Roma3_5.8
- **Type status:** Other material. **Occurrence:** recordedBy: Fattorini S., Di Giulio A.; individualCount: 2; sex: male; lifeStage: adult; occurrenceID: D59C01C7-D2F0-59A4-81D3-968C392BCCDC; **Taxon:** scientificName: Enoplognatha mandibularis (Lucas, 1846); order: Araneae; family: Theridiidae; genus: Enoplognatha; **Location:** country: Italy; countryCode: IT; stateProvince: Rome; county: Rome; municipality: Rome; locality: Appia Antica Regional Park, Rome; locationRemarks: Farnesiana; decimalLatitude: 41.839667; decimalLongitude: 12.525528; geodeticDatum: WGS84; **Identification:** identifiedBy: Tommaso Fusco; dateIdentified: 2022; **Event:** samplingProtocol: Pitfall traps; eventDate: 2013-11-13; **Record Level:** collectionID: Roma3_5.8
- **Type status:** Other material. **Occurrence:** recordedBy: Fattorini S., Di Giulio A.; individualCount: 2; sex: male; lifeStage: adult; occurrenceID: C356C759-E296-5103-A9D6-8433A974AB05; **Taxon:** scientificName: Enoplognatha mandibularis (Lucas, 1846); order: Araneae; family: Theridiidae; genus: Enoplognatha; **Location:** country: Italy; countryCode: IT; stateProvince: Rome; county: Rome; municipality: Rome; locality: Appia Antica Regional Park, Rome; locationRemarks: Farnesiana; decimalLatitude: 41.839667; decimalLongitude: 12.525528; geodeticDatum: WGS84; **Identification:** identifiedBy: Tommaso Fusco; dateIdentified: 2022; **Event:** samplingProtocol: Pitfall traps; eventDate: 2013-11-25; **Record Level:** collectionID: Roma3_5.8
- **Type status:** Other material. **Occurrence:** recordedBy: Fattorini S., Di Giulio A.; individualCount: 3; sex: male; lifeStage: adult; occurrenceID: 58E9BC49-96B5-518A-B32C-1DAAE6D95C4E; **Taxon:** scientificName: Enoplognatha mandibularis (Lucas, 1846); order: Araneae; family: Theridiidae; genus: Enoplognatha; **Location:** country: Italy; countryCode: IT; stateProvince: Rome; county: Rome; municipality: Rome; locality: Appia Antica Regional Park, Rome; locationRemarks: San Sebastiano; decimalLatitude: 41.855733; decimalLongitude: 12.515114; geodeticDatum: WGS84; **Identification:** identifiedBy: Tommaso Fusco; dateIdentified: 2022; **Event:** samplingProtocol: Pitfall traps; eventDate: 2013-11-25; **Record Level:** collectionID: Roma3_5.8
- **Type status:** Other material. **Occurrence:** recordedBy: Fattorini S., Di Giulio A.; individualCount: 1; sex: male; lifeStage: adult; occurrenceID: E042EE0B-7448-5D76-B128-35791400FBF6; **Taxon:** scientificName: Enoplognatha mandibularis (Lucas, 1846); order: Araneae; family: Theridiidae; genus: Enoplognatha; **Location:** country: Italy; countryCode: IT; stateProvince: Rome; county: Rome; municipality: Rome; locality: Appia Antica Regional Park, Rome; locationRemarks: San Sebastiano; decimalLatitude: 41.855733; decimalLongitude: 12.515114; geodeticDatum: WGS84; **Identification:** identifiedBy: Tommaso Fusco; dateIdentified: 2022; **Event:** samplingProtocol: Pitfall traps; eventDate: 2013-12-02; **Record Level:** collectionID: Roma3_5.8
- **Type status:** Other material. **Occurrence:** recordedBy: Fattorini S., Di Giulio A.; individualCount: 1; sex: male; lifeStage: adult; occurrenceID: D4770E6B-6DF5-54B9-B09D-1A6912EC6455; **Taxon:** scientificName: Enoplognatha mandibularis (Lucas, 1846); order: Araneae; family: Theridiidae; genus: Enoplognatha; **Location:** country: Italy; countryCode: IT; stateProvince: Rome; county: Rome; municipality: Rome; locality: Appia Antica Regional Park, Rome; locationRemarks: Tor Marancia; decimalLatitude: 41.850308; decimalLongitude: 12.503178; geodeticDatum: WGS84; **Identification:** identifiedBy: Tommaso Fusco; dateIdentified: 2022; **Event:** samplingProtocol: Pitfall traps; eventDate: 2013-11-18; **Record Level:** collectionID: Roma3_5.8
- **Type status:** Other material. **Occurrence:** recordedBy: Fattorini S., Di Giulio A.; individualCount: 1; sex: male; lifeStage: adult; occurrenceID: A3E2AFD3-1C9E-5A08-B100-97AE29D37E9E; **Taxon:** scientificName: Enoplognatha mandibularis (Lucas, 1846); order: Araneae; family: Theridiidae; genus: Enoplognatha; **Location:** country: Italy; countryCode: IT; stateProvince: Rome; county: Rome; municipality: Rome; locality: Appia Antica Regional Park, Rome; locationRemarks: Tor Marancia; decimalLatitude: 41.850308; decimalLongitude: 12.503178; geodeticDatum: WGS84; **Identification:** identifiedBy: Tommaso Fusco; dateIdentified: 2022; **Event:** samplingProtocol: Pitfall traps; eventDate: 2013-11-27; **Record Level:** collectionID: Roma3_5.8
- **Type status:** Other material. **Occurrence:** recordedBy: Fattorini S., Di Giulio A.; individualCount: 1; sex: male; lifeStage: adult; occurrenceID: FB94E04A-76DC-5EA3-8B25-35203718677C; **Taxon:** scientificName: Enoplognatha mandibularis (Lucas, 1846); order: Araneae; family: Theridiidae; genus: Enoplognatha; **Location:** country: Italy; countryCode: IT; stateProvince: Rome; county: Rome; municipality: Rome; locality: Appia Antica Regional Park, Rome; locationRemarks: Torre Selce; decimalLatitude: 41.816611; decimalLongitude: 12.560667; geodeticDatum: WGS84; **Identification:** identifiedBy: Tommaso Fusco; dateIdentified: 2022; **Event:** samplingProtocol: Pitfall traps; eventDate: 2013-11-07; **Record Level:** collectionID: Roma3_5.8
- **Type status:** Other material. **Occurrence:** recordedBy: Fattorini S., Di Giulio A.; individualCount: 1; sex: male; lifeStage: adult; occurrenceID: 8C07E32F-FF1A-52B4-89A6-611271287DD8; **Taxon:** scientificName: Enoplognatha mandibularis (Lucas, 1846); order: Araneae; family: Theridiidae; genus: Enoplognatha; **Location:** country: Italy; countryCode: IT; stateProvince: Rome; county: Rome; municipality: Rome; locality: Appia Antica Regional Park, Rome; locationRemarks: Torre Selce; decimalLatitude: 41.816611; decimalLongitude: 12.560667; geodeticDatum: WGS84; **Identification:** identifiedBy: Tommaso Fusco; dateIdentified: 2022; **Event:** samplingProtocol: Pitfall traps; eventDate: 2013-11-18; **Record Level:** collectionID: Roma3_5.8
- **Type status:** Other material. **Occurrence:** recordedBy: Fattorini S., Di Giulio A.; individualCount: 5; sex: 4 male, 1 female; lifeStage: adult; occurrenceID: 3B94ED66-D849-5900-9207-AD64FB664ED1; **Taxon:** scientificName: Enoplognatha mandibularis (Lucas, 1846); order: Araneae; family: Theridiidae; genus: Enoplognatha; **Location:** country: Italy; countryCode: IT; stateProvince: Rome; county: Rome; municipality: Rome; locality: Appia Antica Regional Park, Rome; locationRemarks: Torre Selce; decimalLatitude: 41.816611; decimalLongitude: 12.560667; geodeticDatum: WGS84; **Identification:** identifiedBy: Tommaso Fusco; dateIdentified: 2022; **Event:** samplingProtocol: Pitfall traps; eventDate: 2013-11-27; **Record Level:** collectionID: Roma3_5.8
- **Type status:** Other material. **Occurrence:** recordedBy: Fattorini S., Di Giulio A.; individualCount: 4; sex: 3 male, 1 female; lifeStage: adult; occurrenceID: E3F18227-29A3-5E63-9300-8B46DF60134B; **Taxon:** scientificName: Enoplognatha mandibularis (Lucas, 1846); order: Araneae; family: Theridiidae; genus: Enoplognatha; **Location:** country: Italy; countryCode: IT; stateProvince: Rome; county: Rome; municipality: Rome; locality: Appia Antica Regional Park, Rome; locationRemarks: Torre Selce; decimalLatitude: 41.816611; decimalLongitude: 12.560667; geodeticDatum: WGS84; **Identification:** identifiedBy: Tommaso Fusco; dateIdentified: 2022; **Event:** samplingProtocol: Pitfall traps; eventDate: 2013-12-06; **Record Level:** collectionID: Roma3_5.8

##### Distribution

Europe, North Africa, Turkey, Israel, Russia (Europe) to Azerbaijan, China. Palaearctic (PAL) chorotype.

#### Enoplognata testacea

Simon, 1884

7B0B4425-AB69-5065-A5E8-CAE7E3158BA9

##### Materials

- **Type status:** Other material. **Occurrence:** recordedBy: Fattorini S., Di Giulio A.; individualCount: 1; sex: female; lifeStage: adult; occurrenceID: 70B95264-B02F-595E-930F-2F0D4A5C94FC; **Taxon:** scientificName: Enoplognatha testacea Simon, 1884; order: Araneae; family: Theridiidae; genus: Enoplognatha; **Location:** country: Italy; countryCode: IT; stateProvince: Rome; county: Rome; municipality: Rome; locality: Appia Antica Regional Park, Rome; locationRemarks: Appia Antica; decimalLatitude: 41.812575; decimalLongitude: 12.564011; geodeticDatum: WGS84; **Identification:** identifiedBy: Tommaso Fusco; dateIdentified: 2022; **Event:** samplingProtocol: Pitfall traps; eventDate: 2013-12-06; **Record Level:** collectionID: Roma3_5.8
- **Type status:** Other material. **Occurrence:** recordedBy: Fattorini S., Di Giulio A.; individualCount: 1; sex: male; lifeStage: adult; occurrenceID: C23530AF-F2FD-5C85-8BF2-2B21CF973A40; **Taxon:** scientificName: Enoplognatha testacea Simon, 1884; order: Araneae; family: Theridiidae; genus: Enoplognatha; **Location:** country: Italy; countryCode: IT; stateProvince: Rome; county: Rome; municipality: Rome; locality: Appia Antica Regional Park, Rome; locationRemarks: Caffarella Centro; decimalLatitude: 41.864889; decimalLongitude: 12.516389; geodeticDatum: WGS84; **Identification:** identifiedBy: Tommaso Fusco; dateIdentified: 2022; **Event:** samplingProtocol: Pitfall traps; eventDate: 2013-11-13; **Record Level:** collectionID: Roma3_5.8
- **Type status:** Other material. **Occurrence:** recordedBy: Fattorini S., Di Giulio A.; individualCount: 2; sex: male; lifeStage: adult; occurrenceID: 33DD6D7F-3526-57D4-A601-E9778E4C9DD8; **Taxon:** scientificName: Enoplognatha testacea Simon, 1884; order: Araneae; family: Theridiidae; genus: Enoplognatha; **Location:** country: Italy; countryCode: IT; stateProvince: Rome; county: Rome; municipality: Rome; locality: Appia Antica Regional Park, Rome; locationRemarks: Caffarella Centro; decimalLatitude: 41.864889; decimalLongitude: 12.516389; geodeticDatum: WGS84; **Identification:** identifiedBy: Tommaso Fusco; dateIdentified: 2022; **Event:** samplingProtocol: Pitfall traps; eventDate: 2013-11-30; **Record Level:** collectionID: Roma3_5.8
- **Type status:** Other material. **Occurrence:** recordedBy: Fattorini S., Di Giulio A.; individualCount: 1; sex: male; lifeStage: adult; occurrenceID: C1A9B802-2F09-59AB-BBD9-843B46EA779E; **Taxon:** scientificName: Enoplognatha testacea Simon, 1884; order: Araneae; family: Theridiidae; genus: Enoplognatha; **Location:** country: Italy; countryCode: IT; stateProvince: Rome; county: Rome; municipality: Rome; locality: Appia Antica Regional Park, Rome; locationRemarks: Caffarella Nord; decimalLatitude: 41.867753; decimalLongitude: 12.512414; geodeticDatum: WGS84; **Identification:** identifiedBy: Tommaso Fusco; dateIdentified: 2022; **Event:** samplingProtocol: Pitfall traps; eventDate: 2013-11-30; **Record Level:** collectionID: Roma3_5.8
- **Type status:** Other material. **Occurrence:** recordedBy: Fattorini S., Di Giulio A.; individualCount: 6; sex: male; lifeStage: adult; occurrenceID: C29BDAAF-9E73-50F2-98D8-3D2FCC507ED8; **Taxon:** scientificName: Enoplognatha testacea Simon, 1884; order: Araneae; family: Theridiidae; genus: Enoplognatha; **Location:** country: Italy; countryCode: IT; stateProvince: Rome; county: Rome; municipality: Rome; locality: Appia Antica Regional Park, Rome; locationRemarks: Caffarella Sud; decimalLatitude: 41.857247; decimalLongitude: 12.529211; geodeticDatum: WGS84; **Identification:** identifiedBy: Tommaso Fusco; dateIdentified: 2022; **Event:** samplingProtocol: Pitfall traps; eventDate: 2013-11-12; **Record Level:** collectionID: Roma3_5.8
- **Type status:** Other material. **Occurrence:** recordedBy: Fattorini S., Di Giulio A.; individualCount: 2; sex: male; lifeStage: adult; occurrenceID: B922C385-EED4-517B-926D-8B3FF5C8179F; **Taxon:** scientificName: Enoplognatha testacea Simon, 1884; order: Araneae; family: Theridiidae; genus: Enoplognatha; **Location:** country: Italy; countryCode: IT; stateProvince: Rome; county: Rome; municipality: Rome; locality: Appia Antica Regional Park, Rome; locationRemarks: Caffarella Sud; decimalLatitude: 41.857247; decimalLongitude: 12.529211; geodeticDatum: WGS84; **Identification:** identifiedBy: Tommaso Fusco; dateIdentified: 2022; **Event:** samplingProtocol: Pitfall traps; eventDate: 2013-11-20; **Record Level:** collectionID: Roma3_5.8
- **Type status:** Other material. **Occurrence:** recordedBy: Fattorini S., Di Giulio A.; individualCount: 1; sex: female; lifeStage: adult; occurrenceID: 0B8EA2A7-C336-5066-95DA-D8C6F732C5E1; **Taxon:** scientificName: Enoplognatha testacea Simon, 1884; order: Araneae; family: Theridiidae; genus: Enoplognatha; **Location:** country: Italy; countryCode: IT; stateProvince: Rome; county: Rome; municipality: Rome; locality: Appia Antica Regional Park, Rome; locationRemarks: Tor Marancia; decimalLatitude: 41.850308; decimalLongitude: 12.503178; geodeticDatum: WGS84; **Identification:** identifiedBy: Tommaso Fusco; dateIdentified: 2022; **Event:** samplingProtocol: Pitfall traps; eventDate: 2014-05-15; **Record Level:** collectionID: Roma3_5.8
- **Type status:** Other material. **Occurrence:** recordedBy: Fattorini S., Di Giulio A.; individualCount: 1; sex: female; lifeStage: adult; occurrenceID: 977EEAFE-8421-5ECE-B924-D9CAB2A95D22; **Taxon:** scientificName: Enoplognatha testacea Simon, 1884; order: Araneae; family: Theridiidae; genus: Enoplognatha; **Location:** country: Italy; countryCode: IT; stateProvince: Rome; county: Rome; municipality: Rome; locality: Appia Antica Regional Park, Rome; locationRemarks: Tor Marancia; decimalLatitude: 41.850308; decimalLongitude: 12.503178; geodeticDatum: WGS84; **Identification:** identifiedBy: Tommaso Fusco; dateIdentified: 2022; **Event:** samplingProtocol: Pitfall traps; eventDate: 2013-11-07; **Record Level:** collectionID: Roma3_5.8
- **Type status:** Other material. **Occurrence:** recordedBy: Fattorini S., Di Giulio A.; individualCount: 1; sex: male; lifeStage: adult; occurrenceID: 8DBDFDFA-118A-553B-AE84-CF82745E846B; **Taxon:** scientificName: Enoplognatha testacea Simon, 1884; order: Araneae; family: Theridiidae; genus: Enoplognatha; **Location:** country: Italy; countryCode: IT; stateProvince: Rome; county: Rome; municipality: Rome; locality: Appia Antica Regional Park, Rome; locationRemarks: Torre Selce; decimalLatitude: 41.816611; decimalLongitude: 12.560667; geodeticDatum: WGS84; **Identification:** identifiedBy: Tommaso Fusco; dateIdentified: 2022; **Event:** samplingProtocol: Pitfall traps; eventDate: 2013-11-27; **Record Level:** collectionID: Roma3_5.8

##### Distribution

Southern, central Europe to Caucasus. S-European (SEU) chorotype.

#### Enoplognata thoracica

(Hahn, 1833)

7A7D6CF9-719F-51A6-8B1C-D08FD9A000E5

##### Materials

- **Type status:** Other material. **Occurrence:** recordedBy: Fattorini S., Di Giulio A.; individualCount: 1; sex: male; lifeStage: adult; occurrenceID: C2C90007-DF95-5B61-8696-2ECBC62DA1EB; **Taxon:** scientificName: Enoplognathathoracica (Hahn, 1833); order: Araneae; family: Theridiidae; genus: Enoplognatha; **Location:** country: Italy; countryCode: IT; stateProvince: Rome; county: Rome; municipality: Rome; locality: Appia Antica Regional Park, Rome; locationRemarks: Farnesiana; decimalLatitude: 41.839667; decimalLongitude: 12.525528; geodeticDatum: WGS84; **Identification:** identifiedBy: Tommaso Fusco; dateIdentified: 2022; **Event:** samplingProtocol: Pitfall traps; eventDate: 2014-06-19; **Record Level:** collectionID: Roma3_5.8
- **Type status:** Other material. **Occurrence:** recordedBy: Fattorini S., Di Giulio A.; individualCount: 1; sex: male; lifeStage: adult; occurrenceID: E413CC57-3097-5AAF-AF7D-1536D25B04AB; **Taxon:** scientificName: Enoplognathathoracica (Hahn, 1833); order: Araneae; family: Theridiidae; genus: Enoplognatha; **Location:** country: Italy; countryCode: IT; stateProvince: Rome; county: Rome; municipality: Rome; locality: Appia Antica Regional Park, Rome; locationRemarks: Tor Marancia; decimalLatitude: 41.850308; decimalLongitude: 12.503178; geodeticDatum: WGS84; **Identification:** identifiedBy: Tommaso Fusco; dateIdentified: 2022; **Event:** samplingProtocol: Pitfall traps; eventDate: 2014-05-15; **Record Level:** collectionID: Roma3_5.8

##### Distribution

Europe, North Africa, Turkey, Caucasus, Syria, Iran, Turkmenistan. Introduced to North America. Turano-Europeo-Mediterranean (TEM) chorotype.

#### Euryopis dentigera

Simon, 1880

1E3E1AC9-3E81-541F-8DFE-F5C048B4DE64

##### Materials

- **Type status:** Other material. **Occurrence:** recordedBy: Fattorini S., Di Giulio A.; individualCount: 1; sex: female; lifeStage: adult; occurrenceID: 63A7E7BF-004E-5384-931D-709128BBEA67; **Taxon:** scientificName: Euryopisdentigera Simon, 1880; order: Araneae; family: Theridiidae; genus: Euryopis; **Location:** country: Italy; countryCode: IT; stateProvince: Rome; county: Rome; municipality: Rome; locality: Appia Antica Regional Park, Rome; locationRemarks: Cava Fiorucci; decimalLatitude: 41.834106; decimalLongitude: 12.549264; geodeticDatum: WGS84; **Identification:** identifiedBy: Tommaso Fusco; dateIdentified: 2022; **Event:** samplingProtocol: Pitfall traps; eventDate: 2014-06-13; **Record Level:** collectionID: Roma3_5.8
- **Type status:** Other material. **Occurrence:** recordedBy: Fattorini S., Di Giulio A.; individualCount: 1; sex: male; lifeStage: adult; occurrenceID: F726E99A-33CC-5F93-9311-AC9A48DF79BC; **Taxon:** scientificName: Euryopisepisinoides (Walckenaer, 1847); order: Araneae; family: Theridiidae; genus: Euryopis; **Location:** country: Italy; countryCode: IT; stateProvince: Rome; county: Rome; municipality: Rome; locality: Appia Antica Regional Park, Rome; locationRemarks: Casal Verbeni; decimalLatitude: 41.815250; decimalLongitude: 12.552222; geodeticDatum: WGS84; **Identification:** identifiedBy: Tommaso Fusco; dateIdentified: 2022; **Event:** samplingProtocol: Pitfall traps; eventDate: 2014-05-26; **Record Level:** collectionID: Roma3_5.8
- **Type status:** Other material. **Occurrence:** recordedBy: Fattorini S., Di Giulio A.; individualCount: 1; sex: female; lifeStage: adult; occurrenceID: FFFCE429-FC1F-57A6-9C35-718FABE45E5E; **Taxon:** scientificName: Euryopisepisinoides (Walckenaer, 1847); order: Araneae; family: Theridiidae; genus: Euryopis; **Location:** country: Italy; countryCode: IT; stateProvince: Rome; county: Rome; municipality: Rome; locality: Appia Antica Regional Park, Rome; locationRemarks: Farnesiana; decimalLatitude: 41.839667; decimalLongitude: 12.525528; geodeticDatum: WGS84; **Identification:** identifiedBy: Tommaso Fusco; dateIdentified: 2022; **Event:** samplingProtocol: Pitfall traps; eventDate: 2013-10-31; **Record Level:** collectionID: Roma3_5.8

##### Distribution

Small and rare spider only found in some localities in Europe. European (EUR) chorotype.

##### Notes

**Figure 11.**
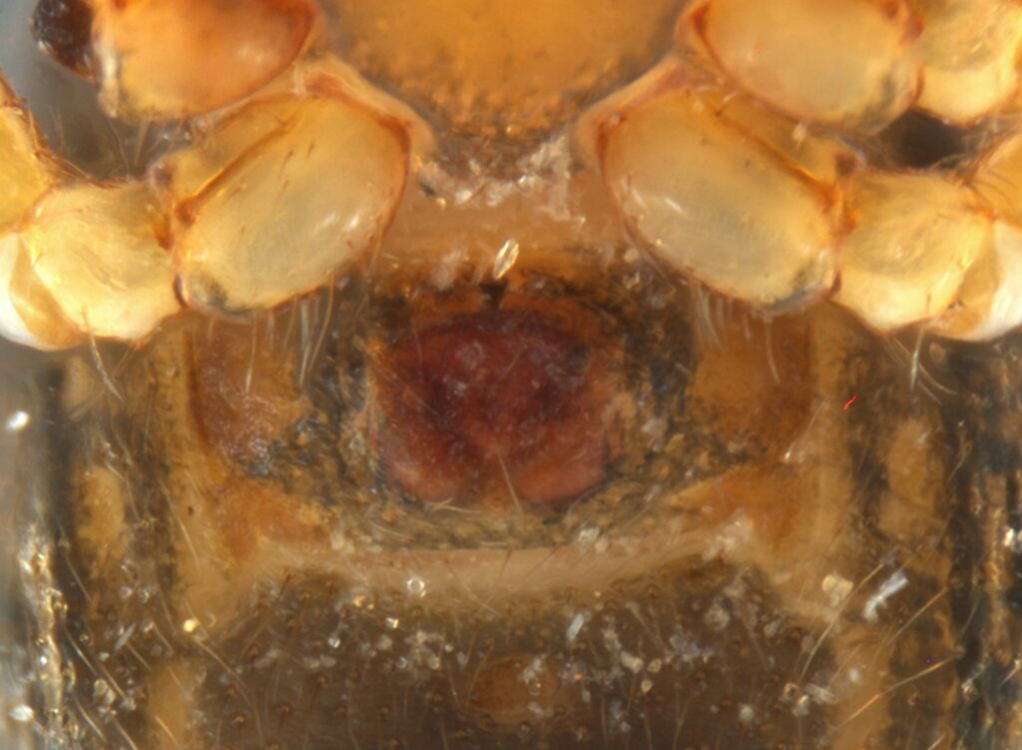
*Euryopisdentigera*, epigyne. Specimen collected in a protected green space in urban Rome, Italy (the Appia Antica Regional Park).

Habitus and epigyne in Figs 10, 11. Although a single female of this species was found, the epigyne matches that of *E.dentigera* illustrated by Oger (2024) and descibed by Kulczyński (1898).

#### Euryopis episinoides

(Walckenaer, 1847)

3F857EFB-165B-534A-8FE4-A332FE5006B0

##### Materials

- **Type status:** Other material. **Occurrence:** recordedBy: Fattorini S., Di Giulio A.; individualCount: 1; sex: male; lifeStage: adult; occurrenceID: 9D97C9D6-D44D-56C7-A6E8-D49BB6A82314; **Taxon:** scientificName: Euryopisepisinoides (Walckenaer, 1847); order: Araneae; family: Theridiidae; genus: Euryopis; **Location:** country: Italy; countryCode: IT; stateProvince: Rome; county: Rome; municipality: Rome; locality: Appia Antica Regional Park, Rome; locationRemarks: Casal Verbeni; decimalLatitude: 41.815250; decimalLongitude: 12.552222; geodeticDatum: WGS84; **Identification:** identifiedBy: Tommaso Fusco; dateIdentified: 2022; **Event:** samplingProtocol: Pitfall traps; eventDate: 2014-05-26; **Record Level:** collectionID: Roma3_5.8
- **Type status:** Other material. **Occurrence:** recordedBy: Fattorini S., Di Giulio A.; individualCount: 1; sex: female; lifeStage: adult; occurrenceID: 176961BC-146F-53C5-A16E-D1BA8751AB7D; **Taxon:** scientificName: Euryopisepisinoides (Walckenaer, 1847); order: Araneae; family: Theridiidae; genus: Euryopis; **Location:** country: Italy; countryCode: IT; stateProvince: Rome; county: Rome; municipality: Rome; locality: Appia Antica Regional Park, Rome; locationRemarks: Farnesiana; decimalLatitude: 41.839667; decimalLongitude: 12.525528; geodeticDatum: WGS84; **Identification:** identifiedBy: Tommaso Fusco; dateIdentified: 2022; **Event:** samplingProtocol: Pitfall traps; eventDate: 2013-10-31; **Record Level:** collectionID: Roma3_5.8

##### Distribution

Cabo Verde, Mediterranean to Turkey, Georgia, Israel. Introduced to South Africa, Reunion, India, China. Mediterranean (MED) chorotype.

#### Euryopis cfr sexalbomaculata

(Lucas, 1846)

55606ADD-4F76-5463-A487-224D54B85273

##### Materials

- **Type status:** Other material. **Occurrence:** recordedBy: Fattorini S., Di Giulio A.; individualCount: 1; sex: female; lifeStage: adult; occurrenceID: 8DEB554E-419F-57A7-8568-9F44E452B830; **Taxon:** scientificName: Euryopissexalbomaculata (Lucas, 1846); order: Araneae; family: Theridiidae; genus: Euryopis; **Location:** country: Italy; countryCode: IT; stateProvince: Rome; county: Rome; municipality: Rome; locality: Appia Antica Regional Park, Rome; locationRemarks: Torre Selce; decimalLatitude: 41.816611; decimalLongitude: 12.560667; geodeticDatum: WGS84; **Identification:** identifiedBy: Tommaso Fusco; dateIdentified: 2022; **Event:** samplingProtocol: Pitfall traps; eventDate: 2014-06-13; **Record Level:** collectionID: Roma3_5.8

##### Distribution

Mediterranean, Ukraine, Caucasus, Iran. Turano-Mediterranean (TUM) chorotype.

##### Notes

**Figure 13.**
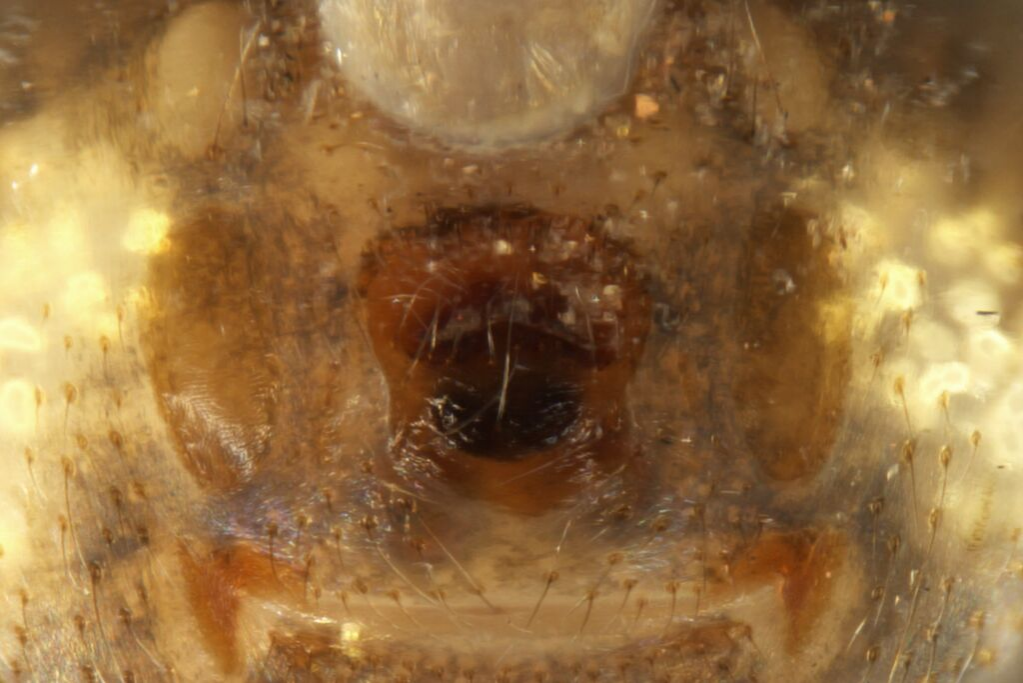
*Euryopiscfrsexalbomaculata*, epigyne. Specimen collected in a protected green space in urban Rome, Italy (the Appia Antica Regional Park).

Habitus and epigyne in Figs 12, 13. A single female of this species was found. The epigyne resembles that of *E.sexalbomaculata* illustrated by Oger (2024) and by Levy and Amitai (1981). A male specimen is needed to confirm this species.

#### Thomisidae

Sundevall, 1833

6287DE1B-244C-584A-B962-24171029FEE4

#### Bassanoides sp.

80154F55-5D0B-551F-B458-1535DC1499C1

##### Materials

- **Type status:** Other material. **Occurrence:** recordedBy: Fattorini S., Di Giulio A.; individualCount: 1; sex: female; lifeStage: adult; occurrenceID: D512B6C4-F48F-5670-88EB-682504334AB2; **Taxon:** scientificName: Bassaniodes Pocock, 1903; order: Araneae; family: Thomisidae; genus: Bassanoides; **Location:** country: Italy; countryCode: IT; stateProvince: Rome; county: Rome; municipality: Rome; locality: Appia Antica Regional Park, Rome; locationRemarks: Farnesiana; decimalLatitude: 41.839667; decimalLongitude: 12.525528; geodeticDatum: WGS84; **Identification:** identifiedBy: Tommaso Fusco; dateIdentified: 2022; **Event:** samplingProtocol: Pitfall traps; eventDate: 2014-06-09; **Record Level:** collectionID: Roma3_5.8

#### Ozyptila confluens

(C. L. Koch, 1845)

3904209C-AEEF-5C16-8ECD-C9DAB58591FB

##### Materials

- **Type status:** Other material. **Occurrence:** recordedBy: Fattorini S., Di Giulio A.; individualCount: 3; sex: male; lifeStage: adult; occurrenceID: B7D195C4-45F0-5675-9189-0BAB57FA8B6C; **Taxon:** scientificName: Ozyptilaconfluens (C. L. Koch, 1845); order: Araneae; family: Thomisidae; genus: Ozyptila; **Location:** country: Italy; countryCode: IT; stateProvince: Rome; county: Rome; municipality: Rome; locality: Appia Antica Regional Park, Rome; locationRemarks: Caffarella Centro; decimalLatitude: 41.864889; decimalLongitude: 12.516389; geodeticDatum: WGS84; **Identification:** identifiedBy: Tommaso Fusco; dateIdentified: 2022; **Event:** samplingProtocol: Pitfall traps; eventDate: 2013-10-31; **Record Level:** collectionID: Roma3_5.8
- **Type status:** Other material. **Occurrence:** recordedBy: Fattorini S., Di Giulio A.; individualCount: 1; sex: female; lifeStage: adult; occurrenceID: ACD1A094-2FCD-5BA6-9C9A-A88EEB2205E0; **Taxon:** scientificName: Ozyptilaconfluens (C. L. Koch, 1845); order: Araneae; family: Thomisidae; genus: Ozyptila; **Location:** country: Italy; countryCode: IT; stateProvince: Rome; county: Rome; municipality: Rome; locality: Appia Antica Regional Park, Rome; locationRemarks: Casal Verbeni; decimalLatitude: 41.815250; decimalLongitude: 12.552222; geodeticDatum: WGS84; **Identification:** identifiedBy: Tommaso Fusco; dateIdentified: 2022; **Event:** samplingProtocol: Pitfall traps; eventDate: 2014-05-26; **Record Level:** collectionID: Roma3_5.8
- **Type status:** Other material. **Occurrence:** recordedBy: Fattorini S., Di Giulio A.; individualCount: 1; sex: female; lifeStage: adult; occurrenceID: 0346A40C-7C95-54FB-9673-DDDB349B4773; **Taxon:** scientificName: Ozyptilaconfluens (C. L. Koch, 1845); order: Araneae; family: Thomisidae; genus: Ozyptila; **Location:** country: Italy; countryCode: IT; stateProvince: Rome; county: Rome; municipality: Rome; locality: Appia Antica Regional Park, Rome; locationRemarks: Cava Fiorucci; decimalLatitude: 41.834106; decimalLongitude: 12.549264; geodeticDatum: WGS84; **Identification:** identifiedBy: Tommaso Fusco; dateIdentified: 2022; **Event:** samplingProtocol: Pitfall traps; eventDate: 2014-06-13; **Record Level:** collectionID: Roma3_5.8
- **Type status:** Other material. **Occurrence:** recordedBy: Fattorini S., Di Giulio A.; individualCount: 1; sex: female; lifeStage: adult; occurrenceID: 7EFD72D4-9FD4-5446-AFDC-6976C9A6128D; **Taxon:** scientificName: Ozyptilaconfluens (C. L. Koch, 1845); order: Araneae; family: Thomisidae; genus: Ozyptila; **Location:** country: Italy; countryCode: IT; stateProvince: Rome; county: Rome; municipality: Rome; locality: Appia Antica Regional Park, Rome; locationRemarks: Caffarella Sud 3; decimalLatitude: 41.856928; decimalLongitude: 12.528406; geodeticDatum: WGS84; **Identification:** identifiedBy: Tommaso Fusco; dateIdentified: 2022; **Event:** samplingProtocol: Pitfall traps; eventDate: 2014-05-27; **Record Level:** collectionID: Roma3_5.8
- **Type status:** Other material. **Occurrence:** recordedBy: Fattorini S., Di Giulio A.; individualCount: 1; sex: female; lifeStage: adult; occurrenceID: CB1F9815-FFC6-5AC0-AC13-99A5C7530D58; **Taxon:** scientificName: Ozyptilaconfluens (C. L. Koch, 1845); order: Araneae; family: Thomisidae; genus: Ozyptila; **Location:** country: Italy; countryCode: IT; stateProvince: Rome; county: Rome; municipality: Rome; locality: Appia Antica Regional Park, Rome; locationRemarks: San Sebastiano; decimalLatitude: 41.855733; decimalLongitude: 12.515114; geodeticDatum: WGS84; **Identification:** identifiedBy: Tommaso Fusco; dateIdentified: 2022; **Event:** samplingProtocol: Pitfall traps; eventDate: 2014-05-28; **Record Level:** collectionID: Roma3_5.8
- **Type status:** Other material. **Occurrence:** recordedBy: Fattorini S., Di Giulio A.; individualCount: 1; sex: female; lifeStage: adult; occurrenceID: 0409AC92-A70A-58FD-A67A-A0465263D99F; **Taxon:** scientificName: Ozyptilaconfluens (C. L. Koch, 1845); order: Araneae; family: Thomisidae; genus: Ozyptila; **Location:** country: Italy; countryCode: IT; stateProvince: Rome; county: Rome; municipality: Rome; locality: Appia Antica Regional Park, Rome; locationRemarks: Torre Selce; decimalLatitude: 41.816611; decimalLongitude: 12.560667; geodeticDatum: WGS84; **Identification:** identifiedBy: Tommaso Fusco; dateIdentified: 2022; **Event:** samplingProtocol: Pitfall traps; eventDate: 2013-11-07; **Record Level:** collectionID: Roma3_5.8
- **Type status:** Other material. **Occurrence:** recordedBy: Fattorini S., Di Giulio A.; individualCount: 2; sex: male; lifeStage: adult; occurrenceID: 315F263D-C338-5D5F-BFBE-010E060B4053; **Taxon:** scientificName: Ozyptilaconfluens (C. L. Koch, 1845); order: Araneae; family: Thomisidae; genus: Ozyptila; **Location:** country: Italy; countryCode: IT; stateProvince: Rome; county: Rome; municipality: Rome; locality: Appia Antica Regional Park, Rome; locationRemarks: Farnesiana; decimalLatitude: 41.839667; decimalLongitude: 12.525528; geodeticDatum: WGS84; **Identification:** identifiedBy: Tommaso Fusco; dateIdentified: 2022; **Event:** samplingProtocol: Pitfall traps; eventDate: 2013-11-04; **Record Level:** collectionID: Roma3_5.8

##### Distribution

Southern Europe. S-European (SEU) chorotype.

#### Ozyptila praticola

(C. L. Koch, 1837)

13B8AF49-EE0F-56DB-B667-8F72432DBD0D

##### Materials

- **Type status:** Other material. **Occurrence:** recordedBy: Fattorini S., Di Giulio A.; individualCount: 2; sex: male; lifeStage: adult; occurrenceID: 646D294E-E823-523E-827A-D7648ED01B66; **Taxon:** scientificName: Ozyptilapraticola (C. L. Koch, 1837); order: Araneae; family: Thomisidae; genus: Ozyptila; **Location:** country: Italy; countryCode: IT; stateProvince: Rome; county: Rome; municipality: Rome; locality: Appia Antica Regional Park, Rome; locationRemarks: Caffarella Nord; decimalLatitude: 41.867753; decimalLongitude: 12.512414; geodeticDatum: WGS84; **Identification:** identifiedBy: Tommaso Fusco; dateIdentified: 2022; **Event:** samplingProtocol: Pitfall traps; eventDate: 2014-06-06; **Record Level:** collectionID: Roma3_5.8
- **Type status:** Other material. **Occurrence:** recordedBy: Fattorini S., Di Giulio A.; individualCount: 1; sex: male; lifeStage: adult; occurrenceID: A9ABFF3D-D5D5-5E93-8195-DB8349E39375; **Taxon:** scientificName: Ozyptilapraticola (C. L. Koch, 1837); order: Araneae; family: Thomisidae; genus: Ozyptila; **Location:** country: Italy; countryCode: IT; stateProvince: Rome; county: Rome; municipality: Rome; locality: Appia Antica Regional Park, Rome; locationRemarks: Acqua Santa; decimalLatitude: 41.850561; decimalLongitude: 12.530861; geodeticDatum: WGS84; **Identification:** identifiedBy: Tommaso Fusco; dateIdentified: 2022; **Event:** samplingProtocol: Pitfall traps; eventDate: 2014-06-19; **Record Level:** collectionID: Roma3_5.8
- **Type status:** Other material. **Occurrence:** recordedBy: Fattorini S., Di Giulio A.; individualCount: 1; sex: male; lifeStage: adult; occurrenceID: 8DEEB875-30E6-5015-8665-677C663C4D3D; **Taxon:** scientificName: Ozyptilapraticola (C. L. Koch, 1837); order: Araneae; family: Thomisidae; genus: Ozyptila; **Location:** country: Italy; countryCode: IT; stateProvince: Rome; county: Rome; municipality: Rome; locality: Appia Antica Regional Park, Rome; locationRemarks: Caffarella Nord; decimalLatitude: 41.867753; decimalLongitude: 12.512414; geodeticDatum: WGS84; **Identification:** identifiedBy: Tommaso Fusco; dateIdentified: 2022; **Event:** samplingProtocol: Pitfall traps; eventDate: 2014-05-27; **Record Level:** collectionID: Roma3_5.8
- **Type status:** Other material. **Occurrence:** recordedBy: Fattorini S., Di Giulio A.; individualCount: 2; sex: 1 male, 1 female; lifeStage: adult; occurrenceID: 365E6450-0329-57CA-A05F-4381DF515390; **Taxon:** scientificName: Ozyptilapraticola (C. L. Koch, 1837); order: Araneae; family: Thomisidae; genus: Ozyptila; **Location:** country: Italy; countryCode: IT; stateProvince: Rome; county: Rome; municipality: Rome; locality: Appia Antica Regional Park, Rome; locationRemarks: Caffarella Centro; decimalLatitude: 41.864889; decimalLongitude: 12.516389; geodeticDatum: WGS84; **Identification:** identifiedBy: Tommaso Fusco; dateIdentified: 2022; **Event:** samplingProtocol: Pitfall traps; eventDate: 2014-06-18; **Record Level:** collectionID: Roma3_5.8

##### Distribution

Europe, Turkey, Caucasus, Russia (Europe to south Siberia), Kazakhstan, Iran, Central Asia. Introduced to Canada, USA, Argentina. Asiatic-European (ASE) chorotype.

#### Ozyptila sanctuaria

(O. Pickard-Cambridge, 1871)

73E168E2-CE6F-56D8-AE2C-AA172F2EFE48

##### Materials

- **Type status:** Other material. **Occurrence:** recordedBy: Fattorini S., Di Giulio A.; individualCount: 1; sex: male; lifeStage: adult; occurrenceID: 2DF800DA-FCD0-5BFE-927A-BBAD2091C713; **Taxon:** scientificName: Ozyptilasanctuaria (O. Pickard-Cambridge, 1871); order: Araneae; family: Thomisidae; genus: Ozyptila; **Location:** country: Italy; countryCode: IT; stateProvince: Rome; county: Rome; municipality: Rome; locality: Appia Antica Regional Park, Rome; locationRemarks: Acqua Santa; decimalLatitude: 41.850561; decimalLongitude: 12.530861; geodeticDatum: WGS84; **Identification:** identifiedBy: Tommaso Fusco; dateIdentified: 2022; **Event:** samplingProtocol: Pitfall traps; eventDate: 2013-11-04; **Record Level:** collectionID: Roma3_5.8
- **Type status:** Other material. **Occurrence:** recordedBy: Fattorini S., Di Giulio A.; individualCount: 1; sex: male; lifeStage: adult; occurrenceID: BBB50667-6D45-5616-970E-2A9E4D2A0393; **Taxon:** scientificName: Ozyptilasanctuaria (O. Pickard-Cambridge, 1871); order: Araneae; family: Thomisidae; genus: Ozyptila; **Location:** country: Italy; countryCode: IT; stateProvince: Rome; county: Rome; municipality: Rome; locality: Appia Antica Regional Park, Rome; locationRemarks: Appia Antica; decimalLatitude: 41.812575; decimalLongitude: 12.564011; geodeticDatum: WGS84; **Identification:** identifiedBy: Tommaso Fusco; dateIdentified: 2022; **Event:** samplingProtocol: Pitfall traps; eventDate: 2013-11-27; **Record Level:** collectionID: Roma3_5.8
- **Type status:** Other material. **Occurrence:** recordedBy: Fattorini S., Di Giulio A.; individualCount: 1; sex: female; lifeStage: adult; occurrenceID: 3CBD9D57-DA26-502F-BCD9-05510C99E25D; **Taxon:** scientificName: Ozyptilasanctuaria (O. Pickard-Cambridge, 1871); order: Araneae; family: Thomisidae; genus: Ozyptila; **Location:** country: Italy; countryCode: IT; stateProvince: Rome; county: Rome; municipality: Rome; locality: Appia Antica Regional Park, Rome; locationRemarks: Appia Antica; decimalLatitude: 41.812575; decimalLongitude: 12.564011; geodeticDatum: WGS84; **Identification:** identifiedBy: Tommaso Fusco; dateIdentified: 2022; **Event:** samplingProtocol: Pitfall traps; eventDate: 2014-06-13; **Record Level:** collectionID: Roma3_5.8
- **Type status:** Other material. **Occurrence:** recordedBy: Fattorini S., Di Giulio A.; individualCount: 1; sex: female; lifeStage: adult; occurrenceID: BB1DDB76-C655-5978-8571-518D410BD61F; **Taxon:** scientificName: Ozyptilasanctuaria (O. Pickard-Cambridge, 1871); order: Araneae; family: Thomisidae; genus: Ozyptila; **Location:** country: Italy; countryCode: IT; stateProvince: Rome; county: Rome; municipality: Rome; locality: Appia Antica Regional Park, Rome; locationRemarks: Caffarella Sud 3; decimalLatitude: 41.856928; decimalLongitude: 12.528406; geodeticDatum: WGS84; **Identification:** identifiedBy: Tommaso Fusco; dateIdentified: 2022; **Event:** samplingProtocol: Pitfall traps; eventDate: 2014-05-19; **Record Level:** collectionID: Roma3_5.8
- **Type status:** Other material. **Occurrence:** recordedBy: Fattorini S., Di Giulio A.; individualCount: 1; sex: male; lifeStage: adult; occurrenceID: 6AD94711-7BE8-59C2-978E-0A3AAC0CFD8C; **Taxon:** scientificName: Ozyptilasanctuaria (O. Pickard-Cambridge, 1871); order: Araneae; family: Thomisidae; genus: Ozyptila; **Location:** country: Italy; countryCode: IT; stateProvince: Rome; county: Rome; municipality: Rome; locality: Appia Antica Regional Park, Rome; locationRemarks: Cava Fiorucci; decimalLatitude: 41.834106; decimalLongitude: 12.549264; geodeticDatum: WGS84; **Identification:** identifiedBy: Tommaso Fusco; dateIdentified: 2022; **Event:** samplingProtocol: Pitfall traps; eventDate: 2013-11-07; **Record Level:** collectionID: Roma3_5.8
- **Type status:** Other material. **Occurrence:** recordedBy: Fattorini S., Di Giulio A.; individualCount: 1; sex: male; lifeStage: adult; occurrenceID: A556940A-9EA2-547C-84FB-5F142A613324; **Taxon:** scientificName: Ozyptilasanctuaria (O. Pickard-Cambridge, 1871); order: Araneae; family: Thomisidae; genus: Ozyptila; **Location:** country: Italy; countryCode: IT; stateProvince: Rome; county: Rome; municipality: Rome; locality: Appia Antica Regional Park, Rome; locationRemarks: Farnesiana; decimalLatitude: 41.839667; decimalLongitude: 12.525528; geodeticDatum: WGS84; **Identification:** identifiedBy: Tommaso Fusco; dateIdentified: 2022; **Event:** samplingProtocol: Pitfall traps; eventDate: 2013-11-13; **Record Level:** collectionID: Roma3_5.8
- **Type status:** Other material. **Occurrence:** recordedBy: Fattorini S., Di Giulio A.; individualCount: 1; sex: female; lifeStage: adult; occurrenceID: 137671DF-19A6-535D-A89E-2DD3C15A4375; **Taxon:** scientificName: Ozyptilasanctuaria (O. Pickard-Cambridge, 1871); order: Araneae; family: Thomisidae; genus: Ozyptila; **Location:** country: Italy; countryCode: IT; stateProvince: Rome; county: Rome; municipality: Rome; locality: Appia Antica Regional Park, Rome; locationRemarks: Farnesiana; decimalLatitude: 41.839667; decimalLongitude: 12.525528; geodeticDatum: WGS84; **Identification:** identifiedBy: Tommaso Fusco; dateIdentified: 2022; **Event:** samplingProtocol: Pitfall traps; eventDate: 2014-05-28; **Record Level:** collectionID: Roma3_5.8
- **Type status:** Other material. **Occurrence:** recordedBy: Fattorini S., Di Giulio A.; individualCount: 1; sex: male; lifeStage: adult; occurrenceID: FA4872C9-5B6A-5EB9-AF99-0904C51258E3; **Taxon:** scientificName: Ozyptilasanctuaria (O. Pickard-Cambridge, 1871); order: Araneae; family: Thomisidae; genus: Ozyptila; **Location:** country: Italy; countryCode: IT; stateProvince: Rome; county: Rome; municipality: Rome; locality: Appia Antica Regional Park, Rome; locationRemarks: Farnesiana; decimalLatitude: 41.839667; decimalLongitude: 12.525528; geodeticDatum: WGS84; **Identification:** identifiedBy: Tommaso Fusco; dateIdentified: 2022; **Event:** samplingProtocol: Pitfall traps; eventDate: 2013-11-04; **Record Level:** collectionID: Roma3_5.8
- **Type status:** Other material. **Occurrence:** recordedBy: Fattorini S., Di Giulio A.; individualCount: 1; sex: female; lifeStage: adult; occurrenceID: A705F84C-E5AC-5D1E-ACEC-29981664C817; **Taxon:** scientificName: Ozyptilasanctuaria (O. Pickard-Cambridge, 1871); order: Araneae; family: Thomisidae; genus: Ozyptila; **Location:** country: Italy; countryCode: IT; stateProvince: Rome; county: Rome; municipality: Rome; locality: Appia Antica Regional Park, Rome; locationRemarks: Torre selce; decimalLatitude: 41.816611; decimalLongitude: 12.560667; geodeticDatum: WGS84; **Identification:** identifiedBy: Tommaso Fusco; dateIdentified: 2022; **Event:** samplingProtocol: Pitfall traps; eventDate: 2014-05-26; **Record Level:** collectionID: Roma3_5.8

##### Distribution

Most of Europe. European (EUR) chorotype.

#### Xysticus kochi

Thorell, 1872

95412DE4-842E-5B97-AC6A-CEF72253626D

##### Materials

- **Type status:** Other material. **Occurrence:** recordedBy: Fattorini S., Di Giulio A.; individualCount: 1; sex: female; lifeStage: adult; occurrenceID: B7A9113F-9F08-53BD-A9D9-7E113A2E49B3; **Taxon:** scientificName: Xysticuskochi Thorell, 1872; order: Araneae; family: Thomisidae; genus: Xysticus; **Location:** country: Italy; countryCode: IT; stateProvince: Rome; county: Rome; municipality: Rome; locality: Appia Antica Regional Park, Rome; locationRemarks: Farnesiana; decimalLatitude: 41.839667; decimalLongitude: 12.525528; geodeticDatum: WGS84; **Identification:** identifiedBy: Tommaso Fusco; dateIdentified: 2022; **Event:** samplingProtocol: Pitfall traps; eventDate: 2014-06-09; **Record Level:** collectionID: Roma3_5.8

##### Distribution

Europe to Central Asia. Sibero-European (SIE) chorotype.

#### Titanoecidae

Lehtinen, 1967

339325D2-A302-57C1-BF46-E7E51979BEEC

#### Titanoeca flavicoma

L. Koch, 1872

72B294C5-2D4B-5A72-9F81-1A6A8A5DD68B

##### Materials

- **Type status:** Other material. **Occurrence:** recordedBy: Fattorini S., Di Giulio A.; individualCount: 2; sex: male; lifeStage: adult; occurrenceID: CA6E514B-0748-5898-A937-D2872945C985; **Taxon:** scientificName: Titanoecaflavicoma L. Koch, 1872; order: Araneae; family: Titanoecidae; genus: Titanoeca; **Location:** country: Italy; countryCode: IT; stateProvince: Rome; county: Rome; municipality: Rome; locality: Appia Antica Regional Park, Rome; locationRemarks: Cava Fiorucci; decimalLatitude: 41.834106; decimalLongitude: 12.549264; geodeticDatum: WGS84; **Identification:** identifiedBy: Tommaso Fusco; dateIdentified: 2022; **Event:** samplingProtocol: Pitfall traps; eventDate: 2014-06-05; **Record Level:** collectionID: Roma3_5.8
- **Type status:** Other material. **Occurrence:** recordedBy: Fattorini S., Di Giulio A.; individualCount: 1; sex: male; lifeStage: adult; occurrenceID: 041D896A-E9CC-55E2-A57B-4FAE248B3A38; **Taxon:** scientificName: Titanoecaflavicoma L. Koch, 1872; order: Araneae; family: Titanoecidae; genus: Titanoeca; **Location:** country: Italy; countryCode: IT; stateProvince: Rome; county: Rome; municipality: Rome; locality: Appia Antica Regional Park, Rome; locationRemarks: Farnesiana; decimalLatitude: 41.839667; decimalLongitude: 12.525528; geodeticDatum: WGS84; **Identification:** identifiedBy: Tommaso Fusco; dateIdentified: 2022; **Event:** samplingProtocol: Pitfall traps; eventDate: 2014-05-28; **Record Level:** collectionID: Roma3_5.8
- **Type status:** Other material. **Occurrence:** recordedBy: Fattorini S., Di Giulio A.; individualCount: 6; sex: male; lifeStage: adult; occurrenceID: 4BB778F0-A788-563A-A7D7-F81DB23C9038; **Taxon:** scientificName: Titanoecaflavicoma L. Koch, 1872; order: Araneae; family: Titanoecidae; genus: Titanoeca; **Location:** country: Italy; countryCode: IT; stateProvince: Rome; county: Rome; municipality: Rome; locality: Appia Antica Regional Park, Rome; locationRemarks: Farnesiana; decimalLatitude: 41.839667; decimalLongitude: 12.525528; geodeticDatum: WGS84; **Identification:** identifiedBy: Tommaso Fusco; dateIdentified: 2022; **Event:** samplingProtocol: Pitfall traps; eventDate: 2014-06-09; **Record Level:** collectionID: Roma3_5.8
- **Type status:** Other material. **Occurrence:** recordedBy: Fattorini S., Di Giulio A.; individualCount: 1; sex: female; lifeStage: adult; occurrenceID: 57DF4416-D12A-585E-982C-8B6BC068F492; **Taxon:** scientificName: Titanoecaflavicoma L. Koch, 1872; order: Araneae; family: Titanoecidae; genus: Titanoeca; **Location:** country: Italy; countryCode: IT; stateProvince: Rome; county: Rome; municipality: Rome; locality: Appia Antica Regional Park, Rome; locationRemarks: Farnesiana; decimalLatitude: 41.839667; decimalLongitude: 12.525528; geodeticDatum: WGS84; **Identification:** identifiedBy: Tommaso Fusco; dateIdentified: 2022; **Event:** samplingProtocol: Pitfall traps; eventDate: 2014-06-19; **Record Level:** collectionID: Roma3_5.8
- **Type status:** Other material. **Occurrence:** recordedBy: Fattorini S., Di Giulio A.; individualCount: 2; sex: 1 male, 1 female; lifeStage: adult; occurrenceID: CA6F2969-6766-582B-A980-DB3D01D5589D; **Taxon:** scientificName: Titanoecaflavicoma L. Koch, 1872; order: Araneae; family: Titanoecidae; genus: Titanoeca; **Location:** country: Italy; countryCode: IT; stateProvince: Rome; county: Rome; municipality: Rome; locality: Appia Antica Regional Park, Rome; locationRemarks: Torre Selce; decimalLatitude: 41.816611; decimalLongitude: 12.560667; geodeticDatum: WGS84; **Identification:** identifiedBy: Tommaso Fusco; dateIdentified: 2022; **Event:** samplingProtocol: Pitfall traps; eventDate: 2014-06-05; **Record Level:** collectionID: Roma3_5.8
- **Type status:** Other material. **Occurrence:** recordedBy: Fattorini S., Di Giulio A.; individualCount: 1; sex: male; lifeStage: adult; occurrenceID: 88D7D113-1074-53A8-8FCA-48539F0550E5; **Taxon:** scientificName: Titanoecaflavicoma L. Koch, 1872; order: Araneae; family: Titanoecidae; genus: Titanoeca; **Location:** country: Italy; countryCode: IT; stateProvince: Rome; county: Rome; municipality: Rome; locality: Appia Antica Regional Park, Rome; locationRemarks: Torre Selce; decimalLatitude: 41.816611; decimalLongitude: 12.560667; geodeticDatum: WGS84; **Identification:** identifiedBy: Tommaso Fusco; dateIdentified: 2022; **Event:** samplingProtocol: Pitfall traps; eventDate: 2014-06-13; **Record Level:** collectionID: Roma3_5.8

##### Distribution

France (Corsica), Italy, Balkans, Israel. Mediterranean (MED) chorotype.

#### Zodariidae

Thorell, 1881

6A3D2D38-1E5C-5838-A98C-232A76C72AC1

#### Zodarion elegans

(Simon, 1873)

670CC587-76D0-5DE5-8A84-0FDF26129B34

##### Materials

- **Type status:** Other material. **Occurrence:** recordedBy: Fattorini S., Di Giulio A.; individualCount: 1; sex: male; lifeStage: adult; occurrenceID: 945343A6-7A32-50D1-8F0F-9609FE3B5335; **Taxon:** scientificName: Zodarionelegans (Simon, 1873); order: Araneae; family: Zodariidae; genus: Zodarion; **Location:** country: Italy; countryCode: IT; stateProvince: Rome; county: Rome; municipality: Rome; locality: Appia Antica Regional Park, Rome; locationRemarks: Casal Verbeni; decimalLatitude: 41.815250; decimalLongitude: 12.552222; geodeticDatum: WGS84; **Identification:** identifiedBy: Tommaso Fusco; dateIdentified: 2022; **Event:** samplingProtocol: Pitfall traps; eventDate: 2014-05-26; **Record Level:** collectionID: Roma3_5.8
- **Type status:** Other material. **Occurrence:** recordedBy: Fattorini S., Di Giulio A.; individualCount: 1; sex: male; lifeStage: adult; occurrenceID: 8A1E5344-AF79-50DC-9E3F-9D191DEA7A80; **Taxon:** scientificName: Zodarionelegans (Simon, 1873); order: Araneae; family: Zodariidae; genus: Zodarion; **Location:** country: Italy; countryCode: IT; stateProvince: Rome; county: Rome; municipality: Rome; locality: Appia Antica Regional Park, Rome; locationRemarks: Cava Fiorucci; decimalLatitude: 41.834106; decimalLongitude: 12.549264; geodeticDatum: WGS84; **Identification:** identifiedBy: Tommaso Fusco; dateIdentified: 2022; **Event:** samplingProtocol: Pitfall traps; eventDate: 2014-06-13; **Record Level:** collectionID: Roma3_5.8
- **Type status:** Other material. **Occurrence:** recordedBy: Fattorini S., Di Giulio A.; individualCount: 2; sex: male; lifeStage: adult; occurrenceID: 85F2F058-6D47-50CC-A9E7-81BCBEE2B15A; **Taxon:** scientificName: Zodarionelegans (Simon, 1873); order: Araneae; family: Zodariidae; genus: Zodarion; **Location:** country: Italy; countryCode: IT; stateProvince: Rome; county: Rome; municipality: Rome; locality: Appia Antica Regional Park, Rome; locationRemarks: Farnesiana; decimalLatitude: 41.839667; decimalLongitude: 12.525528; geodeticDatum: WGS84; **Identification:** identifiedBy: Tommaso Fusco; dateIdentified: 2022; **Event:** samplingProtocol: Pitfall traps; eventDate: 2014-05-26; **Record Level:** collectionID: Roma3_5.8
- **Type status:** Other material. **Occurrence:** recordedBy: Fattorini S., Di Giulio A.; individualCount: 1; sex: male; lifeStage: adult; occurrenceID: 76C025E4-AEEB-5051-8616-41E01ADF962E; **Taxon:** scientificName: Zodarionelegans (Simon, 1873); order: Araneae; family: Zodariidae; genus: Zodarion; **Location:** country: Italy; countryCode: IT; stateProvince: Rome; county: Rome; municipality: Rome; locality: Appia Antica Regional Park, Rome; locationRemarks: Farnesiana; decimalLatitude: 41.839667; decimalLongitude: 12.525528; geodeticDatum: WGS84; **Identification:** identifiedBy: Tommaso Fusco; dateIdentified: 2022; **Event:** samplingProtocol: Pitfall traps; eventDate: 2014-05-28; **Record Level:** collectionID: Roma3_5.8
- **Type status:** Other material. **Occurrence:** recordedBy: Fattorini S., Di Giulio A.; individualCount: 3; sex: male; lifeStage: adult; occurrenceID: EB96A6C8-55B7-5B69-A88B-9FA809613A50; **Taxon:** scientificName: Zodarionelegans (Simon, 1873); order: Araneae; family: Zodariidae; genus: Zodarion; **Location:** country: Italy; countryCode: IT; stateProvince: Rome; county: Rome; municipality: Rome; locality: Appia Antica Regional Park, Rome; locationRemarks: Farnesiana; decimalLatitude: 41.839667; decimalLongitude: 12.525528; geodeticDatum: WGS84; **Identification:** identifiedBy: Tommaso Fusco; dateIdentified: 2022; **Event:** samplingProtocol: Pitfall traps; eventDate: 2014-06-09; **Record Level:** collectionID: Roma3_5.8
- **Type status:** Other material. **Occurrence:** recordedBy: Fattorini S., Di Giulio A.; individualCount: 26; sex: 22 male, 4 female; lifeStage: adult; occurrenceID: 5C959700-E6B4-59D3-88DA-DABE7BD25D6E; **Taxon:** scientificName: Zodarionelegans (Simon, 1873); order: Araneae; family: Zodariidae; genus: Zodarion; **Location:** country: Italy; countryCode: IT; stateProvince: Rome; county: Rome; municipality: Rome; locality: Appia Antica Regional Park, Rome; locationRemarks: Farnesiana; decimalLatitude: 41.839667; decimalLongitude: 12.525528; geodeticDatum: WGS84; **Identification:** identifiedBy: Tommaso Fusco; dateIdentified: 2022; **Event:** samplingProtocol: Pitfall traps; eventDate: 2014-06-19; **Record Level:** collectionID: Roma3_5.8
- **Type status:** Other material. **Occurrence:** recordedBy: Fattorini S., Di Giulio A.; individualCount: 1; sex: male; lifeStage: adult; occurrenceID: 30DF923C-3298-530E-AE3B-E187C5B902E4; **Taxon:** scientificName: Zodarionelegans (Simon, 1873); order: Araneae; family: Zodariidae; genus: Zodarion; **Location:** country: Italy; countryCode: IT; stateProvince: Rome; county: Rome; municipality: Rome; locality: Appia Antica Regional Park, Rome; locationRemarks: Tor Marancia; decimalLatitude: 41.850308; decimalLongitude: 12.503178; geodeticDatum: WGS84; **Identification:** identifiedBy: Tommaso Fusco; dateIdentified: 2022; **Event:** samplingProtocol: Pitfall traps; eventDate: 2013-10-28; **Record Level:** collectionID: Roma3_5.8
- **Type status:** Other material. **Occurrence:** recordedBy: Fattorini S., Di Giulio A.; individualCount: 1; sex: male; lifeStage: adult; occurrenceID: 0DC5F636-130F-5C5A-B7CD-4F7F83E41C1D; **Taxon:** scientificName: Zodarionelegans (Simon, 1873); order: Araneae; family: Zodariidae; genus: Zodarion; **Location:** country: Italy; countryCode: IT; stateProvince: Rome; county: Rome; municipality: Rome; locality: Appia Antica Regional Park, Rome; locationRemarks: Torre Selce; decimalLatitude: 41.816611; decimalLongitude: 12.560667; geodeticDatum: WGS84; **Identification:** identifiedBy: Tommaso Fusco; dateIdentified: 2022; **Event:** samplingProtocol: Pitfall traps; eventDate: 2014-05-26; **Record Level:** collectionID: Roma3_5.8
- **Type status:** Other material. **Occurrence:** recordedBy: Fattorini S., Di Giulio A.; individualCount: 8; sex: male; lifeStage: adult; occurrenceID: D658AA39-CA9E-5CDE-967B-C2B08DA644CE; **Taxon:** scientificName: Zodarionelegans (Simon, 1873); order: Araneae; family: Zodariidae; genus: Zodarion; **Location:** country: Italy; countryCode: IT; stateProvince: Rome; county: Rome; municipality: Rome; locality: Appia Antica Regional Park, Rome; locationRemarks: Torre Selce; decimalLatitude: 41.816611; decimalLongitude: 12.560667; geodeticDatum: WGS84; **Identification:** identifiedBy: Tommaso Fusco; dateIdentified: 2022; **Event:** samplingProtocol: Pitfall traps; eventDate: 2014-06-05; **Record Level:** collectionID: Roma3_5.8
- **Type status:** Other material. **Occurrence:** recordedBy: Fattorini S., Di Giulio A.; individualCount: 2; sex: male; lifeStage: adult; occurrenceID: 05027552-B64A-5434-A051-CA977E4D5F8F; **Taxon:** scientificName: Zodarionelegans (Simon, 1873); order: Araneae; family: Zodariidae; genus: Zodarion; **Location:** country: Italy; countryCode: IT; stateProvince: Rome; county: Rome; municipality: Rome; locality: Appia Antica Regional Park, Rome; locationRemarks: Torre Selce; decimalLatitude: 41.816611; decimalLongitude: 12.560667; geodeticDatum: WGS84; **Identification:** identifiedBy: Tommaso Fusco; dateIdentified: 2022; **Event:** samplingProtocol: Pitfall traps; eventDate: 2014-06-13; **Record Level:** collectionID: Roma3_5.8
- **Type status:** Other material. **Occurrence:** recordedBy: Fattorini S., Di Giulio A.; individualCount: 1; sex: male; lifeStage: adult; occurrenceID: 6D141141-4D8F-590B-921A-E0DEED4A894D; **Taxon:** scientificName: Zodarionelegans (Simon, 1873); order: Araneae; family: Zodariidae; genus: Zodarion; **Location:** country: Italy; countryCode: IT; stateProvince: Rome; county: Rome; municipality: Rome; locality: Appia Antica Regional Park, Rome; locationRemarks: Torre Selce; decimalLatitude: 41.816611; decimalLongitude: 12.560667; geodeticDatum: WGS84; **Identification:** identifiedBy: Tommaso Fusco; dateIdentified: 2022; **Event:** samplingProtocol: Pitfall traps; eventDate: 2014-06-24; **Record Level:** collectionID: Roma3_5.8

##### Distribution

Southern Europe, North Africa. Mediterranean (MED) chorotype.

#### Zodarion italicum

(Canestrini, 1868)

16984B4B-BF0E-5DB6-AEAB-9F04F80F6A19

##### Materials

- **Type status:** Other material. **Occurrence:** recordedBy: Fattorini S., Di Giulio A.; individualCount: 57; sex: 33 male, 24 female; lifeStage: adult; occurrenceID: D7E7525D-AF15-56FF-969B-D38D27D72BA9; **Taxon:** scientificName: Zodarionitalicum (Canestrini, 1868); order: Araneae; family: Zodariidae; genus: Zodarion; **Location:** country: Italy; countryCode: IT; stateProvince: Rome; county: Rome; municipality: Rome; locality: Appia Antica Regional Park, Rome; locationRemarks: Acqua Santa; decimalLatitude: 41.850561; decimalLongitude: 12.530861; geodeticDatum: WGS84; **Identification:** identifiedBy: Tommaso Fusco; dateIdentified: 2022; **Event:** samplingProtocol: Pitfall traps; eventDate: 2014-05-20; **Record Level:** collectionID: Roma3_5.8
- **Type status:** Other material. **Occurrence:** recordedBy: Fattorini S., Di Giulio A.; individualCount: 5; sex: male; lifeStage: adult; occurrenceID: 4AAC01DE-0203-5EAD-9B8A-689EC06F0FD1; **Taxon:** scientificName: Zodarionitalicum (Canestrini, 1868); order: Araneae; family: Zodariidae; genus: Zodarion; **Location:** stateProvince: Rome; county: Rome; municipality: Rome; locality: Appia Antica Regional Park, Rome; locationRemarks: Acqua Santa; decimalLatitude: 41.850561; decimalLongitude: 12.530861; geodeticDatum: WGS84; **Identification:** identifiedBy: Tommaso Fusco; dateIdentified: 2022; **Event:** samplingProtocol: Pitfall traps; eventDate: 2014-05-28; **Record Level:** collectionID: Roma3_5.8
- **Type status:** Other material. **Occurrence:** recordedBy: Fattorini S., Di Giulio A.; individualCount: 18; sex: 17 male, 1 female; lifeStage: adult; occurrenceID: A7DAE3AF-78E9-5A51-B5B3-010DB1BC6DBE; **Taxon:** scientificName: Zodarionitalicum (Canestrini, 1868); order: Araneae; family: Zodariidae; genus: Zodarion; **Location:** stateProvince: Rome; county: Rome; municipality: Rome; locality: Appia Antica Regional Park, Rome; locationRemarks: Acqua Santa; decimalLatitude: 41.850561; decimalLongitude: 12.530861; geodeticDatum: WGS84; **Identification:** identifiedBy: Tommaso Fusco; dateIdentified: 2022; **Event:** samplingProtocol: Pitfall traps; eventDate: 2014-06-09; **Record Level:** collectionID: Roma3_5.8
- **Type status:** Other material. **Occurrence:** recordedBy: Fattorini S., Di Giulio A.; individualCount: 20; sex: 13 male, 7 female; lifeStage: adult; occurrenceID: 4820F5D3-0914-533C-A673-6E55AEA84038; **Taxon:** scientificName: Zodarionitalicum (Canestrini, 1868); order: Araneae; family: Zodariidae; genus: Zodarion; **Location:** stateProvince: Rome; county: Rome; municipality: Rome; locality: Appia Antica Regional Park, Rome; locationRemarks: Acqua Santa; decimalLatitude: 41.850561; decimalLongitude: 12.530861; geodeticDatum: WGS84; **Identification:** identifiedBy: Tommaso Fusco; dateIdentified: 2022; **Event:** samplingProtocol: Pitfall traps; eventDate: 2014-06-19; **Record Level:** collectionID: Roma3_5.8
- **Type status:** Other material. **Occurrence:** recordedBy: Fattorini S., Di Giulio A.; individualCount: 2; sex: male; lifeStage: adult; occurrenceID: 1906987B-C07D-517C-9145-1C741991CEF1; **Taxon:** scientificName: Zodarionitalicum (Canestrini, 1868); order: Araneae; family: Zodariidae; genus: Zodarion; **Location:** stateProvince: Rome; county: Rome; municipality: Rome; locality: Appia Antica Regional Park, Rome; locationRemarks: Caffarella Centro; decimalLatitude: 41.864889; decimalLongitude: 12.516389; geodeticDatum: WGS84; **Identification:** identifiedBy: Tommaso Fusco; dateIdentified: 2022; **Event:** samplingProtocol: Pitfall traps; eventDate: 2014-05-19; **Record Level:** collectionID: Roma3_5.8
- **Type status:** Other material. **Occurrence:** recordedBy: Fattorini S., Di Giulio A.; individualCount: 1; sex: female; lifeStage: adult; occurrenceID: 020287C6-1D90-5128-A7C2-EB47FEAFE354; **Taxon:** scientificName: Zodarionitalicum (Canestrini, 1868); order: Araneae; family: Zodariidae; genus: Zodarion; **Location:** stateProvince: Rome; county: Rome; municipality: Rome; locality: Appia Antica Regional Park, Rome; locationRemarks: Caffarella Centro; decimalLatitude: 41.864889; decimalLongitude: 12.516389; geodeticDatum: WGS84; **Identification:** identifiedBy: Tommaso Fusco; dateIdentified: 2022; **Event:** samplingProtocol: Pitfall traps; eventDate: 2014-05-27; **Record Level:** collectionID: Roma3_5.8
- **Type status:** Other material. **Occurrence:** recordedBy: Fattorini S., Di Giulio A.; individualCount: 1; sex: male; lifeStage: adult; occurrenceID: 1A263398-B8F2-5925-801F-9A96173ED4B8; **Taxon:** scientificName: Zodarionitalicum (Canestrini, 1868); order: Araneae; family: Zodariidae; genus: Zodarion; **Location:** stateProvince: Rome; county: Rome; municipality: Rome; locality: Appia Antica Regional Park, Rome; locationRemarks: Caffarella Centro; decimalLatitude: 41.864889; decimalLongitude: 12.516389; geodeticDatum: WGS84; **Identification:** identifiedBy: Tommaso Fusco; dateIdentified: 2022; **Event:** samplingProtocol: Pitfall traps; eventDate: 2014-06-06; **Record Level:** collectionID: Roma3_5.8
- **Type status:** Other material. **Occurrence:** recordedBy: Fattorini S., Di Giulio A.; individualCount: 9; sex: 5 male, 4 female; lifeStage: adult; occurrenceID: 629CBE3E-C05C-585E-9027-F4EACFFF2D02; **Taxon:** scientificName: Zodarionitalicum (Canestrini, 1868); order: Araneae; family: Zodariidae; genus: Zodarion; **Location:** stateProvince: Rome; county: Rome; municipality: Rome; locality: Appia Antica Regional Park, Rome; locationRemarks: Caffarella Nord; decimalLatitude: 41.867753; decimalLongitude: 12.512414; geodeticDatum: WGS84; **Identification:** identifiedBy: Tommaso Fusco; dateIdentified: 2022; **Event:** samplingProtocol: Pitfall traps; eventDate: 2014-05-19; **Record Level:** collectionID: Roma3_5.8
- **Type status:** Other material. **Occurrence:** recordedBy: Fattorini S., Di Giulio A.; individualCount: 3; sex: 1 male, 2 female; lifeStage: adult; occurrenceID: 971ADA58-6EE4-5EEA-B339-1B20EC53E03F; **Taxon:** scientificName: Zodarionitalicum (Canestrini, 1868); order: Araneae; family: Zodariidae; genus: Zodarion; **Location:** stateProvince: Rome; county: Rome; municipality: Rome; locality: Appia Antica Regional Park, Rome; locationRemarks: Caffarella Nord; decimalLatitude: 41.867753; decimalLongitude: 12.512414; geodeticDatum: WGS84; **Identification:** identifiedBy: Tommaso Fusco; dateIdentified: 2022; **Event:** samplingProtocol: Pitfall traps; eventDate: 2014-05-27; **Record Level:** collectionID: Roma3_5.8
- **Type status:** Other material. **Occurrence:** recordedBy: Fattorini S., Di Giulio A.; individualCount: 7; sex: male; lifeStage: adult; occurrenceID: DDC9C971-A835-52CA-9569-4229A59A9C4D; **Taxon:** scientificName: Zodarionitalicum (Canestrini, 1868); order: Araneae; family: Zodariidae; genus: Zodarion; **Location:** stateProvince: Rome; county: Rome; municipality: Rome; locality: Appia Antica Regional Park, Rome; locationRemarks: Caffarella Nord; decimalLatitude: 41.867753; decimalLongitude: 12.512414; geodeticDatum: WGS84; **Identification:** identifiedBy: Tommaso Fusco; dateIdentified: 2022; **Event:** samplingProtocol: Pitfall traps; eventDate: 2014-06-06; **Record Level:** collectionID: Roma3_5.8
- **Type status:** Other material. **Occurrence:** recordedBy: Fattorini S., Di Giulio A.; individualCount: 1; sex: female; lifeStage: adult; occurrenceID: E76EBD00-0E92-5B96-8030-F71A1277271A; **Taxon:** scientificName: Zodarionitalicum (Canestrini, 1868); order: Araneae; family: Zodariidae; genus: Zodarion; **Location:** stateProvince: Rome; county: Rome; municipality: Rome; locality: Appia Antica Regional Park, Rome; locationRemarks: Caffarella Nord; decimalLatitude: 41.867753; decimalLongitude: 12.512414; geodeticDatum: WGS84; **Identification:** identifiedBy: Tommaso Fusco; dateIdentified: 2022; **Event:** samplingProtocol: Pitfall traps; eventDate: 2013-11-29; **Record Level:** collectionID: Roma3_5.8
- **Type status:** Other material. **Occurrence:** recordedBy: Fattorini S., Di Giulio A.; individualCount: 2; sex: male; lifeStage: adult; occurrenceID: C9DE85F2-360B-5109-8DCB-99D0135E75F5; **Taxon:** scientificName: Zodarionitalicum (Canestrini, 1868); order: Araneae; family: Zodariidae; genus: Zodarion; **Location:** stateProvince: Rome; county: Rome; municipality: Rome; locality: Appia Antica Regional Park, Rome; locationRemarks: Caffarella Nord; decimalLatitude: 41.867753; decimalLongitude: 12.512414; geodeticDatum: WGS84; **Identification:** identifiedBy: Tommaso Fusco; dateIdentified: 2022; **Event:** samplingProtocol: Pitfall traps; eventDate: 2014-06-18; **Record Level:** collectionID: Roma3_5.8
- **Type status:** Other material. **Occurrence:** recordedBy: Fattorini S., Di Giulio A.; individualCount: 1; sex: male; lifeStage: adult; occurrenceID: C6365FFE-4B2F-5D67-846C-7B503006BFA9; **Taxon:** scientificName: Zodarionitalicum (Canestrini, 1868); order: Araneae; family: Zodariidae; genus: Zodarion; **Location:** stateProvince: Rome; county: Rome; municipality: Rome; locality: Appia Antica Regional Park, Rome; locationRemarks: Caffarella Sud; decimalLatitude: 41.857247; decimalLongitude: 12.529211; geodeticDatum: WGS84; **Identification:** identifiedBy: Tommaso Fusco; dateIdentified: 2022; **Event:** samplingProtocol: Pitfall traps; eventDate: 2013-11-12; **Record Level:** collectionID: Roma3_5.8
- **Type status:** Other material. **Occurrence:** recordedBy: Fattorini S., Di Giulio A.; individualCount: 14; sex: 11 male, 3 female; lifeStage: adult; occurrenceID: 378EA686-115A-58A2-BD8E-6686F4175FD9; **Taxon:** scientificName: Zodarionitalicum (Canestrini, 1868); order: Araneae; family: Zodariidae; genus: Zodarion; **Location:** stateProvince: Rome; county: Rome; municipality: Rome; locality: Appia Antica Regional Park, Rome; locationRemarks: Caffarella Sud; decimalLatitude: 41.857247; decimalLongitude: 12.529211; geodeticDatum: WGS84; **Identification:** identifiedBy: Tommaso Fusco; dateIdentified: 2022; **Event:** samplingProtocol: Pitfall traps; eventDate: 2014-05-19; **Record Level:** collectionID: Roma3_5.8
- **Type status:** Other material. **Occurrence:** recordedBy: Fattorini S., Di Giulio A.; individualCount: 2; sex: male; lifeStage: adult; occurrenceID: E2BF1E69-81B3-58B5-BE33-BF68A94E75D2; **Taxon:** scientificName: Zodarionitalicum (Canestrini, 1868); order: Araneae; family: Zodariidae; genus: Zodarion; **Location:** stateProvince: Rome; county: Rome; municipality: Rome; locality: Appia Antica Regional Park, Rome; locationRemarks: Caffarella Sud; decimalLatitude: 41.857247; decimalLongitude: 12.529211; geodeticDatum: WGS84; **Identification:** identifiedBy: Tommaso Fusco; dateIdentified: 2022; **Event:** samplingProtocol: Pitfall traps; eventDate: 2014-05-27; **Record Level:** collectionID: Roma3_5.8
- **Type status:** Other material. **Occurrence:** recordedBy: Fattorini S., Di Giulio A.; individualCount: 2; sex: male; lifeStage: adult; occurrenceID: 494C1450-02BE-53A2-85A2-C703FE319944; **Taxon:** scientificName: Zodarionitalicum (Canestrini, 1868); order: Araneae; family: Zodariidae; genus: Zodarion; **Location:** stateProvince: Rome; county: Rome; municipality: Rome; locality: Appia Antica Regional Park, Rome; locationRemarks: Caffarella Sud; decimalLatitude: 41.857247; decimalLongitude: 12.529211; geodeticDatum: WGS84; **Identification:** identifiedBy: Tommaso Fusco; dateIdentified: 2022; **Event:** samplingProtocol: Pitfall traps; eventDate: 2014-06-06; **Record Level:** collectionID: Roma3_5.8
- **Type status:** Other material. **Occurrence:** recordedBy: Fattorini S., Di Giulio A.; individualCount: 4; sex: male; lifeStage: adult; occurrenceID: 242F6A5A-BFE7-5BC9-BCB1-DCDEEBDA4F96; **Taxon:** scientificName: Zodarionitalicum (Canestrini, 1868); order: Araneae; family: Zodariidae; genus: Zodarion; **Location:** stateProvince: Rome; county: Rome; municipality: Rome; locality: Appia Antica Regional Park, Rome; locationRemarks: Caffarella Sud; decimalLatitude: 41.857247; decimalLongitude: 12.529211; geodeticDatum: WGS84; **Identification:** identifiedBy: Tommaso Fusco; dateIdentified: 2022; **Event:** samplingProtocol: Pitfall traps; eventDate: 2014-06-18; **Record Level:** collectionID: Roma3_5.8
- **Type status:** Other material. **Occurrence:** recordedBy: Fattorini S., Di Giulio A.; individualCount: 8; sex: male; lifeStage: adult; occurrenceID: 4CAF275C-D07B-5FFA-9273-0EA5DDF135C9; **Taxon:** scientificName: Zodarionitalicum (Canestrini, 1868); order: Araneae; family: Zodariidae; genus: Zodarion; **Location:** stateProvince: Rome; county: Rome; municipality: Rome; locality: Appia Antica Regional Park, Rome; locationRemarks: Cava Fiorucci; decimalLatitude: 41.834106; decimalLongitude: 12.549264; geodeticDatum: WGS84; **Identification:** identifiedBy: Tommaso Fusco; dateIdentified: 2022; **Event:** samplingProtocol: Pitfall traps; eventDate: 2014-05-26; **Record Level:** collectionID: Roma3_5.8
- **Type status:** Other material. **Occurrence:** recordedBy: Fattorini S., Di Giulio A.; individualCount: 6; sex: male; lifeStage: adult; occurrenceID: 2B07401D-0031-5728-BA0B-3EF9F54498F0; **Taxon:** scientificName: Zodarionitalicum (Canestrini, 1868); order: Araneae; family: Zodariidae; genus: Zodarion; **Location:** stateProvince: Rome; county: Rome; municipality: Rome; locality: Appia Antica Regional Park, Rome; locationRemarks: Cava Fiorucci; decimalLatitude: 41.834106; decimalLongitude: 12.549264; geodeticDatum: WGS84; **Identification:** identifiedBy: Tommaso Fusco; dateIdentified: 2022; **Event:** samplingProtocol: Pitfall traps; eventDate: 2014-06-05; **Record Level:** collectionID: Roma3_5.8
- **Type status:** Other material. **Occurrence:** recordedBy: Fattorini S., Di Giulio A.; individualCount: 1; sex: male; lifeStage: adult; occurrenceID: 370C269C-67B2-504F-9B05-7CB008C0FA6C; **Taxon:** scientificName: Zodarionitalicum (Canestrini, 1868); order: Araneae; family: Zodariidae; genus: Zodarion; **Location:** stateProvince: Rome; county: Rome; municipality: Rome; locality: Appia Antica Regional Park, Rome; locationRemarks: Cava Fiorucci; decimalLatitude: 41.834106; decimalLongitude: 12.549264; geodeticDatum: WGS84; **Identification:** identifiedBy: Tommaso Fusco; dateIdentified: 2022; **Event:** samplingProtocol: Pitfall traps; eventDate: 2014-06-13; **Record Level:** collectionID: Roma3_5.8
- **Type status:** Other material. **Occurrence:** recordedBy: Fattorini S., Di Giulio A.; individualCount: 3; sex: male; lifeStage: adult; occurrenceID: CD968F42-A7DD-57D6-B801-F7AC876DF283; **Taxon:** scientificName: Zodarionitalicum (Canestrini, 1868); order: Araneae; family: Zodariidae; genus: Zodarion; **Location:** stateProvince: Rome; county: Rome; municipality: Rome; locality: Appia Antica Regional Park, Rome; locationRemarks: Cava Fiorucci; decimalLatitude: 41.834106; decimalLongitude: 12.549264; geodeticDatum: WGS84; **Identification:** identifiedBy: Tommaso Fusco; dateIdentified: 2022; **Event:** samplingProtocol: Pitfall traps; eventDate: 2014-06-24; **Record Level:** collectionID: Roma3_5.8
- **Type status:** Other material. **Occurrence:** recordedBy: Fattorini S., Di Giulio A.; individualCount: 2; sex: male; lifeStage: adult; occurrenceID: 9DF0CF14-2C92-518E-A42A-0DDB69D86B52; **Taxon:** scientificName: Zodarionitalicum (Canestrini, 1868); order: Araneae; family: Zodariidae; genus: Zodarion; **Location:** stateProvince: Rome; county: Rome; municipality: Rome; locality: Appia Antica Regional Park, Rome; locationRemarks: Farnesiana; decimalLatitude: 41.839667; decimalLongitude: 12.525528; geodeticDatum: WGS84; **Identification:** identifiedBy: Tommaso Fusco; dateIdentified: 2022; **Event:** samplingProtocol: Pitfall traps; eventDate: 2014-05-26; **Record Level:** collectionID: Roma3_5.8
- **Type status:** Other material. **Occurrence:** recordedBy: Fattorini S., Di Giulio A.; individualCount: 1; sex: male; lifeStage: adult; occurrenceID: 3EC6F1D5-679E-5E98-B85C-57979CA3030C; **Taxon:** scientificName: Zodarionitalicum (Canestrini, 1868); order: Araneae; family: Zodariidae; genus: Zodarion; **Location:** stateProvince: Rome; county: Rome; municipality: Rome; locality: Appia Antica Regional Park, Rome; locationRemarks: Farnesiana; decimalLatitude: 41.839667; decimalLongitude: 12.525528; geodeticDatum: WGS84; **Identification:** identifiedBy: Tommaso Fusco; dateIdentified: 2022; **Event:** samplingProtocol: Pitfall traps; eventDate: 2014-05-20; **Record Level:** collectionID: Roma3_5.8
- **Type status:** Other material. **Occurrence:** recordedBy: Fattorini S., Di Giulio A.; individualCount: 1; sex: male; lifeStage: adult; occurrenceID: 78395670-743A-515B-90B9-25925710020C; **Taxon:** scientificName: Zodarionitalicum (Canestrini, 1868); order: Araneae; family: Zodariidae; genus: Zodarion; **Location:** stateProvince: Rome; county: Rome; municipality: Rome; locality: Appia Antica Regional Park, Rome; locationRemarks: Farnesiana; decimalLatitude: 41.839667; decimalLongitude: 12.525528; geodeticDatum: WGS84; **Identification:** identifiedBy: Tommaso Fusco; dateIdentified: 2022; **Event:** samplingProtocol: Pitfall traps; eventDate: 2014-05-28; **Record Level:** collectionID: Roma3_5.8
- **Type status:** Other material. **Occurrence:** recordedBy: Fattorini S., Di Giulio A.; individualCount: 1; sex: male; lifeStage: adult; occurrenceID: 5AB0B263-8D2A-51B7-A2C8-5B4A244A5164; **Taxon:** scientificName: Zodarionitalicum (Canestrini, 1868); order: Araneae; family: Zodariidae; genus: Zodarion; **Location:** stateProvince: Rome; county: Rome; municipality: Rome; locality: Appia Antica Regional Park, Rome; locationRemarks: Farnesiana; decimalLatitude: 41.839667; decimalLongitude: 12.525528; geodeticDatum: WGS84; **Identification:** identifiedBy: Tommaso Fusco; dateIdentified: 2022; **Event:** samplingProtocol: Pitfall traps; eventDate: 2014-06-09; **Record Level:** collectionID: Roma3_5.8
- **Type status:** Other material. **Occurrence:** recordedBy: Fattorini S., Di Giulio A.; individualCount: 4; sex: 3 male, 1 female; lifeStage: adult; occurrenceID: 5D4E81BE-98C0-512F-88E4-842B7E532D83; **Taxon:** scientificName: Zodarionitalicum (Canestrini, 1868); order: Araneae; family: Zodariidae; genus: Zodarion; **Location:** stateProvince: Rome; county: Rome; municipality: Rome; locality: Appia Antica Regional Park, Rome; locationRemarks: Farnesiana; decimalLatitude: 41.839667; decimalLongitude: 12.525528; geodeticDatum: WGS84; **Identification:** identifiedBy: Tommaso Fusco; dateIdentified: 2022; **Event:** samplingProtocol: Pitfall traps; eventDate: 2014-06-19; **Record Level:** collectionID: Roma3_5.8
- **Type status:** Other material. **Occurrence:** recordedBy: Fattorini S., Di Giulio A.; individualCount: 15; sex: 12 male, 3 female; lifeStage: adult; occurrenceID: 695BA12D-9241-5F24-B8CC-45F6361D04FA; **Taxon:** scientificName: Zodarionitalicum (Canestrini, 1868); order: Araneae; family: Zodariidae; genus: Zodarion; **Location:** stateProvince: Rome; county: Rome; municipality: Rome; locality: Appia Antica Regional Park, Rome; locationRemarks: San Sebastiano; decimalLatitude: 41.855733; decimalLongitude: 12.515114; geodeticDatum: WGS84; **Identification:** identifiedBy: Tommaso Fusco; dateIdentified: 2022; **Event:** samplingProtocol: Pitfall traps; eventDate: 2014-05-20; **Record Level:** collectionID: Roma3_5.8
- **Type status:** Other material. **Occurrence:** recordedBy: Fattorini S., Di Giulio A.; individualCount: 1; sex: male; lifeStage: adult; occurrenceID: 969C3D67-12FC-5068-9318-0138C9EA1126; **Taxon:** scientificName: Zodarionitalicum (Canestrini, 1868); order: Araneae; family: Zodariidae; genus: Zodarion; **Location:** stateProvince: Rome; county: Rome; municipality: Rome; locality: Appia Antica Regional Park, Rome; locationRemarks: San Sebastiano; decimalLatitude: 41.855733; decimalLongitude: 12.515114; geodeticDatum: WGS84; **Identification:** identifiedBy: Tommaso Fusco; dateIdentified: 2022; **Event:** samplingProtocol: Pitfall traps; eventDate: 2014-06-09; **Record Level:** collectionID: Roma3_5.8
- **Type status:** Other material. **Occurrence:** recordedBy: Fattorini S., Di Giulio A.; individualCount: 5; sex: 4 male, 1 female; lifeStage: adult; occurrenceID: 05B91189-AC4D-5ACF-B1EC-8EBDD5279A93; **Taxon:** scientificName: Zodarionitalicum (Canestrini, 1868); order: Araneae; family: Zodariidae; genus: Zodarion; **Location:** stateProvince: Rome; county: Rome; municipality: Rome; locality: Appia Antica Regional Park, Rome; locationRemarks: San Sebastiano; decimalLatitude: 41.855733; decimalLongitude: 12.515114; geodeticDatum: WGS84; **Identification:** identifiedBy: Tommaso Fusco; dateIdentified: 2022; **Event:** samplingProtocol: Pitfall traps; eventDate: 2014-05-28; **Record Level:** collectionID: Roma3_5.8
- **Type status:** Other material. **Occurrence:** recordedBy: Fattorini S., Di Giulio A.; individualCount: 3; sex: male; lifeStage: adult; occurrenceID: DB41E9CE-BAAA-5A9B-BF6B-F041E10A7545; **Taxon:** scientificName: Zodarionitalicum (Canestrini, 1868); order: Araneae; family: Zodariidae; genus: Zodarion; **Location:** stateProvince: Rome; county: Rome; municipality: Rome; locality: Appia Antica Regional Park, Rome; locationRemarks: San Sebastiano; decimalLatitude: 41.855733; decimalLongitude: 12.515114; geodeticDatum: WGS84; **Identification:** identifiedBy: Tommaso Fusco; dateIdentified: 2022; **Event:** samplingProtocol: Pitfall traps; eventDate: 2014-06-09; **Record Level:** collectionID: Roma3_5.8
- **Type status:** Other material. **Occurrence:** recordedBy: Fattorini S., Di Giulio A.; individualCount: 3; sex: male; lifeStage: adult; occurrenceID: 43628F3A-B178-54E2-BFCF-1006F5EA44B7; **Taxon:** scientificName: Zodarionitalicum (Canestrini, 1868); order: Araneae; family: Zodariidae; genus: Zodarion; **Location:** stateProvince: Rome; county: Rome; municipality: Rome; locality: Appia Antica Regional Park, Rome; locationRemarks: San Sebastiano; decimalLatitude: 41.855733; decimalLongitude: 12.515114; geodeticDatum: WGS84; **Identification:** identifiedBy: Tommaso Fusco; dateIdentified: 2022; **Event:** samplingProtocol: Pitfall traps; eventDate: 2014-06-19; **Record Level:** collectionID: Roma3_5.8
- **Type status:** Other material. **Occurrence:** recordedBy: Fattorini S., Di Giulio A.; individualCount: 2; sex: 1 male, 1 female; lifeStage: adult; occurrenceID: 26E99610-3B8C-561E-9429-AB6A0DF90806; **Taxon:** scientificName: Zodarionitalicum (Canestrini, 1868); order: Araneae; family: Zodariidae; genus: Zodarion; **Location:** stateProvince: Rome; county: Rome; municipality: Rome; locality: Appia Antica Regional Park, Rome; locationRemarks: Tor Marancia; decimalLatitude: 41.850308; decimalLongitude: 12.503178; geodeticDatum: WGS84; **Identification:** identifiedBy: Tommaso Fusco; dateIdentified: 2022; **Event:** samplingProtocol: Pitfall traps; eventDate: 2013-10-28; **Record Level:** collectionID: Roma3_5.8
- **Type status:** Other material. **Occurrence:** recordedBy: Fattorini S., Di Giulio A.; individualCount: 1; sex: 3 male, 2 female; lifeStage: adult; occurrenceID: E50608FD-65EB-568F-AC1E-2AF10C44C5E9; **Taxon:** scientificName: Zodarionitalicum (Canestrini, 1868); order: Araneae; family: Zodariidae; genus: Zodarion; **Location:** stateProvince: Rome; county: Rome; municipality: Rome; locality: Appia Antica Regional Park, Rome; locationRemarks: Tor Marancia; decimalLatitude: 41.850308; decimalLongitude: 12.503178; geodeticDatum: WGS84; **Identification:** identifiedBy: Tommaso Fusco; dateIdentified: 2022; **Event:** samplingProtocol: Pitfall traps; eventDate: 2014-05-15; **Record Level:** collectionID: Roma3_5.8
- **Type status:** Other material. **Occurrence:** recordedBy: Fattorini S., Di Giulio A.; individualCount: 2; sex: female; lifeStage: adult; occurrenceID: EF7E597F-F7BE-5CFC-AA88-3ACEEB0C0309; **Taxon:** scientificName: Zodarionitalicum (Canestrini, 1868); order: Araneae; family: Zodariidae; genus: Zodarion; **Location:** stateProvince: Rome; county: Rome; municipality: Rome; locality: Appia Antica Regional Park, Rome; locationRemarks: Tor Marancia; decimalLatitude: 41.850308; decimalLongitude: 12.503178; geodeticDatum: WGS84; **Identification:** identifiedBy: Tommaso Fusco; dateIdentified: 2022; **Event:** samplingProtocol: Pitfall traps; eventDate: 2014-05-26; **Record Level:** collectionID: Roma3_5.8
- **Type status:** Other material. **Occurrence:** recordedBy: Fattorini S., Di Giulio A.; individualCount: 1; sex: 6 male, 2 female; lifeStage: adult; occurrenceID: A0C4E0F8-0AD0-52B4-819F-24BFDAD6B96A; **Taxon:** scientificName: Zodarionitalicum (Canestrini, 1868); order: Araneae; family: Zodariidae; genus: Zodarion; **Location:** stateProvince: Rome; county: Rome; municipality: Rome; locality: Appia Antica Regional Park, Rome; locationRemarks: Tor Marancia; decimalLatitude: 41.850308; decimalLongitude: 12.503178; geodeticDatum: WGS84; **Identification:** identifiedBy: Tommaso Fusco; dateIdentified: 2022; **Event:** samplingProtocol: Pitfall traps; eventDate: 2014-06-05; **Record Level:** collectionID: Roma3_5.8
- **Type status:** Other material. **Occurrence:** recordedBy: Fattorini S., Di Giulio A.; individualCount: 4; sex: 2 male, 2 female; lifeStage: adult; occurrenceID: F293CE80-7313-558D-9683-5FD5DECFB4D9; **Taxon:** scientificName: Zodarionitalicum (Canestrini, 1868); order: Araneae; family: Zodariidae; genus: Zodarion; **Location:** stateProvince: Rome; county: Rome; municipality: Rome; locality: Appia Antica Regional Park, Rome; locationRemarks: Tor Marancia; decimalLatitude: 41.850308; decimalLongitude: 12.503178; geodeticDatum: WGS84; **Identification:** identifiedBy: Tommaso Fusco; dateIdentified: 2022; **Event:** samplingProtocol: Pitfall traps; eventDate: 2014-06-17; **Record Level:** collectionID: Roma3_5.8
- **Type status:** Other material. **Occurrence:** recordedBy: Fattorini S., Di Giulio A.; individualCount: 3; sex: 2 male, 1 female; lifeStage: adult; occurrenceID: 2204478B-D1AC-5BE5-B243-4986129015DF; **Taxon:** scientificName: Zodarionitalicum (Canestrini, 1868); order: Araneae; family: Zodariidae; genus: Zodarion; **Location:** stateProvince: Rome; county: Rome; municipality: Rome; locality: Appia Antica Regional Park, Rome; locationRemarks: Torre Selce; decimalLatitude: 41.816611; decimalLongitude: 12.560667; geodeticDatum: WGS84; **Identification:** identifiedBy: Tommaso Fusco; dateIdentified: 2022; **Event:** samplingProtocol: Pitfall traps; eventDate: 2014-05-26; **Record Level:** collectionID: Roma3_5.8

##### Distribution

Europe, Caucasus. European (EUR) chorotype.

#### Zodarion pusio

Simon, 1914

E9D85944-E9ED-52E4-ACC0-11D3B264C0CB

##### Materials

- **Type status:** Other material. **Occurrence:** recordedBy: Fattorini S., Di Giulio A.; individualCount: 3; sex: male; lifeStage: adult; occurrenceID: 468E20E2-A114-514A-9DEE-0EF759761825; **Taxon:** scientificName: Zodarionpusio Simon, 1914; order: Araneae; family: Zodariidae; genus: Zodarion; **Location:** country: Italy; countryCode: IT; stateProvince: Rome; county: Rome; municipality: Rome; locality: Appia Antica Regional Park, Rome; locationRemarks: Casal Verbeni; decimalLatitude: 41.815250; decimalLongitude: 12.552222; geodeticDatum: WGS84; **Identification:** identifiedBy: Tommaso Fusco; dateIdentified: 2022; **Event:** samplingProtocol: Pitfall traps; eventDate: 2014-05-26; **Record Level:** collectionID: Roma3_5.8
- **Type status:** Other material. **Occurrence:** recordedBy: Fattorini S., Di Giulio A.; individualCount: 1; sex: male; lifeStage: adult; occurrenceID: 64B68F82-0797-5CEA-A420-D9A2881BE05D; **Taxon:** scientificName: Zodarionpusio Simon, 1914; order: Araneae; family: Zodariidae; genus: Zodarion; **Location:** country: Italy; countryCode: IT; stateProvince: Rome; county: Rome; municipality: Rome; locality: Appia Antica Regional Park, Rome; locationRemarks: Casal Verbeni; decimalLatitude: 41.815250; decimalLongitude: 12.552222; geodeticDatum: WGS84; **Identification:** identifiedBy: Tommaso Fusco; dateIdentified: 2022; **Event:** samplingProtocol: Pitfall traps; eventDate: 2014-06-05; **Record Level:** collectionID: Roma3_5.8
- **Type status:** Other material. **Occurrence:** recordedBy: Fattorini S., Di Giulio A.; individualCount: 2; sex: 1 male, 1 female; lifeStage: adult; occurrenceID: D06524E8-34E2-5A36-974A-9E6DADC2D61F; **Taxon:** scientificName: Zodarionpusio Simon, 1914; order: Araneae; family: Zodariidae; genus: Zodarion; **Location:** country: Italy; countryCode: IT; stateProvince: Rome; county: Rome; municipality: Rome; locality: Appia Antica Regional Park, Rome; locationRemarks: Casal Verbeni; decimalLatitude: 41.815250; decimalLongitude: 12.552222; geodeticDatum: WGS84; **Identification:** identifiedBy: Tommaso Fusco; dateIdentified: 2022; **Event:** samplingProtocol: Pitfall traps; eventDate: 2014-06-13; **Record Level:** collectionID: Roma3_5.8
- **Type status:** Other material. **Occurrence:** recordedBy: Fattorini S., Di Giulio A.; individualCount: 1; sex: male; lifeStage: adult; occurrenceID: A24F5A4B-19B3-59E6-84BF-CCAD81D9C0B5; **Taxon:** scientificName: Zodarionpusio Simon, 1914; order: Araneae; family: Zodariidae; genus: Zodarion; **Location:** country: Italy; countryCode: IT; stateProvince: Rome; county: Rome; municipality: Rome; locality: Appia Antica Regional Park, Rome; locationRemarks: Casal Verbeni; decimalLatitude: 41.815250; decimalLongitude: 12.552222; geodeticDatum: WGS84; **Identification:** identifiedBy: Tommaso Fusco; dateIdentified: 2022; **Event:** samplingProtocol: Pitfall traps; eventDate: 2014-06-24; **Record Level:** collectionID: Roma3_5.8
- **Type status:** Other material. **Occurrence:** recordedBy: Fattorini S., Di Giulio A.; individualCount: 1; sex: male; lifeStage: adult; occurrenceID: FFD4A78C-910C-5E02-A261-D3B338095110; **Taxon:** scientificName: Zodarionpusio Simon, 1914; order: Araneae; family: Zodariidae; genus: Zodarion; **Location:** country: Italy; countryCode: IT; stateProvince: Rome; county: Rome; municipality: Rome; locality: Appia Antica Regional Park, Rome; locationRemarks: Farnesiana; decimalLatitude: 41.839667; decimalLongitude: 12.525528; geodeticDatum: WGS84; **Identification:** identifiedBy: Tommaso Fusco; dateIdentified: 2022; **Event:** samplingProtocol: Pitfall traps; eventDate: 2014-06-19; **Record Level:** collectionID: Roma3_5.8
- **Type status:** Other material. **Occurrence:** recordedBy: Fattorini S., Di Giulio A.; individualCount: 1; sex: female; lifeStage: adult; occurrenceID: 83C06CA8-941F-5F16-9504-7525B8C6B2C8; **Taxon:** scientificName: Zodarionpusio Simon, 1914; order: Araneae; family: Zodariidae; genus: Zodarion; **Location:** country: Italy; countryCode: IT; stateProvince: Rome; county: Rome; municipality: Rome; locality: Appia Antica Regional Park, Rome; locationRemarks: San Sebastiano; decimalLatitude: 41.855733; decimalLongitude: 12.515114; geodeticDatum: WGS84; **Identification:** identifiedBy: Tommaso Fusco; dateIdentified: 2022; **Event:** samplingProtocol: Pitfall traps; eventDate: 2014-06-09; **Record Level:** collectionID: Roma3_5.8
- **Type status:** Other material. **Occurrence:** recordedBy: Fattorini S., Di Giulio A.; individualCount: 1; sex: male; lifeStage: adult; occurrenceID: 05F566DC-E3E4-5398-9981-C23B26D067CF; **Taxon:** scientificName: Zodarionpusio Simon, 1914; order: Araneae; family: Zodariidae; genus: Zodarion; **Location:** country: Italy; countryCode: IT; stateProvince: Rome; county: Rome; municipality: Rome; locality: Appia Antica Regional Park, Rome; locationRemarks: Torre selce; decimalLatitude: 41.816611; decimalLongitude: 12.560667; geodeticDatum: WGS84; **Identification:** identifiedBy: Tommaso Fusco; dateIdentified: 2022; **Event:** samplingProtocol: Pitfall traps; eventDate: 2014-05-26; **Record Level:** collectionID: Roma3_5.8
- **Type status:** Other material. **Occurrence:** recordedBy: Fattorini S., Di Giulio A.; individualCount: 1; sex: male; lifeStage: adult; occurrenceID: 17B290AE-1FBD-5FB5-B0E1-31E7C65F6F96; **Taxon:** scientificName: Zodarionpusio Simon, 1914; order: Araneae; family: Zodariidae; genus: Zodarion; **Location:** country: Italy; countryCode: IT; stateProvince: Rome; county: Rome; municipality: Rome; locality: Appia Antica Regional Park, Rome; locationRemarks: Torre Selce; decimalLatitude: 41.816611; decimalLongitude: 12.560667; geodeticDatum: WGS84; **Identification:** identifiedBy: Tommaso Fusco; dateIdentified: 2022; **Event:** samplingProtocol: Pitfall traps; eventDate: 2014-06-05; **Record Level:** collectionID: Roma3_5.8

##### Distribution

France, Italy, Slovenia, Croatia, Bosnia and Herzegovina, Tunisia. Mediterranean (MED) chorotype.

## Analysis

A total of 1756 individuals, belonging to 120 species, 83 genera and 28 families, were identified (119 at species level, one at genus level). Seventy species are new for the Province of Rome, thirty-nine for the Latium Region and two are new additions to the Italian fauna (Table 1; Pantini and Isaia (2019)): *Pelecopsisdigitulus* and *Palliduphantesarenicola*. Forty-one species were collected in the autumn/winter period and 107 species in the spring/summer period.

The species recorded in the study area (ca. 46 km^2^) represent about 37% of the Province of Rome (5,363 km^2^), 28% of the Latium Region (17,232 km^2^) and 7% of the whole Italian territory (302,073 km^2^).

Using the *c*-parameter of the species-area relationship (SAR) as a measure of species richness standardised by area with *z* = 0.25, we obtained an estimate of about 46 species for an area of one km^2^ in the study area, about 38 species for an area of one km^2^ in the Rome Province and in the Latium Region and about 73 species for an area of one km^2^ in the whole Italian territory. With *z* = 0.18, we obtained an estimate of about 60 species per unit area (one km^2^) in the study area, about 69 species per unit area in the Rome Province, about 74 species per unit area in the Latium Region and about 177 species per unit area in the whole Italian territory. Finally, with *z* = 0.14, we obtained an estimate of about 70 species per unit area (one km^2^) in the study area, about 97 species per unit area in the Rome Province, 110 species per unit area in the Latium Region and about 293 species per unit area in the whole Italian Peninsula.

**Figure 14.**
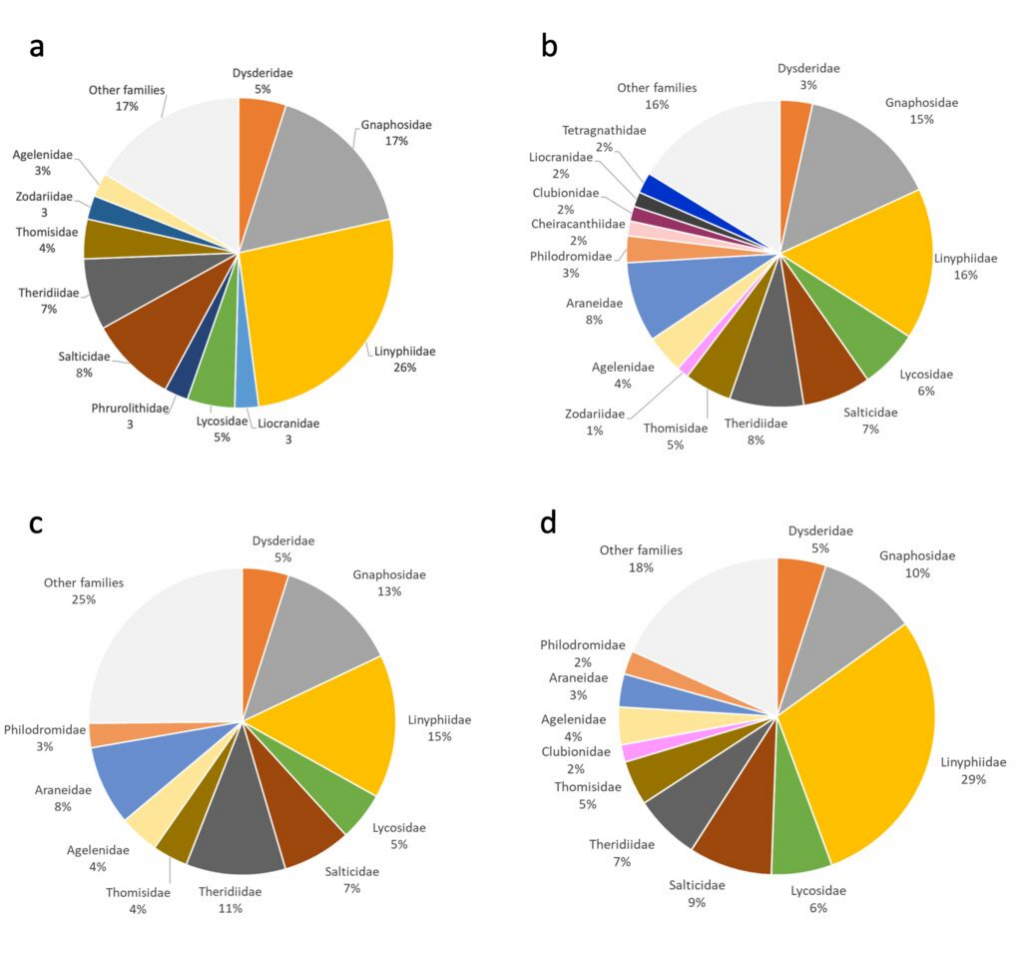
Taxonomic composition (% of species in each family) for the spider fauna recorded in a protected green space in urban Rome, Italy (the Appia Antica Regional Park) (**a**) in comparison with that obtained for the Province of Rome (**b**), the Latium Region (**c**) and the whole Italian territory (**d**) (data was obtained from araneae.it, Pantini and Isaia (2019)).

The most represented families in the study area in terms of species richness were Gnaphosidae and Linyphiidae, which taken together accounted for more than 40% of the sampled fauna (Fig. 14a). Gnaphosidae and Linyphiidae are also the richest families in the Rome Province (Fig. 14b), in Latium Region (Fig. 14c) and in the whole Italian territory (Fig. 14d).

**Figure 15.**
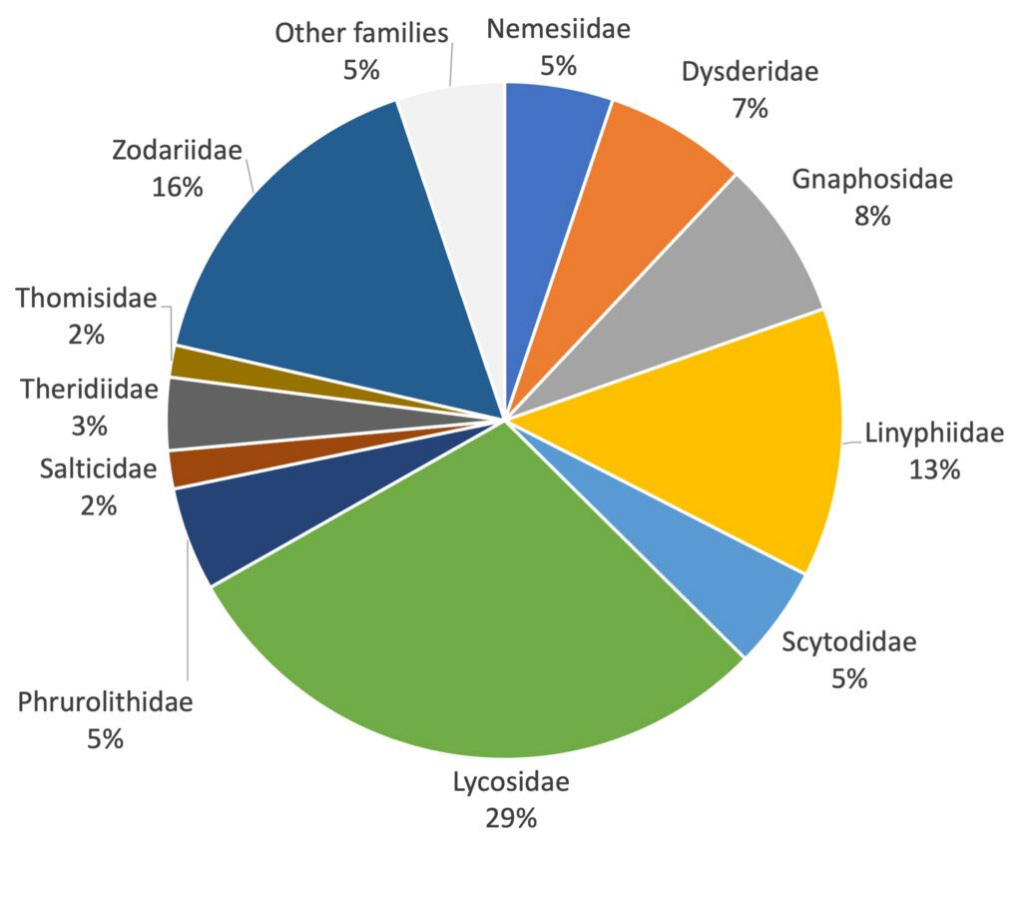
Abundance (% of sampled individuals) of spider families in a protected green space in urban Rome, Italy (the Appia Antica Regional Park).

In terms of abundance, Lycosidae are the most represented family, followed by Zodariidae, Linyphiidae and Gnaphosidae (Fig. 15). Thus, Linyphiidae and (to a lower extent) Gnaphosidae are, in the ground spider fauna of the study area, important families in terms of both species richness and abundance, but the contribution of other families to richness and abundance was very different, with Lycosidae and Zodariidae being numerically abundant, but poor in species.

**Figure 16.**
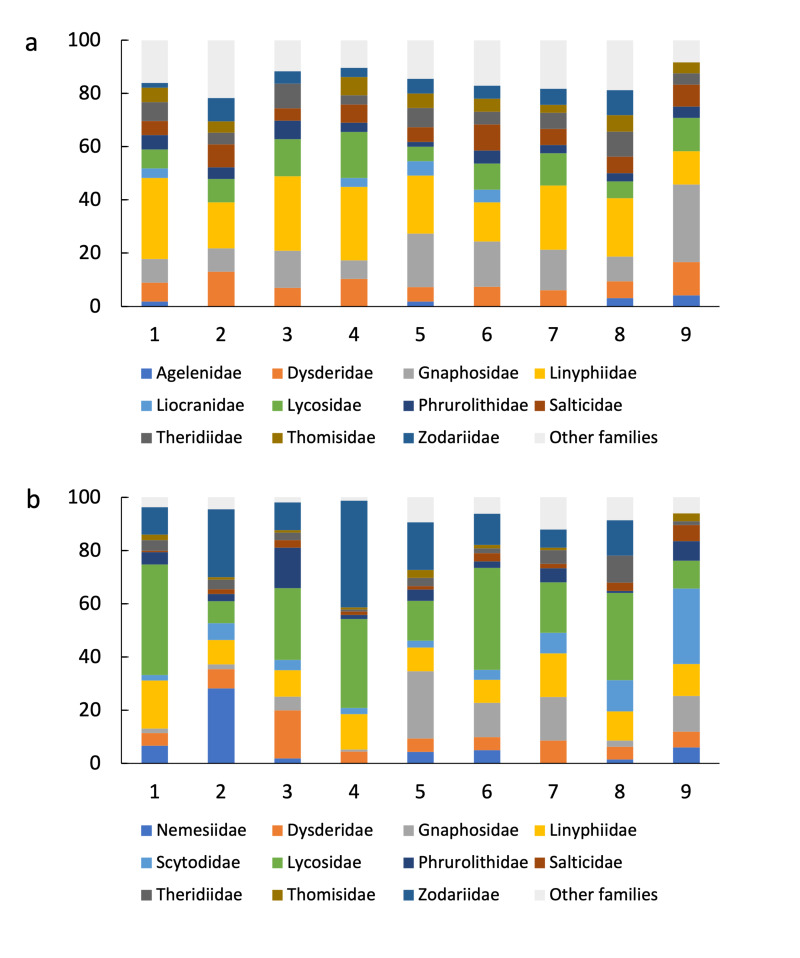
Taxonomic composition (% of species in each family, **a**) and abundance (% of individuals in each family, **b**) of spider assemblages sampled in different sites (numbered as in Fig. 1) within a protected green space in urban Rome, Italy (the Appia Antica Regional Park)

At the site level, Linyphiidae were the richest family almost everywhere, while Gnaphosidae were particularly rich in species in the most peripheral site (Fig. 16a). Site 1 (in the Caffarella Park) was the site that had the highest richness of species (57), but was also the site with the highest number of pitfall traps (19), followed by site 5 (55 species) that, however, had a lower number of pitfall traps (5). The abundance of the various families varied greatly amongst sampling sites, with no apparent distinct patterns. Lycosidae were, in general, amongst the most abundant spiders everywhere, whereas Scytodidae, which are, in general, scarce, were the most abundant in the most peripherical site (Fig. 16b).

**Figure 17.**
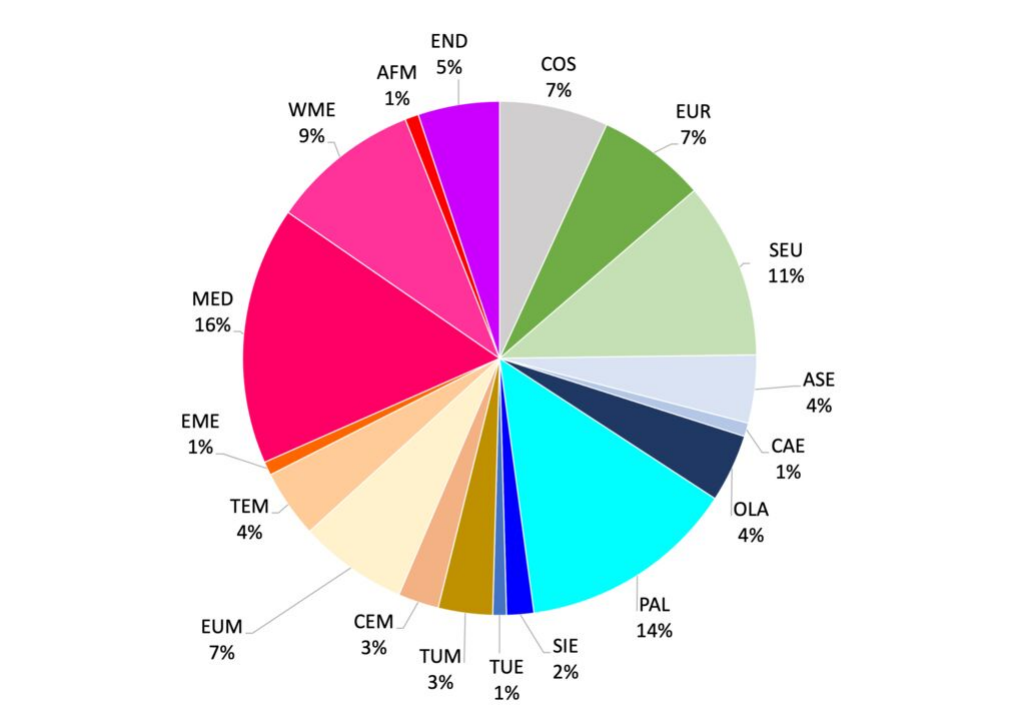
Biogeographical composition (% of chorotypes) of the spider fauna recorded in a protected green space in urban Rome, Italy (the Appia Antica Regional Park). Chorotypes are as follows: AFM = Afrotropico-Mediterranean, ASE = Asiatic-European, CAE = Centralasiatic-European, CEM = Centralasiatic-Europeo-Mediterranean, COS = Cosmopolitan, END = Italian endemic, EME = E-Mediterranean, EUM = Europeo-Mediterannean, EUR = European, MED = Mediterranean, OLA = Holarctic, PAL = Palearctic, SEU = S-European, SIE = Sibero-European, TEM = Turano-Europeo-Mediterranean, TUE = Turano-European, TUM = Turano-Mediterranean, WEU = W- European, WME = W-Mediterranean

**Figure 18.**
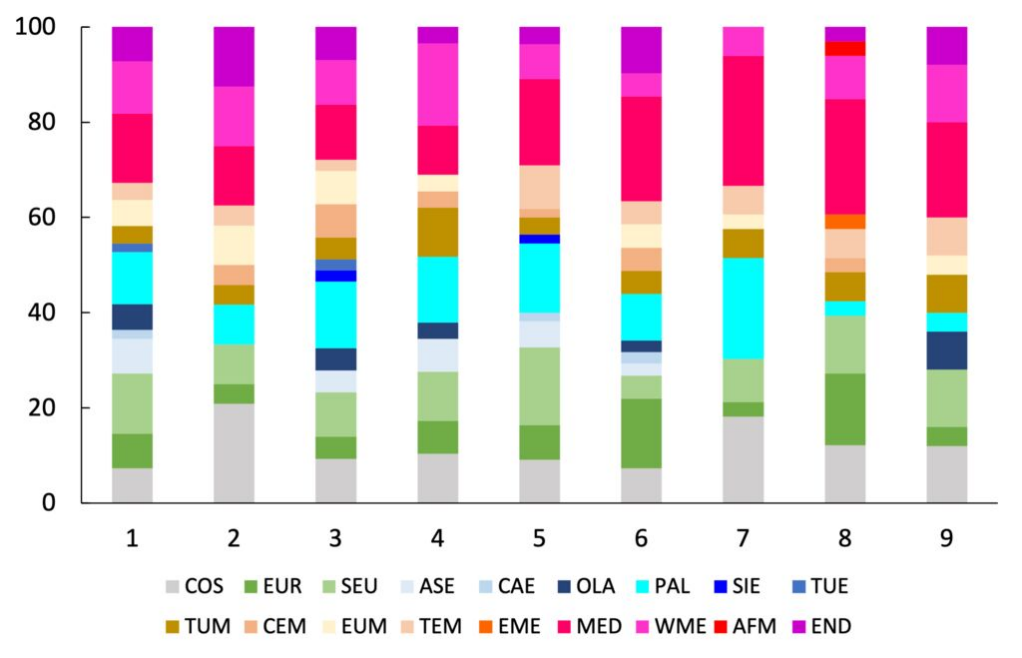
Biogeographical composition (% of chorotypes) of the spider assemblages sampled in different sites (numbered as in Fig. 1) in a protected green space in urban Rome, Italy (the Appia Antica Regional Park). Chorotypes codes are as in Fig. 14.

From a biogeographical point of view, most of the species belong to chorotypes that extend for large areas across Europe and the Palaearctic (Fig. 17). This pattern is also found at the site level (Fig. 18). However, the study area also hosts species with more restricted distribution and about 5% of the recorded species are endemic to Italy.

## Discussion

Studies examining urban araneofauna in Italy are relatively scarce, with only a handful of works conducted in the cities of Pavia (Giordano et al. 2002), Milan (Pilon et al. 2010), Venice (Hansen 1988, Hansen 1992, Hansen 1995, Hansen 1996), Verona (Ballarin and Petri 2021) and Turin (Mammola et al. 2018, Piano et al. 2020a).

Our research represents a novelty in Italy, as previous studies in urban areas were conducted only in the north of the country. Moreover, our study considered a green space of special importance, as it covers a very large area within the city, encompassing the full rural-urban gradient. This green space is also one of the largest urban green spaces in Europe and hosts an exceptionally high botanic diversity (Iamonico 2022), which can explain the very high number of spider species and families recorded in this study, compared to those of other urban areas. For example, Ballarin and Petri (2021) found 46 species in Verona in an area of about 100 hectares, Piano et al. (2020a) identified 66 species in Turin in an area of around 27 hectares and Pilon et al. (2010) found 27 species near Milan in urban parks with a total area of around 240 hectares. Hansen, through his numerous studies in many urban areas around Venice with over 190 species reported (Hansen 1988, Hansen 1992, Hansen 1995, Hansen 1996), has shown that intensive studies can lead to the collection of high numbers of species in these areas.

As the presence of a complex vegetation structure can increase the diversity of spider communities by allowing them to exploit a great variety of microhabitats (Langellotto and Denno 2004, Sarthou et al. 2014, Delgado de la Flor et al. 2017, Trigos-Peral et al. 2020), the variety of vegetation forms in the study area (Iamonico 2022) might represent an important reason for the high diversity of its spider fauna, which may be similar to or even richer (species per unit area) than those of the Province of Rome and Latium Region when using *z* = 0.25. When using lower values of *z*, however, the number of species per unit area in the Appia Regional Park appears lower than those that can be calculated at larger spatial scales. Moreover, faunal inventories for the Province of Rome and Latium Region may be largely incomplete and, hence, the species richness at this scale underestimated. On the other hand, it is important to note that our sampling approach allowed the collection of only ground-dwelling species, whereas estimates of spider richness at larger scales included also species with different ecology.

Despite the lower numbers of species collected in autumn, we found in this period some species that were not present in the spring sampling, which highlights the importance of performing spider sampling in different seasons to obtain an adequate estimate of the spider richness in temperate ecosystems because of the presence of winter specialists (Uetz and Unzicker 1975, Curtis 1980, Churchill and Arthur 1999). Despite this, it seems that the best time to sample spiders in the Mediterranean environment remains the late spring period, as already indicated by Cardoso et al. (2007).

We found two species that are new to the Italian fauna. *Pelecopsisdigitulus* (1♀) (Linyphiidae) is a rare species previously known from semi-arid to humid areas of Algeria and Corsica (Bosmans and Abrous 1992, Lissner 2016). *Palliduphantesarenicola* (2♀, 3♂) (Linyphiidae) is a species associated with open grasslands and dry meadows, previously reported from France and Switzerland (Denis 1964, Pozzi and Hänggi 1998). Furthermore, we found some interesting and rare species of ground spiders, such as: *Zangherellaalgerica* (1♀), already known from central Italy and possibly with a distribution in the Mediterranean wider than previously assumed (Brignoli 1981); *Silhouettellaloricatula* (2♀, 2♂), a small oonopid found mostly in southern Europe in litter and under stones (Roewer 1942, Blick et al. 2016); *Orchestinalongipes* (5♀, 1♂), a rare litter species known only from a few localities in Latium, Tuscany and Veneto (Brignoli 1967a, Ballarin and Petri 2021), in Corsica (Déjean 2024) and Portugal (Wunderlich 2023); *Argennasubnigra* (11♂), which, up to now, was only recorded in northern Italy (Pantini and Isaia 2019); *Micariapallipes* (1♀), a rare ant-mimicking Gnaphosidae with a Turano-Mediterranean distribution (Tuneva 2007, Branco et al. 2019, Pantini and Isaia 2019, Benhacene et al. 2023, Danışman et al. 2024); *Palliduphantesbyzantinus* (5♂), mostly known from east Europe (Brignoli 1979c, Blagoev et al. 2018, Danışman et al. 2024) and recorded for Italy only from southern areas (Ijland and Van Helsdingen 2016); *Scutpelecopsiskrausi* (4♀, 1♂), only found in the Balkans (Schröder et al. 2010, Blagoev et al. 2018, Blick 2018) and in north Italy (Hansen 1995); *Euryopisdentigera* (1♀), a small and rare Theridiidae only known from a few localities in Europe (Simon 1880, Blagoev 2002, Ijland and Van Helsdingen 2016). Another interesting species that was found in the study area is *Turkozelotesnoname* (1♀) (Gnaphosidae). This species, described from France (Mazzia and Cornic 2020) and recently found in Italy for the first time (Trotta 2024), was collected in agricultural patches that are common in the Appia Antica Regional Park. On the whole, six Italian endemic species were found in the study area: *Dysderaromana*, known exclusively from lowland and hillside localities near the Tyrrhenian coast of central and south Latium and from Emilia-Romagna (Gasparo and Di Franco 2008, Lami et al. 2023); the recently-described *Centromerustongiorgii*, which was already found in Latium at Canale Monterano (Ballarin and Pantini 2020), but which is also present in Abruzzo, Basilicata, Marche, Umbria, Emilia-Romagna, Liguria, Lombardy and Veneto (Ballarin and Pantini 2020, Nardi and Marini 2021); *Cybaeodesmarinae* a nocturnal spider found in Thyrrenian areas of Latium, Sardinia, Calabria, Sicily, Liguria and Tuscany (Di Franco 1989, Pantini and Mazzoleni 2018, Picchi 2020, Caria et al. 2021, Trotta 2023); *Harpacteasardoa* found in south Sardinia and Latium (Alicata 1966, Brignoli 1979a); *Pseudeuophrysperdifumo*, only known from a few sites in Calabria and Campania (Van Helsdingen 2015); *Nemesiabosmansi*, recently described by Decae (2024) from sandy dunes of the Parco Nationale del Circeo (Latium). It is also important to mention some species with limited distribution like *Araeoncuslongisculus* and *Syedranigrotibialis*, which can be found only in mainland Italy, Sardinia and Corsica (Simon 1926, Müller 1986, Pantini and Isaia 2019); *Dysderalantosquensis*, which has an Alpino-Appenninic distribution and is mainly distributed in central and northern Italy and on the French border (Řezáč et al. 2018); *Dysderabottaziae*, an Appennino-Dinaric sub-endemic species found in southern Italy, Croatia and Bosnia and Herzegovina (Deeleman-Reinhold and Deeleman 1988, Komnenov 2010, Pantini and Isaia 2019); *Mecopistheslatinus*, a small Linyphiidae mostly found in central Italy (Pantini and Isaia 2019), but also recorded in southern Switzerland (Hänggi 1990). We also found two alien, non-European species: *Osteariusmelanopygius* and *Erigoneautumnalis*. *Osteariusmelanopygius* is a South American species first reported for Europe from England in 1906 (Locket and Millidge 1953) and which then expanded eastwards (Růžička 1995). In Italy, it is distributed in many regions, both in the north and in the south (Pantini and Isaia 2019), but this is the first record for Latium. *Erigoneautumnalis*, native to North-Central America (Crosby and Bishop 1928) has been widespread in Europe since 1988 (Hänggi 1990) due to its ability to disperse over large distances by ballooning (Forster 1971, Dean and Sterling 1985). In fact, as many spiders can be easily transported (Decae 1987, Luo and Li 2015), exotic species found in Europe are probably more widespread than generally assumed, remaining undetected from many areas because of the lack of specific studies (Kempf et al. 2021). In particular, urban green spaces are an easy target for invasive species that can benefit from movements of plants and materials (Bestelmeyer et al. 2015). In Italy, this species has been widespread since 1994 and can be found in most of the regions (Pantini and Isaia 2019), but also, in this case, it is a new record for Latium, although it is probably quite common there.

Linyphiidae and Gnaphosidae were the most species-rich families in our samples, which is consistent with the fact that they are also the two families with the greatest richness in Latium and in Italy. Linyphiidae and Gnaphosidae are also recorded amongst the richest families in urban ecosystems (Fedoriak et al. 2012, Horváth et al. 2014, Otoshi et al. 2015, Polchaninova 2016, Delgado de la flor et al. 2020, Argañaraz et al. 2023). Species in these groups are often associated with the ground and are, therefore, easily captured with the use of pitfall traps, which can explain their abundance in our study. Moreover, Gnaphosidae are generally abundant in all Mediterranean areas (Cardoso et al. 2007) and the dry shrublands common in the study area may represent a suitable habitat for these spiders. Another species-rich group in our study was the Salticidae, which is consistent with other urban studies (Argañaraz et al. 2018) and their preference for warm climates (Nyffeler and Sunderland 2003). Theridiidae, which may exhibit a high richness in urban environments (Otoshi et al. 2015, Argañaraz et al. 2018, Kempf et al. 2021), ranked fourth in species richness in our study. As theridids include species that are poorly sampled by pitfall trapping, it is possible that the richness of this family in our study was underestimated.

Taxonomic composition varied amongst sites without any clear pattern with, however, a higher relative richness of Gnaphosidae in the most peripheral site. This lack of clear patterns could probably be due to the high vegetational diversity of the study area (Iamonico 2022) which could create many different microhabitats that favour different species.

Lycosidae were the most abundant family (26%), as observed in other urban studies (Fraser and Frankie 1986, Shochat et al. 2004, Petillon et al. 2011, Horváth et al. 2014, Otoshi et al. 2015, Delgado de la flor et al. 2020) followed by Zodariidae (16%) and Linyphiidae (15%). The high abundance of Lycosidae and Linyphiidae in the study area and in most of the sampling sites can be explained by the prevalence of agricultural fields and open habitats with Mediterranean vegetation. Lycosidae are known to be mainly associated with semi-arid open areas (Toft 1999, Shochat et al. 2004, Polchaninova 2016) and their activity density shows a positive correlation with the extent of agricultural landscapes (Otoshi et al. 2015), as they may provide these spiders with diverse and abundant prey (Sunderland and Samu 2000). The relatively high abundance of linyphiids can be explained by the fact that small-sized linyphiids are favoured in highly-disturbed sites, where larger spiders, such as agelenids and lycosids, which are typically found in meadows and pocket grasslands (Delgado de la flor et al. 2020), are disadvantaged. For example, during ecological successions, Linyphiidae often act as pioneer species in intensively-managed grasslands before being largely replaced by Lycosidae (Nentwig 1988, Shochat et al. 2004). The high abundance of linyphiids in the study area is largely due to the dominance of two urbanophilous species: *Diplostylaconcolor*, which is common in urban environments (Fedoriak et al. 2012, Horváth et al. 2014) and *Tenuiphantestenuis*, which is associated with open environments and urban green areas (Trigos-Peral et al. 2020). The high abundance of Zodariidae, especially in some sites placed closer to the city centre or at intermediate distances, can be explained by their feeding habits. All zodariids collected in this study belong the genus *Zodarion*. This genus is known to feed on ants (Pekár 2004, Pekár 2005, Pekár et al. 2008), which are abundant arthropods even in urbanised and disturbed areas. Moreover, these spiders are active hunters and, therefore, can be caught with pitfall traps in large numbers, especially if the traps are placed close to ant nests or ant trails. Lycosidae were dominant in most sites and Linyphiidae (although never particularly abundant) were recorded in similar proportions through the gradient. Thus, both groups showed no particular preference for the ecological conditions subsumed by the rural-urban gradient, while Gnaphosidae were particularly abundant in the central part of the gradient (sites, 5, 6 and 7), probably because most of the species found in this study are linked to Mediterranean areas and agroecosystems which are more abundant in this part of the gradient. The Scytodidae, represented in our sampling by a single species (*Scytodesthoracica*), which is a common species in natural and urban environments (Roberts 1995, Le Peru 2011, Nentwig et al. 2024), dominated the spider community of the most peripheral site, which suggests a preference for less anthropogenised habitats for this species in our study area.

From a biogeographical point of view, most species appear to be widely distributed in Europe and in the Palaearctic Region. Under the assumption that species with wider ranges have broader ecological tolerances (Fattorini et al. 2013), these results are consistent with the higher percentage of generalist species commonly found in urban habitats (Antov et al. 2004, Argañaraz et al. 2018, Trigos-Peral et al. 2020). However, the presence of species with restricted distributions and endemic to the Italian teritory suggests that the study area has a relatively high degree of naturalness.

Overall, these results support the idea that urban green spaces, which are known to host rare spiders, can play an important role in the conservation of ground-dwelling spiders (Horváth et al. 2014).

In conclusion, our study showed how a single urban green space can host a high diversity of spiders. The abundance of various spider species, including rare and often overlooked ones, recorded during our sampling, underlines the critical role that urban green spaces may play as reservoirs for biodiversity (Horváth et al. 2014, Argañaraz et al. 2018) providing spiders with a variety of habitats and, hence, supporting species with varied ecological needs (Trigos-Peral et al. 2020). Finally, our findings illustrate how detailed studies conducted at the local scale may provide important new information on broader scales with the discovery of species new at regional or even national scale.

## Figures and Tables

**Figure 2a.**
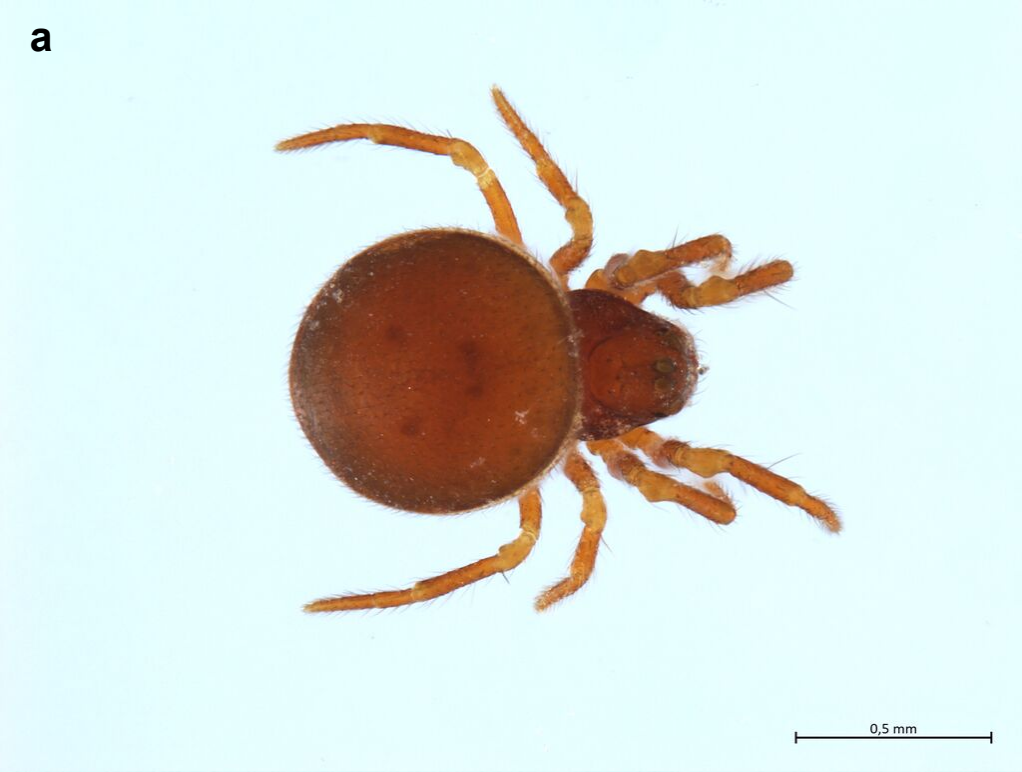
Habitus, dorsal view;

**Figure 2b.**
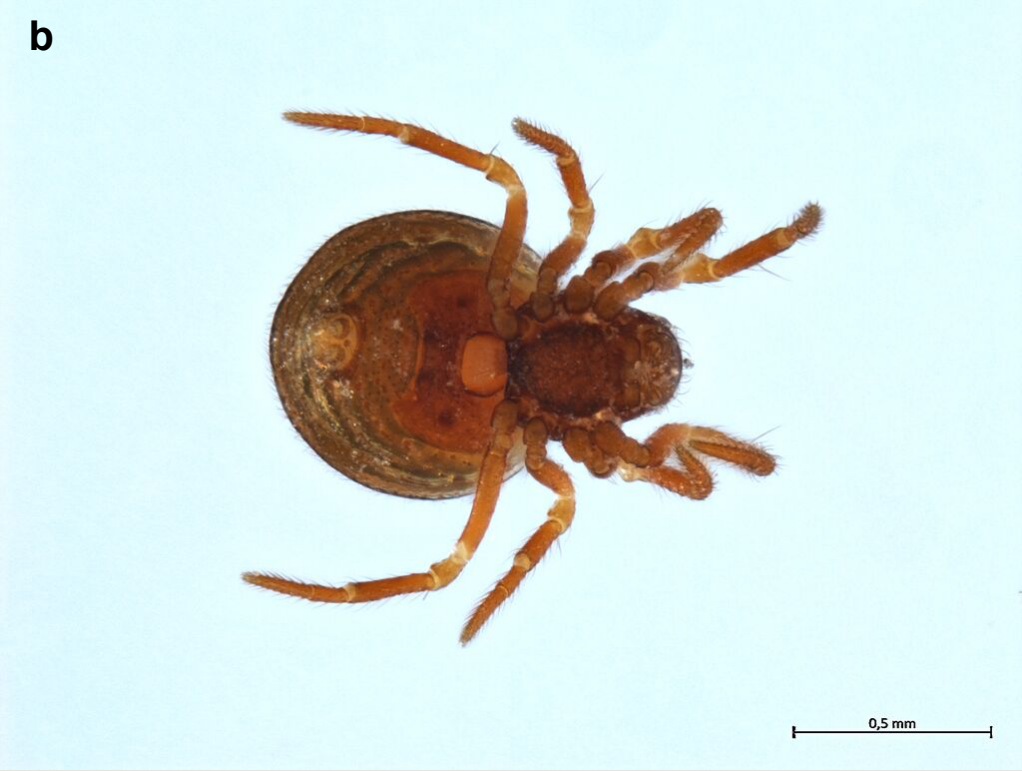
Habitus, ventral view.

**Figure 3a.**
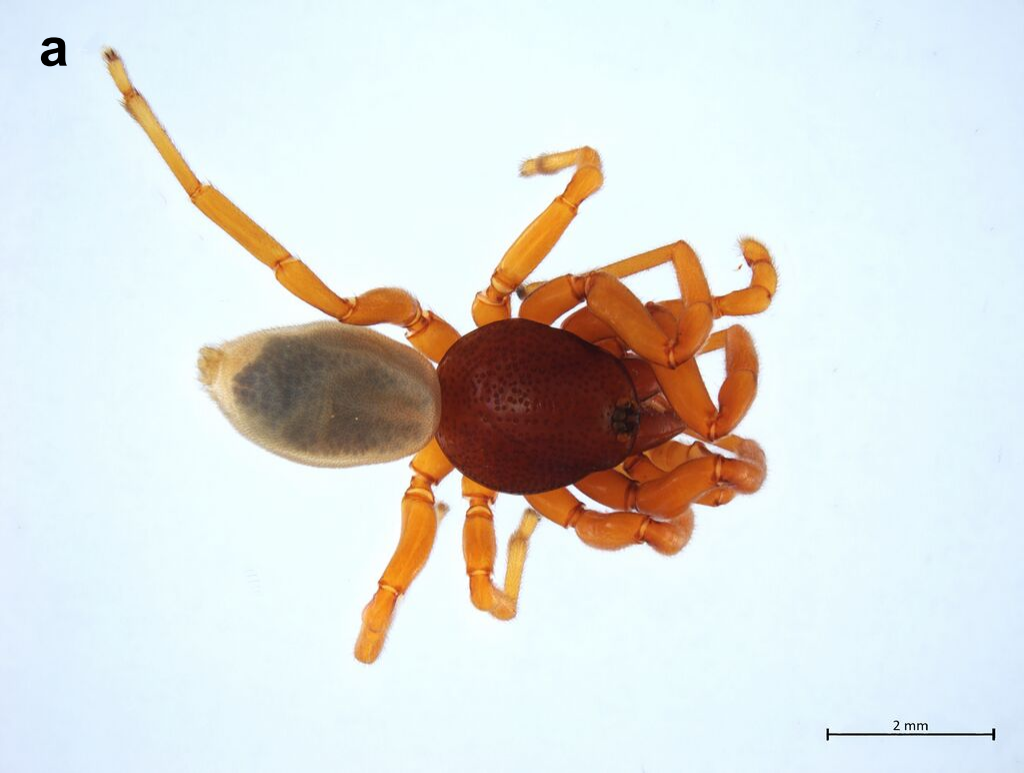
Habitus, dorsal view;

**Figure 3b.**
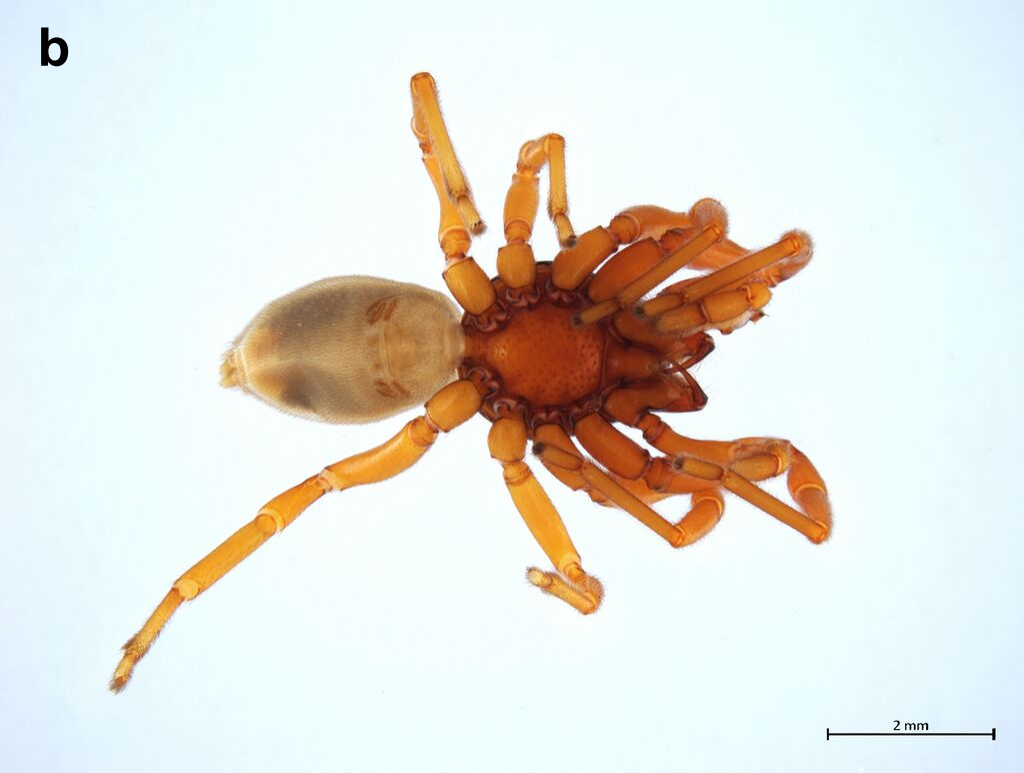
Habitus, ventral view;

**Figure 3c.**
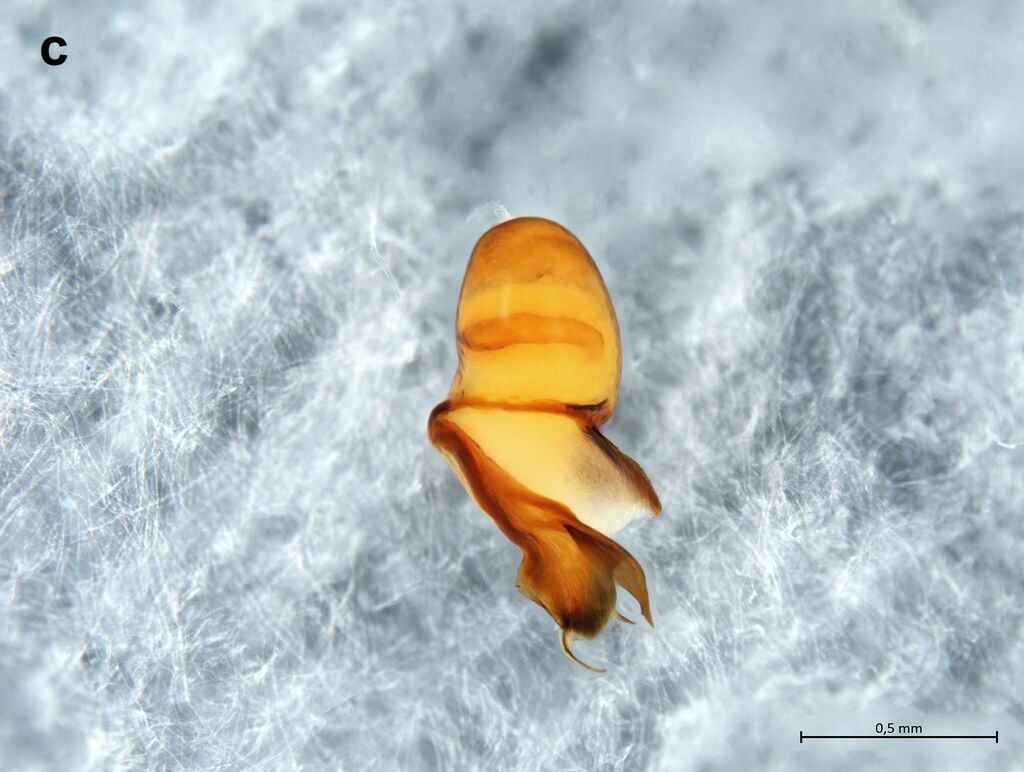
Pedipalp, anterior view;

**Figure 3d.**
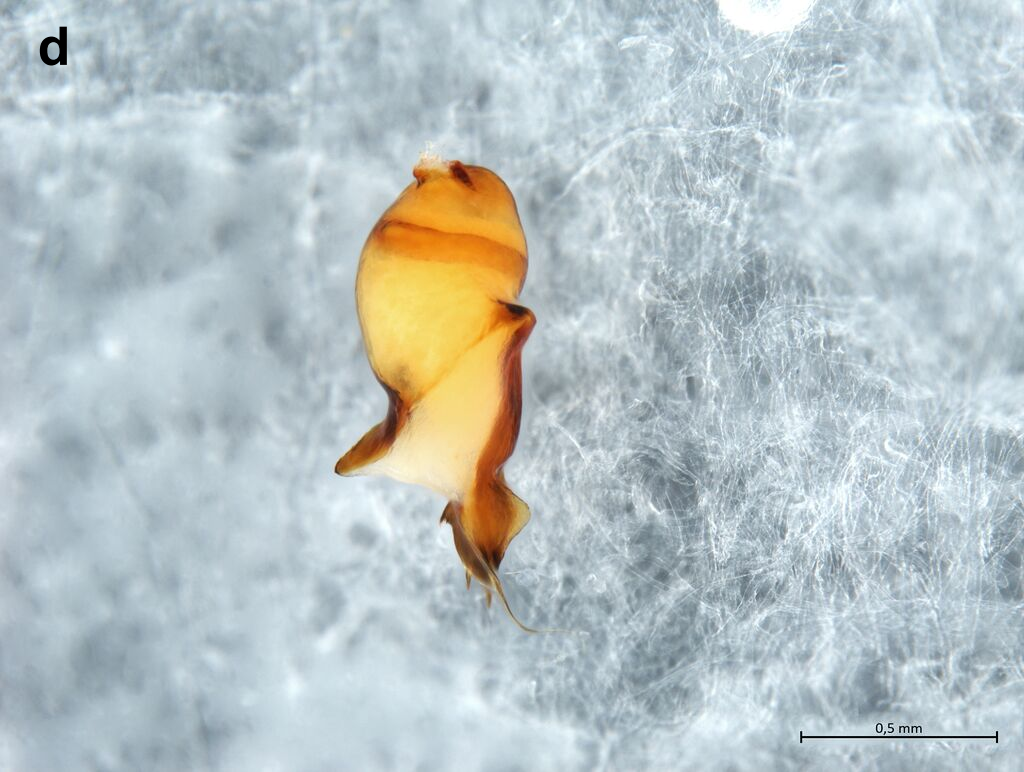
Pedipalp, posterior view.

**Figure 4a.**
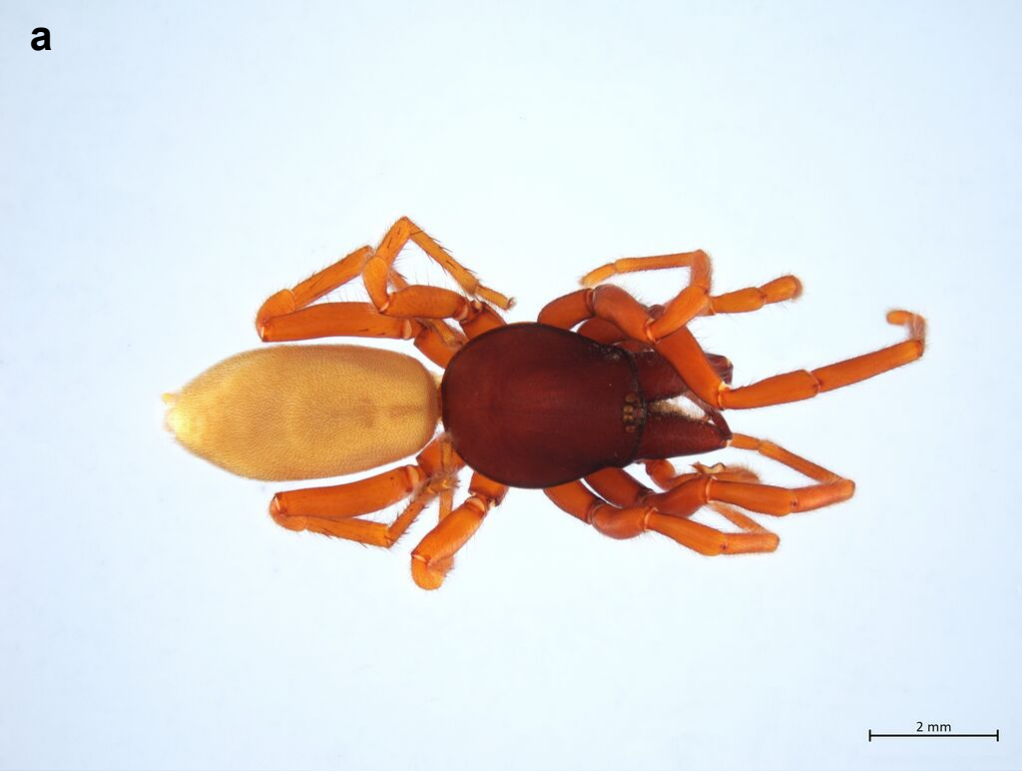
Habitus, dorsal view;

**Figure 4b.**
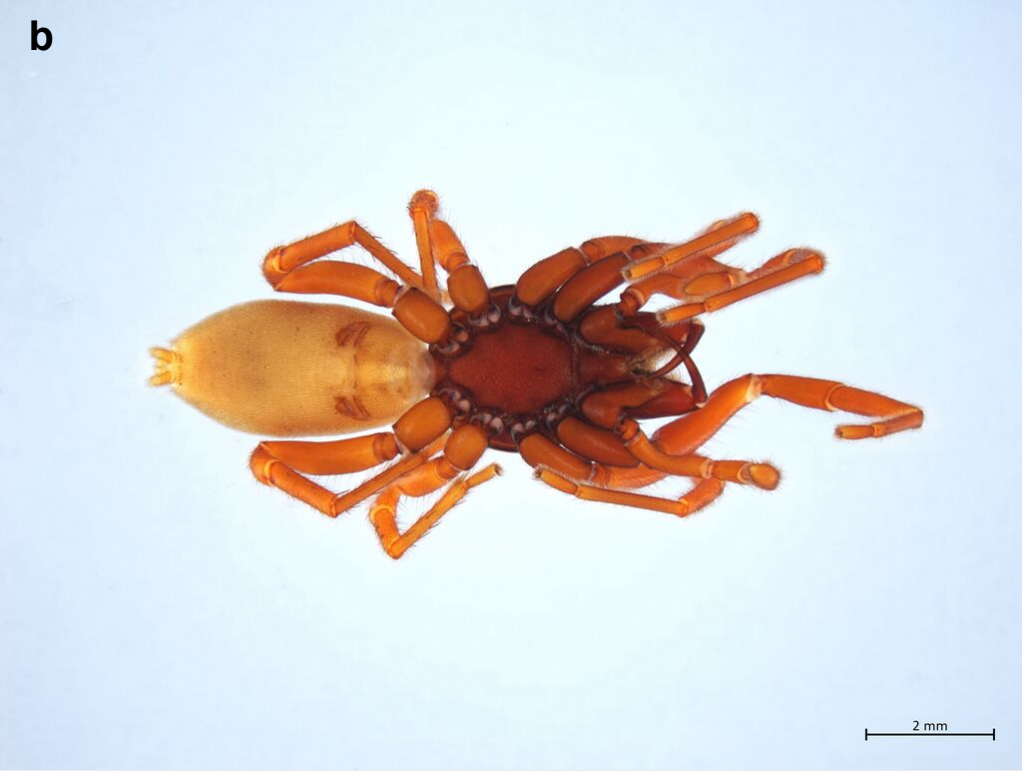
Habitus, ventral view.

**Figure 5a.**
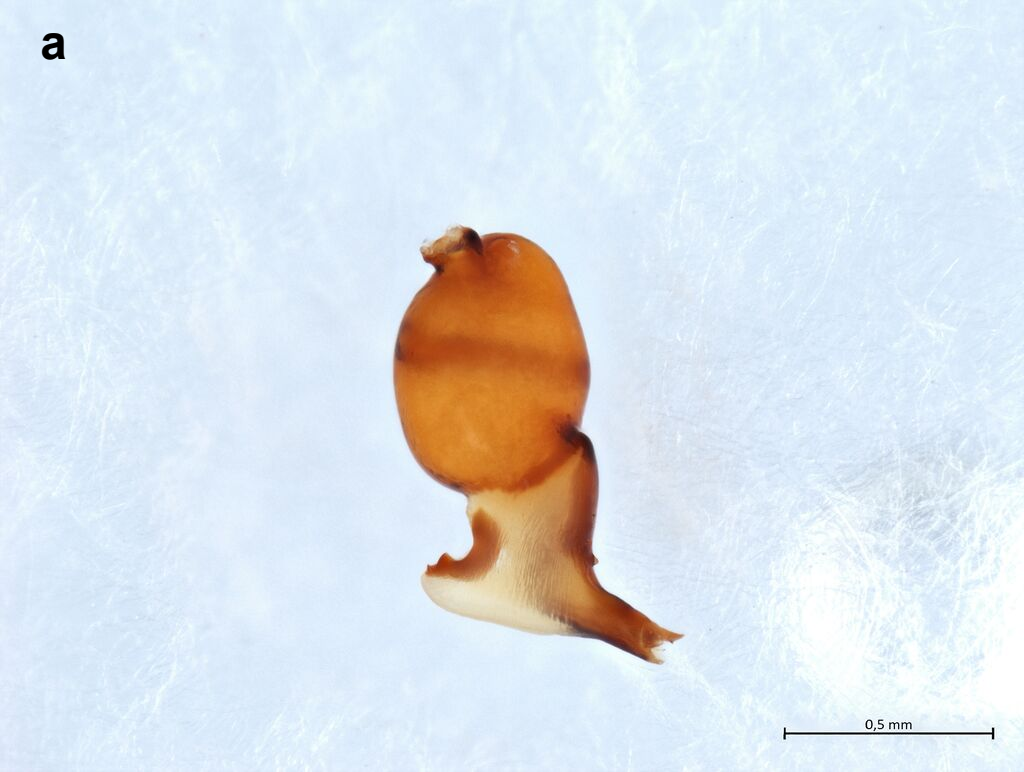
Pedipalp, left side view;

**Figure 5b.**
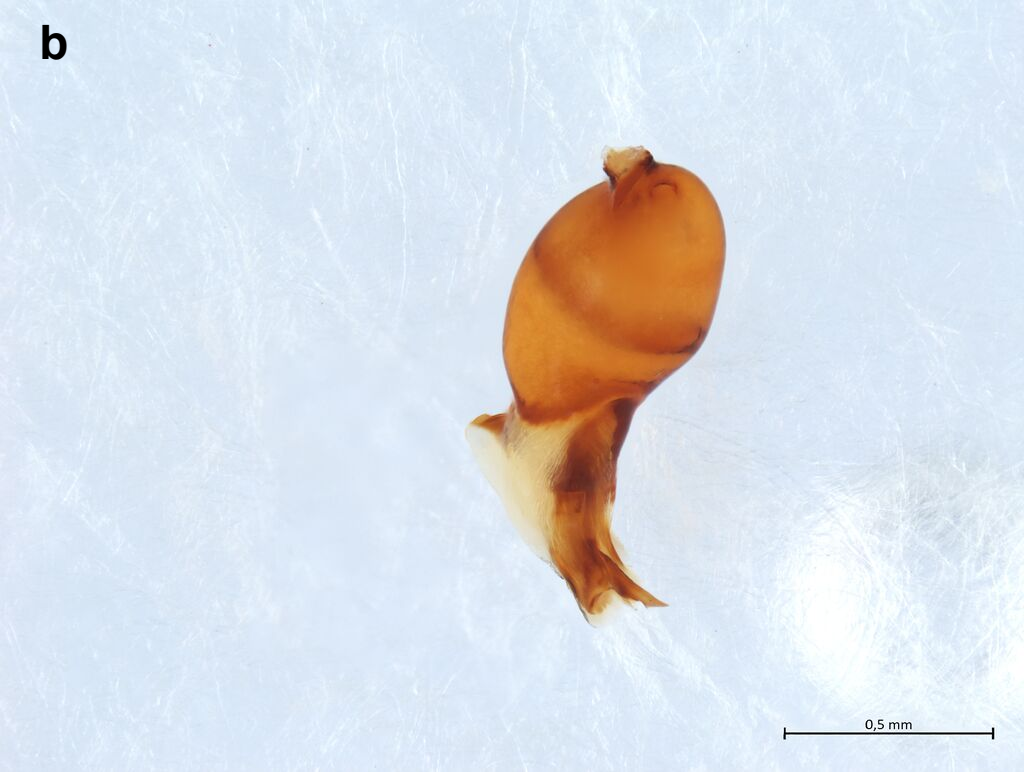
Pedipalp, postero-lateral view;

**Figure 5c.**
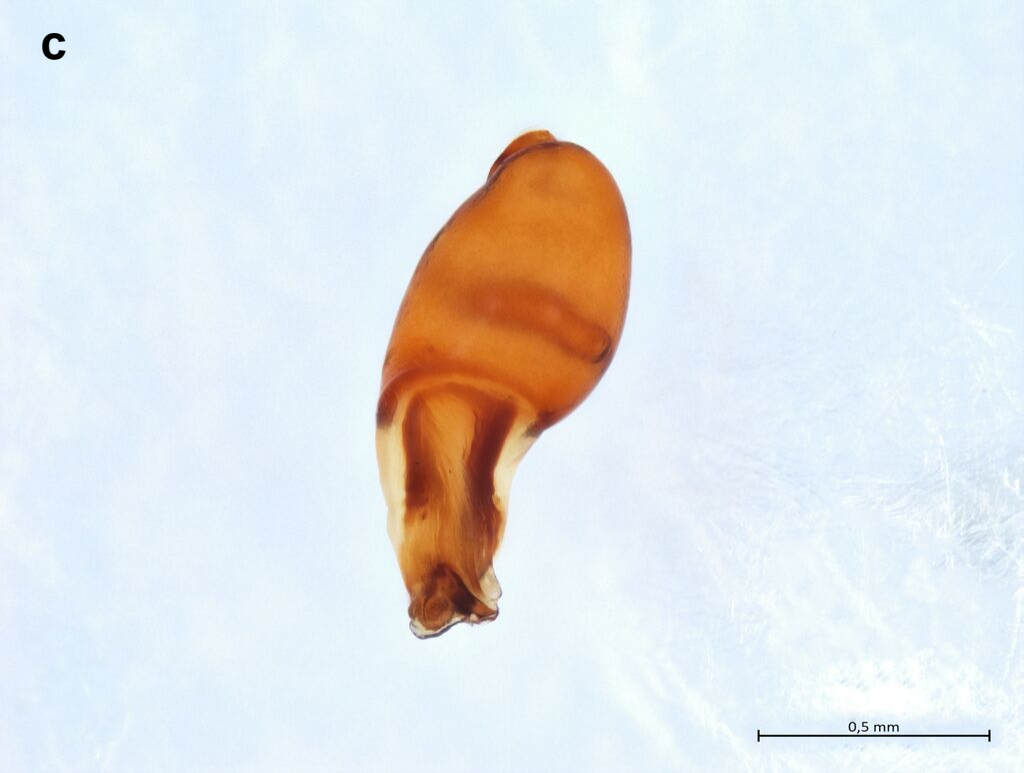
Pedipalp, posterior view;

**Figure 5d.**
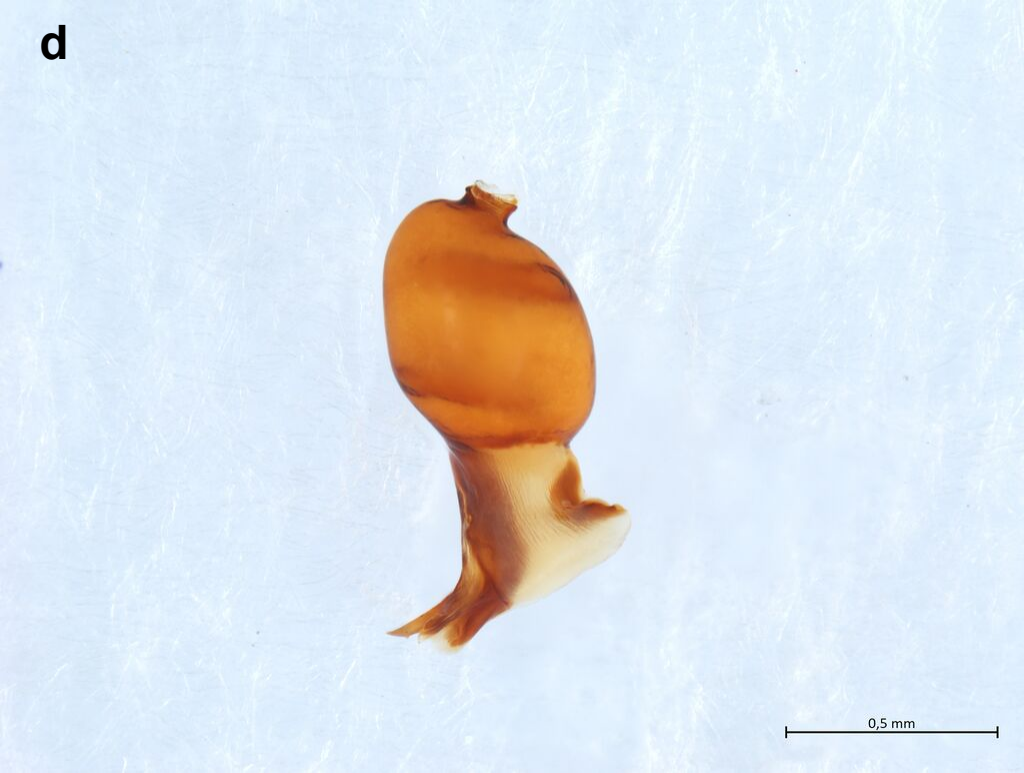
Pedipalp, right side view.

**Figure 6a.**
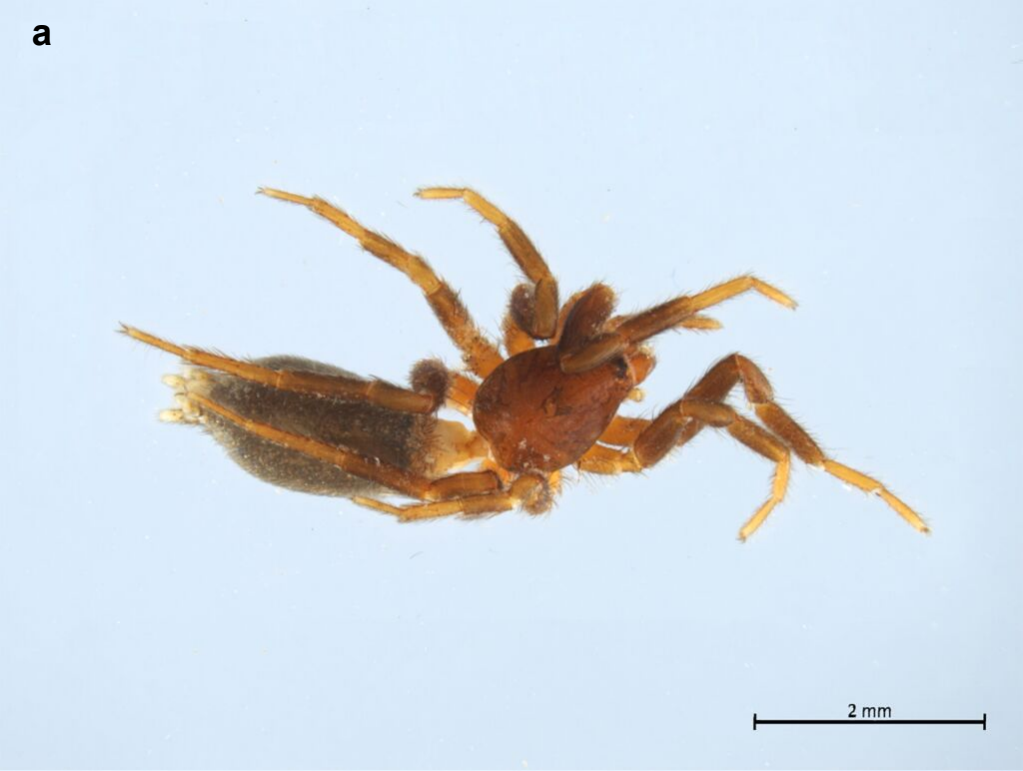
Habitus, dorsal view;

**Figure 6b.**
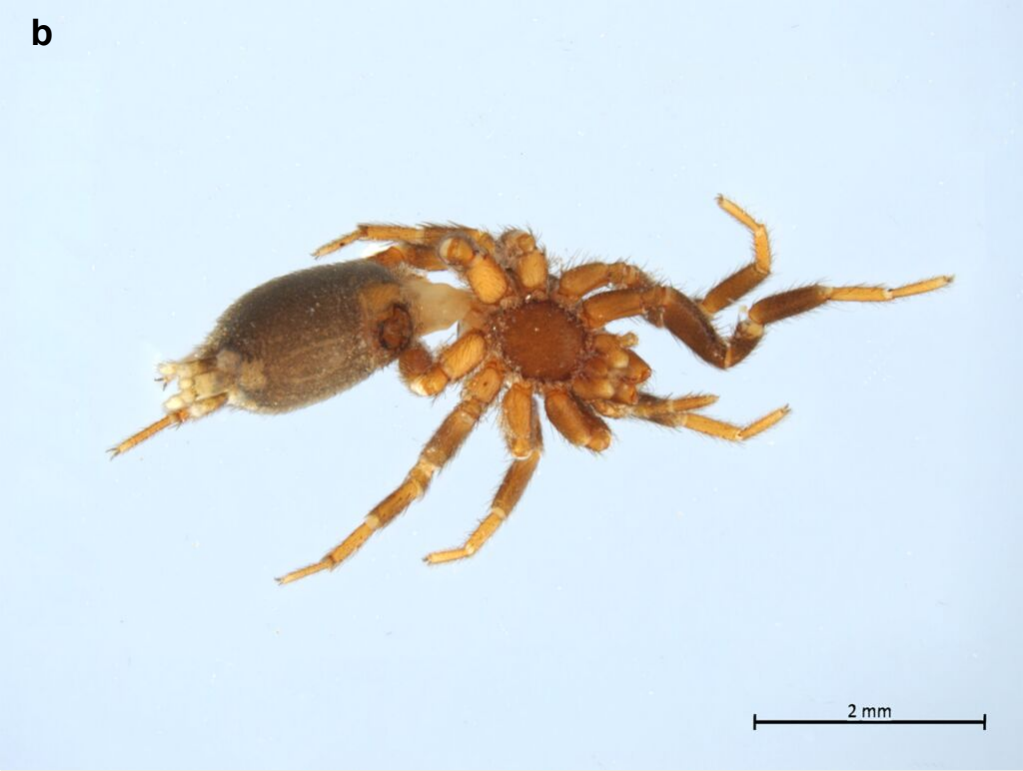
Habitus, ventral view.

**Figure 7a.**
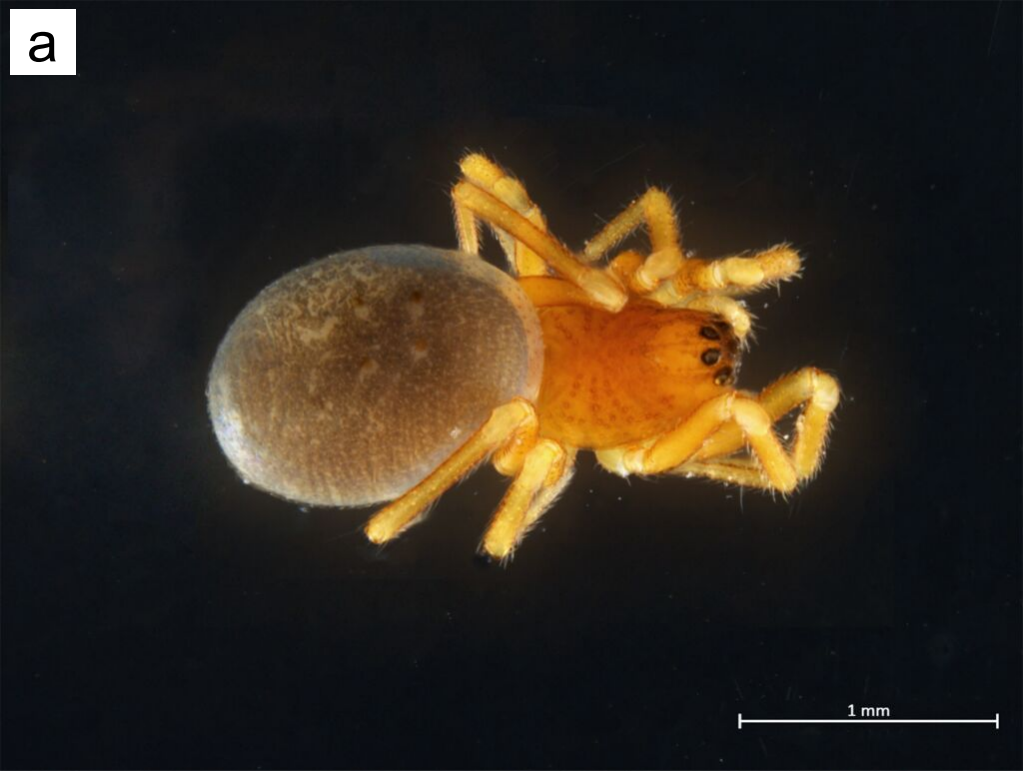
Habitus, dorsal view;

**Figure 7b.**
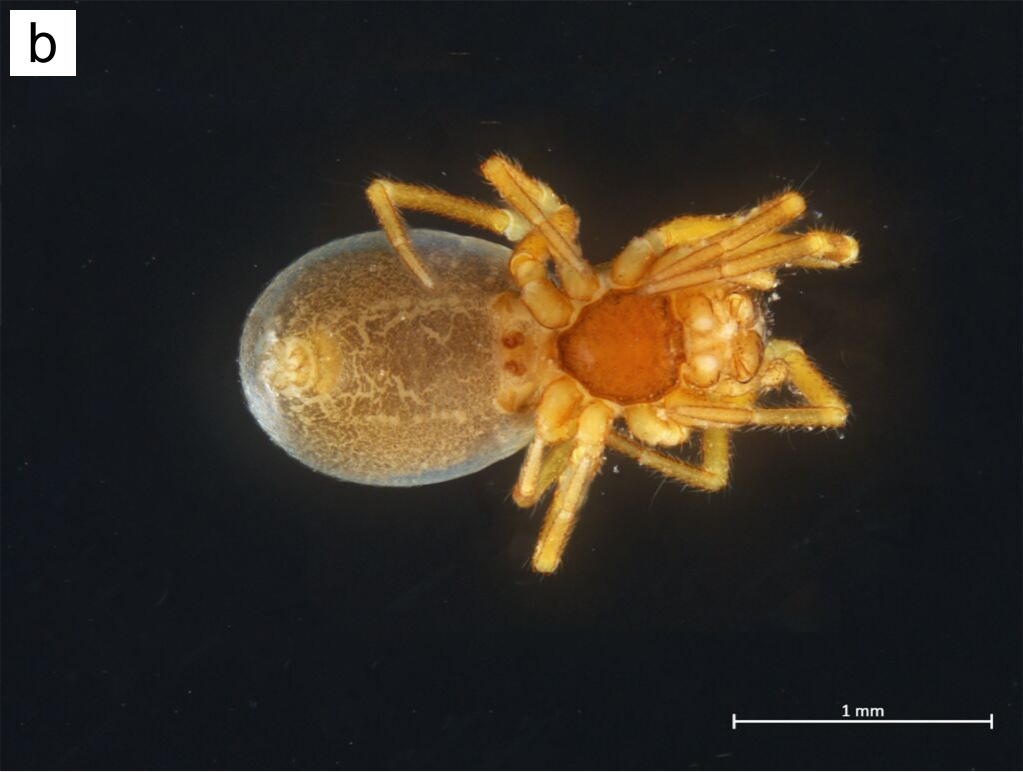
Habitus, ventral view.

**Figure 9a.**
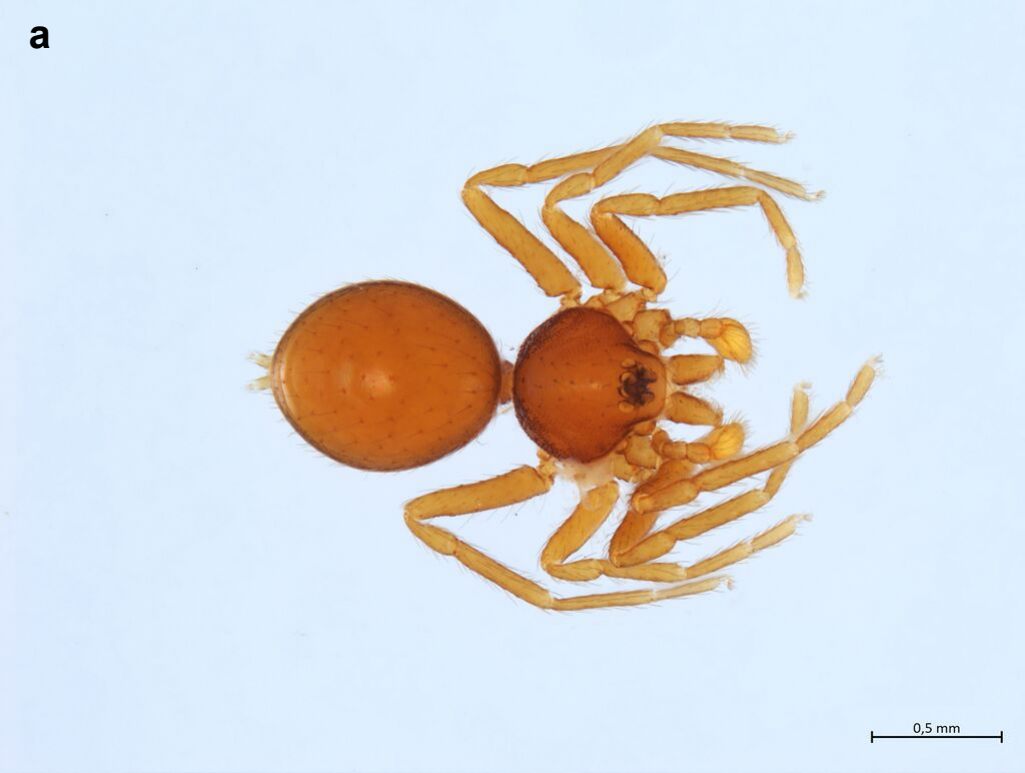
Habitus, dorsal view;

**Figure 9b.**
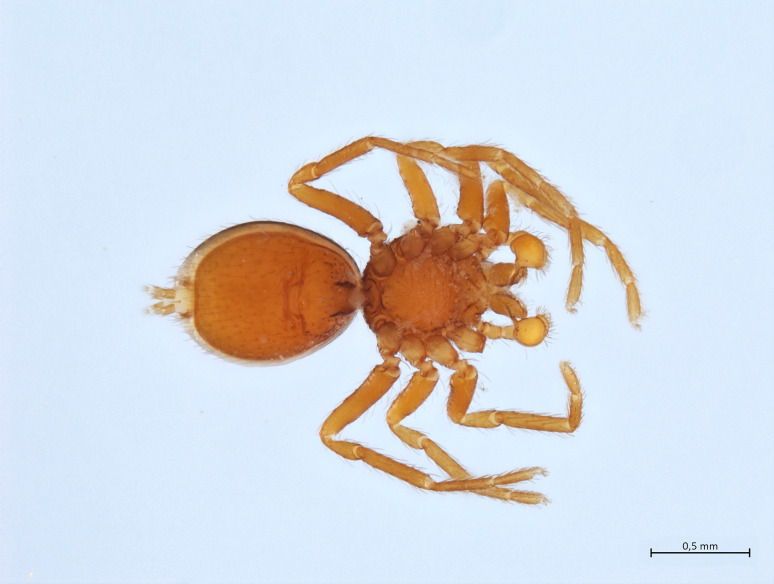
Habitus, ventral view.

**Figure 10a.**
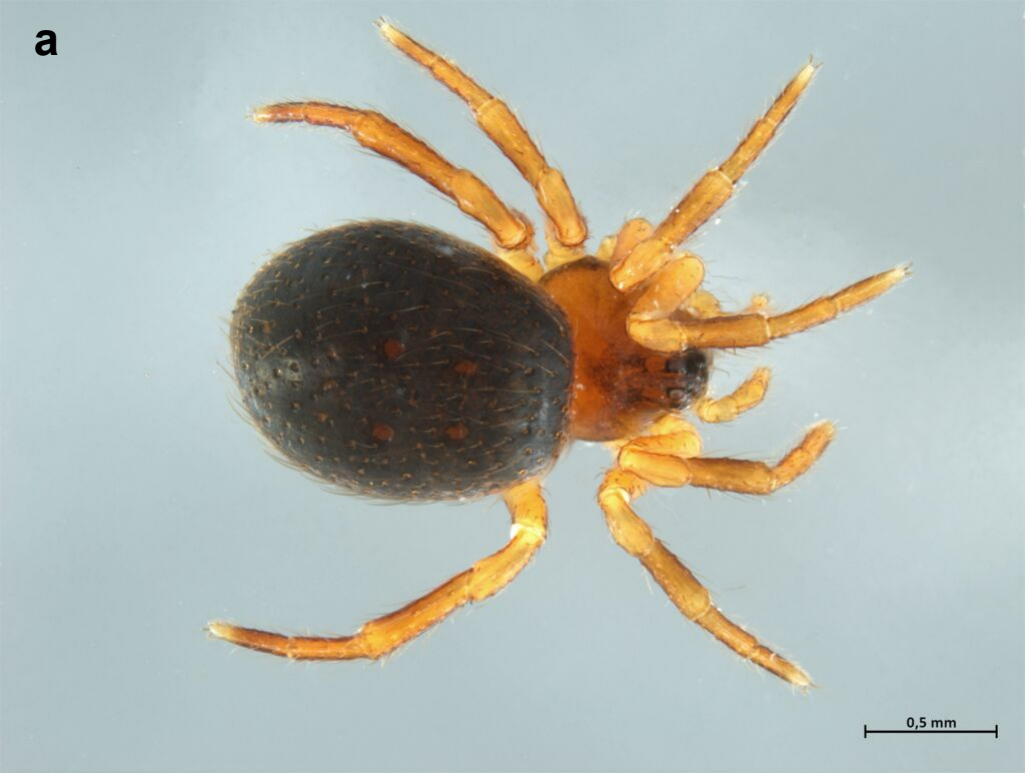
Habitus, dorsal view;

**Figure 10b.**
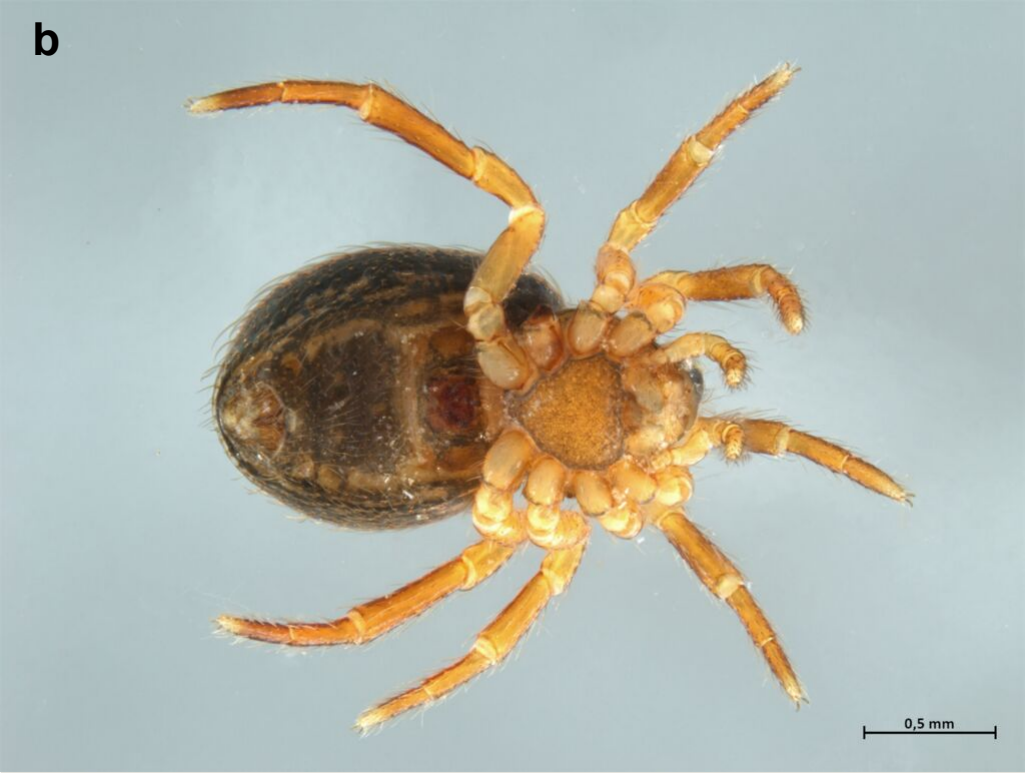
Habitus, ventral view.

**Figure 12a.**
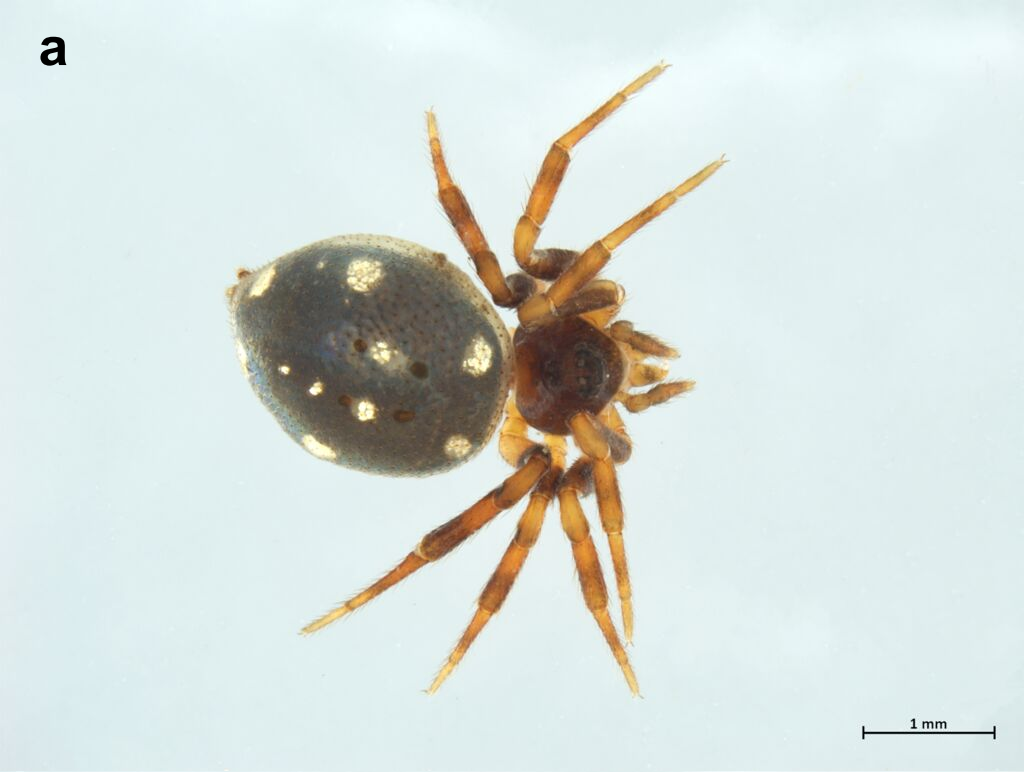
Habitus, dorsal view;

**Figure 12b.**
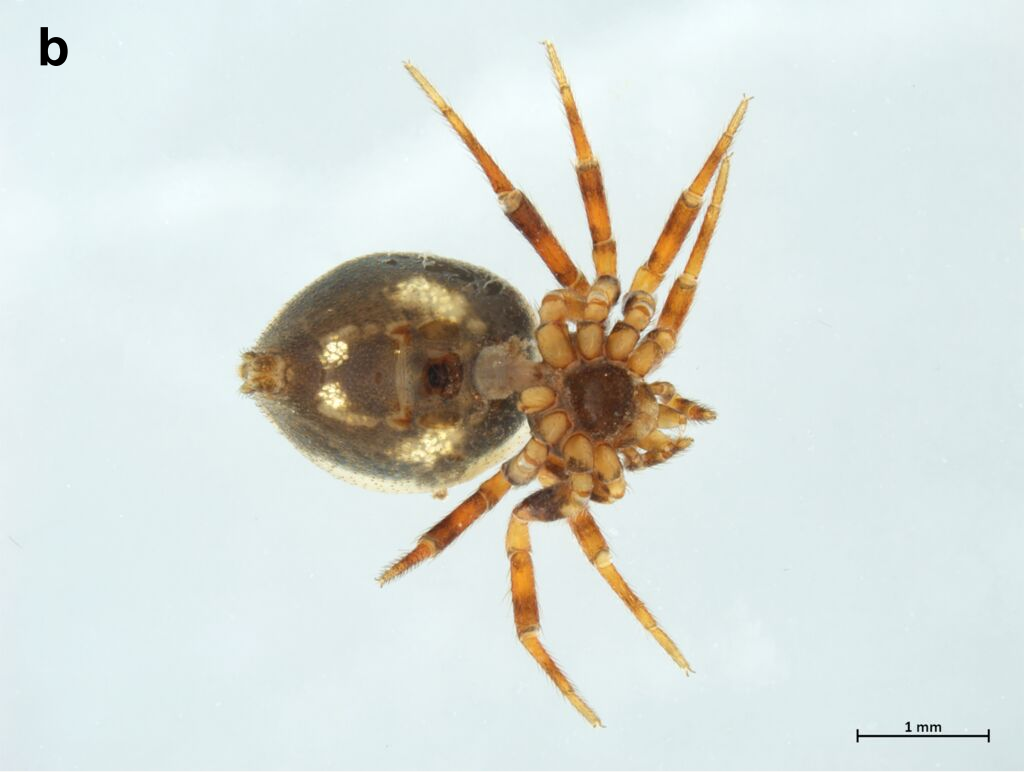
Habitus, ventral view.

**Table 1.** List of species sampled in the Appia Antica Regional Park.

**Familiy**	**Species**	**Novelty**
Agelenidae	*Lycosoidescoarcata*	
	*Tegenariadalmatica*	
	*Tegenariahasperi*	new for Rome
Amaurobiidae	*Amaurobiuserberi*	
Anapidae	*Zangherellaalgerica*	
Atypidae	*Atypusaffinis*	new for Latium, new for Rome
Cheiracanthiidae	*Cheiracantiummildei*	
Dictynidae	*Argennasubnigra*	new for Latium, new for Rome
Dysderidae	*Dysderacrocata*	
	*Dysderabottazziae*	new for Latium, new for Rome
	*Dysderakollari*	
	*Dysderalantosquensis*	
	*Dysderaromana*	
	*Harpacteasardoa*	new for Rome
Eresidae	*Eresuskollari*	
Gnaphosidae	*Anagraphisochracea*	
	*Haplodrassusdalmatensis*	
	*Haplodrassussignifer*	
	*Hesernilicola*	new for Rome
	*Leptodrassusalbidus*	new for Latium, new for Rome
	*Leptodrassusfemineus*	new for Latium, new for Rome
	*Marinarozelotesadriaticus*	
	*Marinarozelotesbarbatus*	
	*Marinarozeloteshuberti*	
	*Micariamicans*	new for Latium, new for Rome
	*Micariapallipes*	new for Latium, new for Rome
	*Nomisiaexornata*	
	*Phaeocedusbraccatus*	
	*Setaphiscarmeli*	
	*Trachyzelotespedestris*	
	*Turkozelotesnoname*	new for Latium, new for Rome
	*Urozelotesrusticus*	new for Latium, new for Rome
	*Zelotesatrocaeruleus*	
	*Zelotesfemellus*	
	*Zelotestenuis*	
Hahniidae	*Iberinacandida*	new for Rome
Linyphiidae	*Agynetafuscipalpa*	new for Rome
	*Agynetamollis*	new for Latium, new for Rome
	*Alioranuspauper*	new for Rome
	*Araeoncushumilis*	new for Latium, new for Rome
	*Araeoncuslongiusculus*	new for Rome
	*Centromerussylvaticus*	new for Latium, new for Rome
	*Centromerustongiorgii*	
	*Ceratinellabrevis*	new for Latium, new for Rome
	*Diplocephalusgraecus*	new for Rome
	*Diplostylaconcolor*	new for Latium, new for Rome
	*Erigoneautumnalis*	new for Latium, new for Rome
	*Erigonedentipalpis*	
	*Gonatiumbiimpressum*	new for Rome
	*Mecopistheslatinus*	
	*Microctenonyxsubitaneus*	new for Rome
	*Micronetaviaria*	new for Latium, new for Rome
	*Oedothoraxpaludigena*	
	*Osteariusmelanopygius*	new for Latium, new for Rome
	*Ouediarufithorax*	new for Rome
	*Palliduphantesarenicola*	new for Latium, new for Rome, new for Italy
	*Palliduphantesbyzantinus*	new for Latium, new for Rome
	*Palliduphantesistrianus*	
	*Pelecopsisdigitulus*	new for Latium, new for Rome, new for Italy
	*Prinerigonevagans*	new for Rome
	*Scutpelecopsiskrausi*	new for Latium, new for Rome
	*Sintularetroversus*	new for Latium, new for Rome
	*Syedranigrotibialis*	new for Rome
	*Tenuiphantesherbicola*	new for Rome
	*Tenuiphantestenuis*	
	*Trichoncusaffinis*	new for Latium, new for Rome
	*Trichoncushackmani*	new for Latium, new for Rome
	*Trichoncussordidus*	new for Rome
	*Walckenaeriaantica*	new for Latium, new for Rome
Liocranidae	*Agraecinalineata*	new for Latium, new for Rome
	*Agroecacuprea*	new for Rome
	*Cybaeodesmarinae*	
Lycosidae	*Alopecosaalbofasciata*	
	*Arctosapersonata*	new for Rome
	*Auloniaalbimana*	
	*Pardosaprativaga*	new for Latium, new for Rome
	*Pardosaproxima*	
	*Trochosahispanica*	new for Rome
Miturgidae	*Zoraspinimana*	new for Rome
Nemesiidae	*Nemesiabosmansi*	new for Rome
Nesticidae	*Kryptonesticuseremita*	
Oecobiidae	*Oecobiusmaculatus*	new for Latium, new for Rome
	*Oecobiusnavus*	new for Rome
Oonopidae	*Orchestinalongipes*	new for Rome
	*Silhouettellaloricatula*	
Philodromidae	*Philodromusrufus*	
	*Pulchellodromusbistigma*	new for Rome
Phrurolithidae	*Liophrurillusflavitarsis*	
	*Phrurolithusminimus*	new for Rome
Salticidae	*Aelurilusv-insignitus*	new for Latium, new for Rome
	*Euophrysfrontalis*	new for Rome
	*Euophrysrufibarbis*	new for Latium, new for Rome
	*Euophryssulfurea*	new for Latium, new for Rome
	*Euophrysterrestris*	new for Latium, new for Rome
	*Evarchajucunda*	
	*Philaeuschrysops*	new for Latium, new for Rome
	*Phlegrabresnieri*	
	*Pseudeuophryscfrperdifumo*	new for Latium, new for Rome
	*Pseudeuophrysvafra*	
Scytodidae	*Scytodesthoracica*	
Sparassidae	*Oliosargelasius*	
Tetragnathidae	*Pachygnathadegeeri*	
Theridiidae	*Asagenaitalica*	new for Rome
	*Crustulinaguttata*	new for Latium, new for Rome
	*Crustulinascabripes*	new for Rome
	*Enoplognatamandibularis*	
	*Enoplognatatestacea*	new for Rome
	*Enoplognatathoracica*	new for Rome
	*Euryopisdentigera*	new for Latium, new for Rome
	*Euryopisepisinoides*	
	*Euryopiscfrsexalbomaculata*	new for Latium, new for Rome
Thomisidae	*Bassanoides* sp.	
	*Ozyptilaconfluens*	new for Rome
	*Ozyptilapraticola*	new for Latium, new for Rome
	*Ozyptilasanctuaria*	new for Rome
	*Xysticuskochi*	
Titanoecidae	*Titanoecaflavicoma*	new for Latium, new for Rome
Zodariidae	*Zodarionelegans*	
	*Zodarionitalicum*	
	*Zodarionpusio*	
